# Propellane Alkaloid Biosynthesis and Total Synthesis via Interrupted Reaction Pathways

**DOI:** 10.1021/acscentsci.6c00057

**Published:** 2026-04-03

**Authors:** John M. Billingsley, Jiaming Ding, Allison T. Hands, Kanji Niwa, Nathan J. Adamson, Lukas A. Wein, Bruno Perlatti, Neil K. Garg, Yi Tang

**Affiliations:** † Department of Chemical and Biomolecular Engineering, 8783 University of California, Los Angeles, California 90095, United States; ‡ Department of Chemistry and Biochemistry, 8783 University of California, Los Angeles, California 90095, United States

## Abstract

Interrupted reactions, in which an intermediate is redirected from its conventional mechanistic pathway, offer a unique approach to the assembly of complex natural products. This study details the biosynthesis of a newly discovered family of alkaloids named the subrubines alongside the total synthesis of the penultimate member, pensubrubine, featuring distinct interrupted reaction pathways. These natural products, identified using high-resolution genome mining of an active site mutation, represent the only reported microbial diaza[3.3.3]­propellane pyrrolidinoindolines. We demonstrate through complete pathway reconstitution that the putatively annotated ene-reductase SubF functions as the propellane synthase that directs an enolate intermediate toward an intramolecular Mannich cyclization. Concurrently, a concise 7-step total synthesis of pensubrubine was developed, employing a diastereoselective interrupted Fischer indolization reaction to rapidly construct the diaza[3.3.3]­propellane core and establish the absolute configuration of pensubrubine. The reported bio- and total syntheses of subrubines showcase the value of interrupted pathways in assembling complex scaffolds.

## Graphical abstract

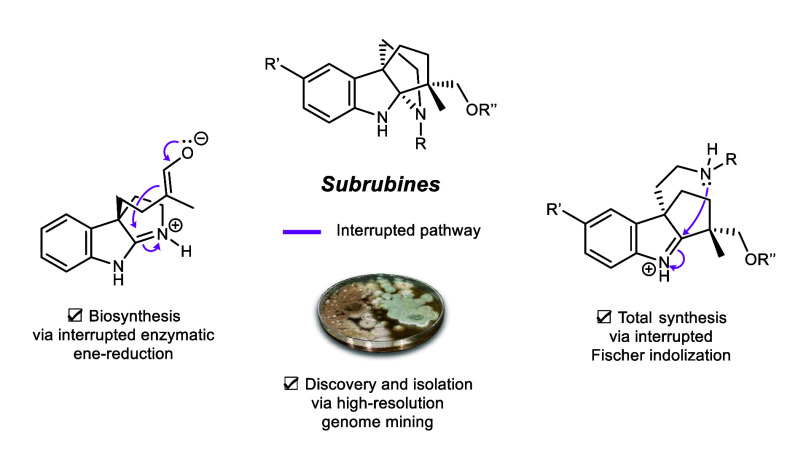

## Introduction

Interrupted reactions, in which an intermediate is redirected from its conventional mechanistic pathway, are powerful approaches for constructing complex scaffolds.^1^ As reviewed by Yudin and co-workers, several types of interrupted processes exist including: (a) pathways in which an intermediate is redirected from a conventional pathway to a more favorable alternative, (b) scenarios where a given intermediate takes on a new route owing to a conventional pathway no longer being available, (c) new pathways that simply diverge from a reactant, and (d) processes in which a change in reaction conditions leads to an intermediate in a conventional pathway undergoing a different reaction outcome. Interrupted reactions have been leveraged by synthetic organic chemists in method development and natural product synthesis, offering great potential for future use. Interrupted enzymatic pathways are also documented, wherein active site mutations reroute canonical intermediates to generate novel structures^2–7^ or, more broadly, any scenario in which a biosynthetic intermediate is redirected from a more conventional pathway. In the present study, we discovered the subrubine family of indole alkaloids (Figure 1a, **1–6**), which are the first examples of microbial-derived pyrrolidinoindoline natural products containing a diaza[3.3.3]­propellane scaffold. High-resolution mining of ene-reductases for mutation of a highly conserved tyrosine residue and comparative multiomics enabled identification of the subrubine biosynthetic gene cluster (BGC). We subsequently elucidate the biosynthesis of these natural products, uncovering an interrupted ene-reduction that results in a reductive Mannich cyclization to build the azapropellane core. Simultaneously, a concise 7-step total synthesis of pensubrubine (**4**) was developed, which relies on a mechanistically distinct interrupted process (i.e., interrupted Fischer indolization) to rapidly construct the aforementioned azapropellane core bearing three contiguous fully substituted stereocenters.

**Figure 1:**
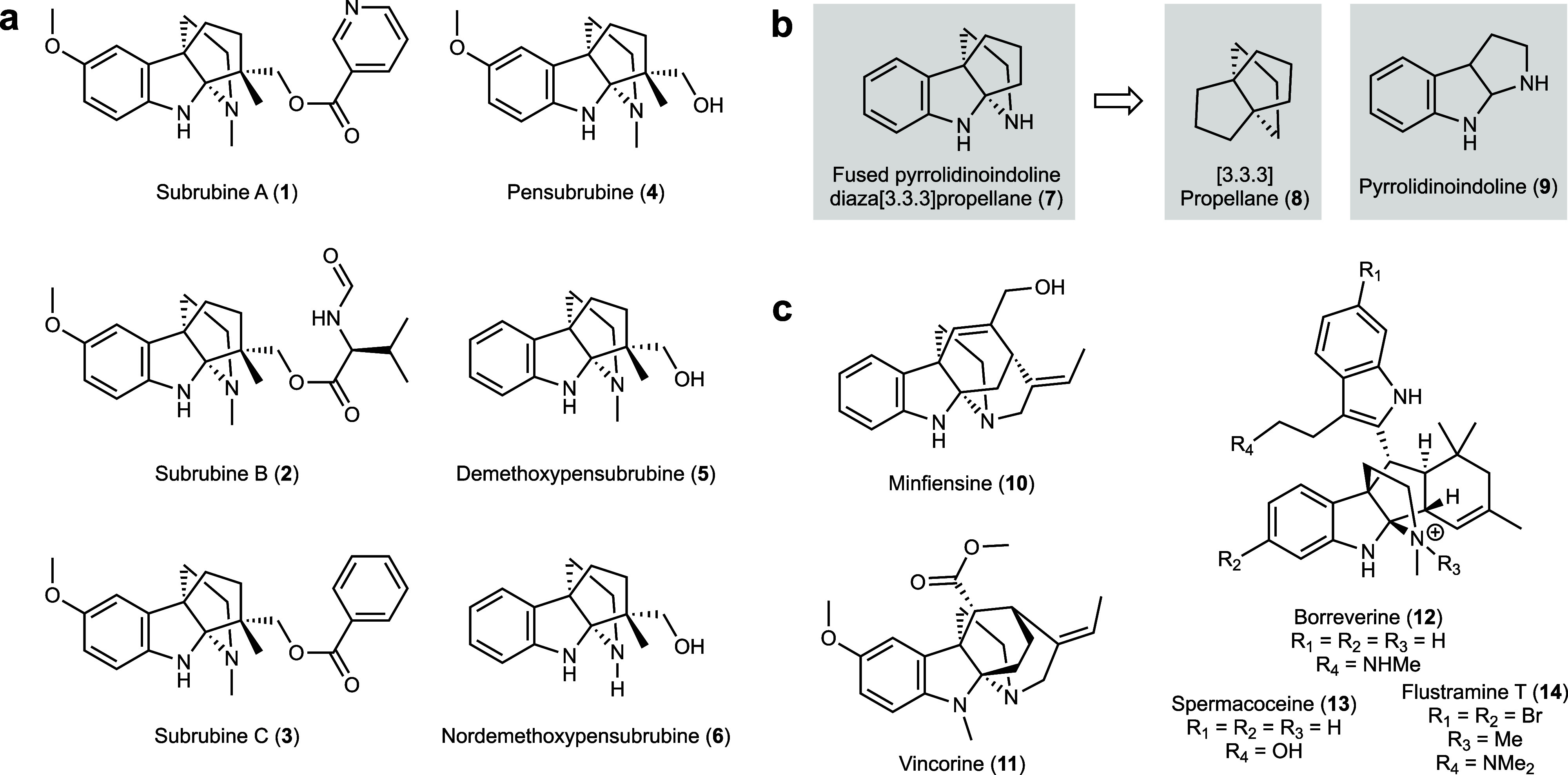
**Propellane pyrrolidinoindolines. a,** Subrubine family of indole alkaloids (**1**–**6**) discovered in this study. **b,** Core diaza[3.3.3]­propellane structure (**7**) of the subrubine family. **c,** Examples of pyrrolidinoindoline natural products possessing azapropellane scaffolds.

The subrubine natural products (Figure 1a) feature embedded propellane and pyrrolidinoindoline units, as seen in core structure **7** (Figure 1b). Propellanes, as exemplified by [3.3.3]­propellane **8**, are molecules in which three rings share a single carbon–carbon bond, and have captivated chemists since their description in the 1960s.^8–10^ Pyrrolidinoindoline alkaloids, of which there are over 500 reported examples, are represented by structure **9**. These are known for their intriguing structures and a breadth of bioactivities.^11,12^ Examples of natural products containing both propellane and pyrrolidinoindoline frameworks are minfiensine (**10**) and vincorine (**11**). However, there are only three reported natural products that possess a more compact diaza[3.3.3]­propellane pyrrolidinoindoline core: borreverine (**12**), spermacoceine (**13**), and flustramine T (**14**) (Figure 1c).^13–15^ Few chemical methods exist to synthesize these rare structures,^16–21^ and limited knowledge regarding the biosynthesis of their compact propellane core has been disclosed.^22^ Thus, following the discovery of subrubines, we elucidated the full biosynthetic pathway and explored synthetic access. Central to both the bio- and chemical syntheses of the diaza[3.3.3]­propellane pyrrolidinoindoline core was the application of interrupted reactions.

## Results and Discussion

### Discovery and Isolation of Subrubines

As noted earlier, interrupted enzymatic reactions are common if one considers a broad definition in which a biosynthetic intermediate is redirected from a more conventional pathway. One elegant example of an interrupted enzymatic reaction occurs during the lysergic acid biosynthesis as described by O’Connor and Panaccione, wherein a predicted ene-reductase EasA catalyzes *E/Z* isomerization of an enal intermediate instead of the canonical conjugate reduction (Figure S1).^5,23,24^ In the EasA reaction, protonation of an enolate is drastically impeded due to absence of an acidic tyrosine in the active site, which reroutes the enolate intermediate toward σ-bond rotation and hydride removal by an oxidized flavin cofactor (Figure 2a). Replacement of this tyrosine with phenylalanine (designated Y196F in the model yeast ene-reductase) was shown to decrease the canonical reaction rate by 6 orders of magnitude.^25^ Interrupted ene-reduction has also been leveraged in biocatalytic C–C bond forming reactions.^26,27^ In 2018, Breinbauer and co-workers described an enzymatic reductive carbocyclization catalyzed by an engineered ene-reductase, in which installation of the active site Tyr-to-Phe mutation effectively redirected the enolate toward alkylation with a pendant haloalkane to form cyclized products (Figure 2a).^7^ Given the diverse reactivity profile of enolates and the ubiquity of oxidoreductases in natural product BGCs,^28–30^ we hypothesized that these enzymes could facilitate additional interrupted transformations in natural product biosyntheses beyond the reactions described above. Informed by early studies that demonstrated the pivotal role of the active-site Tyr-to-Phe mutation, we proposed a genome mining approach underpinned by high-resolution analysis of the Y196 residue in ene-reductases. This could potentially lead to the identification of novel biocatalytic processes and, ultimately, discovery of natural products possessing unique structures.

**Figure 2:**
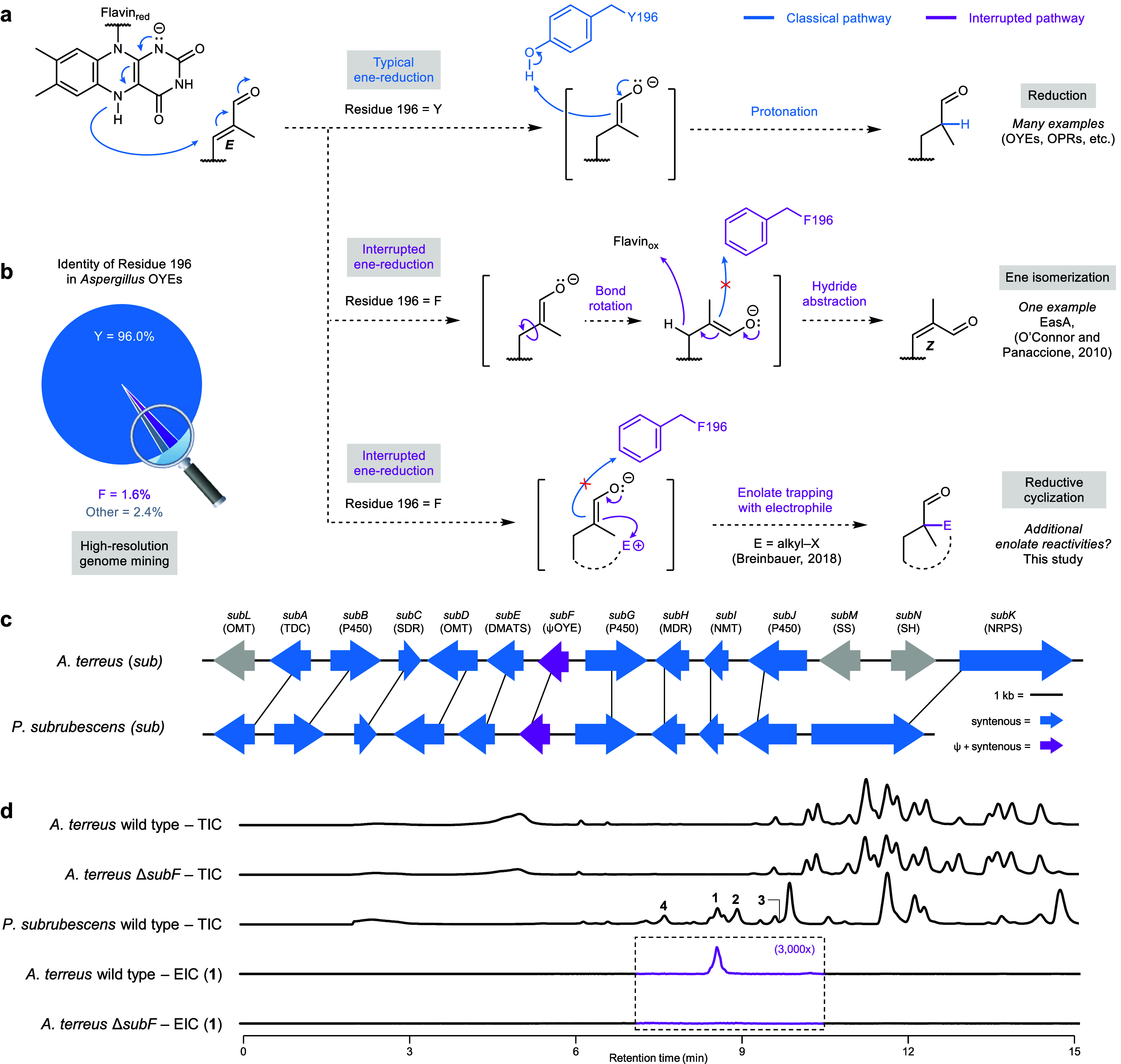
**Genome mining for interrupted biochemistry. a,** Outcomes of typical and interrupted ene-reduction reactions performed by ene-reductases. Residue numbering corresponds to yeast OYE. **b,** Comparison of the identity of residue 196 in *Aspergillus* OYEs. **c,** Subrubine (*sub*) biosynthetic gene clusters. OMT, *O*-methyltransferase; TDC, tryptophan decarboxylase; SDR, short-chain dehydrogenase/reductase; DMATS, dimethylallyl tryptophan synthase; OYE, old yellow enzyme; MDR, medium-chain dehydrogenase/reductase; NMT, *N*-methyltransferase; SS, salicylate synthase; SH, salicylate hydroxylase; NRPS, nonribosomal peptide synthetase. **d,** Analysis of *A. terreus* metabolites via comparative metabolomics by HPLC-MS; EIC of 394.2125 under positive ionization is shown.

We performed a phylogenetic analysis of OYEs (“old yellow enzymes” – the canonical yeast ene-reductase) within *Aspergillus* genomes (Figures S2–S5) and inspected the position corresponding to Y196 for mutations.^31,32^ A subcollection of predicted noncanonical-OYEs (ψOYEs) emerged with the Y196F substitution, each coclustered with a nonribosomal peptide synthetase (NRPS) in the respective BGCs. All but one of the BGCs were homologous to lysergic acid-producing *eas* BGCs^33^ or diketopiperazine-producing *lnb* BGCs.^34^ The remaining uncharacterized BGC (*sub*) is present in the widely studied *Aspergillus terreus* NIH2624 (Figure 2c), with a syntenous BGC found in just two other organisms in the NCBI database, *Penicillium subrubescens* and *Penicillium ochrochloron.* Conserved enzymes encoded in all three BGCs include a tryptophan decarboxylase (TDC, SubA), three P450 monooxygenases (SubB, SubG and SubJ), two alcohol dehydrogenases (SubC and SubH), ψOYE (SubF), dimethylallyl tryptophan synthase (DMATS, SubE), two methyltransferases (SubD and SubI) and a single-module NRPS (A-T-C, SubK). While the *sub* BGC shares six homologous genes with the *eas* BGC, no homologues to EasE (BBE-like oxidase) or EasC (catalase) – the two enzymes required to construct the indole-fused carbocycle in lysergic acid^35,36^ – are present.

Comparative metabolomics was used to determine the natural product associated with *sub* BGC. CRISPR-Cas9-mediated deletion^37,38^ of the ψOYE-encoding *A. terreus subF* was performed (Figure S6) followed by untargeted metabolite analysis on CD agar (Figure 2d). However, no discernible difference above noise was observed in extracts from the wild-type and Δ*subF* strains. We therefore examined the metabolite profile of *P. subrubescens*, a fungus that has attracted interest due to its ability to produce high amounts of inulinase.^39^ The strain was cultured on CD agar and produced >20 metabolites detectable by LC-MS. The *m*/*z* values found in *P. subrubescens* extracts were used as targeted metabolomics queries in *A. terreus* (Figure S7). Gratifyingly, one metabolite **1** produced by *P. subrubescens* (∼11 mg/L) was produced in trace amounts (∼0.02 mg/L) by the wild-type *A. terreus*, but was absent from the Δ*subF* strain (Figure 2d). Despite ∼350 natural products have been isolated from *A. terreus* strains,^40^ the low titer of **1** prevented its identification via untargeted analysis and may account for its absence from prior reports.

As the BGC encoded tryptophan-related enzymes, supplementation of *P. subrubescens* cultures with l-tryptophan-(*indole*-d_5_) (Figure S9) resulted in deuterium incorporation into four major (**1**, **2**, **3**, and **4**) and two minor metabolites (**5** and **6**). Growth on *Helianthus tuberosus* (sunchokes), the tuber from which *P. subrubescens* was isolated, led to high-level production (Figure S10), enabling purification and characterization of **1**–**4** (Figures S32–S55, Tables S8–S11). The isolated compounds contain a diaza[3.3.3]­propellane fused pyrrolidinoindoline, methoxylated at C5 of the indoline, and *N-*methylated at the pyrrolidine nitrogen. Pensubrubine (**4**) contains a hydroxymethyl substituent in the fused cyclopentane, while this position in subrubine A (**1**), subrubine B (**2**), and subrubine C (**3**) is esterified with nicotinic acid, *N-*formyl-l-valine, and benzoic acid, respectively. 2D NMR experimentation was used to establish connectivities (HSQC/HMBC/COSY) and relative configurations (NOESY/ROESY) of all newly described subrubine congeners, with absolute configuration of the C3–C2–C3′ stereotriad ultimately deduced by enantioselective total synthesis of (−)-**4** (*vide infra*). The diaza[3.3.3]­propellane present in **1–4** represents the first example of this structure found in a microbial natural product.

### *In Vivo* and *In Vitro* Pyrrolidinoindoline Construction

Based on the structures of **1–4** and predicted functions of the *sub* biosynthetic enzymes, retrobiosynthetic analysis was performed (Figure S11). The adenylation (A) domain of NRPS SubK is predicted to activate nicotinic acid, *N*-formyl-l-valine, or benzoic acid, generating a T-domain phosphopantetheinyl thioester, followed by condensation (C) domain-catalyzed esterification with **4** to form **1**, **2**, or **3**, respectively.^41–43^ We propose that the oxygen atoms in **4** are inserted via reactions catalyzed by two of the three P450s (SubB, SubG, and SubJ), while the *O-* and *N-*methyl groups are installed by the two methyltransferases (SubD and SubI). Such analysis enabled the prediction of **6** as the unmodified and earliest isolable propellane intermediate in the pathway. Decarboxylation of l-tryptophan by SubA (TDC) and prenylation by SubE (DMATS) could be the initial biosynthetic steps, with subsequent modifications by remaining unassigned enzymes, including the ψOYE SubF, to construct the propellane core.

To map the individual biosynthetic steps, bottom-up pathway reconstitution was performed in *A. nidulans* ΔSTΔEM^44,45^ (Figures 3a and 4a). Expression of SubA (TDC) resulted in accumulation of ∼18 mg/L indole-3-acetic acid (Figure S12), a metabolite derived from tryptamine via the TAM pathway (Figure S13).^46^ Expression of SubE (DMATS) yielded a new compound (**15**) at trace levels with a mass corresponding to prenylated tryptamine, hinting that SubE can act on endogenous tryptamine in the host and should follow SubA in the biosynthetic sequence. To identify compound **15**, coexpression of *subA* and *subE* was performed in *S. cerevisiae* and *A. nidulans*, resulting in titers of **15** reaching 0.6 and 0.1 mg/L respectively (Figures 4a and S14). Isolation of the compound from yeast was performed and NMR analysis was carried out (Figures S68–S73 and Table S14) which revealed **15** to be the C3-prenylated pyrrolidinoindoline (Figure 3a). SubE therefore catalyzes a reaction analogous to AnaPT^47^ and ComQ,^48^ during which an indolinium is generated via stereoselective prenylation at indole C3, followed by intramolecular aminoethyl nitrogen capture.^49^ In these reported pyrrolidinoindoline forming transferases (PFTs), C3 alkylation and cyclization occur after tryptophan is incorporated into a peptidyl natural product (Figures S16–S17).^50–53^ In contrast, SubE is the first PFT identified acting on freestanding tryptamine and is therefore a potential biocatalyst for construction of substituted pyrrolidinoindoline building blocks.

**Figure 3:**
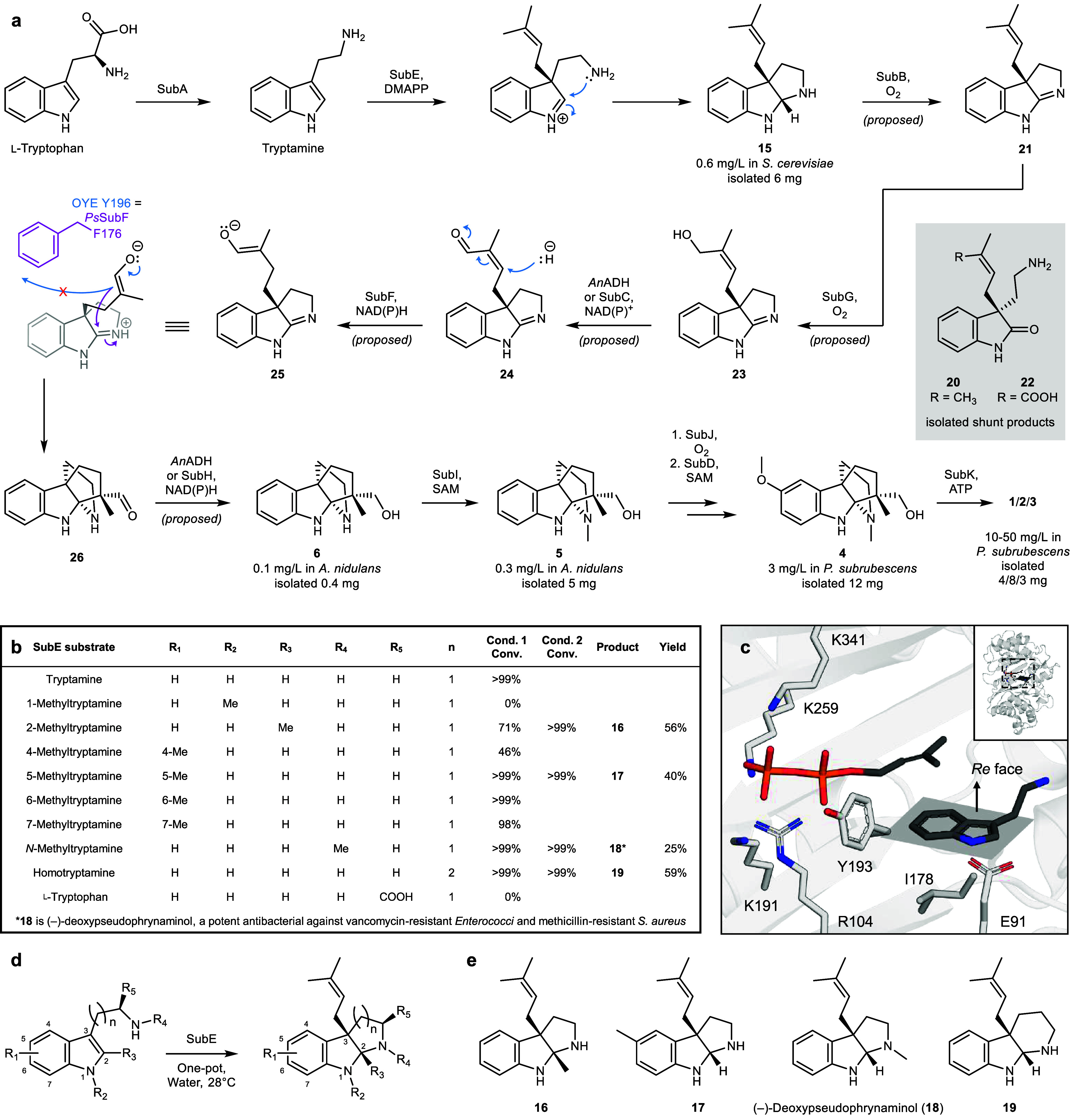
**Subrubine biosynthesis. a,** Proposed formation of the diaza[3.3.3]­propellane core is shown via a cascaded interrupted ene-reduction Mannich cyclization cascade. Isolated compounds were characterized by NMR spectroscopy and high-resolution mass spectrometry. **b,** Conversions and yields of substituted tryptamines when fed to SubE. Cond. 1, μg-scale from DMAPP; Cond. 2, mg-scale from prenol and ATP. **c,** AlphaFold model of SubE showing docked substrate, predicted indole face selectivity, and key active site residues. **d,** SubE reaction enabling one-pot synthesis of pyrrolidinoindolines from tryptamine analogues. **e**, Structures of bioactive and new-to-nature indolines enzymatically synthesized and characterized.

**Figure 4:**
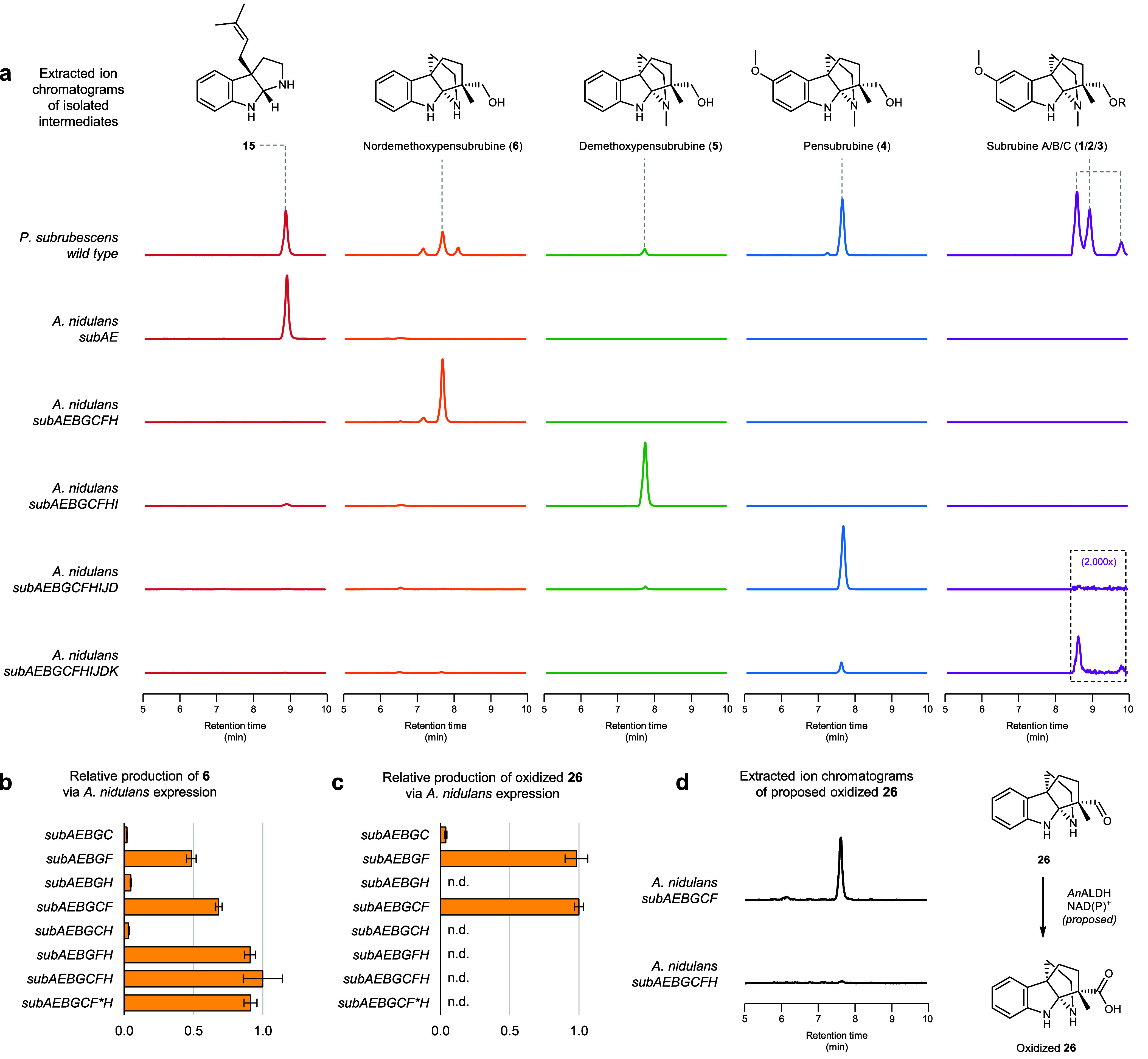
**Complete heterologous pathway reconstitution in *A. nidulans*. a,** Relative abundance of pathway intermediates by LC-MS is shown, normalized to maximum EIC of individual metabolites across strains. **b,** Relative production of the propellane **6** upon combinatorial expression of pathway enzymes was measured. The gene subF* encodes the tyrosine reversion variant SubF F176Y. **c,** Relative production of proposed oxidized **26** upon combinatorial expression of pathway enzymes was measured; n.d., not detected. **d,** Relative abundance of proposed oxidized **26** by LC-MS is shown across strains as well as proposed conversion of the propellane aldehyde to the propellane acid. EIC of **1**/**2**/**3** (merged), 394.2125/416.2544/393.2173 [M + H]^+^; EIC of **4**, 289.1911, [M + H]^+^; EIC of **5**, 259.1805, [M + H]^+^; EIC of **6**, 245.1648, [M + H]^+^; EIC of **15**, 212.1434, [M+H-NH_3_]^+^; EIC of proposed oxidized **26**, 259.1441, [M + H]^+^. Error bars denote standard deviation across *n* = 3 biological replicates.

We assessed the promiscuity of SubE via incubation with DMAPP and tryptamine analogs, including those containing methyl substitutions at six different positions in the indole ring (Figure 3b). All methylated tryptamines were converted by SubE into prenylated products (Figure S18) except *N*1*-*Me-tryptamine. This is expected as the indole nitrogen forms a hydrogen bond with a strictly conserved glutamate in structurally characterized indole prenyltransferases.^54^ Whereas l-tryptophan was not prenylated, as is consistent with *in vivo* results, SubE efficiently converted homotryptamine to a new product. To isolate products for structural determination, 50 mL-scale reactions were performed with 5 mM of 2-methyl-, 5-methyl-, *N-*methyl- and homotryptamine. DMAPP was synthesized *in situ* from prenol and ATP using alcohol kinases (*Ec*ThiM and *Mj*IPK).^55^ From the one-pot reaction, isolated yields ranged from 25 to 59% (15 to 36 mg). NMR characterization showed prenylation and cyclization occurred for all substrates to give *cis*-fused pyrrolidinoindolines **16**, **17** and **18**, and piperidinoindoline **19** (Figures 3e, S74–S97 and Tables S15–S18). Notably, **16** contains C2–C3 vicinal fully substituted carbons, while **18** is a known antibacterial natural product found in the skin of *Pseudophryne coriacea* (red-backed toadlet).^56,57^ **18** exhibits activity against vancomycin-resistant *Enterococci* and methicillin-resistant *Staphylococcus aureus* (MIC of 20–40 μg/mL), with (−)-**18** showing 43-fold greater activity than (+)-**18**.^58^ Optical rotation value of enzymatically synthesized **18** was in agreement with the reported value for (−)-**18**, which has (*2R,3S*) absolute stereochemistry. Additionally, AlphaFold modeling and substrate docking were used to map the prenyl position relative to tryptamine in the SubE active site (Figures 4c, S19).^59–63^ The predicted active site consists of a coplanar DMAPP anchored by positively charged residues (R104, K191, K259, and K341) on the *Re* face of the indole adjacent to the catalytic E91, also consistent with formation of the (*2R,3S*) enantiomer. Finally, our total synthesis of (−)-pensubrubine (**4**) (*vide infra*), supports the proposed (*2R,3S*) configuration of SubE-derived indolines (**15**–**19**).

### Complete Heterologous Reconstitution of Subrubine Biosynthesis

To determine the next steps in diaza[3.3.3]­propellane formation, the P450-encoding genes (*subB*, *subG*, and *subJ*) were individually coexpressed alongside *subAE* in both *S. cerevisiae* and *A. nidulans*. Only coexpression of *subB* in *A. nidulans* led to the depletion of **15** with the concomitant appearance of the oxindole **20** as confirmed by NMR analysis (Figures S20, S98–S102, and Table S19). Accordingly, *A. nidulans* was chosen as the heterologous host for the remaining reconstitution experiments. We propose **20** is a shunt product formed after SubB-catalyzed oxidation of **15** in the absence of downstream enzymes: following hydroxylation of the C2 bridgehead position in **15**, dehydration would yield the amidine intermediate **21** that can hydrolytically ring open to give **20** (Figure S21). Next, coexpression of *subG* with *subAEB* in *A. nidulans* resulted in disappearance of **20** and emergence of the carboxylated shunt product **22** (Figures S22, S103–S108, and Table S20). (*Z*)-configuration of the prenyl olefin in **22** was determined by the observed NOESY correlation between the vinylic and methyl protons. This suggests SubG catalyzes hydroxylation of the *cis* methyl group in the prenyl chain to convert **21** to the alcohol **23** (Figure S23). *A. nidulans* alcohol dehydrogenase (ADH) could then oxidize **23** to enal **24**, which can be further oxidized to a carboxylic acid by endogenous α,β-unsaturated aldehyde detoxifications pathways,^64,65^ followed by subsequent hydrolysis to **22**.

Isolation of shunt products **20** and **22** allowed the proposal of alcohol **23** as an on-pathway intermediate to **6**, with one mechanistic hypothesis shown in Figure 3a. Mirroring the biosynthetic pathway of lysergic acid (Figure S24), enzymatic oxidation of **23** would result in the enal **24** that is the substrate of SubF, which could catalyze 1,4-hydride addition to generate enolate **25**. In contrast to the canonical quenching pathway mediated by the conserved tyrosine, in the interrupted variant, SubF facilitates a Mannich addition of the enolate to the amidine (or amidinium), affording the propellane aldehyde **26**. Enzyme-catalyzed aldehyde reduction would prevent retro-Mannich cleavage, giving alcohol **6** as the product. Coexpression of *subF* with *subAEBG* in *A. nidulans* indeed led to the appearance of a metabolite with a mass corresponding to **6**, along with the disappearance of **22**. Additional coexpression of the coclustered ADHs *subC* and *subH* with *subAEBGF* led to the highest titer of **6** at 0.1 mg/L (Figures 4b and S25). Large-scale fermentation and isolation enabled complete NMR characterization, confirming **6** is nordemethoxypensubrubine (Figures S62–S67, Table S13). Individual functions of SubC and SubH cannot be directly assayed due to lack of available substrate; we propose SubC and SubH catalyze the alcohol oxidation and aldehyde reduction steps that sandwich the SubF reaction, respectively, for the following reasons: (1) SubC shares 30% identity with EasC which catalyzes α,β-unsaturated alcohol oxidation in lysergic acid biosynthesis,^66^ (2) Cofactory 1.0 ^67^ predicted SubC to be NAD^+^ dependent while SubH to be NADPH dependent; and (3) a species with an exact mass consistent with oxidized **26** in the *subAEBGCF* strain disappeared upon coexpression of *subH*, which may accelerate reduction of **26** to **6** (Figures 4c, 4d, S23).

We next expressed the F176Y mutant of SubF in *A. nidulans* to evaluate the role of the active site Phe in propellane formation. Notably, reversion of the Phe back to Tyr resulted in only a small decrease (<10%) in the amount of **6** produced in the heterologous host (Figure 4b), with no apparent formation of ene-reduced shunt products. Therefore, the propellane synthase activity of SubF can be attributed to broader active site residues that reroute the canonical enolate intermediate toward an intramolecular Mannich cyclization. This is consistent with prior work by Cheng et al., in which the corresponding Phe-to-Tyr mutation in EasA retained significant isomerase activity.^5^ Thus, while the presence of Phe in place of Tyr serves as a useful genome-mining signature, it is not strictly required for noncanonical functionality in SubF. To complete the final steps in the biosynthesis of **1**, remaining genes were introduced into the *A. nidulans* strain that produces **6** (Figure 4a). Coexpression of *subI* with *subAEBGCFH* resulted in complete conversion of **6** to the *N*-methylated product demethoxypensubrubine (**5**) at 0.3 mg/L (Figures S27, S56–S61 and Table S12). Coexpression of *subJ* (P450) and *subD* (OMT) in the *subAEBGCFHI* strain led to near complete conversion of **5** to pensubrubine (**4**) at 2.5 mg/L (Figure S28). Finally, the expression of all 11 genes in *A. nidulans* resulted in *de novo* production of **1** (∼0.01 mg/L) and **3** (∼0.01 mg/L) (Figures 4a and S29). Potential explanations for low titers of **1** and **3** in *A. nidulans* include limited endogenous supply of nicotinic acid and benzoic acid; suboptimal activity of SubK due to insufficient T-domain phosphopantetheinylation; or upregulated detoxification pathways due to yet undetermined biological activities of subrubine esters. Formation of **2** was not detected, likely a result of undetectable levels of *N*-formyl-l-valine in *A. nidulans.* Instead, diastereomers of **2** were readily prepared from **4** via chemical esterification with either *N*-formyl-l-valine or *N*-formyl-d-valine, which enabled confirmation that **2** is the *N*-formyl-l-valine ester of **4** (Figure S30).

### Total Synthesis of Pensubrubine Using the Interrupted Fischer Indolization Reaction

The total synthesis of pensubrubine (**4**, Figure 5a) was also pursued. As seen by the 3D depiction, this pyrrolidinoindoline natural product contains a unique diaza[3.3.3]­propellane and features three contiguous fully substituted stereocenters, rendering **4** as a challenging yet attractive synthetic target. Moreover, at the outset of our studies, the stereochemical assignment of **4** was unknown but could plausibly be established unambiguously by total synthesis. Lastly, our approach would rely on an interrupted Fischer indolization reaction, detailed below. Interrupted chemical transformations are strategically valuable^1^ and, indeed, the interrupted Fischer indolization has been used previously to assemble pyrrolidinoindolines and related scaffolds^68–70^ with applications in complex molecule synthesis.^71–76^ However, this methodology has not previously been employed to construct a diaza[3.3.3]­propellane scaffold and, if successful, would push the known limits of interrupted Fischer indolization chemistry. Additionally, the interrupted Fischer indolization approach is mechanistically distinct from the previously discussed biosynthesis, which renders it an effective complementary tactic for accessing the subrubine natural products.

**Figure 5:**
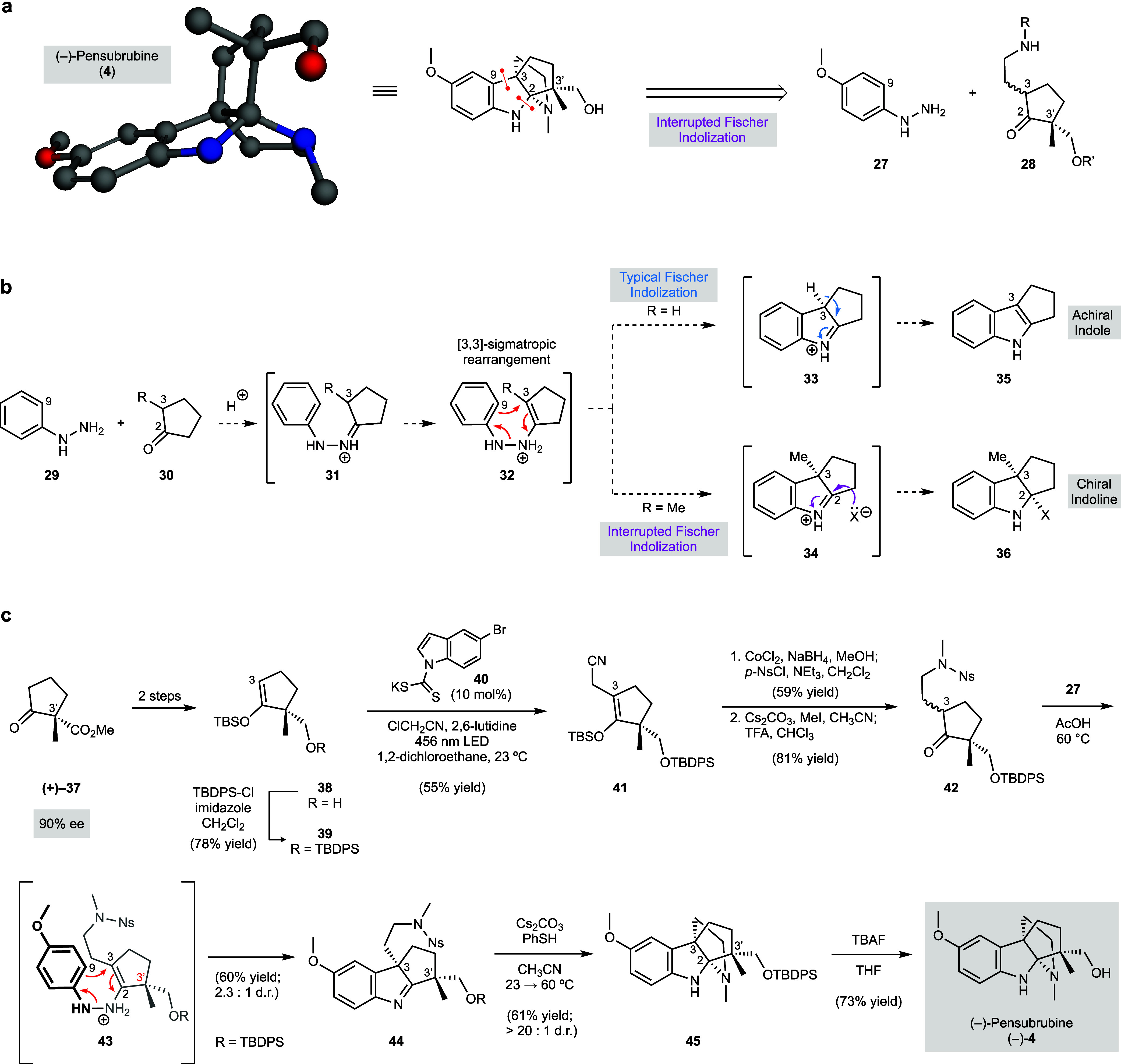
**Total synthesis of (−)-pensubrubine (4) using an interrupted Fischer indolization strategy. a,** Structural features and key retrosynthetic disconnection of (−)-pensubrubine (**4**). **b,** Distinct mechanistic pathways of classic and interrupted Fischer indolizations. **c,** Concise synthesis of (−)-pensubrubine (**4**).

Our retrosynthetic analysis for (−)-pensubrubine (**4**) is depicted in Figure 5a. We envisioned that the diaza[3.3.3]­propellane scaffold could be assembled using a diastereoselective interrupted Fischer indolization reaction of two readily accessible fragments: 4-methoxyphenylhydrazine (**27**) and enantioenriched ketone **28**. We anticipated that the single established stereocenter in ketone **28** (at C3′) could be leveraged to ultimately introduce two newly formed stereocenters, the fully substituted stereocenters at C2 and C3 of **4**, thus establishing the challenging pensubrubine C3–C2–C3′ stereotriad.

Figure 5b highlights key differences between typical Fischer indolizations and the interrupted variant using phenylhydrazine (**29**) and cyclopentanones **30** (R = H or Me) as exemplary substrates. The compounds are condensed, typically under acidic conditions, yielding hydrazonium species **31**. Subsequent tautomerization sets the stage for [3,3]-sigmatropic rearrangement (see transition structure **32**), leading to indolenium **33** or **34**. In a typical Fischer indolization (R = H), deprotonation of **33** at C3 enables rearomatization and gives the achiral indole **35**. However, in the interrupted variant (e.g., R = Me), the standard pathway of deprotonation/rearomatization is unavailable because of the newly formed C3 quaternary stereocenter.^69,77^ Indolenium **34** can instead be captured with a nucleophile, in a direct or stepwise manner, to give the chiral indoline product **36**. This capture can occur intramolecularly, as shown below, to rapidly build the structural complexity.

Following a successful model system study that allowed us to quickly build the diaza[3.3.3]­propellane core of pensubrubine (**4**) using an efficient interrupted Fischer indolization reaction (see Supporting Information, Experimental Procedure 13.2 for details), we commenced the total synthesis. Known compound **38** ^78^ was prepared in two steps from commercially available enantioenriched β-ketoester (+)-**37** (Figure 5c). Next, silyl protection of the alcohol (**38** → **39**), followed by cyanomethylation of silyl enol ether **39** using the photocatalytic conditions developed by Melchiorre and co-workers^79^ delivered nitrile **41**. From **41**, the desired interrupted Fischer indolization substrate, ketone **42**, was readily accessed via a straightforward sequence involving nitrile reduction, *N*-functionalization, and silyl enol ether hydrolysis.

Having constructed enantioenriched cyclopentanone **42**, we turned our attention to the key diastereoselective interrupted Fischer reaction (Figure 5c). Upon heating ketone **42** and 4-methoxyphenylhydrazine (**27**) in degassed acetic acid at 60 °C, we were delighted to observe the desired transformation. Diastereomers **44** were obtained in 61% yield (dr = 2.3:1). The C3 and C3′ stereocenters of the major epimer (as depicted) were assigned based on NOESY analysis of the subsequent intermediate in our synthesis (i.e., **45**) based on the assumption that both of these quaternary stereocenters were not prone to epimerization. We surmise that diastereoselectivity is governed in the [3,3]-sigmatropic rearrangement step (see transition structure **43**), wherein the aryl ring approaches from the more accessible β face to avoid the large C3′ silyloxymethyl substituent. This transformation establishes the challenging quaternary stereocenter at C3 and sets the stage for the completion of the synthesis. Removal of the nosyl group of diastereomers **44** proceeded with cyclization to form the pyrrolidinoindoline fused diaza[3.3.3]­propellane core of the natural product, thus introducing the C3–C2–C3′ stereotriad. The isomeric products were separable and the desired diastereomer **45** was isolated in 61% yield (d.r. > 20:1). Finally, deprotection of **45** using tetrabutylammonium fluoride (TBAF) delivered (−)-pensubrubine (**4**). Characterization data of our synthetic sample matched that of the biosynthetic sample and, importantly, provided unambiguous evidence of the absolute stereochemistry of (−)-**4** (Figure S139–S144 and Table S11).

Our total synthesis of (−)-**4** was achieved in just seven steps from known compound **38**. The concise nature of our approach can be attributed to the strategic use of interrupted Fischer indolization chemistry to construct the core framework in a rapid and diastereoselective fashion. This methodology will enable the applications of interrupted Fischer indolization reactions in total synthesis while also prompting the further development of interrupted reactions more generally to build structural complexity.

## Conclusion

In this study, we demonstrate two distinct interrupted processes in the bio- and total synthesis of subrubine alkaloids – a novel family of fungal pyrrolidinoindolines featuring a congested diaza[3.3.3]­propellane core. The key biosynthetic step in the formation of subrubine alkaloids is an interrupted ene-reduction catalyzed by noncanonical ene-reductase SubF, which reroutes the enolate intermediate from the conventional protonation pathway to undergo a reductive Mannich cyclization. This C–C bond formation strategy underscores the versatility of two-electron reaction manifolds accessible by flavoenzymes in biosynthetic pathways.^28,29^ We have also developed a 7-step enantioselective total synthesis of pensubrubine using a diastereoselective interrupted Fischer reaction. Together, these complementary approaches provide reliable access to subrubines and related analogues, setting the stage for future investigations into the biological function of this unique family of alkaloids. In both cases, the use of interrupted reaction pathways is essential for the assembly of the intricate azapropellane framework.

## Supplementary Material





## Notes

Experimental details and additional data, including HRMS, UV–vis spectra, and NMR spectra for all compounds, as well as all relevant gene and protein sequences (PDF)
Transparent Peer Review report available (PDF)

## §

eXoZymes, Inc., Monrovia, California 91016, United States

## ∥

Science & Innovation Center, Mitsubishi Chemical Corporation, 1000 Kamoshida-cho, Aoba-ku, Yokohama-shi, Kanagawa 227-8502, Japan

## ■

Genentech, South San Francisco, California 94080, United States

## ⊥

Syngenta Group, Münchwilen, 4333 Aargau, Switzerland

## ¶

Hexagon Bio, Menlo Park, California 94025, United States

## Abbreviations

BGC: biosynthetic gene cluster
OYE: old yellow enzyme
NRPS: nonribosomal peptide synthetase
OMT: O-methyltransferase
TDC: tryptophan decarboxylase
SDR: short-chain dehydrogenase/reductase
DMATS: dimethylallyl tryptophan synthase
MDR: medium-chain dehydrogenase/reductase
NMT: *N*-methyltransferase
SS: salicylate synthase
SH: salicylate hydroxylase
PFT: pyrrolidinoindoline forming transferase
MIC: minimum inhibitory concentration
NOESY: nuclear Overhauser effect spectroscopy
ADH: alcohol dehydrogenase
TBAF: tetrabutylammonium fluoride
