# Correction to “Propellane Alkaloid Biosynthesis and Total Synthesis via Interrupted Reaction Pathways”

**DOI:** 10.1021/acscentsci.6c01223

**Published:** 2026-07-29

**Authors:** John M. Billingsley, Jiaming Ding, Allison T. Hands, Kanji Niwa, Nathan J. Adamson, Lukas A. Wein, Bruno Perlatti, Neil K. Garg, Yi Tang

Under Acknowledgments, the grant number CHE-1900178 should read CHE-2246904.

