# Supplementary material for: Propellane Alkaloid Biosynthesis and Total Synthesis via Interrupted Reaction Pathways
Source: ACS Cent Sci. 2026 Apr 3;12(4):532–42. doi: 10.1021/acscentsci.6c00057 (PMC13107220; doi:10.1021/acscentsci.6c00057)
Supplement: Supplementary file 1 [file oc6c00057_si_001.pdf]

SUPPORTING INFORMATION

Propellane alkaloid biosynthesis and total synthesis via interrupted reaction pathways

John M. Billingsley[1,3,8], Jiaming Ding[2,8], Allison T. Hands[2], Kanji Niwa[1,4], Nathan J. Adamson[2,5], Lukas A. Wein[2,6], Bruno Perlatti[2,7], Neil K. Garg[2]*, Yi Tang[1,2]*

[1]Department of Chemical and Biomolecular Engineering, University of California, Los Angeles, CA 90095, USA, [2]Department of Chemistry and Biochemistry, University of California, Los Angeles, CA 90095, USA, [3]Present address: eXoZymes, Inc., Monrovia, CA 91016, USA, [4]Present address: Science & Innovation Center, Mitsubishi Chemical Corporation, 1000 Kamoshida-cho, Aoba-ku, Yokohama-shi, Kanagawa 227-8502, Japan, [5]Present address: Genentech, South San Francisco, CA 94080, USA, [6]Present address: Syngenta Group, Münchwilen, 4333 Aargau, Switzerland, [7]Present address: Hexagon Bio, Menlo Park, CA 94025, USA, [8]These authors contributed equally.

*yitang@ucla.edu, neilgarg@ucla.edu

Table of Contents

Experimental procedures

**1.**	Strains and general culture conditions.	S6
**2.**	General DNA manipulation techniques.	S6
**3.**	Heterologous expression in *Aspergillus nidulans.*	S6
**4.**	Heterologous expression in *Saccharomyces cerevisiae.*	S6
**5.**	Analysis and separation of metabolites.	S6
**6.**	Isolation of compounds.	S7
**7.**	Spectroscopic analyses of isolated compounds.	S8
**8.**	Phylogenetic analysis of OYEs.	S8
**9.**	Deletion of *Aspergillus terreus* OYE using CRISPR-Cas9.	S9
**10.**	Expression and purification of SubE, EcThiM, and MjIPK.	S10
**11.**	*In vitro* reaction of SubE.	S10
**12.**	Structural modelling of SubE.	S10
**13.**	Total synthesis of (–)-pensubrubine (**4**)	S10

Supplementary Tables

**Table S1.**	Aspergillus OYEs identified via NCBI Blast.	S19
**Table S2.**	Bioinformatic analysis of the *sub* gene cluster from *P. subrubescens.*	S23
**Table S3.**	gDNA sequences and predicted protein sequences.	S24
**Table S4.**	Primers and gene fragments used in this study.	S26
**Table S5.**	Plasmids used in this study.	S28
**Table S6.**	Strains constructed in this study.	S29
**Table S7.**	LC-MS chromatograms presented in this study.	S30
**Table S8.**	Spectroscopic data for subrubine A (**1**).	S31
**Table S9.**	Spectroscopic data for subrubine B (**2**).	S32
**Table S10.**	Spectroscopic data for subrubine C (**3**).	S33
**Table S11.**	Spectroscopic data for isolated and synthetic pensubrubine (**4**).	S34
**Table S12.**	Spectroscopic data for demethoxypensubrubine (**5**).	S35
**Table S13.**	Spectroscopic data of nordemethoxypensubrubine (**6**).	S36
**Table S14.**	Spectroscopic data of compound **15**.	S37
**Table S15.**	Spectroscopic data of compound **16**.	S38
**Table S16.**	Spectroscopic data of compound **17**.	S39
**Table S17.**	Spectroscopic data of (–)-deoxypseudophrynaminol (**18**).	S40
**Table S18.**	Spectroscopic data of compound **19**.	S41
**Table S19.**	Spectroscopic data of compound **20**.	S42
**Table S20.**	Spectroscopic data of compound **22**.	S43

Supplementary Figures

**Figure S1.**	The role of EasA in ergot alkaloid lysergic acid biosynthesis.	S44
**Figure S2.**	Phylogenetic tree of Aspergillus OYEs.	S45
**Figure S3.**	*Aspergillus* ψOYEs - Group A.	S46
**Figure S4.**	*Aspergillus* ψOYEs - Group B.	S47
**Figure S5.**	*Aspergillus* ψOYEs - Group C.	S48
**Figure S6.**	Deletion of *sub* cluster from *A. terreus.*	S49
**Figure S7.**	Identification of sub gene cluster product via comparative metabolomics.	S50

**Figure S8.**	Prediction of molecular formula of subrubine A (**1**) via HR-MS.	S51
**Figure S9.**	Incorporation of L-tryptophan-(*indole*-d5) into subrubines.	S52
**Figure S10.**	Production of subrubines by *P. subrubescens* on a variety of fungal growth media.	S53
**Figure S11.**	Retrobiosynthetic analysis of subrubines.	S54
**Figure S12.**	*In vivo* characterization of *subA*.	S55
**Figure S13.**	Proposed formation of indole-3-acetic acid via TAM (tryptamine) pathway.	S56
**Figure S14.**	*In vivo* characterization of *subAE*.	S57
**Figure S15.**	*In vitro* characterization of SubE.	S58
**Figure S16.**	Pyrrolidinoindoline forming prenyltransferases.	S59
**Figure S17.**	Pyrrolidinoindoline forming methyltransferases.	S60
**Figure S18.**	SubE time course on modified tryptamines.	S61
**Figure S19.**	Comparison of DMATS active sites and proposed mechanisms of prenylation.	S62
**Figure S20.**	*In vivo* characterization of *A. nidulans* expressing *subAEB*.	S63
**Figure S21.**	Proposed formation of shunt product **20**.	S64
**Figure S22.**	*In vivo* characterization of *A. nidulans* expressing *subAEBG*.	S65
**Figure S23.**	Proposed formation of shunt product **22**.	S66
**Figure S24.**	Ergot alkaloid formation versus proposed propellane alkaloid formation.	S67
**Figure S25.**	*In vivo* characterization of *A. nidulans* expressing *subAEBGCFH*.	S68
**Figure S26.**	*In vivo* detection of putative propellane acid in the absence of *subH*.	S69
**Figure S27.**	*In vivo* characterization of *A. nidulans* expressing *subAEBGCFHI*.	S70
**Figure S28.**	*In vivo* characterization of *A. nidulans* expressing *subAEBGCFHIJD*.	S71
**Figure S29.**	*In vivo* characterization of *A. nidulans* expressing *subAEBGCFHIJDK*.	S72
**Figure S30.**	Determination of *N*-formyl-valine configuration.	S73
**Figure S31.**	SDS−PAGE analysis of EcThiM, MjIPK, and SubE expressed in *E. coli.*	S74
**Figure S32.**	^{1}H NMR spectrum of subrubine A (**1**) in CD_3OD (500 MHz).	S75
**Figure S33.**	^{13}C NMR spectrum of subrubine A (**1**) in CD_3OD (125 MHz).	S75
**Figure S34.**	^{1}H-^{1}H COSY spectrum of subrubine A (**1**) in CD_3OD (500 MHz).	S76
**Figure S35.**	HSQC spectrum of subrubine A (**1**) in CD_3OD (500 MHz).	S76
**Figure S36.**	HMBC spectrum of subrubine A (**1**) in CD_3OD (500 MHz).	S77
**Figure S37.**	NOESY spectrum of subrubine A (**1**) in CD_3OD (500 MHz).	S77
**Figure S38.**	^{1}H NMR spectrum of subrubine B (**2**) in DMSO-d_6 (500 MHz).	S78
**Figure S39.**	^{13}C NMR spectrum of subrubine B (**2**) in DMSO-d_6 (125 MHz).	S78
**Figure S40.**	^{1}H-^{1}H COSY spectrum of subrubine B (**2**) in DMSO-d_6 (500 MHz).	S79
**Figure S41.**	HSQC spectrum of subrubine B (**2**) in DMSO-d_6 (500 MHz).	S79
**Figure S42.**	HMBC spectrum of subrubine B (**2**) in DMSO-d_6 (500 MHz).	S80
**Figure S43.**	ROESY spectrum of subrubine B (**2**) in DMSO-d_6 (500 MHz).	S80
**Figure S44.**	^{1}H NMR spectrum of subrubine C (**3**) in DMSO-d_6 (500 MHz).	S81
**Figure S45.**	^{13}C NMR spectrum of subrubine C (**3**) in DMSO-d_6 (125 MHz).	S81
**Figure S46.**	^{1}H-^{1}H COSY spectrum of subrubine C (**3**) in DMSO-d_6 (500 MHz).	S82
**Figure S47.**	HSQC spectrum of subrubine C (**3**) in DMSO-d_6 (500 MHz).	S82
**Figure S48.**	HMBC spectrum of subrubine C (**3**) in DMSO-d_6 (500 MHz).	S83
**Figure S49.**	ROESY spectrum of subrubine C (**3**) in DMSO-d_6 (500 MHz).	S83
**Figure S50.**	^{1}H NMR spectrum of pensubrubine (**4**) in CD_3OD (600 MHz).	S84
**Figure S51.**	^{13}C NMR spectrum of pensubrubine (**4**) in CD_3OD (151 MHz).	S84
**Figure S52.**	^{1}H-^{1}H COSY spectrum of pensubrubine (**4**) in CD_3OD (600 MHz).	S85
**Figure S53.**	HSQC spectrum of pensubrubine (**4**) in CD_3OD (600 MHz).	S85

Figure S54. HMBC spectrum of pensubrubine (**4**) in CD_3OD (600 MHz). S86
Figure S55. NOESY spectrum of pensubrubine (**4**) in CD_3OD (600 MHz). S86
Figure S56. ^1H NMR spectrum of demethoxypensubrubine (**5**) in CD_3OD (500 MHz). S87
Figure S57. ^{13}C NMR spectrum of demethoxypensubrubine (**5**) in CD_3OD (125 MHz). S87
Figure S58. ^1H-^1H COSY spectrum of demethoxypensubrubine (**5**) in CD_3OD (500 MHz). S88
Figure S59. HSQC spectrum of demethoxypensubrubine (**5**) in CD_3OD (500 MHz). S88
Figure S60. HMBC spectrum of demethoxypensubrubine (**5**) in CD_3OD (500 MHz). S89
Figure S61. NOESY spectrum of demethoxypensubrubine (**5**) in CD_3OD (500 MHz). S89
Figure S62. ^1H NMR spectrum of nordemethoxypensubrubine (**6**) in CD_3OD (500 MHz). S90
Figure S63. ^{13}C NMR spectrum of nordemethoxypensubrubine (**6**) in CD_3OD (125 MHz). S90
Figure S64. ^1H-^1H COSY spectrum of nordemethoxypensubrubine (**6**) in CD_3OD (500 MHz). S91
Figure S65. HSQC spectrum of nordemethoxypensubrubine (**6**) in CD_3OD (500 MHz). S91
Figure S66. HMBC spectrum of nordemethoxypensubrubine (**6**) in CD_3OD (500 MHz). S92
Figure S67. NOESY spectrum of nordemethoxypensubrubine (**6**) in CD_3OD (500 MHz). S92
Figure S68. ^1H NMR spectrum of **15** in pyridine-d_5 (500 MHz). S93
Figure S69. ^{13}C NMR spectrum of **15** in pyridine-d_5 (125 MHz). S93
Figure S70. ^1H-^1H COSY spectrum of **15** in pyridine-d_5 (500 MHz). S94
Figure S71. HSQC spectrum of **15** in pyridine-d_5 (500 MHz). S94
Figure S72. HMBC spectrum of **15** in pyridine-d_5 (500 MHz). S95
Figure S73. ROESY spectrum of **15** in pyridine-d_5 (500 MHz). S95
Figure S74. ^1H NMR spectrum of **16** in acetonitrile-d_3 (500 MHz). S96
Figure S75. ^{13}C NMR spectrum of **16** in acetonitrile-d_3 (125 MHz). S96
Figure S76. ^1H-^1H COSY spectrum of **16** in acetonitrile-d_3 (500 MHz). S97
Figure S77. HSQC spectrum of **16** in acetonitrile-d_3 (500 MHz). S97
Figure S78. HMBC spectrum of **16** in acetonitrile-d_3 (500 MHz). S98
Figure S79. NOESY spectrum of **16** in acetonitrile-d_3 (500 MHz). S98
Figure S80. ^1H NMR spectrum of **17** in pyridine-d_5 (500 MHz). S99
Figure S81. ^{13}C NMR spectrum of **17** in pyridine-d_5 (125 MHz). S99
Figure S82. ^1H-^1H COSY spectrum of **17** in pyridine-d_5 (500 MHz). S100
Figure S83. HSQC spectrum of **17** in pyridine-d_5 (500 MHz). S100
Figure S84. HMBC spectrum of **17** in pyridine-d_5 (500 MHz). S101
Figure S85. ROESY spectrum of **17** in pyridine-d_5 (500 MHz). S101
Figure S86. ^1H NMR spectrum of (–)-deoxypseudophrynaminol (**18**) in $CDCl_3$ (500 MHz). S102
Figure S87. ^{13}C NMR spectrum of (–)-deoxypseudophrynaminol (**18**) in $CDCl_3$ (500 MHz). S102
Figure S88. ^1H-^1H COSY spectrum of (–)-deoxypseudophrynaminol (**18**) in $CDCl_3$ (500 MHz). S103
Figure S89. HSQC spectrum of (–)-deoxypseudophrynaminol (**18**) in $CDCl_3$ (500 MHz). S103
Figure S90. HMBC spectrum of (–)-deoxypseudophrynaminol (**18**) in $CDCl_3$ (500 MHz). S104
Figure S91. NOESY spectrum of (–)-deoxypseudophrynaminol (**18**) in $CDCl_3$ (500 MHz). S104
Figure S92. ^1H NMR spectrum of **19** in $CDCl_3$ (500 MHz). S105
Figure S93. ^{13}C NMR spectrum of **19** in $CDCl_3$ (500 MHz). S105
Figure S94. ^1H-^1H COSY spectrum of **19** in $CDCl_3$ (500 MHz). S106
Figure S95. HSQC spectrum of **19** in $CDCl_3$ (500 MHz). S106
Figure S96. HMBC spectrum of **19** in $CDCl_3$ (500 MHz). S107
Figure S97. NOESY spectrum of **19** in $CDCl_3$ (500 MHz). S107
Figure S98. ^1H NMR spectrum of **20** in CD_3OD (500 MHz). S108
Figure S99. ^{13}C NMR spectrum of **20** in CD_3OD (125 MHz). S108

Figure S100. ^{1}H-^{1}H COSY spectrum of **20** in CD_3OD (500 MHz). S109
Figure S101. HSQC spectrum of **20** in CD_3OD (500 MHz). S109
Figure S102. HMBC spectrum of **20** in CD_3OD (500 MHz). S110
Figure S103. ^{1}H NMR spectrum of **22** in CD_3OD (500 MHz). S110
Figure S104. ^{13}C NMR spectrum of **22** in CD_3OD (125 MHz). S111
Figure S105. ^{1}H-^{1}H COSY spectrum of **22** in CD_3OD (500 MHz). S111
Figure S106. HSQC spectrum of **22** in CD_3OD (500 MHz). S112
Figure S107. HMBC spectrum of **22** in CD_3OD (500 MHz). S112
Figure S108. NOESY spectrum of **22** in CD_3OD (500 MHz). S113
Figure. S109. ^{1}H NMR spectrum of **47** in $CDCl_3$ (600 MHz). S114
Figure. S110. ^{13}C NMR spectrum of **47** in $CDCl_3$ (151 MHz). S114
Figure. S111. ^{1}H NMR spectrum of **49** in $CDCl_3$ (600 MHz). S115
Figure. S112. ^{13}C NMR spectrum of **49** in $CDCl_3$ (126 MHz). S115
Figure. S113. ^{1}H NMR spectrum of **50** in $CDCl_3$ (600 MHz). S116
Figure. S114. ^{13}C NMR spectrum of **50** in $CDCl_3$ (151 MHz). S116
Figure. S115. HSQC spectrum of **50** in $CDCl_3$ (600 MHz). S117
Figure. S116. HMBC spectrum of **50** in $CDCl_3$ (600 MHz). S117
Figure. S117. ^{1}H-^{1}H COSY spectrum of **50** in $CDCl_3$ (600 MHz). S118
Figure. S118. NOESY spectrum of **50** in $CDCl_3$ (600 MHz). S118
Figure. S119. ^{1}H NMR spectrum of (–)-**39** in $CDCl_3$ (600 MHz). S119
Figure. S120. ^{13}C NMR spectrum of (–)-**39** in $CDCl_3$ (126 MHz). S119
Figure. S121. ^{1}H NMR spectrum of (–)-**41** in $CDCl_3$ (600 MHz). S120
Figure. S122. ^{13}C NMR spectrum of (–)-**41** in $CDCl_3$ (151 MHz). S120
Figure. S123. ^{1}H NMR spectrum of **42** in $CDCl_3$ (500 MHz). S121
Figure. S124. ^{13}C NMR spectrum of **42** in $CDCl_3$ (126 MHz). S121
Figure. S125. ^{1}H NMR spectrum of **44a** and **44b** in $CDCl_3$ (600 MHz). S122
Figure. S126. ^{13}C NMR spectrum of **44a** and **44b** in $CDCl_3$ (151 MHz). S122
Figure. S127. ^{1}H NMR spectrum of (–)-**45** in $CDCl_3$ (600 MHz). S123
Figure. S128. ^{13}C NMR spectrum of (–)-**45** in $CDCl_3$ (151 MHz). S123
Figure. S129. HSQC spectrum of (–)-**45** in $CDCl_3$ (600 MHz). S124
Figure. S130. HMBC spectrum of (–)-**45** in $CDCl_3$ (600 MHz). S124
Figure. S131. ^{1}H-^{1}H COSY spectrum of (–)-**45** in $CDCl_3$ (600 MHz). S125
Figure. S132. NOESY spectrum of (–)-**45** in $CDCl_3$ (600 MHz). S125
Figure. S133. ^{1}H NMR spectrum of (+)-**52** in $CDCl_3$ (600 MHz). S126
Figure. S134. ^{13}C NMR spectrum of (+)-**52** in $CDCl_3$ (151 MHz). S126
Figure. S135. HSQC spectrum of (+)-**52** in $CDCl_3$ (600 MHz). S127
Figure. S136. HMBC spectrum of (+)-**52** in $CDCl_3$ (600 MHz). S127
Figure. S137. ^{1}H-^{1}H COSY spectrum of (+)-**52** in $CDCl_3$ (600 MHz). S128
Figure. S138. NOESY spectrum of (+)-**52** in $CDCl_3$ (600 MHz). S128
Figure. S139. ^{1}H NMR spectrum of synthetic (–)-pensubrubine (**4**) in CD_3OD (600 MHz). S129
Figure. S140. ^{13}C NMR spectrum of synthetic (–)-pensubrubine (**4**) in CD_3OD (151 MHz). S129
Figure. S141. HSQC spectrum of synthetic (–)-pensubrubine (**4**) in CD_3OD (600 MHz). S130
Figure. S142. HMBC spectrum of synthetic (–)-pensubrubine (**4**) in CD_3OD (600 MHz). S130
Figure. S143. ^{1}H-^{1}H COSY spectrum of synthetic (–)-pensubrubine (**4**) in CD_3OD (600 MHz). S131
Figure. S144. NOESY spectrum of synthetic (–)-pensubrubine (**4**) in CD_3OD (600 MHz). S131
References S132

Experimental procedures

1. Strains and general culture conditions

Aspergillus terreus NIH2624 (FJB001, obtained from the Fungal Genetics Stock Center) and *Penicillium subrubescens* CBS 132785 (FJB004, obtained from Westerdijk Fungal Biodiversity Institute) were maintained on potato dextrose agar at 28 °C for proliferation and isolation of genomic DNA. *Aspergillus nidulans* A1145ΔEMΔST was grown at 37 °C in CD agar for proliferation and heterologous expression. *Saccharomyces cerevisiae* JHY651 (obtained from Stanford Genome Technology Center) was cultured in YPD 28 °C for proliferation and heterologous expression. All *Escherichia coli* strains were cultured in LB media at 37 °C.

2. General DNA manipulation techniques

All DNA manipulations in this study were conducted according to the manufacturer's protocol. *E. coli* TOP10 was used for cloning, following standard recombinant DNA techniques. PCR was performed using Q5 High-Fidelity DNA Polymerase (NEB). *E. coli* expression and pFC plasmids were constructed via digestion-ligation with NheI/NotI or PacI/BglII and T7 DNA Ligase (NEB). *S. cerevisiae* and *A. nidulans* vectors were constructed via yeast homologous recombination (strain JHY651) and selection using an auxotrophic uracil marker. All resulting plasmids were confirmed by DNA sequencing. *A. terreus* and *P. subrubescens* gDNA was isolated using the Quick-DNA Fungal/Bacterial Microprep Kit (Zymo).

3. Heterologous expression in *A. nidulans*

Standard methods for *A. nidulans* transformation were used, with several modifications. Germinated spores were generated through stationary culture of YG media (5 g/L yeast extract, 20 g/L glucose, and 1x trace elements) at 37 °C for 12 h instead of shaking in liquid CD. Mycelia were collected and incubated for 4 hours with twice the standard amount of Lysing enzymes from Trichoderma and Yatalase. The resulting protoplasts were harvested via filtration as opposed to centrifugation with Trapping buffer. Finally, 3-5 µg of each plasmid was added to each 100 µL aliquot of protoplasts instead of the typical 0.3-0.5 µg. Following *A. nidulans* transformation, correct transformants were selected for on CD sorbitol agar. Colonies were repatched onto CD agar, grown at 28 °C for 7 days, and then subject to metabolite analysis.

4. Heterologous expression in *S. cerevisiae*

Frozen-EZ Yeast Transformation II Kit (Zymo) was used for transformation. The resulting lawn was pooled and transferred to 2 mL liquid uracil dropout media, and transferred to a shaker at 250 rpm and 28 °C for 24 h. The resulting densely grown seed culture (100 µL) was then used to inoculate YPD (2 mL) which was transferred to a shaker at 250 rpm and 28 °C for 72 h.

5. Analysis and separation of metabolites

Fungal solid cultures were sampled by extracting a solid agar plug (0.2 cm^3) with acetone (1 mL). Culture extracts were vortexed for 5 minutes, and then centrifuged at 17,000 g for 10 minutes. The supernatant (200 µL) was dried down by Speedvac and then re-suspended in MeOH (50 µL) by vortexing

for 5 minutes, and centrifuging at 17,000 g for 10 minutes. The resulting supernatant was then subjected to HPLC-MS analysis. Liquid yeast cultures (100 μL) were directly extracted into MeOH (900 μL), vortexed for 5 minutes, and then centrifuged at 17,000 g for 10 minutes. Extracts were subjected to HPLC-MS analysis on an Agilent 1260 Infinity II LC equipped with an InfinityLab Poroshell 120 EC-C18 column (2.7 μm, 3.0 × 50 mm) and a 6545 Q-TOF high resolution mass spectrometer (UCLA Molecular Instrumentation Center). After a 2 min hold at the initial column conditions, the sample was eluted with a linear gradient of 1–95% MeCN containing 0.1% formic acid over 14 min. The LC-MS data was acquired with MassHunter Workstation 10.0 (Agilent) and analyzed with MassHunter Qualitative Analysis 10.0 (Agilent). Medium pressure liquid chromatography (MPLC) was carried out on RediSep Gold® C18 (Teledyne Isco Inc., Lincoln, USA). HPLC was performed on COSMOSIL PBr (5 μm, ϕ10 x 250 mm, Nacalai Tesque, Inc., Japan), COSMOSIL AR-II (5 μm, ϕ20 x 250 mm), and Luna C8 (2) 100 Å (5 μm, ϕ10 × 250 mm, Phenomenex Inc., Torrance, USA).

6. Isolation of compounds

6.1 Isolation of subrubines A−C (1−3) and pensubrubine (4).

P. subrubescens was grown on 10 kg of steam sterilized *Helianthus tuberosus* tubers for 5 days at 28 °C and then extracted with methanol and acetone. The extract was partitioned between EtOAc and H_2O. The EtOAc soluble materials were separated by ODS column (MeCN/H_2O/formic acid (FA), 10:90:0.1−100:0:0.1) to furnish three fractions (frs. 1−3). Fr.1 was separated by ODS HPLC {Luna C8 (2) 100 Å C8, MeCN/H_2O/formic acid, 17:83:0.1} to afford subrubine B (**2**, 8 mg) and C (**3**, 3 mg). Pensubrubine (**4**, 12 mg) was isolated from fr.2 by ODS HPLC (COSMOSIL 5C_{18}-AR-II, MeCN/H_2O/FA, 10:90:0.1−50:50:0.1, 30 min). Fr.3 was purified by ODS HPLC (COSMOSIL 5C_{18}-MS-II, MeCN/H_2O/formic acid, 45:55:0.1) to obtain subrubine A (**1**, 4 mg).

6.2 Isolation of demethoxypensubrubine (5).

A. nidulans strain expressing *subA, subE, subB, subG, subC, subF, subH,* and *subI* (FJB020) was grown on 4 L of CD agar + 20 mg/L tryptophan for 3 days at 28 °C and then extracted with acetone. The extract was partitioned between EtOAc and H_2O. The EtOAc soluble materials were separated by ODS column (MeCN/H_2O/FA, 10:90:0.1−100:0:0.1), and further purified by ODS HPLC (COSMOSIL 5C_{18}-AR-II, MeCN/H_2O/formic acid, 10:90:0.1−50:50:0.1, 30 min) to furnish demethoxypensubrubine (**5**, 5 mg).

6.3 Isolation of nordemethoxypensubrubine (6).

A. nidulans strain expressing *subA, subE, subB, subG, subC, subF,* and *subH* (FJB060) was grown on 4 L of CD agar + 20 mg/L tryptophan for 3 days at 28 °C and then extracted with acetone. The extract was partitioned between EtOAc and H_2O. The EtOAc soluble materials were separated by ODS column (MeCN/H_2O/FA, 10:90:0.1−100:0:0.1), and further purified by ODS HPLC (COSMOSIL 5C_{18}-AR-II, MeCN/H_2O/FA, 10:90:0.1−50:50:0.1, 30 min) to furnish nordemethoxypensubrubine (**6**, 0.4 mg).

6.4 Isolation of 15.

S. cerevisiae transformant expressing *subA* and *subE* (YJB092) was grown in 50 mL liquid uracil droupout media for 1 day at 28 °C and then transferred to 2 L of YPD + 20 mg/L tryptophan for 3 days at 28 °C. Cells and supernatant were separated via centrifugation at 1,000 g for 10 minutes. The supernatant was directly loaded and separated by ODS column (MeCN/H_2O/FA, 10:90:0.1−100:0:0.1), and further purified by ODS HPLC {Luna C8 (2) 100 Å C8, MeCN/H_2O/FA, 20:80:0.1} to furnish **15** (6 mg).

6.5 Isolation of 16.

To obtain **16**, a large scale 50 mL *in vitro* reaction was extracted with methanol (50 mL x 3). The extract was purified by ODS column (MeCN/H_2O/FA, 0:100:0.1−50:50:0.1) to furnish **16** (34 mg).

6.6 Isolation of 17.

To obtain **17**, a large scale 50 mL *in vitro* was extracted with methanol (50 mL x 3). The extract was purified by ODS column (MeCN/H_2O/FA, 0:100:0.1−50:50:0.1) to furnish **17** (24 mg).

6.7 Isolation of (–)-deoxypseudophrynaminol (18).

To obtain **18**, a large scale 50 mL *in vitro* reaction was extracted with methanol (50 mL x 3). The extract was purified by ODS column (MeCN/H_2O/FA, 0:100:0.1−50:50:0.1) to furnish **18** (15 mg).

6.8 Isolation of 19.

To obtain **19**, a large scale 50 mL *in vitro* reaction was extracted with methanol (50 mL x 3). The extract was purified by ODS column (MeCN/H_2O/FA, 0:100:0.1−50:50:0.1) to furnish **19** (36 mg).

6.9 Isolation of 20.

A. nidulans strain expressing *subA, subE,* and *subB* (FJB065) was grown on 4 L of CD agar + 20 mg/L tryptophan for 3 days at 28 °C and then extracted with methanol and acetone. The extract was partitioned between EtOAc and H_2O. The EtOAc soluble materials were separated by ODS column (MeCN/H_2O/FA, 10:90:0.1−100:0:0.1), and further purified by ODS HPLC (COSMOSIL 5C_{18}-AR-II, MeCN/H_2O/FA, 22:78:0.1) to furnish **20** (0.4 mg).

6.10 Isolation of 22.

A. nidulans strain expressing *subA, subE, subB,* and *subG* (FJB054) was grown on 4 L of CD agar + 20 mg/L tryptophan for 3 days at 28 °C and then extracted with methanol and acetone. The extract was separated by ODS column (MeCN/H_2O/FA, 0:100:0.1−50:50:0.1), and further purified by ODS HPLC (COSMOSIL PBr, MeCN/H_2O/FA, 15:85:0.1) to furnish **22** (3 mg).

7. Spectroscopic analyses of isolated compounds

NMR spectra of isolated biosynthesized compounds were measured by a Bruker Avance 500 spectrometer with a 5 mm dual cryoprobe at the UCLA Molecular Instrumentation Center (^{1}H NMR 500 MHz, ^{13}C NMR 125 MHz). The resonances of residual DMSO (δ_H 2.49 and δ_C 39.7), pyridine (δ_H 8.71 and δ_C 123.5), methanol (δ_H 3.30 and δ_C 49.0), acetonitrile (δ_H 1.93 and δ_C 0.99), and $CHCl_3$ (δ_H 7.24 and δ_C 77.0) were used as internal references for the ^{1}H and ^{13}C NMR spectra. High resolution mass spectra were obtained from an Agilent Quadrupole Time of Flight LC/MS (6545 LC/Q-TOF) at the UCLA Molecular Instrumentation Center. UV spectra were recorded on Agilent 1260 Infinity II Diode Array Detector WR or Shimadzu SPD-M20A Photodiode Array Detector. Optical rotations were measured on a Rudolph Research Analytical Autopol III Automatic Polarimeter.

8. Phylogenetic analysis of OYEs

The OYE2 protein sequence was obtained from the Saccharomyces Genome Database. NCBI BLAST (blastp) was used to retrieve non-redundant homologues in the genus *Aspergillus* (taxid:5052) with an expected threshold of 0.05 and maximum target sequence value of 1000. The sequences were

downloaded and aligned using Clustal Omega (1.2.4) multiple sequence alignment. The output phylogenetic tree was then visualized using iTOL default parameters, which matched the phylogenetic classifications performed by Nizam *et al.* and Robescu *et al.* The aligned sequences were manually parsed, and the 40 OYE homologues with active site residues other than tyrosine were compiled and annotated on the phylogenetic tree. Corresponding genomic contigs ±20 kb were subsequently retrieved from NCBI, and submitted to 2ndFind for analysis of the surrounding genes.

9. Deletion of *A. terreus* OYE using CRISPR-Cas9

A. terreus protoplasts were generated using the same method that was used for *A. nidulans* protoplasts. Initially, OYE deletion was attempted using a linear hygromycin marker flanked by homology arms ≥ 2 kb. High transformation efficiency using the soft agar method was evidenced by hygromycin resistant isolates, however we repeatedly observed an intact XP_001212002.1 locus. Initially, we attributed this to the Ku non-homologous end joining (NHEJ) protein facilitating random incorporation of our selection fragment, which was still present in the wild-type strain. After multiple failed attempts to delete *A. terreus* OYE using the split-marker method, we turned to the pFC CRISPR-Cas9 system to improve knockout efficiency. An expression cassette encoding a constitutively expressed gRNA targeting the XP_001212002 encoding gene was ordered from IDT and cloned into pFC330 (gBlock sequence below, protospacer in the gRNA is underlined). Only after introduction of the circular gene-targeting donor DNA as repair template (previously shown to mediate replacement by eliminating NHEJ) were we successful in deleting XP_001212002. *A. terreus* protoplasts (60 μL) were incubated with 1 mg Cas9 plasmid and 1 mg HR donor plasmid on ice. After 1 hour, 600 μL PEG solution (60% PEG 4000, 50 mM $CaCl_2$, and 50 mM Tris-HCI, pH 7.5) was added, and the protoplasts were plated on 60 mL PDA (2% agar) + 1.2 M sorbitol. After 12 hours, a second layer consisting of 20 mL PDA (1% agar) + 1.2 M sorbitol with 800 mg/L hygromycin was added. Dilution streaking was performed to obtain single isolates, and the knockout was confirmed by genomic DNA purification, PCR, and sequencing.

>XP_001212002_knockout_gRNA_expression_cassette (gBlock)
GCGTAAGCTCCCTAATTGGCCCATCCGGCATCTGTAGGGCGTCCAAATATCGTGCCTCTCCT
GCTTTGCCCGGTGTATGAAACCGGAAAGGCCGCTCAGGAGCTGGCCAGCGGCGCAGACCG
GGAACACAAGCTGGCAGTCGACCCATCCGGTGCTCTGCACTCGACCTGCTGAGGTCCCTCA
GTCCCTGGTAGGCAGCTTTGCCCCGTCTGTCCGCCCGGTGTGTCGGCGGGGTTGACAAGGT
CGTTGCGTCAGTCCAACATTTGTTGCCATATTTTCCTGCTCTCCCCACCAGCTGCTCTTTTCT
TTTCTCTTTCTTTTCCCATCTTCAGTATATTCATCTTCCCATCCAAGAACCTTTATTTCCCCTA
AGTAAGTACTTTGCTACATCCATACTCCATCCTTCCCATCCCTTATTCCTTTGAACCTTTCAG
TTCGAGCTTTCCCACTTCATCGCAGCTTGACTAACAGCTACCCCGCTTGAGCAGACATCACC
GATATAGCTGATGAGTCCGTGAGGACGAAACGAGTAAGCTCGTCCTATATTGAGGACTATG
CGCGTTTTAGAGCTAGAAATAGCAAGTTAAAATAAGGCTAGTCCGTTATCAACTTGAAAAA
GTGGCACCGAGTCGGTGCTTTTGGCCGGCATGGTCCCAGCCTCCTCGCTGGCGCCGGCTGG
GCAACATGCTTCGGCATGGCGAATGGGACTGATTTAATAGCTCCATGTCAACAAGAATAAA
ACGCGTTTCGGGTTTACCTCTTCCAGATACAGCTCATCTGCAATGCATTAATGCATTGGACC
TCGCAACCCTAGTACGCCCTTCAGGCTCCGGCGAAGCAGAAGAATAGCTTAGCAGAGTCTA
TTTTCATTTTCGGGAGACGAGATCAAGCAGATCAACGGTCGTCAAGAGACCTACGAGACTG
AGGAATCCGCTCTTGGCTC

10. Expression and purification of SubE, EcThiM, and MjIPK

EcThiM and MjIPK were expressed as described previously. SubE was purified using standard methods. Following cloning into the pET28a vector, plasmid was transformed into *E. coli* BL21(DE3) (Invitrogen) and selected on LB agar + Kanamycin (50 mg/L). A single colony was picked into 4 mL LB + Kanamycin (50 mg/L) and grown in a shaker at 250 rpm and 37 °C for 12 h. The overnight culture was transferred to 1 L LB + Kanamycin (50 mg/L) and returned to the shaker at 250 rpm and 37 °C until an OD_{600} of approximately 0.6 was reached. The shaker temperature was lowered to 16 °C, and 0.4 mM IPTG was added to cultures. After 16 hours, the cells were harvested via centrifugation. Cell pellet was resuspended in Wash Buffer (25 mM Tris-HCl, 200 mM NaCl, 10 mM imidazole, pH = 8) and lysed via sonication. The lysate was clarified via centrifugation (17,000 g for 30 min at 4 °C) and then incubated with Ni-NTA resin (Qiagen) for 3 hours. The resin was washed with 3 column volumes of Wash Buffer and then eluted with 1 column volume Elution Buffer (25 mM Tris-HCl, 200 mM NaCl, 250 mM imidazole, 10 vol% glycerol, pH = 8). Protein concentration was determined using Bradford Reagent, and purity was analyzed via SDS-PAGE (Fig. S31). The protein was aliquoted and stored at -80 °C.

11. *In vitro* reaction of SubE

Assays for SubE activity were carried out in 100 mM Tris-HCl buffer (pH 7.5) containing 1 mM substrate (tryptamine or substituted tryptamine), 10 mM $MgCl_2$, 5 mM DMAPP, and 3 μM SubE with a reaction volume of 100 μL. The reaction also contained 1% DMSO from the substrate stock solution. Stationary reactions were incubated at 28 °C and monitored for 48 hours. Reactions were quenched via 100x dilution into MeOH, briefly vortexed, centrifuged, and subject to HPLC analysis.

Modified conditions for large scale SubE reactions using *in situ* DMAPP production from ATP and prenol were employed. The reactions were carried out in 100 mM Tris-HCl buffer (pH 7.5) containing 5 mM substrate (tryptamine or substituted tryptamine), 30 mM $MgCl_2$, 10 mM prenol, 25 mM ATP, 5 μM SubE, 125 μM EcThiM, and 50 μM MjIPK with a reaction volume of 50 mL. The reaction also contained 5% DMSO from the substrate stock solution. Stationary reactions were incubated at 28 °C and purified after 72 hours.

12. Structural modelling of SubE

The SubE structure was obtained from the AlphaFold Protein Structure Database. AlphaFill was used for ligand placement using the top scoring homolog NotF. The prenyl donor analogue dimethylallyl S-thiolodiphosphate (DMASPP) was transplanted into the active site. To mimic DMAPP, the sulfur atom in DMASPP was replaced with oxygen. For ligand docking, the AlphaFill-derived SubE model was submitted to SwissDock to evaluate binding of tryptamine. Docking was performed using the Attracting Cavities 2.0 method and medium sampling exhaustivity. The highest-scoring pose according to the SwissDock scoring function was selected for subsequent analysis.

13. Total synthesis of (–)-pensubrubine (4)

13.1. Materials and Methods

Unless stated otherwise, reactions were conducted in flame-dried glassware under an atmosphere of nitrogen, and commercially obtained reagents were used as received unless otherwise specified. Anhydrous solvents were either freshly distilled or passed through activated alumina columns.

Tetrahydrofuran (THF), dichloromethane (CH_2Cl_2), diethyl ether (Et_2O), triethylamine (NEt_3), acetonitrile (CH_3CN), and methanol (MeOH) were passed through an activated alumina column prior to use, unless otherwise stated. Glacial acetic acid (AcOH) was purchased from Fisher Scientific and degassed via Freeze-Pump-Thaw technique prior to use. Non-commercially available reagents and catalysts are synthesized according to known preparations, or following protocol specified in the experimental procedures. *tert*-Butyldiphenylsilyl chloride (TBDPSCl) and sodium borohydride ($NaBH_4$) were purchased from Oakwood chemicals. Cobalt dichloride hexahydrate ($CoCl_2 \bullet 6\ H_2O$) was purchased from J. T. Baker. Dimethylhydrazine, Ketone **46**, *n*-butyllithium (*n*-BuLi), (+)-B-chlorodiisopinocampheyl-borane ((+)-DIP-Cl™), chloroacetonitrile, 2,6-lutidine, cesium carbonate (Cs_2CO_3), thiophenol (PhSH) and tetrabutylammonium fluoride (TBAF) were purchased from Sigma Aldrich. Diisopropylamine and iodomethane (MeI) was purchased from Thermo Fisher Scientific. *N*-Tosylaziridine was purchased from BLDpharm.

Reaction temperatures at or above 23 °C were controlled using an IKAmag temperature modulator. Oil baths were used to heat reactions. Thin layer chromatography (TLC) was conducted with EMD gel 60 F254 pre-coated plates (0.25 mm for analytical chromatography and 0.50 mm for preparative thin layer chromatography) and visualized using a combination of UV, anisaldehyde, potassium permanganate, iodine, phosphomolybdic acid, and cerium ammonium molybdate staining techniques. Silicycle Siliaflash P60 (particle size 40–63 μm) was used for flash column chromatography and RediSep Gold Silica (20-40 μm) was used for automated chromatography on a CombiFlash NextGen 300+ instrument unless otherwise specified. ^{1}H-NMR and 2D-NOESY spectra of all synthetic compounds were recorded on Bruker spectrometers (at 500 and 600 MHz) and are reported relative to the residual solvent signal. Data for ^{1}H-NMR spectra are reported as follows: chemical shift (δ ppm), multiplicity, coupling constant (Hz), and integration. ^{13}C-NMR spectra were recorded on Bruker spectrometers (at 125 and 150 MHz) and are reported relative to the residual solvent signal. Data for ^{13}C-NMR spectra are reported in terms of chemical shift (δ ppm). IR spectra were recorded on a Perkin-Elmer UATR Two FT-IR spectrometer and are reported in terms of frequency absorption (cm^{-1}). DART-MS spectra were collected on a Thermo Exactive Plus MSD (Thermo Scientific) equipped with an ID-CUBE ion source and a Vapur Interface (IonSense Inc.). Both the source and MSD were controlled by Excalibur software version 3.0. The analyte was spotted onto OpenSpot sampling cards (IonSense Inc.) using diethyl ether or CH_2Cl_2 as the solvent. Ionization was accomplished using UHP He plasma with no additional ionization agents. The mass calibration was carried out using Pierce LTQ Velos ESI (+) and (–) Ion calibration solutions (Thermo Fisher Scientific). Optical rotations were measured with a Rudolf Autopol III Automatic Polarimeter.

Photocatalyst **40**,[1] 4-methoxyhydrazine free base **27**[2] and alcohol **38**[3] were prepared according to literature procedures. 4-Methoxyhydrazine free base **27** is air-sensitive and was therefore stored in the glovebox prior to use.

13.2. Model System Studies

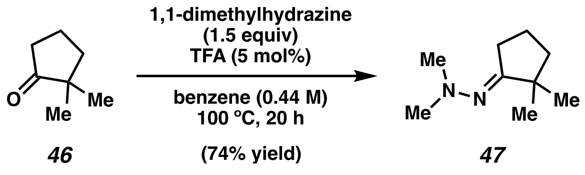

Hydrazone 47. To a solution of ketone **46** (2.2 mL, 17.8 mmol, 1.0 equiv) in benzene (36 mL) in a 100 mL round bottom flask equipped with a stir bar was added 1,1-dimethylhydrazine (2.0 mL, 26.7 mmol, 1.5 equiv) in one portion then trifluoroacetic acid (0.69 mL, 0.89 mmol, 5 mol%) in one portion. The flask was then equipped with a Dean–Stark apparatus equipped with a reflux condenser. The reaction mixture was heated to 100 ºC and allowed to stir at this temperature under ambient atmosphere for 20 h. Then, the reaction mixture was concentrated under reduced pressure and the crude residue was diluted with Et_2O (15 mL) and saturated aq. $NaHCO_3$ (15 mL). The layers were separated, and the aqueous layer was extracted with Et_2O (3 x 15 mL). The organic extracts were combined, dried over Na_2SO_4, filtered, and concentrated under reduced pressure. The crude material was purified by flash chromatography (SiO_2, 25% pentane:Et_2O) to afford hydrazone **47** (1.84 g, 13.2 mmol, 74% yield) as a yellow oil. **Hydrazone 47:** R_f=0.38 (2.3:1 hexanes:EtOAc); ^{1}H NMR (600 MHz, $CDCl_3$): δ 2.45 (d, J = 7.4 Hz, 1H), 2.43 (m, 7H, overlapping signals), 1.71 (p, J = 7.2 Hz, 2H), 1.56 (t, J = 6.9 Hz, 2H), 1.08 (s, 6H); ^{13}C NMR (150 MHz, $CDCl_3$): δ 180.1, 47.0, 42.1, 40.4, 29.1, 26.8, 21.1; IR (film): 2954, 2857, 2772, 1654, 1466 cm^{-1}; HRMS-APCI (*m/z*) [M+H]$^+$ calculated for $C_9H_{19}N_2^+$, 155.1543; found 155.1532.

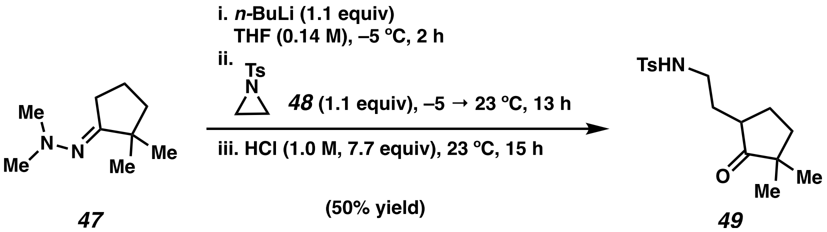

47 **49**

Ketone 49. To a 100 mL round bottom flask equipped with a stir bar containing a solution of hydrazone **47** (100 mg, 0.65 mmol, 1.0 equiv) in THF (3.3 mL) cooled in a salt water/ice bath at –5 ºC under N_2 was added *n*-BuLi (2.48 M in hexanes, 0.30 mL, 0.71 mmol, 1.1 equiv) dropwise via syringe over 5 min. The resulting solution was allowed to stir for 2 h at –5 ºC. Then, a solution of *N*-tosylaziridine **48** (141.0 mg, 0.71 mmol, 1.1 equiv) in THF (1.3 mL) was added via cannula dropwise over 5 min. This solution was allowed to stir at –5 ºC for 1 h and then was allowed to warm to 23 ºC over 13 h. After this time, aqueous HCl (1.0 M, 5.0 mL, 5.0 mmol, 7.7 equiv) was added in one portion and the resulting mixture was allowed to stir for 15 h at 23 ºC. After this time, the reaction was quenched with saturated aq. $NaHCO_3$ (30 mL) portion wise over 1 min. The biphasic mixture was then poured into a separatory funnel and the layers were separated. The aqueous layer was extracted with EtOAc (3 x 10 mL) the combined organic fractions dried over $MgSO_4$, filtered, and concentrated under reduced pressure. The crude material was purified by flash chromatography (SiO_2, 30→50% hexanes:EtOAc) to provide ketone **49** (101.2 mg, 0.33 mmol, 50% yield) as a viscous colorless oil. **Ketone 49:** R_f= 0.62 (1:1 hexanes:EtOAc); ^{1}H NMR (600 MHz, $CDCl_3$): δ 7.74 (d, J = 8.3 Hz, 2H), 7.30 (d, J = 8.0 Hz, 2H), 4.91 (t, J = 6.3 Hz, 1H), 3.09 (sx, J = 6.6 Hz, 1H), 3.03–2.96 (m, 1H), 2.42 (s, 3H), 2.24–2.15 (m, 1H), 2.12–2.05 (m, 1H), 1.83–1.76 (m, 2H), 1.65 (td, J = 12.4, 7.0 Hz, 1H, overlaps with HDO at 1.60), 1.58–1.52 (m, 1H, overlaps with broad singlet at 1.60), 1.52–1.46 (m, 1H), 1.06 (s, 3H), 0.95 (s, 3H); ^{13}C NMR (150 MHz, $CDCl_3$): δ 224.8, 143.4, 137.0, 129.7, 127.1, 46.5, 45.2, 41.8, 36.4, 30.3, 26.0, 24.7, 24.0, 21.5; IR (film): 3280, 1731, 1328, 1159, 1093 cm^{-1}; HRMS-APCI (*m/z*) [M+H]$^+$ calculated for $C_{16}H_{24}NO_3S^+$, 310.1471; found 310.1458.

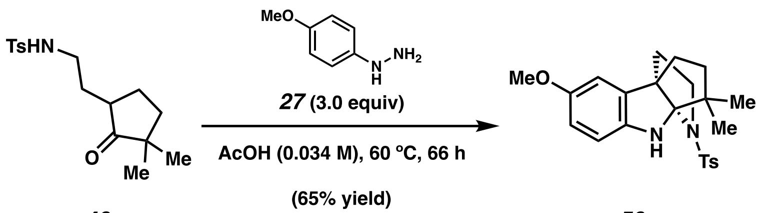

49 *50*

Indoline 50. To a 20 mL scintillation vial equipped with a stir bar was added ketone **49** (20.3 mg, 0.067 mmol, 1.0 equiv) and 4-methoxyphenylhydrazine **27** (27.4 mg, 0.20 mmol, 3.0 equiv). To this mixture was then added AcOH (2 mL) in one portion. The resulting solution was then sparged with N_2 for 10 min. The vial was then sealed with a Teflon-lined cap and stirred at 60 °C under N_2 atmosphere for 66 h. After this time, the reaction mixture was allowed to cool to 23 °C and transferred to a separatory funnel containing saturated aq. $NaHCO_3$ (10 mL) and EtOAc (10 mL). The organic layer was collected, and the aqueous layer was extracted with EtOAc (2 x 10 mL). The organic extracts were combined, dried over $MgSO_4$, filtered, and concentrated under reduced pressure. The crude material was purified by flash chromatography (SiO_2, 10→17% hexanes:Et_2O) to afford indoline **50** (17.7 mg, 0.043 mmol, 65% yield) as a pale-yellow foam. **Indoline 50:** R_f = 0.30 (5:1 hexanes:EtOAc); ^{1}H NMR (600 MHz, $CDCl_3$): δ 7.74–7.68 (d, J = 8.5 Hz, 2H), 7.22–7.18 (d, J = 8.0 Hz, 2H), 6.58 (m, 2H, overlapping signals), 6.39–6.35 (d, J = 8.7 Hz, 1H), 4.90 (brs, 1H), 3.72 (s, 3H), 3.53 (ddd, J = 9.5, 7.4, 3.9 Hz, 1H), 3.10 (td, J = 9.2, 7.1 Hz, 1H), 2.36 (s, 3H), 2.17 (ddd, J = 12.5, 8.9, 7.4 Hz, 1H), 2.08 (ddd, J = 12.4, 7.1, 3.9 Hz, 1H), 1.99 (td, J = 12.3, 6.8 Hz, 1H), 1.88–1.83 (m, 1H), 1.83–1.77 (m, 1H), 1.65 (ddd, J = 12.7, 6.8, 1.6 Hz, 1H), 1.30 (s, 3H), 1.23 (s, 3H); ^{13}C NMR (150 MHz, $CDCl_3$) δ 153.5, 143.4, 143.0, 136.8, 135.4, 129.5, 127.9, 113.2, 109.9, 107.9, 103.6, 69.1, 56.1, 51.7, 46.9, 44.1, 38.7, 36.7, 25.5, 24.2, 21.6; IR (film): 2923, 1494, 1329, 1225, 1160 cm^{-1}. HRMS-APCI (*m/z*) $[M]^+$ calculated for $C_{23}H_{28}N_2O_3S^+$, 412.1815; found 412.1800.

The structure of **50** was verified by 2D-NOESY, as the following interaction was observed:

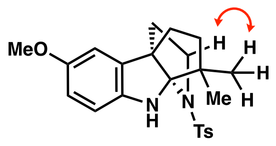

50

13.3. Synthesis of Ketone 42

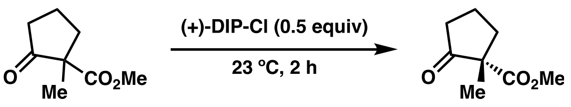

(±)-37 *(+)-37* *90% ee*

Ketone (+)-**37.** Enantioenriched (+)-**37** was prepared from racemic (±)-**37**[4] according to the reported procedure.[5] **Ketone** (+)-**37:** ^{1}H NMR (600 MHz, $CDCl_3$): δ 3.70 (s, 3H), 2.51 (dt, J = 12.9, 5.8 Hz, 1H), 2.43 (ddd, J = 18.6, 8.9, 6.9 Hz, 1H), 2.32 (ddd, J = 18.6, 8.4, 6.3 Hz, 1H), 2.08–2.05 (m, 1H), 1.96–1.89 (m, 1H), 1.88–1.83 (m, 1H), 1.31 (s, 3H); $[\alpha]^{25.1}_{D}$ = +10.2 (c = 1.00, $CHCl_3$, 589 nm). Enantiomeric excess (ee) of (+)-**37** was determined to be 90% by comparison of optical rotation value to the reported value.[5]

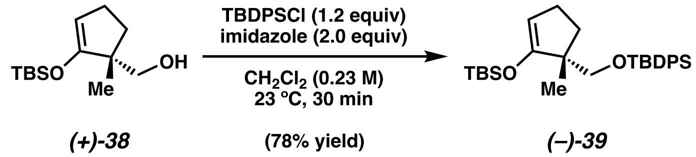

Silyl Ether (–)-**39.** To a solution of alcohol (+)-**38** (440.0 mg, 1.82 mmol, 1.0 equiv) in CH_2Cl_2 (8 mL) in a 20 mL scintillation vial equipped with a stir bar was added imidazole (247.1 mg, 3.63 mmol, 2.0 equiv) at 23 ºC. Then, *tert*-butyldiphenylchlorosilane (0.56 mL, 2.18 mmol, 1.2 equiv) was added dropwise via syringe over 5 min. After stirring at 23 ºC for 30 min under ambient atmosphere, the reaction was quenched by the slow addition of saturated aq. $NaHCO_3$ (10 mL) over 1 min. The mixture was transferred to a separatory funnel and the layers were separated. The aqueous layer was extracted with hexanes (3 x 10 mL). Then, the organic extracts were combined, dried over Na_2SO_4 and concentrated under reduced pressure. The crude material was purified by flash chromatography (SiO_2, 5% hexanes:EtOAc) to afford silyl ether (–)-**39** (679.2 mg, 78% yield) as a yellow oil. **Silyl Ether** (–)-**39:** R_f = 0.78 (9:1 hexanes:EtOAc); ^{1}H NMR (600 MHz, $CDCl_3$): δ 7.68 (dd, *J* = 7.9, 1.3 Hz, 4H), 7.44–7.40 (m, 2H), 7.40–7.35 (m, 4H), 4.56 (t, *J* = 2.1 Hz, 1H), 3.59 (d, *J* = 9.0 Hz, 1H), 3.38 (d, *J* = 9.6 Hz, 1H), 2.27–2.20 (m, 1H), 2.20–2.14 (m, 2H), 1.59 (ddd, *J* = 9.7, 8.2 Hz, 1H), 1.06 (s, 9H), 0.99 (s, 3H), 0.89 (s, 9H), 0.15 (s, 3H), 0.13 (s, 3H); ^{13}C NMR (150 MHz, $CDCl_3$): δ 157.5, 135.7, 135.7, 134.2, 134.1, 129.4, 129.4, 127.5, 127.5, 100.2, 68.8, 49.3, 32.7, 29.7, 26.9, 25.7, 21.4, 19.5, 18.0, -4.7, -5.5; IR (film): 3072, 2930, 2858, 1649, 1428 cm^{-1}; HRMS-APCI (*m/z*) [M+H]$^+$ calculated for $C_{29}H_{45}O_2Si_2^+$, 481.2958; found 481.2940; $[\alpha]^{25.4}_D$ = –4.65 (c 1.00, $CHCl_3$, 589 nm).

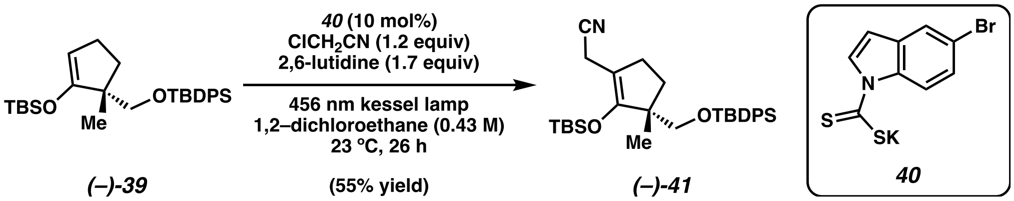

Nitrile (–)-**41.** An 8-dram vial equipped with a stir bar and a septum containing silyl enol ether (–)-**39** (647.5 mg, 1.30 mmol, 1.0 equiv) and photocatalyst **40** (41.8 mg, 0.14 mmol, 10 mol%) was evacuated and backfilled with N_2 three times. Then, 2,6-lutidine (0.27 mL, 2.29 mmol, 1.7 equiv) and 1,2-dichloroethane (3.0 mL) were added sequentially via syringe, each in one portion. Then, 2-chloroacetonitrile (90 μL, 1.41 mmol, 1.2 equiv) was added via syringe in one portion. The vial was sealed with a Teflon-lined cap under rapid N_2 flow and irradiated with a Kessel lamp (456 nm, 100% intensity, ~ 10 cm distance to the vial) and the reaction was stirred at 900 rpm at 23 ºC for 26 h. Then, the reaction was diluted with hexanes (40 mL), and the resulting heterogeneous mixture was filtered through a fritted funnel, eluting with hexanes (10 mL). The filtrate was concentrated under reduced pressure. The crude material was purified via flash chromatography (SiO_2, 0→3% hexanes:Et_2O) to provide nitrile (–)-**41** (389.2 mg, 55% yield) as yellow oil. **Nitrile** (–)-**41:** R_f = 0.68 (3:1 hexanes/EtOAc); ^{1}H NMR (600 MHz, $CDCl_3$): δ 7.66–7.62 (m, 4H), 7.44–7.40 (m, 2H), 7.39–7.35 (m, 4H), 3.53 (d, *J* = 10.3 Hz, 1H), 3.35 (d, *J* = 10.3 Hz, 1H), 3.18 (d, *J* = 18.5 Hz, 1H), 3.02 (d, *J* = 18.5 Hz, 1H), 2.37–2.27 (m, 2H), 2.21–2.14 (m, 1H), 1.65–1.58 (m, 1H), 1.05 (s, 9H), 0.99 (s, 3H), 0.90 (s, 9H), 0.12 (s, 6H); ^{13}C NMR (150 MHz, $CDCl_3$): δ 154.1, 135.8, 135.8, 133.9, 133.8, 129.7, 127.7, 117.7, 105.0, 68.8, 50.1, 31.8, 28.7, 30.0, 26.0, 21.6, 19.5, 18.6, 16.4, –3.1, –3.3; IR (film): 2932, 2858, 1682, 1473, 1255 cm^{-1}; HRMS-APCI (*m/z*); [M+H]$^+$ calculated for $C_{31}H_{46}NO_2Si_2^+$, 520.3067; found 520.3067; $[\alpha]^{25.3}_D$ = –15.6 (c 1.00, $CHCl_3$, 589 nm).

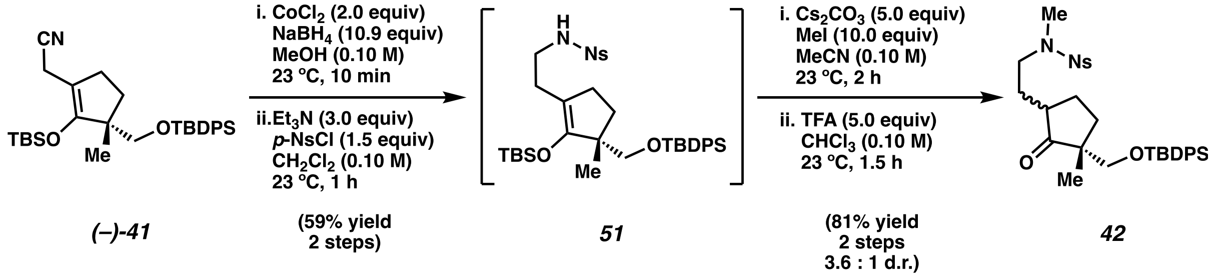

Ketone 42. To an 8-dram vial equipped with a stir bar containing a solution of nitrile (–)-**41** (165.2 mg, 0.32 mmol, 1.0 equiv) in methanol (3.1 mL) was added cobalt (II) chloride hexahydrate (151.2 mg, 0.64 mmol, 2.0 equiv). Then, granular sodium borohydride (132.2 mg, 3.49 mmol, 10.9 equiv) was added in three portions under ambient atmosphere. Upon completion of addition, the vial was quickly sealed with a Teflon-lined cap and stirred at 23 ºC for 10 mins. The reaction mixture was then transferred into a separatory funnel containing saturated aq. NH_4Cl (60 mL) and EtOAc (50 mL). The layers were separated, and the aqueous layer was extracted with EtOAc (3 x 20 mL). The organic extracts were combined, dried over Na_2SO_4, filtered and concentrated under reduced pressure. The crude material was azeotroped with hexanes (2 x 2 mL) under reduced pressure and used directly in the next step without further purification.

The crude material was dissolved in CH_2Cl_2 (3.1 mL), and triethylamine (96.5 mg, 0.95 mmol, 3.0 equiv) and 4-nitrobenzenesulfonylchloride (105.6 mg, 0.48 mmol, 1.5 equiv) were sequentially added at 23 ºC, each in one portion, under ambient atmosphere. After stirring at 23 ºC for 1 h, the reaction was quenched by the addition of saturated aq. $NaHCO_3$ (10 mL). The mixture was transferred to a separatory funnel and the layers were separated. The aqueous layer was extracted with hexanes (3 x 10 mL). The organic layers were combined, dried over Na_2SO_4, filtered, and concentrated under reduced pressure. The crude material was purified by flash chromatography (SiO_2, 0→6% hexanes:EtOAc) to provide sulfonamide **51** (133.6 mg, 0.19 mmol, 59% yield over 2 steps), which was directly used in the subsequent step.

In an 8-dram vial equipped with a stir bar, sulfonamide **51** from the above step (122.6 mg, 0.17 mmol, 1.0 equiv) was dissolved in acetonitrile (1.75 mL). Then, solid Cs_2CO_3 (281.7 mg, 0.86 mmol, 5.0 equiv) was added, followed by the dropwise addition of methyl iodide (122.7 mg, 54 µL, 0.86 mmol, 5.0 equiv) over 1 min under ambient atmosphere. The reaction was stirred at 23 ºC for 1.5 h, then additional methyl iodide (122.7 mg, 54 µL, 0.86 mmol, 5.0 equiv) was added dropwise over 1 min. After stirring at 23ºC for another 30 mins, the reaction mixture was filtered through a plug of celite, eluting with ethyl acetate (2 x 7.5 mL). The filtrate was concentrated under reduced pressure. The crude material was transferred into a scintillation vial equipped with a stir bar and dissolved in $CHCl_3$ (1.6 mL). Then, trifluoroacetic acid (0.07 mL, 0.086 mmol, 5.0 equiv) was added in one portion and the resulting mixture was stirred under ambient atmosphere at 23 ºC for 1.5 h. The reaction was quenched with saturated aq. $NaHCO_3$ (3 mL) and the organic layer was collected. The aqueous layer was extracted with CH_2Cl_2 (4 x 2 mL). The organic extracts were combined, dried over Na_2SO_4, filtered, and concentrated under reduced pressure. The crude material was purified via flash chromatography (SiO_2, 25% hexanes:EtOAc) to afford ketone **42** as a yellow oil (85.3 mg, 0.14 mmol, 81% yield, 3.6:1 d.r.). **Ketone 42:** R_f = 0.50 (benzene); ^1H NMR (600 MHz, $CDCl_3$): δ 8.42–8.32 (m, 2 H), 7.99–7.92 (m, 2H), 7.65–7.58 (m, 4H), 7.46–7.36 (m, 6H), 3.80–3.70 (m, 1H), 3.40–3.32 (m, 1H), 3.31–3.24 (m, 1H), 3.12–3.01 (m, 1H), 2.80–2.74 (m, 3H), 2.43–2.29 (m, 2H), 2.30–2.17 (m, 1H), 2.12–1.98 (m, 1H), 1.77–1.64 (m, 2H), 1.5–1.42 (m, 1H), 1.03–0.99 (m, 9H), 0.95 (s, 2H), 0.86 (s, 1H); ^{13}C NMR (150 MHz, $CDCl_3$): δ 223.1, 222.3, 150.1, 143.49, 143.46, 135.64, 135.62, 135.6, 133.3, 133.0, 132.9, 130.0, 129.8, 129.8, 129.74, 129.72, 128.9, 128.53, 128.48, 127.8, 127.73, 127.71, 124.4, 77.2, 69.2, 68.2, 53.4, 51.1, 50.8, 48.43, 48.36, 47.9, 45.7, 34.5, 32.3, 31.9, 30.6, 29.7, 29.5, 29.33, 29.25,

29.1, 27.8, 27.6, 26.81, 26.80, 26.6, 25.7, 22.7, 21.2, 19.34, 19.22, 19.2; IR (film): 2931, 2859, 1734, 1531, 1349 cm^{-1}; HRMS-APCI (*m/z*) $[M+H]^+$ calculated for $C_{32}H_{41}N_2O_6SSi^+$, 609.2455; found 609.2470.

*Note: Ketone **42** was obtained as a mixture of diastereomers. Multiple stereoisomers are present in the ^{1}H NMR spectra. The structures are indicated on the spectrum, and the ratio of each isomer is determined by integration of the ^{1}H NMR spectrum of the crude product.*

13.4. Diastereoselective Interrupted Fischer Indolization/End Game

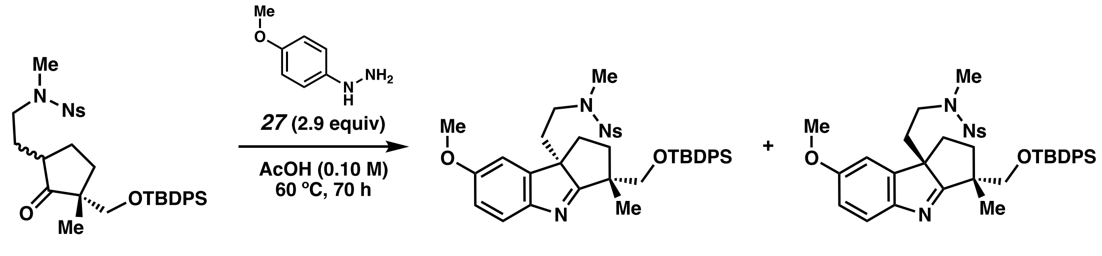

42

(60% yield
2.3 : 1 dr)

44a
(major)

44b
(minor)

Indolenine 44a and **44b.** To a 2-dram vial equipped with a stir bar containing ketone **42** (72 mg, 0.12 mmol, mixture of diastereomers, 1.0 equiv) was added 4-methoxyphenylhydrazine **27** (49 mg, 0.35 mmol, 2.9 equiv) in the glovebox. The vial was sealed with a septum and brought out of the glovebox. Acetic acid (1.2 mL) was then added via syringe in one portion, and the resulting solution was stirred under N_2 at 60 ºC for 70 h. The reaction mixture was then cooled to 23 ºC, and the reaction mixture was transferred to a separatory funnel containing saturated aq. $NaHCO_3$ (20 mL) and EtOAc (5 mL) and layers were separated. The aqueous layer was extracted with EtOAc (2 x 5 mL) and the organic extracts were combined, dried over Na_2SO_4, filtered, and concentrated under reduced pressure. The crude material was purified by preparative thin layer chromatography (SiO_2, 7:3 hexanes:EtOAc) followed by flash chromatography (SiO_2, 0 →17% hexanes:EtOAc) to afford indolenine **44a** and **44b** (50 mg, 0.72 mmol, 60% yield, 2.3:1 d.r.) as a colorless oil. **Indolenine 44a** and **44b:** R_f = 0.20 (5:1 hexanes:EtOAc); ^{1}H NMR (600 MHz, $CDCl_3$): δ 8.32–8.14 (m, 3H), 7.78–7.73 (m, 1H), 7.70 (dt, *J* = 6.7, 1.5 Hz, 2H), 7.67 (dt, *J* = 6.7, 1.6 Hz, 3H), 7.64–7.61 (m, 1H), 7.61–7.56 (m, 2H), 7.44–7.33 (m, 10H), 6.91–6.76 (m, 3H), 3.85–3.80 (m, 6H), 2.78–2.69 (m, 2H), 2.67 (s, 1H), 2.28–2.10 (m, 6H), 1.99 (dt, *J* = 12.7, 6.3 Hz, 2H), 1.47 (s, 1H), 1.41 (s, 3H), 1.40–1.31 (m, 2H), 1.09 (s, 9H), 0.97 (s, 4H); ^{13}C NMR (150 MHz, $CDCl_3$): δ 198.0, 197.9, 158.2, 158.0, 152.0, 152.0, 150.1, 150.0, 144.3, 143.9, 143.6, 143.3, 135.9, 135.8, 135.8, 133.7, 133.5, 133.5, 133.4, 130.0, 129.9, 129.7, 128.4, 128.4, 127.9, 127.9, 127.8, 127.7, 124.4, 124.3, 121.1, 120.9, 113.2, 112.9, 109.5, 109.4, 69.8, 69.7, 64.5, 64.3, 55.9, 55.9, 46.4, 46.1, 43.6, 42.1, 40.4, 39.6, 35.7, 35.3, 34.4, 33.3, 30.2, 29.0, 27.0, 26.9, 23.7, 22.8, 19.6, 19.4; IR (film): 2958, 2858, 1589, 1531, 1466, 1350 cm^{-1}; HRMS-APCI (*m/z*) $[M+H]^+$ calculated for $C_{39}H_{46}N_3O_6SSi^+$, 712.2871; found 712.2936.

*Note: Indolenines **44a** and **44b** were obtained as a mixture of diastereomers. Multiple stereoisomers are present in the ^{1}H NMR spectrum. The structures are indicated on the spectrum, and the ratio of each isomer is determined by integration of the ^{1}H NMR spectrum of the crude product.*

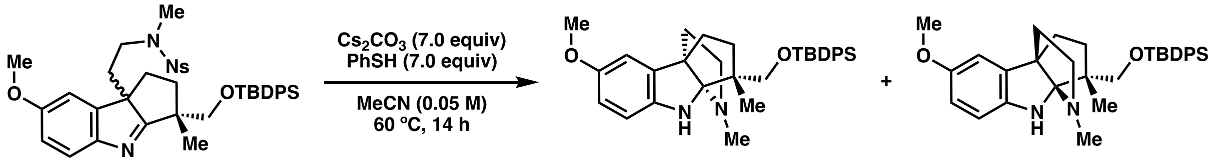

44a,b (2.3 : 1 d.r.) — **(–)-45** (61% yield) — **(+)-52** (15% yield)

Indoline (–)-45. A suspension of indolenine **44a** and **44b** (40 mg, 0.56 mmol, 2.3:1 d.r., 1.0 equiv) and Cs_2CO_3 (130 mg, 0.39 mmol, 7.0 equiv) in MeCN (1.1 mL) in a 2-dram vial equipped with a stir bar was sparged with N_2 for 5 min. Then, thiophenol (40 µL, 0.39 mmol, 7.0 equiv) was added via micro syringe in one portion. The reaction mixture was heated to 60 ºC and stirred for 14 h. The reaction mixture was cooled to 23 ºC, and diluted with Et_2O (2 mL). The mixture was passed through a plug of celite, eluting with Et_2O (4 x 2 mL). The filtrate was concentrated under reduced pressure. The crude material was purified by preparative thin layer chromatography (SiO_2, 30% hexanes:EtOAc) followed by flash chromatography (SiO_2, 0→15% hexanes:EtOAc) to afford indoline (–)-**45** (18 mg, 61% yield) and indoline (+)-**52** (4.3 mg, 15% yield) as colorless oils. **Indoline** (–)-**45:** R_f = 0.44 (5:1 hexanes:EtOAc). ^{1}H NMR (600 MHz, $CDCl_3$): δ 7.71–7.68 (m, 2H), 7.68–7.64 (m, 2H), 7.45–7.41 (m, 2H), 7.41–7.36 (m, 4H), 6.62 (d, J = 2.5 Hz, 1H), 6.58 (dd, J = 8.4, 2.6 Hz, 1H), 6.44 (d, J = 8.4 Hz, 1H), 3.82 (d, J = 10.2 Hz, 1H), 3.74 (s, 3H), 3.56 (d, J = 10.2 Hz, 1H), 2.78 (dd, J = 9.1, 6.5 Hz, 1H), 2.34 (m, 1H, overlapping signal), 2.33 (s, 3H, overlapping signal), 1.94 (dd, J = 11.7, 5.1 Hz, 1H), 1.89–1.83 (m, 1H), 1.83–1.75 (m, 3H), 1.29–1.22 (m, 1H), 1.11 (s, 9H), 0.95 (s, 3H); ^{13}C NMR (150 MHz, $CDCl_3$): δ 153.1, 146.2, 137.4, 136.1, 136.0, 133.9, 133.9, 129.8, 129.7, 127.8, 127.7, 112.5, 110.3, 107.5, 99.5, 68.1, 67.5, 56.9, 56.1, 51.6, 41.0, 37.6, 36.3, 35.9, 27.3, 19.5, 19.3; IR (film): 3420, 2933, 2858, 1493, 1028 cm^{-1}; HRMS-APCI (*m/z*) [M+H]$^+$ calculated for $C_{33}H_{43}N_2O_2Si^+$, 527.3088; found 527.3136; $[\alpha]_D^{25.2}$ = –49.3 (c 1.20, $CHCl_3$, 589 nm). **Indoline** (+)-**52:** R_f = 0.50 (3:2 Hexanes:EtOAc); ^{1}H NMR (600 MHz, $CDCl_3$): δ 7.66 (d, J = 6.8 Hz, 2H), 7.61–7.56 (d, J = 6.8 Hz, 2H), 7.45–7.40 (m, 2H), 7.39–7.36 (m, 2H), 7.34 (t, J = 7.5 Hz, 2H), 6.60 (s, 1H, overlapping signal), 6.60–6.58 (m, 1H, overlapping signal), 6.37–6.32 (dd, J = 6.7, 2.4 Hz, 1H), 4.77 (brs, 1H), 3.75 (s, 3H), 3.58 (d, J = 10.4 Hz, 1H), 3.28 (d, J = 10.4 Hz, 1H), 2.96–2.87 (m, 1H), 2.44 (m, 1H), 2.41 (s, 3H), 1.98–1.94 (m, 2H), 1.91 (dt, J = 12.6, 6.4 Hz, 1H), 1.69 (ddd, J = 12.3, 8.2, 6.3 Hz, 1H), 1.65–1.56 (m, 1H, signal overlapping with HDO), 1.35 (dt, J = 12.7, 6.3 Hz, 1H), 1.25 (s, 3H), 1.09 (s, 9H); ^{13}C NMR (150 MHz, $CDCl_3$) δ 153.0, 146.3, 137.5, 134.0, 135.7, 133.2, 132.9, 130.0, 129.9, 128.5, 127.9, 127.9, 112.7, 110.1, 108.1, 100.3, 69.6, 68.8, 56.8, 56.2, 51.8, 40.5, 38.8, 37.6, 35.7, 27.1, 20.8, 19.5; IR (film): 3375, 2991, 2870, 1492, 1143 cm^{-1}; HRMS-APCI (*m/z*) [M+H]$^+$ calculated for $C_{33}H_{43}N_2O_2Si^+$, 527.3088; found 527.3134; $[\alpha]^{25.2}_D$ = +59.8 (c 0.29, CH_2Cl_2, 589 nm).

The structure of (–)-**45** was verified by 2D-NOESY, as the following interaction was observed:

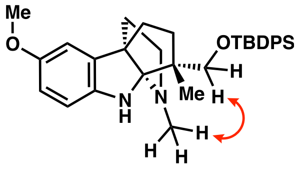

(–)-45

The structure of (+)-**52** was verified by 2D-NOESY, as the following interaction was observed:

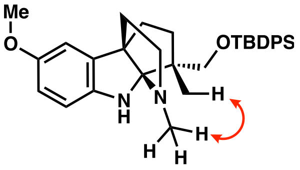

(+)-52

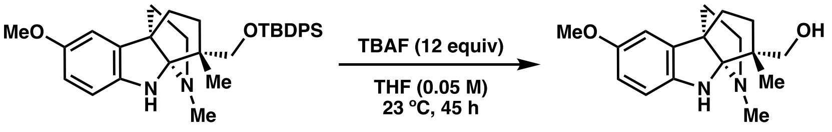

(–)-45 — **TBAF (12 equiv)**, THF (0.05 M), 23 °C, 45 h (73% yield) — *(–)-Pensubrubine (4)*

(–)-**Pensubrubine (4).** To a 1-dram vial containing indoline (–)-**45** (18 mg, 0.034 mmol, 1.0 equiv) equipped with a stir bar was added THF (0.4 mL) via syringe. The solution was sparged with N_2 for 5 min, before TBAF (0.41 mL, 1.0 M in THF, 0.41 mmol, 12 equiv) was added via syringe in one portion. The reaction mixture was stirred at 23 ºC for 45 h. The solvent was then removed under reduced pressure. The crude material was purified by preparative thin layer chromatography (SiO_2, 1% NH_4OH in EtOAc) to afford (–)-**pensubrubine (4)** (7.2 mg, 0.025 mmol, 73% yield) as a colorless foam. (–)-**Pensubrubine (4):** R_f = 0.44 (1% NH_4OH in EtOAc); ^{1}H NMR (600 MHz, CD_3OD): δ 6.62 (d, J = 2.6 Hz, 1H), 6.55 (dd, J = 8.4, 2.6 Hz, 1H), 6.38 (d, J = 8.4 Hz, 1H), 3.69 (s, 3H), 3.65 (d, J = 11.2 Hz, 1H), 3.63 (d, J = 11.2 Hz, 1H), 2.91 (dd, J = 9.1, 6.0 Hz, 1H), 2.37 (s, 3H), 2.32 (ddd, J = 12.2, 9.2, 5.5 Hz, 1H), 2.08–1.94 (m, 4H), 1.82–1.74 (m, 1H), 1.45–1.38 (m, 1H), 0.91 (s, 3H); ^{13}C NMR (150 MHz, CD_3OD): δ 154.1, 147.3, 137.4, 114.2, 110.8, 107.8, 101.2, 69.4, 68.1, 57.8, 56.6, 51.3, 41.0, 38.1, 37.5, 36.2, 19.8; IR (film): 3384, 2991, 2871, 1493, 1143 cm^{-1}; HRMS-APCI (m/z) $[M+H]^+$ calculated for $C_{17}H_{25}N_2O_2^+$, 289.1911; found 289.1915; $[\alpha]^{25.2}_D$ = –86.7 (c 0.48, CH_2Cl_2, 589 nm). All characterization data of the synthetic (–)-**4** match that of the biosynthetic sample (See Page S34, Table S11).

The structure of (–)-pensubrubine (**4**) was verified by 2D-NOESY, as the following interaction was observed:

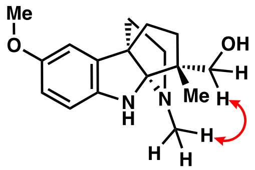

(–)-Pensubrubine (4)

Supplementary Tables

Table S1. *Aspergillus* OYEs identified via NCBI Blast.

NCBI Protein ID	Strain	Residue	NCBI Protein ID	Strain	Residue	NCBI Protein ID	Strain	Residue
4QNW A	*Aspergillus fumigatus*	Y	KAH2904598.1	*Aspergillus fumigatus*	-	XP 025447737.1	*Aspergillus brunneoviolaceus CBS 621.78*	Y
7QFX B	*Aspergillus niger*	Y	KAH3027167.1	*Aspergillus fumigatus*	Y	XP 025452563.1	*Aspergillus niger CBS 101883*	Y
ADY39251.1	*Aspergillus fumigatus*	Y	KAH3140847.1	*Aspergillus fumigatus*	Y	XP 025453594.1	*Aspergillus niger CBS 101883*	E
B8NWW6.1	*Aspergillus flavus NRRL3357*	Y	KAH3148351.1	*Aspergillus fumigatus*	Y	XP 025455540.1	*Aspergillus niger CBS 101883*	Y
BCS11815.1	*Aspergillus luchuensis*	Y	KAH3200654.1	*Aspergillus fumigatus*	Y	XP 025457913.1	*Aspergillus niger CBS 101883*	Y
BCS16148.1	*Aspergillus luchuensis*	Y	KAH3363111.1	*Aspergillus fumigatus*	Y	XP 025460268.1	*Aspergillus niger CBS 101883*	Y
CAK44043.1	*Aspergillus niger*	Y	KAI2813664.1	*Aspergillus niger*	Y	XP 025461849.1	*Aspergillus sclerotioniger CBS 115572*	Y
CEL00569.1	*Aspergillus calidoustus*	Y	KAI2814175.1	*Aspergillus niger*	-	XP 025464079.1	*Aspergillus sclerotioniger CBS 115572*	Y
CEL02159.1	*Aspergillus calidoustus*	Y	KAI2814413.1	*Aspergillus niger*	Y	XP 025464850.1	*Aspergillus sclerotioniger CBS 115572*	Y
CEL03463.1	*Aspergillus calidoustus*	Y	KAI2815553.1	*Aspergillus niger*	Y	XP 025467919.1	*Aspergillus sclerotioniger CBS 115572*	Y
CEL05232.1	*Aspergillus calidoustus*	Y	KAI2815842.1	*Aspergillus niger*	Y	XP 025470751.1	*Aspergillus sclerotioniger CBS 115572*	Y
CEL06147.1	*Aspergillus calidoustus*	Y	KAI2825919.1	*Aspergillus niger*	-	XP 025470988.1	*Aspergillus sclerotioniger CBS 115572*	Y
CEL10148.1	*Aspergillus calidoustus*	Y	KAI2830296.1	*Aspergillus niger*	Y	XP 025472824.1	*Aspergillus sclerotioniger CBS 115572*	Y
CEL10327.1	*Aspergillus calidoustus*	Y	KAI2830726.1	*Aspergillus niger*	Y	XP 025472940.1	*Aspergillus sclerotioniger CBS 115572*	Y
CEL10850.1	*Aspergillus calidoustus*	Y	KAI2831831.1	*Aspergillus niger*	Y	XP 025474773.1	*Aspergillus neoniger CBS 115656*	Y
CEL11162.1	*Aspergillus calidoustus*	F	KAI2832461.1	*Aspergillus niger*	Y	XP 025476389.1	*Aspergillus neoniger CBS 115656*	Y
CEL11539.1	*Aspergillus calidoustus*	Y	KAI2840411.1	*Aspergillus niger*	Y	XP 025476568.1	*Aspergillus neoniger CBS 115656*	Y
CEN59348.1	*Aspergillus calidoustus*	Y	KAI2844357.1	*Aspergillus niger*	Y	XP 025477257.1	*Aspergillus neoniger CBS 115656*	Y
CEN59920.1	*Aspergillus calidoustus*	Y	KAI2850053.1	*Aspergillus niger*	-	XP 025477449.1	*Aspergillus neoniger CBS 115656*	Y
CEN60398.1	*Aspergillus calidoustus*	Y	KAI2865214.1	*Aspergillus niger*	Y	XP 025477713.1	*Aspergillus neoniger CBS 115656*	Y
CEN60982.1	*Aspergillus calidoustus*	Y	KAI2865996.1	*Aspergillus niger*	Y	XP 025478138.1	*Aspergillus neoniger CBS 115656*	Y
CEN61141.1	*Aspergillus calidoustus*	Y	KAI2884067.1	*Aspergillus niger*	Y	XP 025478820.1	*Aspergillus neoniger CBS 115656*	Y
CEN61325.1	*Aspergillus calidoustus*	Y	KAI2887754.1	*Aspergillus niger*	Y	XP 025480593.1	*Aspergillus neoniger CBS 115656*	Y
EAA63084.1	*Aspergillus nidulans FGSC A4*	Y	KAI2889682.1	*Aspergillus niger*	Y	XP 025481151.1	*Aspergillus neoniger CBS 115656*	Y
EDP54088.1	*Aspergillus fumigatus A1163*	Y	KAI2893042.1	*Aspergillus niger*	Y	XP 025485900.1	*Aspergillus uvarum CBS 121591*	Y
EDP55299.1	*Aspergillus fumigatus A1163*	Y	KAI2893787.1	*Aspergillus niger*	Y	XP 025486105.1	*Aspergillus uvarum CBS 121591*	Y
EHA18401.1	*Aspergillus niger ATCC 1015*	Y	KAI2895102.1	*Aspergillus niger*	Y	XP 025487164.1	*Aspergillus uvarum CBS 121591*	Y
EHA19907.1	*Aspergillus niger ATCC 1015*	Y	KAI2896339.1	*Aspergillus niger*	Y	XP 025487196.1	*Aspergillus uvarum CBS 121591*	Y
EHA20973.1	*Aspergillus niger ATCC 1015*	Y	KAI2908224.1	*Aspergillus niger*	Y	XP 025487599.1	*Aspergillus uvarum CBS 121591*	Y
EHA21620.1	*Aspergillus niger ATCC 1015*	Y	KAI2913738.1	*Aspergillus niger*	-	XP 025488877.1	*Aspergillus uvarum CBS 121591*	Y
EHA21687.1	*Aspergillus niger ATCC 1015*	Y	KAI2916817.1	*Aspergillus niger*	Y	XP 025490730.1	*Aspergillus uvarum CBS 121591*	Y
EHA22639.1	*Aspergillus niger ATCC 1015*	Y	KAI2922042.1	*Aspergillus niger*	Y	XP 025490835.1	*Aspergillus uvarum CBS 121591*	Y
EHA23348.1	*Aspergillus niger ATCC 1015*	Y	KAI2922892.1	*Aspergillus niger*	Y	XP 025491780.1	*Aspergillus uvarum CBS 121591*	Y
EHA26139.1	*Aspergillus niger ATCC 1015*	F	KAI2926148.1	*Aspergillus niger*	Y	XP 025495970.1	*Aspergillus uvarum CBS 121591*	Y
EHA27961.1	*Aspergillus niger ATCC 1015*	Y	KAI2935787.1	*Aspergillus niger*	Y	XP 025499230.1	*Aspergillus aculeatinus CBS 121060*	Y
EIT73296.1	*Aspergillus oryzae 3.042*	Y	KAI2961106.1	*Aspergillus niger*	Y	XP 025499597.1	*Aspergillus aculeatinus CBS 121060*	Y
EIT73387.1	*Aspergillus oryzae 3.042*	Y	KAI2961725.1	*Aspergillus niger*	Y	XP 025500137.1	*Aspergillus aculeatinus CBS 121060*	Y
EIT76912.1	*Aspergillus oryzae 3.042*	Y	KAI2963030.1	*Aspergillus niger*	Y	XP 025501118.1	*Aspergillus aculeatinus CBS 121060*	Y
EIT77670.1	*Aspergillus oryzae 3.042*	Y	KAI2990652.1	*Aspergillus niger*	Y	XP 025501964.1	*Aspergillus aculeatinus CBS 121060*	Y
EIT78852.1	*Aspergillus oryzae 3.042*	Y	KAI2994127.1	*Aspergillus niger*	Y	XP 025502558.1	*Aspergillus aculeatinus CBS 121060*	Y
EIT79709.1	*Aspergillus oryzae 3.042*	Y	KAI2996946.1	*Aspergillus niger*	Y	XP 025502617.1	*Aspergillus aculeatinus CBS 121060*	Y
GAA83853.1	*Aspergillus luchuensis IFO 4308*	Y	KAI3002789.1	*Aspergillus niger*	Y	XP 025502966.1	*Aspergillus aculeatinus CBS 121060*	Y
GAQ05910.1	*Aspergillus lentulus*	Y	KAI3003304.1	*Aspergillus niger*	Y	XP 025504256.1	*Aspergillus aculeatinus CBS 121060*	-
GAQ07144.1	*Aspergillus lentulus*	C	KAI3005113.1	*Aspergillus niger*	Y	XP 025506762.1	*Aspergillus aculeatinus CBS 121060*	Y
GAQ08751.1	*Aspergillus lentulus*	Y	KAI3005983.1	*Aspergillus niger*	Y	XP 025508956.1	*Aspergillus aculeatinus CBS 121060*	Y
GAQ09921.1	*Aspergillus lentulus*	Y	KAI3008954.1	*Aspergillus niger*	Y	XP 025509285.1	*Aspergillus aculeatinus CBS 121060*	Y
GAQ35137.1	*Aspergillus niger*	Y	KAI3009899.1	*Aspergillus niger*	Y	XP 025509551.1	*Aspergillus piperis CBS 112811*	Y
GAQ35665.1	*Aspergillus niger*	Y	KAI3010493.1	*Aspergillus niger*	Y	XP 025510454.1	*Aspergillus piperis CBS 112811*	Y
GAQ39922.1	*Aspergillus niger*	Y	KAI3010792.1	*Aspergillus niger*	Y	XP 025511662.1	*Aspergillus piperis CBS 112811*	Y
GAQ46970.1	*Aspergillus niger*	Y	KAI3011373.1	*Aspergillus niger*	Y	XP 025512027.1	*Aspergillus piperis CBS 112811*	Y
GCB17738.1	*Aspergillus awamori*	Y	KAI3013266.1	*Aspergillus niger*	Y	XP 025512598.1	*Aspergillus piperis CBS 112811*	Y
GCB18161.1	*Aspergillus awamori*	Y	KAI3041352.1	*Aspergillus niger*	Y	XP 025513669.1	*Aspergillus piperis CBS 112811*	Y
GCB19122.1	*Aspergillus awamori*	Y	KAI3063012.1	*Aspergillus niger*	Y	XP 025514146.1	*Aspergillus piperis CBS 112811*	Y
GCB20777.1	*Aspergillus awamori*	Y	KAI3071364.1	*Aspergillus niger*	Y	XP 025515278.1	*Aspergillus piperis CBS 112811*	Y
GCB21346.1	*Aspergillus awamori*	S	KEY79286.1	*Aspergillus fumigatus var. RP-2014*	Y	XP 025517462.1	*Aspergillus piperis CBS 112811*	Y
GCB21780.1	*Aspergillus awamori*	F	KIA75349.1	*Aspergillus ustus*	Y	XP 025518568.1	*Aspergillus piperis CBS 112811*	Y
GCB22986.1	*Aspergillus awamori*	Y	KIA75421.1	*Aspergillus ustus*	Y	XP 025521100.1	*Aspergillus piperis CBS 112811*	Y
GCB25384.1	*Aspergillus awamori*	Y	KJJ30539.1	*Aspergillus flavus AF70*	Y	XP 025521446.1	*Aspergillus piperis CBS 112811*	Y
GCB25555.1	*Aspergillus awamori*	F	KJJ34500.1	*Aspergillus flavus AF70*	Y	XP 025522470.1	*Aspergillus japonicus CBS 114.51*	Y
GCB25568.1	*Aspergillus awamori*	F	KJK60782.1	*Aspergillus parasiticus SU-1*	Y	XP 025522892.1	*Aspergillus japonicus CBS 114.51*	Y
GCB26244.1	*Aspergillus awamori*	Y	KJK63372.1	*Aspergillus parasiticus SU-1*	Y	XP 025523211.1	*Aspergillus japonicus CBS 114.51*	Y
GES60471.1	*Aspergillus terreus*	Y	KJK64656.1	*Aspergillus parasiticus SU-1*	Y	XP 025526073.1	*Aspergillus japonicus CBS 114.51*	Y
GES63623.1	*Aspergillus terreus*	Y	KJK68167.1	*Aspergillus parasiticus SU-1*	Y	XP 025526360.1	*Aspergillus japonicus CBS 114.51*	Y
GES63988.1	*Aspergillus terreus*	Y	KKK12179.1	*Aspergillus rambellii*	Y	XP 025527482.1	*Aspergillus japonicus CBS 114.51*	Y
GES65215.1	*Aspergillus terreus*	Y	KKK16043.1	*Aspergillus ochraceoroseus*	Y	XP 025528103.1	*Aspergillus japonicus CBS 114.51*	Y
GES66310.1	*Aspergillus terreus*	Y	KOC08147.1	*Aspergillus flavus AF70*	Y	XP 025531309.1	*Aspergillus japonicus CBS 114.51*	Y
GFF27997.1	*Aspergillus udagawae*	Y	KOC08331.1	*Aspergillus flavus AF70*	Y	XP 025532139.1	*Aspergillus japonicus CBS 114.51*	W
GFF29466.1	*Aspergillus udagawae*	Y	KOC14476.1	*Aspergillus flavus AF70*	Y	XP 025533875.1	*Aspergillus costaricaensis CBS 115574*	Y
GFF34507.1	*Aspergillus udagawae*	Y	KOC16569.1	*Aspergillus flavus AF70*	Y	XP 025534880.1	*Aspergillus costaricaensis CBS 115574*	Y
GFF39717.1	*Aspergillus udagawae*	Y	KOC18458.1	*Aspergillus flavus AF70*	Y	XP 025535743.1	*Aspergillus costaricaensis CBS 115574*	Y
GFF43823.1	*Aspergillus udagawae*	Y	ODM14522.1	*Aspergillus cristatus*	Y	XP 025536678.1	*Aspergillus costaricaensis CBS 115574*	Y
GFF47647.1	*Aspergillus udagawae*	Y	ODM14598.1	*Aspergillus cristatus*	Y	XP 025539628.1	*Aspergillus costaricaensis CBS 115574*	Y
GFF47773.1	*Aspergillus udagawae*	Y	ODM15006.1	*Aspergillus cristatus*	Y	XP 025540197.1	*Aspergillus costaricaensis CBS 115574*	Y
GFF48255.1	*Aspergillus udagawae*	Y	ODM15550.1	*Aspergillus cristatus*	Y	XP 025540247.1	*Aspergillus costaricaensis CBS 115574*	Y
GFF53248.1	*Aspergillus udagawae*	Y	ODM16488.1	*Aspergillus cristatus*	Y	XP 025540603.1	*Aspergillus costaricaensis CBS 115574*	Y
GFF55055.1	*Aspergillus udagawae*	Y	ODM19048.1	*Aspergillus cristatus*	Y	XP 025540783.1	*Aspergillus costaricaensis CBS 115574*	Y
GFF56736.1	*Aspergillus lentulus*	Y	ODM19769.1	*Aspergillus cristatus*	Y	XP 025541214.1	*Aspergillus costaricaensis CBS 115574*	Y
GFF58062.1	*Aspergillus udagawae*	Y	ODM20137.1	*Aspergillus cristatus*	Y	XP 025542626.1	*Aspergillus costaricaensis CBS 115574*	Y
GFF58608.1	*Aspergillus udagawae*	Y	ODM22847.1	*Aspergillus cristatus*	Y	XP 025543083.1	*Aspergillus costaricaensis CBS 115574*	Y
GFF62131.1	*Aspergillus lentulus*	Y	OJI81832.1	*Aspergillus tubingensis CBS 134.48*	Y	XP 025546102.1	*Aspergillus homomorphus CBS 101889*	Y
GFF76799.1	*Aspergillus udagawae*	Y	OJI82297.1	*Aspergillus tubingensis CBS 134.48*	Y	XP 025546205.1	*Aspergillus homomorphus CBS 101889*	Y
GFF78251.1	*Aspergillus lentulus*	Y	OJI83219.1	*Aspergillus tubingensis CBS 134.48*	Y	XP 025550372.1	*Aspergillus homomorphus CBS 101889*	Y

GFF79189.1	*Aspergillus udagawae*	Y	OJI83220.1	*Aspergillus tubingensis CBS 134.48*	Y	XP 025550480.1	*Aspergillus homomorphus CBS 101889*	Y
GFF87021.1	*Aspergillus udagawae*	Y	OJI84748.1	*Aspergillus tubingensis CBS 134.48*	Y	XP 025551819.1	*Aspergillus homomorphus CBS 101889*	Y
GFF87682.1	*Aspergillus udagawae*	Y	OJI85582.1	*Aspergillus tubingensis CBS 134.48*	Y	XP 025553659.1	*Aspergillus homomorphus CBS 101889*	Y
GFG01037.1	*Aspergillus lentulus*	Y	OJI86062.1	*Aspergillus tubingensis CBS 134.48*	Y	XP 025554352.1	*Aspergillus homomorphus CBS 101889*	F
GFG08837.1	*Aspergillus udagawae*	Y	OJI88376.1	*Aspergillus tubingensis CBS 134.48*	Y	XP 025557557.1	*Aspergillus vadensis CBS 113365*	W
GFG25902.1	*Aspergillus udagawae*	Y	OJI89396.1	*Aspergillus tubingensis CBS 134.48*	Y	XP 025558775.1	*Aspergillus vadensis CBS 113365*	Y
GFG26589.1	*Aspergillus udagawae*	Y	OJI89935.1	*Aspergillus tubingensis CBS 134.48*	Y	XP 025560169.1	*Aspergillus vadensis CBS 113365*	Y
GFG26761.1	*Aspergillus udagawae*	Y	OJI91293.1	*Aspergillus tubingensis CBS 134.48*	G	XP 025561222.1	*Aspergillus vadensis CBS 113365*	Y
GJP96910.1	*Aspergillus niger*	Y	OJJ65653.1	*Aspergillus brasiliensis CBS 101740*	Y	XP 025562125.1	*Aspergillus vadensis CBS 113365*	Y
KAB8067995.1	*Aspergillus leporis*	F	OJJ65689.1	*Aspergillus brasiliensis CBS 101740*	Y	XP 025563260.1	*Aspergillus vadensis CBS 113365*	Y
KAB8071188.1	*Aspergillus leporis*	Y	OJJ65885.1	*Aspergillus brasiliensis CBS 101740*	Y	XP 025563869.1	*Aspergillus vadensis CBS 113365*	G
KAB8071228.1	*Aspergillus leporis*	Y	OJJ65911.1	*Aspergillus brasiliensis CBS 101740*	Y	XP 025564028.1	*Aspergillus vadensis CBS 113365*	Y
KAB8071285.1	*Aspergillus leporis*	F	OJJ67783.1	*Aspergillus brasiliensis CBS 101740*	Y	XP 025564248.1	*Aspergillus vadensis CBS 113365*	Y
KAB8071424.1	*Aspergillus leporis*	Y	OJJ68229.1	*Aspergillus brasiliensis CBS 101740*	Y	XP 025566836.1	*Aspergillus vadensis CBS 113365*	Y
KAB8073418.1	*Aspergillus leporis*	F	OJJ68438.1	*Aspergillus brasiliensis CBS 101740*	Y	XP 025569183.1	*Aspergillus ibericus CBS 121593*	Y
KAB8073434.1	*Aspergillus leporis*	Y	OJJ70106.1	*Aspergillus brasiliensis CBS 101740*	Y	XP 025570136.1	*Aspergillus ibericus CBS 121593*	Y
KAB8076065.1	*Aspergillus leporis*	Y	OJJ70831.1	*Aspergillus brasiliensis CBS 101740*	Y	XP 025570148.1	*Aspergillus ibericus CBS 121593*	Y
KAB8076213.1	*Aspergillus leporis*	Y	OJJ72209.1	*Aspergillus brasiliensis CBS 101740*	Y	XP 025572153.1	*Aspergillus ibericus CBS 121593*	Y
KAB8077854.1	*Aspergillus leporis*	Y	OJJ72900.1	*Aspergillus brasiliensis CBS 101740*	Y	XP 025573042.1	*Aspergillus ibericus CBS 121593*	Y
KAB8077918.1	*Aspergillus leporis*	Y	OJJ73330.1	*Aspergillus brasiliensis CBS 101740*	Y	XP 025573878.1	*Aspergillus ibericus CBS 121593*	Y
KAB8078081.1	*Aspergillus leporis*	Y	OJJ74972.1	*Aspergillus brasiliensis CBS 101740*	Y	XP 025577591.1	*Aspergillus ibericus CBS 121593*	Y
KAB8078114.1	*Aspergillus leporis*	Y	OJJ75757.1	*Aspergillus brasiliensis CBS 101740*	Y	XP 026598675.1	*Aspergillus mulundensis*	Y
KAB8199090.1	*Aspergillus parasiticus*	Y	OJJ76597.1	*Aspergillus brasiliensis CBS 101740*	Y	XP 026598877.1	*Aspergillus mulundensis*	Y
KAB8200846.1	*Aspergillus parasiticus*	Y	OJJ77276.1	*Aspergillus brasiliensis CBS 101740*	Y	XP 026599248.1	*Aspergillus mulundensis*	Y
KAB8203782.1	*Aspergillus parasiticus*	Y	OJJ78150.1	*Aspergillus brasiliensis CBS 101740*	Y	XP 026599516.1	*Aspergillus mulundensis*	Y
KAB8204017.1	*Aspergillus parasiticus*	Y	OJZ79750.1	*Aspergillus luchuensis CBS 106.47*	Y	XP 026599712.1	*Aspergillus mulundensis*	Y
KAB8206040.1	*Aspergillus parasiticus*	Y	OJZ80185.1	*Aspergillus luchuensis CBS 106.47*	Y	XP 026600149.1	*Aspergillus mulundensis*	Y
KAB8206153.1	*Aspergillus parasiticus*	Y	OJZ84686.1	*Aspergillus luchuensis CBS 106.47*	Y	XP 026600813.1	*Aspergillus mulundensis*	Y
KAB8206815.1	*Aspergillus parasiticus*	Y	OJZ86961.1	*Aspergillus luchuensis CBS 106.47*	Y	XP 026601038.1	*Aspergillus mulundensis*	Y
KAB8208491.1	*Aspergillus parasiticus*	Y	OJZ89210.1	*Aspergillus luchuensis CBS 106.47*	Y	XP 026601288.1	*Aspergillus mulundensis*	Y
KAB8209632.1	*Aspergillus parasiticus*	Y	OJZ90059.1	*Aspergillus luchuensis CBS 106.47*	Y	XP 026605014.1	*Aspergillus mulundensis*	Y
KAB8212278.1	*Aspergillus parasiticus*	Y	OJZ91202.1	*Aspergillus luchuensis CBS 106.47*	Y	XP 026605425.1	*Aspergillus mulundensis*	Y
KAB8214480.1	*Aspergillus novoparasiticus*	Y	OOF89981.1	*Aspergillus carbonarius ITEM 5010*	Y	XP 026608576.1	*Aspergillus mulundensis*	Y
KAB8215100.1	*Aspergillus novoparasiticus*	Y	OOF91819.1	*Aspergillus carbonarius ITEM 5010*	Y	XP 026612299.1	*Aspergillus thermomutatus*	Y
KAB8215538.1	*Aspergillus novoparasiticus*	Y	OOF93151.1	*Aspergillus carbonarius ITEM 5010*	Y	XP 026614066.1	*Aspergillus thermomutatus*	Y
KAB8217201.1	*Aspergillus novoparasiticus*	Y	OOF93956.1	*Aspergillus carbonarius ITEM 5010*	Y	XP 026614319.1	*Aspergillus thermomutatus*	Y
KAB8219460.1	*Aspergillus novoparasiticus*	Y	OOF96830.1	*Aspergillus carbonarius ITEM 5010*	Y	XP 026615262.1	*Aspergillus thermomutatus*	Y
KAB8221140.1	*Aspergillus novoparasiticus*	Y	OOF97622.1	*Aspergillus carbonarius ITEM 5010*	Y	XP 026620606.1	*Aspergillus welwitschiae*	Y
KAB8222069.1	*Aspergillus novoparasiticus*	Y	OOF98720.1	*Aspergillus carbonarius ITEM 5010*	Y	XP 026621018.1	*Aspergillus welwitschiae*	E
KAB8224128.1	*Aspergillus novoparasiticus*	Y	OOO06507.1	*Aspergillus oryzae*	Y	XP 026621568.1	*Aspergillus welwitschiae*	Y
KAB8224573.1	*Aspergillus novoparasiticus*	Y	OOO10638.1	*Aspergillus oryzae*	Y	XP 026622258.1	*Aspergillus welwitschiae*	Y
KAB8225997.1	*Aspergillus novoparasiticus*	Y	OOO11374.1	*Aspergillus oryzae*	Y	XP 026622793.1	*Aspergillus welwitschiae*	Y
KAB8240849.1	*Aspergillus flavus*	Y	PIG69322.1	*Aspergillus arachidicola*	Y	XP 026625204.1	*Aspergillus welwitschiae*	Y
KAB8242432.1	*Aspergillus flavus*	Y	PIG79279.1	*Aspergillus arachidicola*	Y	XP 026626339.1	*Aspergillus welwitschiae*	Y
KAB8243569.1	*Aspergillus flavus*	Y	PIG88166.1	*Aspergillus arachidicola*	Y	XP 026629417.1	*Aspergillus welwitschiae*	Y
KAB8251541.1	*Aspergillus flavus*	Y	PIG89093.1	*Aspergillus arachidicola*	Y	XP 026630912.1	*Aspergillus welwitschiae*	Y
KAB8251911.1	*Aspergillus flavus*	Y	PLN83311.1	*Aspergillus taichungensis*	Y	XP 026631329.1	*Aspergillus welwitschiae*	Y
KAB8259668.1	*Aspergillus pseudonomiae*	Y	PLN86938.1	*Aspergillus taichungensis*	Y	XP 026632132.1	*Aspergillus welwitschiae*	Y
KAB8268132.1	*Aspergillus minisclerotigenes*	Y	PYH87783.1	*Aspergillus ellipticus CBS 707.79*	Y	XP 031893919.1	*Aspergillus alliaceus*	Y
KAB8269357.1	*Aspergillus minisclerotigenes*	Y	PYH89625.1	*Aspergillus ellipticus CBS 707.79*	Y	XP 031895710.1	*Aspergillus alliaceus*	Y
KAB8269681.1	*Aspergillus minisclerotigenes*	Y	PYH90688.1	*Aspergillus ellipticus CBS 707.79*	Y	XP 031896397.1	*Aspergillus alliaceus*	Y
KAB8271625.1	*Aspergillus minisclerotigenes*	Y	PYH94577.1	*Aspergillus ellipticus CBS 707.79*	Y	XP 031898107.1	*Aspergillus alliaceus*	Y
KAB8273570.1	*Aspergillus minisclerotigenes*	Y	PYH95281.1	*Aspergillus ellipticus CBS 707.79*	Y	XP 031898446.1	*Aspergillus alliaceus*	Y
KAB8273718.1	*Aspergillus minisclerotigenes*	Y	PYH97286.1	*Aspergillus ellipticus CBS 707.79*	Y	XP 031899413.1	*Aspergillus alliaceus*	Y
KAB8274460.1	*Aspergillus minisclerotigenes*	Y	PYH98215.1	*Aspergillus ellipticus CBS 707.79*	Y	XP 031900989.1	*Aspergillus alliaceus*	Y
KAB8274952.1	*Aspergillus minisclerotigenes*	Y	PYI03187.1	*Aspergillus sclerotiicarbonarius*	Y	XP 031901981.1	*Aspergillus alliaceus*	Y
KAB8279575.1	*Aspergillus minisclerotigenes*	Y	PYI03523.1	*Aspergillus sclerotiicarbonarius*	Y	XP 031905297.1	*Aspergillus alliaceus*	Y
KAE8145933.1	*Aspergillus avenaceus*	Y	PYI04540.1	*Aspergillus sclerotiicarbonarius*	Y	XP 031907515.1	*Aspergillus pseudotamarii*	Y
KAE8148339.1	*Aspergillus avenaceus*	Y	PYI05788.1	*Aspergillus sclerotiicarbonarius*	Y	XP 031907546.1	*Aspergillus pseudotamarii*	Y
KAE8148876.1	*Aspergillus avenaceus*	Y	PYI06238.1	*Aspergillus sclerotiicarbonarius*	Y	XP 031908966.1	*Aspergillus pseudotamarii*	Y
KAE8149373.1	*Aspergillus avenaceus*	Y	PYI09503.1	*Aspergillus sclerotiicarbonarius*	Y	XP 031911244.1	*Aspergillus pseudotamarii*	Y
KAE8151066.1	*Aspergillus avenaceus*	Y	PYI09779.1	*Aspergillus sclerotiicarbonarius*	Y	XP 031912315.1	*Aspergillus pseudotamarii*	Y
KAE8151189.1	*Aspergillus avenaceus*	Y	PYI10973.1	*Aspergillus sclerotiicarbonarius*	W	XP 031913728.1	*Aspergillus pseudotamarii*	Y
KAE8151966.1	*Aspergillus avenaceus*	Y	PYI10979.1	*Aspergillus sclerotiicarbonarius*	Y	XP 031913983.1	*Aspergillus pseudotamarii*	Y
KAE8153023.1	*Aspergillus avenaceus*	Y	PYI12326.1	*Aspergillus sclerotiicarbonarius*	Y	XP 031914573.1	*Aspergillus pseudotamarii*	Y
KAE8153391.1	*Aspergillus avenaceus*	Y	PYI15782.1	*Aspergillus violaceofuscus CBS 115571*	W	XP 031914938.1	*Aspergillus pseudotamarii*	Y
KAE8153931.1	*Aspergillus avenaceus*	Y	PYI16902.1	*Aspergillus violaceofuscus CBS 115571*	Y	XP 031917080.1	*Aspergillus pseudotamarii*	Y
KAE8154537.1	*Aspergillus avenaceus*	Y	PYI17421.1	*Aspergillus violaceofuscus CBS 115571*	Y	XP 031917903.1	*Aspergillus pseudotamarii*	Y
KAE8157301.1	*Aspergillus tamarii*	Y	PYI17659.1	*Aspergillus violaceofuscus CBS 115571*	Y	XP 031918866.1	*Aspergillus pseudotamarii*	Y
KAE8158103.1	*Aspergillus tamarii*	Y	PYI24414.1	*Aspergillus violaceofuscus CBS 115571*	Y	XP 031919233.1	*Aspergillus pseudotamarii*	Y
KAE8159550.1	*Aspergillus tamarii*	Y	PYI24566.1	*Aspergillus violaceofuscus CBS 115571*	Y	XP 031920296.1	*Aspergillus caelatus*	Y
KAE8159617.1	*Aspergillus tamarii*	Y	PYI25926.1	*Aspergillus indologenus CBS 114.80*	Y	XP 031920939.1	*Aspergillus caelatus*	Y
KAE8159815.1	*Aspergillus tamarii*	Y	PYI26484.1	*Aspergillus indologenus CBS 114.80*	Y	XP 031921260.1	*Aspergillus caelatus*	Y
KAE8160123.1	*Aspergillus tamarii*	Y	PYI30925.1	*Aspergillus indologenus CBS 114.80*	Y	XP 031922710.1	*Aspergillus caelatus*	Y
KAE8162550.1	*Aspergillus tamarii*	Y	PYI31222.1	*Aspergillus indologenus CBS 114.80*	Y	XP 031922760.1	*Aspergillus caelatus*	Y
KAE8164405.1	*Aspergillus tamarii*	Y	PYI31632.1	*Aspergillus indologenus CBS 114.80*	Y	XP 031923025.1	*Aspergillus caelatus*	Y
KAE8166750.1	*Aspergillus tamarii*	Y	PYI33275.1	*Aspergillus indologenus CBS 114.80*	Y	XP 031923716.1	*Aspergillus caelatus*	Y
KAE8167283.1	*Aspergillus tamarii*	Y	PYI33855.1	*Aspergillus indologenus CBS 114.80*	Y	XP 031924390.1	*Aspergillus caelatus*	Y
KAE8168412.1	*Aspergillus tamarii*	Y	PYI35955.1	*Aspergillus indologenus CBS 114.80*	Y	XP 031926552.1	*Aspergillus caelatus*	Y
KAE8308620.1	*Aspergillus transmontanensis*	Y	PYI36170.1	*Aspergillus indologenus CBS 114.80*	Y	XP 031927106.1	*Aspergillus caelatus*	Y
KAE8310456.1	*Aspergillus transmontanensis*	Y	QMW38285.1	*Aspergillus flavus*	Y	XP 031927796.1	*Aspergillus caelatus*	G
KAE8311495.1	*Aspergillus transmontanensis*	Y	QMW42505.1	*Aspergillus flavus*	Y	XP 031929533.1	*Aspergillus caelatus*	Y
KAE8314309.1	*Aspergillus transmontanensis*	Y	QMW43140.1	*Aspergillus flavus*	Y	XP 031932766.1	*Aspergillus caelatus*	Y
KAE8315910.1	*Aspergillus transmontanensis*	Y	QMW46681.1	*Aspergillus flavus*	Y	XP 031934162.1	*Aspergillus pseudonomiae*	Y
KAE8317658.1	*Aspergillus transmontanensis*	Y	QMW48303.1	*Aspergillus flavus*	Y	XP 031938078.1	*Aspergillus pseudonomiae*	Y
KAE8318583.1	*Aspergillus transmontanensis*	Y	QRD82136.1	*Aspergillus flavus*	Y	XP 031942699.1	*Aspergillus pseudonomiae*	Y
KAE8319782.1	*Aspergillus transmontanensis*	Y	QRD83427.1	*Aspergillus flavus*	Y	XP 031944048.1	*Aspergillus pseudonomiae*	Y
KAE8320008.1	*Aspergillus transmontanensis*	Y	RAQ49801.1	*Aspergillus flavus*	Y	XP 031944770.1	*Aspergillus pseudonomiae*	Y
KAE8320613.1	*Aspergillus sergii*	Y	RAQ51408.1	*Aspergillus flavus*	Y	XP 031945768.1	*Aspergillus pseudonomiae*	Y
KAE8322641.1	*Aspergillus sergii*	Y	RAQ51458.1	*Aspergillus flavus*	Y	XP 031946304.1	*Aspergillus pseudonomiae*	Y
KAE8323899.1	*Aspergillus sergii*	Y	RAQ60170.1	*Aspergillus flavus*	Y	XP 031946990.1	*Aspergillus pseudonomiae*	Y
KAE8324379.1	*Aspergillus sergii*	Y	RAQ60703.1	*Aspergillus flavus*	Y	XP 033411567.1	*Aspergillus lentulus*	Y

KAE8324546.1	*Aspergillus sergii*	Y	RDH14738.1	*Aspergillus niger ATCC 13496*	E	XP 033413104.1	*Aspergillus lentulus*	Y
KAE8328655.1	*Aspergillus sergii*	Y	RDK38195.1	*Aspergillus phoenicis ATCC 13157*	Y	XP 033415769.1	*Aspergillus lentulus*	Y
KAE8329722.1	*Aspergillus sergii*	Y	RDK38345.1	*Aspergillus phoenicis ATCC 13157*	Y	XP 033418504.1	*Aspergillus lentulus*	Y
KAE8330065.1	*Aspergillus sergii*	Y	RDK41204.1	*Aspergillus phoenicis ATCC 13157*	Y	XP 033418697.1	*Aspergillus lentulus*	Y
KAE8333672.1	*Aspergillus sergii*	Y	RDK42060.1	*Aspergillus phoenicis ATCC 13157*	Y	XP 033420507.1	*Aspergillus lentulus*	C
KAE8336789.1	*Aspergillus arachidicola*	Y	RDK42213.1	*Aspergillus phoenicis ATCC 13157*	Y	XP 033423662.1	*Aspergillus tanneri*	Y
KAE8339099.1	*Aspergillus arachidicola*	Y	RDK43391.1	*Aspergillus phoenicis ATCC 13157*	Y	XP 033424287.1	*Aspergillus tanneri*	Y
KAE8339558.1	*Aspergillus arachidicola*	Y	RDK43706.1	*Aspergillus phoenicis ATCC 13157*	Y	XP 033426238.1	*Aspergillus tanneri*	Y
KAE8343082.1	*Aspergillus arachidicola*	Y	RHZ49817.1	*Aspergillus turcosus*	Y	XP 033426760.1	*Aspergillus tanneri*	Y
KAE8343392.1	*Aspergillus arachidicola*	Y	RHZ53289.1	*Aspergillus turcosus*	Y	XP 033432210.1	*Aspergillus tanneri*	Y
KAE8345043.1	*Aspergillus arachidicola*	Y	RHZ53464.1	*Aspergillus turcosus*	Y	XP 035352076.1	*Aspergillus tubingensis*	Y
KAE8346865.1	*Aspergillus arachidicola*	Y	RHZ71381.1	*Aspergillus turcosus*	Y	XP 035354385.1	*Aspergillus tubingensis*	Y
KAE8347061.1	*Aspergillus arachidicola*	Y	RJE20186.1	*Aspergillus sclerotialis*	Y	XP 035354860.1	*Aspergillus tubingensis*	Y
KAE8349611.1	*Aspergillus coremiiformis*	Y	RJE23841.1	*Aspergillus sclerotialis*	Y	XP 035355640.1	*Aspergillus tubingensis*	Y
KAE8351818.1	*Aspergillus coremiiformis*	F	RJE25462.1	*Aspergillus sclerotialis*	Y	XP 035357240.1	*Aspergillus tubingensis*	Y
KAE8352052.1	*Aspergillus coremiiformis*	Y	RJE27135.1	*Aspergillus sclerotialis*	Y	XP 035358053.1	*Aspergillus tubingensis*	Y
KAE8354004.1	*Aspergillus coremiiformis*	Y	RJE27435.1	*Aspergillus sclerotialis*	Y	XP 035358445.1	*Aspergillus tubingensis*	Y
KAE8355889.1	*Aspergillus coremiiformis*	Y	RLM00088.1	*Aspergillus turcosus*	Y	XP 035358854.1	*Aspergillus tubingensis*	Y
KAE8373688.1	*Aspergillus bertholletiae*	Y	RMJ23114.1	*Aspergillus sp. HF37*	Y	XP 035359023.1	*Aspergillus tubingensis*	Y
KAE8373979.1	*Aspergillus bertholletiae*	Y	RMJ28529.1	*Aspergillus sp. HF37*	Y	XP 035359380.1	*Aspergillus tubingensis*	Y
KAE8374606.1	*Aspergillus bertholletiae*	Y	RMZ43082.1	*Aspergillus flavus*	Y	XP 035360383.1	*Aspergillus tubingensis*	Y
KAE8377277.1	*Aspergillus bertholletiae*	Y	THC88631.1	*Aspergillus tanneri*	Y	XP 040634541.1	*Aspergillus ruber CBS 135680*	Y
KAE8377286.1	*Aspergillus bertholletiae*	Y	THC98905.1	*Aspergillus tanneri*	Y	XP 040634574.1	*Aspergillus ruber CBS 135680*	Y
KAE8378628.1	*Aspergillus bertholletiae*	Y	TPR09452.1	*Aspergillus niger*	Y	XP 040634690.1	*Aspergillus ruber CBS 135680*	Y
KAE8378823.1	*Aspergillus bertholletiae*	Y	UDD55615.1	*Aspergillus flavus*	Y	XP 040636838.1	*Aspergillus ruber CBS 135680*	Y
KAE8378925.1	*Aspergillus bertholletiae*	Y	UDD63563.1	*Aspergillus flavus*	Y	XP 040639288.1	*Aspergillus ruber CBS 135680*	Y
KAE8380178.1	*Aspergillus bertholletiae*	Y	XP 001211883.1	*Aspergillus terreus NIH2624*	Y	XP 040639710.1	*Aspergillus ruber CBS 135680*	Y
KAE8381235.1	*Aspergillus bertholletiae*	Y	XP 001212002.1	*Aspergillus terreus NIH2624*	F	XP 040641187.1	*Aspergillus ruber CBS 135680*	Y
KAE8381802.1	*Aspergillus bertholletiae*	Y	XP 001215108.1	*Aspergillus terreus NIH2624*	Y	XP 040642436.1	*Aspergillus ruber CBS 135680*	Y
KAE8386187.1	*Aspergillus alliaceus*	Y	XP 001217203.1	*Aspergillus terreus NIH2624*	Y	XP 040662123.1	*Aspergillus versicolor CBS 583.65*	Y
KAE8388972.1	*Aspergillus alliaceus*	Y	XP 001217695.1	*Aspergillus terreus NIH2624*	Y	XP 040663352.1	*Aspergillus versicolor CBS 583.65*	Y
KAE8391207.1	*Aspergillus alliaceus*	Y	XP 001218397.1	*Aspergillus terreus NIH2624*	Y	XP 040664150.1	*Aspergillus versicolor CBS 583.65*	Y
KAE8392269.1	*Aspergillus alliaceus*	Y	XP 001218562.1	*Aspergillus terreus NIH2624*	Y	XP 040665371.1	*Aspergillus versicolor CBS 583.65*	Y
KAE8392816.1	*Aspergillus alliaceus*	Y	XP 001259238.1	*Aspergillus fischeri NRRL 181*	Y	XP 040668899.1	*Aspergillus versicolor CBS 583.65*	Y
KAE8394020.1	*Aspergillus alliaceus*	Y	XP 001260040.1	*Aspergillus fischeri NRRL 181*	Y	XP 040669638.1	*Aspergillus versicolor CBS 583.65*	Y
KAE8394494.1	*Aspergillus alliaceus*	Y	XP 001260063.1	*Aspergillus fischeri NRRL 181*	Y	XP 040670248.1	*Aspergillus versicolor CBS 583.65*	Y
KAE8394831.1	*Aspergillus alliaceus*	Y	XP 001261594.1	*Aspergillus fischeri NRRL 181*	Y	XP 040671313.1	*Aspergillus versicolor CBS 583.65*	Y
KAE8410885.1	*Aspergillus pseudocaelatus*	Y	XP 001266387.1	*Aspergillus fischeri NRRL 181*	Y	XP 040671641.1	*Aspergillus versicolor CBS 583.65*	Y
KAE8411374.1	*Aspergillus pseudocaelatus*	Y	XP 001272755.1	*Aspergillus clavatus NRRL 1*	Y	XP 040671991.1	*Aspergillus versicolor CBS 583.65*	Y
KAE8412943.1	*Aspergillus pseudocaelatus*	Y	XP 001272777.1	*Aspergillus clavatus NRRL 1*	Y	XP 040672929.1	*Aspergillus versicolor CBS 583.65*	Y
KAE8417497.1	*Aspergillus pseudocaelatus*	Y	XP 001273480.1	*Aspergillus clavatus NRRL 1*	Y	XP 040673657.1	*Aspergillus versicolor CBS 583.65*	Y
KAE8417615.1	*Aspergillus pseudocaelatus*	Y	XP 001274702.1	*Aspergillus clavatus NRRL 1*	Y	XP 040674028.1	*Aspergillus versicolor CBS 583.65*	Y
KAE8419060.1	*Aspergillus pseudocaelatus*	Y	XP 001276393.1	*Aspergillus clavatus NRRL 1*	Y	XP 040674361.1	*Aspergillus versicolor CBS 583.65*	Y
KAE8419865.1	*Aspergillus pseudocaelatus*	Y	XP 001389551.2	*Aspergillus niger CBS 513.88*	Y	XP 040683369.1	*Aspergillus wentii DTO 134E9*	Y
KAE8421229.1	*Aspergillus pseudocaelatus*	Y	XP 001389993.1	*Aspergillus niger CBS 513.88*	Y	XP 040684588.1	*Aspergillus wentii DTO 134E9*	Y
KAE8422016.1	*Aspergillus pseudocaelatus*	Y	XP 001390054.1	*Aspergillus niger CBS 513.88*	Y	XP 040685894.1	*Aspergillus wentii DTO 134E9*	Y
KAE8422227.1	*Aspergillus pseudocaelatus*	Y	XP 001391174.1	*Aspergillus niger CBS 513.88*	Y	XP 040686221.1	*Aspergillus wentii DTO 134E9*	Y
KAE8422277.1	*Aspergillus pseudocaelatus*	Y	XP 001391950.1	*Aspergillus niger CBS 513.88*	Y	XP 040686383.1	*Aspergillus wentii DTO 134E9*	Y
KAF4159503.1	*Aspergillus lentulus*	Y	XP 001393044.1	*Aspergillus niger CBS 513.88*	Y	XP 040689261.1	*Aspergillus wentii DTO 134E9*	Y
KAF4161510.1	*Aspergillus lentulus*	Y	XP 001394816.2	*Aspergillus niger CBS 513.88*	Y	XP 040689680.1	*Aspergillus wentii DTO 134E9*	Y
KAF4166679.1	*Aspergillus lentulus*	Y	XP 001397404.1	*Aspergillus niger CBS 513.88*	Y	XP 040689721.1	*Aspergillus wentii DTO 134E9*	Y
KAF4166976.1	*Aspergillus lentulus*	Y	XP 001399273.1	*Aspergillus niger CBS 513.88*	Y	XP 040689758.1	*Aspergillus wentii DTO 134E9*	Y
KAF4170163.1	*Aspergillus lentulus*	Y	XP 001401198.1	*Aspergillus niger CBS 513.88*	Y	XP 040689773.1	*Aspergillus wentii DTO 134E9*	Y
KAF4176619.1	*Aspergillus lentulus*	Y	XP 001727650.1	*Aspergillus oryzae RIB40*	Y	XP 040690647.1	*Aspergillus wentii DTO 134E9*	Y
KAF4183382.1	*Aspergillus lentulus*	Y	XP 001817541.1	*Aspergillus oryzae RIB40*	Y	XP 040691679.1	*Aspergillus wentii DTO 134E9*	Y
KAF4185275.1	*Aspergillus lentulus*	Y	XP 001817710.1	*Aspergillus oryzae RIB40*	Y	XP 040695615.1	*Aspergillus wentii DTO 134E9*	Y
KAF4185463.1	*Aspergillus lentulus*	Y	XP 001818449.1	*Aspergillus oryzae RIB40*	Y	XP 040696138.1	*Aspergillus sydowii CBS 593.65*	Y
KAF4186756.1	*Aspergillus lentulus*	Y	XP 001819432.1	*Aspergillus oryzae RIB40*	Y	XP 040696528.1	*Aspergillus sydowii CBS 593.65*	Y
KAF4208022.1	*Aspergillus lentulus*	Y	XP 015402090.1	*Aspergillus nomiae NRRL 13137*	Y	XP 040697943.1	*Aspergillus sydowii CBS 593.65*	Y
KAF4213261.1	*Aspergillus fumigatiaffinis*	Y	XP 015403903.1	*Aspergillus nomiae NRRL 13137*	Y	XP 040698815.1	*Aspergillus sydowii CBS 593.65*	Y
KAF4214235.1	*Aspergillus fumigatiaffinis*	Y	XP 015404024.1	*Aspergillus nomiae NRRL 13137*	Y	XP 040698847.1	*Aspergillus sydowii CBS 593.65*	Y
KAF4217351.1	*Aspergillus fumigatiaffinis*	Y	XP 015405249.1	*Aspergillus nomiae NRRL 13137*	Y	XP 040699197.1	*Aspergillus sydowii CBS 593.65*	Y
KAF4221443.1	*Aspergillus fumigatiaffinis*	Y	XP 015409281.1	*Aspergillus nomiae NRRL 13137*	Y	XP 040700295.1	*Aspergillus sydowii CBS 593.65*	Y
KAF4221735.1	*Aspergillus fumigatiaffinis*	Y	XP 015410234.1	*Aspergillus nomiae NRRL 13137*	Y	XP 040700983.1	*Aspergillus sydowii CBS 593.65*	Y
KAF4225521.1	*Aspergillus fumigatiaffinis*	Y	XP 015411263.1	*Aspergillus nomiae NRRL 13137*	Y	XP 040703433.1	*Aspergillus sydowii CBS 593.65*	Y
KAF4234664.1	*Aspergillus fumigatiaffinis*	Y	XP 015411747.1	*Aspergillus nomiae NRRL 13137*	Y	XP 040706056.1	*Aspergillus sydowii CBS 593.65*	Y
KAF4253213.1	*Aspergillus fumigatus*	Y	XP 020052312.1	*Aspergillus aculeatus ATCC 16872*	Y	XP 040707166.1	*Aspergillus sydowii CBS 593.65*	Y
KAF4253273.1	*Aspergillus fumigatus*	Y	XP 020052353.1	*Aspergillus aculeatus ATCC 16872*	Y	XP 040707780.1	*Aspergillus sydowii CBS 593.65*	Y
KAF4257036.1	*Aspergillus fumigatus*	Y	XP 020052766.1	*Aspergillus aculeatus ATCC 16872*	Y	XP 040708101.1	*Aspergillus sydowii CBS 593.65*	Y
KAF4257699.1	*Aspergillus fumigatus*	Y	XP 020056001.1	*Aspergillus aculeatus ATCC 16872*	Y	XP 040796266.1	*Aspergillus fijiensis CBS 313.89*	Y
KAF4276960.1	*Aspergillus fumigatus*	Y	XP 020057897.1	*Aspergillus aculeatus ATCC 16872*	Y	XP 040797278.1	*Aspergillus fijiensis CBS 313.89*	Y
KAF4283050.1	*Aspergillus fumigatus*	Y	XP 020057976.1	*Aspergillus aculeatus ATCC 16872*	Y	XP 040798354.1	*Aspergillus fijiensis CBS 313.89*	Y
KAF5858195.1	*Aspergillus burnettii*	Y	XP 020059377.1	*Aspergillus aculeatus ATCC 16872*	Y	XP 040799704.1	*Aspergillus fijiensis CBS 313.89*	Y
KAF5858392.1	*Aspergillus burnettii*	Y	XP 020061266.1	*Aspergillus aculeatus ATCC 16872*	Y	XP 040799789.1	*Aspergillus fijiensis CBS 313.89*	Y
KAF5861629.1	*Aspergillus burnettii*	Y	XP 022382893.1	*Aspergillus bombycis*	Y	XP 040801049.1	*Aspergillus fijiensis CBS 313.89*	Y
KAF5866063.1	*Aspergillus burnettii*	Y	XP 022383284.1	*Aspergillus bombycis*	Y	XP 040801511.1	*Aspergillus fijiensis CBS 313.89*	W
KAF7114952.1	*Aspergillus hiratsukae*	Y	XP 022384212.1	*Aspergillus bombycis*	Y	XP 040804497.1	*Aspergillus fijiensis CBS 313.89*	Y
KAF7114981.1	*Aspergillus hiratsukae*	F	XP 022384627.1	*Aspergillus bombycis*	Y	XP 040804881.1	*Aspergillus fijiensis CBS 313.89*	Y
KAF7115296.1	*Aspergillus hiratsukae*	Y	XP 022385618.1	*Aspergillus bombycis*	Y	XP 040805201.1	*Aspergillus fijiensis CBS 313.89*	Y
KAF7122204.1	*Aspergillus hiratsukae*	Y	XP 022390927.1	*Aspergillus bombycis*	W	XP 040805262.1	*Aspergillus fijiensis CBS 313.89*	Y
KAF7134172.1	*Aspergillus hiratsukae*	Y	XP 022391105.1	*Aspergillus bombycis*	Y	XP 041140464.1	*Aspergillus flavus NRRL3357*	Y
KAF7157324.1	*Aspergillus felis*	Y	XP 022391156.1	*Aspergillus bombycis*	Y	XP 041141207.1	*Aspergillus flavus NRRL3357*	Y
KAF7158383.1	*Aspergillus hiratsukae*	Y	XP 022391946.1	*Aspergillus bombycis*	Y	XP 041142719.1	*Aspergillus flavus NRRL3357*	Y
KAF7159369.1	*Aspergillus felis*	Y	XP 022392652.1	*Aspergillus bombycis*	Y	XP 041145437.1	*Aspergillus flavus NRRL3357*	Y
KAF7161731.1	*Aspergillus felis*	Y	XP 022394273.1	*Aspergillus bombycis*	Y	XP 041146091.1	*Aspergillus flavus NRRL3357*	Y
KAF7167500.1	*Aspergillus felis*	Y	XP 022394370.1	*Aspergillus bombycis*	Y	XP 041147032.1	*Aspergillus flavus NRRL3357*	Y
KAF7169902.1	*Aspergillus hiratsukae*	Y	XP 022395312.1	*Aspergillus glaucus CBS 516.65*	Y	XP 041149614.1	*Aspergillus flavus NRRL3357*	Y
KAF7171195.1	*Aspergillus hiratsukae*	Y	XP 022395525.1	*Aspergillus glaucus CBS 516.65*	Y	XP 041151252.1	*Aspergillus flavus NRRL3357*	Y
KAF7173692.1	*Aspergillus felis*	Y	XP 022395808.1	*Aspergillus glaucus CBS 516.65*	Y	XP 041536825.1	*Aspergillus luchuensis*	Y
KAF7586492.1	*Aspergillus hancockii*	Y	XP 022396041.1	*Aspergillus glaucus CBS 516.65*	Y	XP 041537643.1	*Aspergillus luchuensis*	Y
KAF7587843.1	*Aspergillus hancockii*	Y	XP 022396887.1	*Aspergillus glaucus CBS 516.65*	Y	XP 041538130.1	*Aspergillus luchuensis*	Y
KAF7588053.1	*Aspergillus hancockii*	F	XP 022397517.1	*Aspergillus glaucus CBS 516.65*	Y	XP 041538765.1	*Aspergillus luchuensis*	Y

KAF7588100.1	*Aspergillus hancockii*	Y	XP 022398265.1	*Aspergillus glaucus CBS 516.65*	Y	XP 041540326.1	*Aspergillus luchuensis*	Y
KAF7591663.1	*Aspergillus hancockii*	Y	XP 022398441.1	*Aspergillus glaucus CBS 516.65*	Y	XP 041543281.1	*Aspergillus luchuensis*	Y
KAF7592211.1	*Aspergillus hancockii*	Y	XP 022399159.1	*Aspergillus glaucus CBS 516.65*	-	XP 041545155.1	*Aspergillus luchuensis*	Y
KAF7592323.1	*Aspergillus hancockii*	Y	XP 022399568.1	*Aspergillus glaucus CBS 516.65*	Y	XP 041546949.1	*Aspergillus luchuensis*	Y
KAF7593911.1	*Aspergillus hancockii*	Y	XP 022401172.1	*Aspergillus glaucus CBS 516.65*	Y	XP 041548678.1	*Aspergillus luchuensis*	Y
KAF7594563.1	*Aspergillus hancockii*	Y	XP 022402646.1	*Aspergillus glaucus CBS 516.65*	Y	XP 041551140.1	*Aspergillus puulaauensis*	Y
KAF7594783.1	*Aspergillus hancockii*	Y	XP 022404916.1	*Aspergillus glaucus CBS 516.65*	Y	XP 041552957.1	*Aspergillus puulaauensis*	Y
KAF7594846.1	*Aspergillus hancockii*	Y	XP 022405452.1	*Aspergillus glaucus CBS 516.65*	Y	XP 041554022.1	*Aspergillus puulaauensis*	Y
KAF7594902.1	*Aspergillus hancockii*	Y	XP 023089550.1	*Aspergillus oryzae RIB40*	Y	XP 041555618.1	*Aspergillus puulaauensis*	Y
KAF7595948.1	*Aspergillus hancockii*	Y	XP 023089884.1	*Aspergillus oryzae RIB40*	Y	XP 041557136.1	*Aspergillus puulaauensis*	Y
KAF7619826.1	*Aspergillus flavus NRRL3357*	Y	XP 023090867.1	*Aspergillus oryzae RIB40*	Y	XP 041557903.1	*Aspergillus puulaauensis*	Y
KAF9883322.1	*Aspergillus nanangensis*	Y	XP 023092327.1	*Aspergillus oryzae RIB40*	Y	XP 041559885.1	*Aspergillus puulaauensis*	Y
KAF9885474.1	*Aspergillus nanangensis*	Y	XP 024669659.1	*Aspergillus candidus*	Y	XP 041560762.1	*Aspergillus puulaauensis*	Y
KAF9885688.1	*Aspergillus nanangensis*	Y	XP 024669972.1	*Aspergillus candidus*	Y	XP 041561746.1	*Aspergillus puulaauensis*	Y
KAF9886163.1	*Aspergillus nanangensis*	Y	XP 024679167.1	*Aspergillus novofumigatus IBT 16806*	Y	XP 041562191.1	*Aspergillus puulaauensis*	Y
KAF9886749.1	*Aspergillus nanangensis*	Y	XP 024685019.1	*Aspergillus novofumigatus IBT 16806*	Y	XP 041562947.1	*Aspergillus puulaauensis*	Y
KAF9888670.1	*Aspergillus nanangensis*	Y	XP 024686348.1	*Aspergillus novofumigatus IBT 16806*	Y	XP 043122320.1	*Aspergillus viridinutans*	Y
KAF9889016.1	*Aspergillus nanangensis*	Y	XP 024686927.1	*Aspergillus novofumigatus IBT 16806*	Y	XP 043122343.1	*Aspergillus viridinutans*	Y
KAF9890364.1	*Aspergillus nanangensis*	Y	XP 024686955.1	*Aspergillus novofumigatus IBT 16806*	Y	XP 043122850.1	*Aspergillus viridinutans*	Y
KAF9890558.1	*Aspergillus nanangensis*	Y	XP 024688622.1	*Aspergillus campestris IBT 28561*	Y	XP 043127029.1	*Aspergillus viridinutans*	Y
KAF9895094.1	*Aspergillus nanangensis*	Y	XP 024694543.1	*Aspergillus campestris IBT 28561*	Y	XP 043128331.1	*Aspergillus viridinutans*	Y
KAF9895264.1	*Aspergillus nanangensis*	Y	XP 024698654.1	*Aspergillus steynii IBT 23096*	Y	XP 043134473.1	*Aspergillus chevalieri*	Y
KAG2026315.1	*Aspergillus fischeri*	Y	XP 024698678.1	*Aspergillus steynii IBT 23096*	Y	XP 043138742.1	*Aspergillus chevalieri*	Y
KAG2411631.1	*Aspergillus terreus*	Y	XP 024699132.1	*Aspergillus steynii IBT 23096*	F	XP 043139245.1	*Aspergillus chevalieri*	Y
KAG2414700.1	*Aspergillus terreus*	Y	XP 024699442.1	*Aspergillus steynii IBT 23096*	Y	XP 043139661.1	*Aspergillus chevalieri*	Y
KAG2417102.1	*Aspergillus terreus*	Y	XP 024700821.1	*Aspergillus steynii IBT 23096*	Y	XP 043140709.1	*Aspergillus chevalieri*	Y
KAG2417479.1	*Aspergillus terreus*	Y	XP 024704013.1	*Aspergillus steynii IBT 23096*	Y	XP 043141095.1	*Aspergillus chevalieri*	Y
KAG2418561.1	*Aspergillus terreus*	F	XP 024704193.1	*Aspergillus steynii IBT 23096*	Y	XP 043141966.1	*Aspergillus udagawae*	Y
KAG2421291.1	*Aspergillus terreus*	Y	XP 024705403.1	*Aspergillus steynii IBT 23096*	Y	XP 043141993.1	*Aspergillus udagawae*	Y
KAH1278553.1	*Aspergillus fumigatus*	Y	XP 024705961.1	*Aspergillus steynii IBT 23096*	Y	XP 043143597.1	*Aspergillus udagawae*	Y
KAH1306293.1	*Aspergillus fumigatus*	Y	XP 024708747.1	*Aspergillus steynii IBT 23096*	Y	XP 043145340.1	*Aspergillus udagawae*	Y
KAH1330903.1	*Aspergillus fumigatus*	Y	XP 024708886.1	*Aspergillus steynii IBT 23096*	Y	XP 043146304.1	*Aspergillus udagawae*	Y
KAH1338146.1	*Aspergillus fumigatus*	Y	XP 024709209.1	*Aspergillus steynii IBT 23096*	Y	XP 043147505.1	*Aspergillus udagawae*	Y
KAH1339640.1	*Aspergillus fumigatus*	Y	XP 024709236.1	*Aspergillus steynii IBT 23096*	W	XP 043150937.1	*Aspergillus udagawae*	Y
KAH1404135.1	*Aspergillus fumigatus*	Y	XP 024710872.1	*Aspergillus steynii IBT 23096*	Y	XP 043153448.1	*Aspergillus pseudoviridinutans*	Y
KAH1428112.1	*Aspergillus fumigatus*	Y	XP 025382123.1	*Aspergillus eucalypticola CBS 122712*	Y	XP 043153471.1	*Aspergillus pseudoviridinutans*	Y
KAH1434756.1	*Aspergillus fumigatus*	Y	XP 025382284.1	*Aspergillus eucalypticola CBS 122712*	Y	XP 043155883.1	*Aspergillus pseudoviridinutans*	Y
KAH1444201.1	*Aspergillus fumigatus*	Y	XP 025384666.1	*Aspergillus eucalypticola CBS 122712*	Y	XP 043157107.1	*Aspergillus pseudoviridinutans*	Y
KAH1456261.1	*Aspergillus fumigatus*	Y	XP 025386052.1	*Aspergillus eucalypticola CBS 122712*	Y	XP 043157278.1	*Aspergillus pseudoviridinutans*	Y
KAH1491166.1	*Aspergillus fumigatus*	Y	XP 025387721.1	*Aspergillus eucalypticola CBS 122712*	Y	XP 043158894.1	*Aspergillus pseudoviridinutans*	Y
KAH1494480.1	*Aspergillus fumigatus*	Y	XP 025387789.1	*Aspergillus eucalypticola CBS 122712*	Y	XP 043161970.1	*Aspergillus pseudoviridinutans*	Y
KAH1557488.1	*Aspergillus fumigatus*	Y	XP 025391051.1	*Aspergillus eucalypticola CBS 122712*	Y	XP 045938471.1	*Aspergillus melleus*	Y
KAH1566824.1	*Aspergillus fumigatus*	Y	XP 025392394.1	*Aspergillus eucalypticola CBS 122712*	Y	XP 045939841.1	*Aspergillus melleus*	Y
KAH1597662.1	*Aspergillus fumigatus*	Y	XP 025392909.1	*Aspergillus eucalypticola CBS 122712*	Y	XP 045940492.1	*Aspergillus melleus*	Y
KAH1636854.1	*Aspergillus fumigatus*	Y	XP 025394652.1	*Aspergillus heteromorphus CBS 117.55*	Y	XP 045943028.1	*Aspergillus melleus*	Y
KAH1679113.1	*Aspergillus fumigatus*	Y	XP 025395425.1	*Aspergillus heteromorphus CBS 117.55*	Y	XP 045945579.1	*Aspergillus melleus*	F
KAH1747039.1	*Aspergillus fumigatus*	Y	XP 025395691.1	*Aspergillus heteromorphus CBS 117.55*	Y	XP 045946014.1	*Aspergillus melleus*	Y
KAH1764307.1	*Aspergillus fumigatus*	Y	XP 025396011.1	*Aspergillus heteromorphus CBS 117.55*	Y	XP 045946206.1	*Aspergillus melleus*	Y
KAH1793438.1	*Aspergillus fumigatus*	Y	XP 025403647.1	*Aspergillus heteromorphus CBS 117.55*	Y	XP 045947359.1	*Aspergillus melleus*	Y
KAH1839399.1	*Aspergillus fumigatus*	Y	XP 025404056.1	*Aspergillus heteromorphus CBS 117.55*	Y	XP 045948277.1	*Aspergillus melleus*	Y
KAH1933200.1	*Aspergillus fumigatus*	Y	XP 025427279.1	*Aspergillus saccharolyticus JOP 1030-1*	Y	XP 045949134.1	*Aspergillus melleus*	Y
KAH1948437.1	*Aspergillus fumigatus*	Y	XP 025428967.1	*Aspergillus saccharolyticus JOP 1030-1*	Y	XP 045949563.1	*Aspergillus melleus*	Y
KAH2119130.1	*Aspergillus fumigatus*	Y	XP 025434634.1	*Aspergillus saccharolyticus JOP 1030-1*	Y	XP 050468568.1	*Aspergillus nidulans FGSC A4*	Y
KAH2141343.1	*Aspergillus fumigatus*	Y	XP 025434700.1	*Aspergillus saccharolyticus JOP 1030-1*	Y	XP 657934.1	*Aspergillus nidulans FGSC A4*	Y
KAH2236131.1	*Aspergillus fumigatus*	Y	XP 025435041.1	*Aspergillus saccharolyticus JOP 1030-1*	Y	XP 659486.1	*Aspergillus nidulans FGSC A4*	Y
KAH2342411.1	*Aspergillus fumigatus*	Y	XP 025435563.1	*Aspergillus saccharolyticus JOP 1030-1*	Y	XP 661749.1	*Aspergillus nidulans FGSC A4*	Y
KAH2376807.1	*Aspergillus fumigatus*	Y	XP 025440450.1	*Aspergillus brunneoviolaceus CBS 621.78*	Y	XP 662832.1	*Aspergillus nidulans FGSC A4*	Y
KAH2416158.1	*Aspergillus fumigatus*	Y	XP 025440544.1	*Aspergillus brunneoviolaceus CBS 621.78*	Y	XP 664357.1	*Aspergillus nidulans FGSC A4*	Y
KAH2456554.1	*Aspergillus fumigatus*	Y	XP 025441424.1	*Aspergillus brunneoviolaceus CBS 621.78*	Y	XP 682446.1	*Aspergillus nidulans FGSC A4*	Y
KAH2524711.1	*Aspergillus fumigatus*	Y	XP 025441506.1	*Aspergillus brunneoviolaceus CBS 621.78*	Y	XP 682614.1	*Aspergillus nidulans FGSC A4*	Y
KAH2524833.1	*Aspergillus fumigatus*	Y	XP 025444048.1	*Aspergillus brunneoviolaceus CBS 621.78*	Y	XP 748197.1	*Aspergillus fumigatus Af293*	Y
KAH2529039.1	*Aspergillus fumigatus*	Y	XP 025444292.1	*Aspergillus brunneoviolaceus CBS 621.78*	Y	XP 748868.1	*Aspergillus fumigatus Af293*	Y
KAH2657265.1	*Aspergillus fumigatus*	Y	XP 025445460.1	*Aspergillus brunneoviolaceus CBS 621.78*	W	XP 749538.1	*Aspergillus fumigatus Af293*	Y
KAH2675591.1	*Aspergillus fumigatus*	Y	XP 025445800.1	*Aspergillus brunneoviolaceus CBS 621.78*	Y	XP 749564.1	*Aspergillus fumigatus Af293*	Y
KAH2711053.1	*Aspergillus fumigatus*	Y	XP 025447680.1	*Aspergillus brunneoviolaceus CBS 621.78*	Y	XP 753224.1	*Aspergillus fumigatus Af293*	Y
KAH2904598.1	*Aspergillus fumigatus*	-	XP 025447737.1	*Aspergillus brunneoviolaceus CBS 621.78*	Y	XP 756133.1	*Aspergillus fumigatus Af293*	Y

Table S2. Bioinformatic analysis of the *sub* gene cluster from *P. subrubescens*.

Protein	Size (kDa)	Protein (% identity)	Organism	Accession	Associated Biosynthesis	Function
**SubA**	54.7	CnsB (42.89%)	*Penicillium expansum*	A0A0A2IDH4	Communesins	L-tryptophan decarboxylase
**SubB**	58.5	AflU (42.4%)	*Aspergillus parasiticus* SU-1	Q6UEH4	Aflatoxins	Cytochrome P450 monooxygenase
**SubC**	28.6	FabH (36.80%)	*Thermotoga maritima* MSB8 (bacteria)	Q9X248	N/A	3-oxoacyl-[acyl-carrier-protein] reductase
**SubD**	47.9	FtmMT (44.54%)	*Aspergillus fumigatus* Af293	Q4WAW6	Fumitremorgins	6-hydroxytryprostatin B O-methyltransferase
**SubE**	47.5	MalE (36.98%)	*Malbranchea aurantiaca*	L0E2P7	Malbrancheamides	Malbrancheamide prenyltransferase
**SubF**	41.2	EasA (55.62%)	*Aspergillus fumigatus* Af293	Q4WZ70	Ergot alkaloids	Chanoclavine-I aldehyde reductase
**SubG**	57.5	FlvC (33.08%)	*Aspergillus flavus* NRRL3357	B8NHD8	Flavunoidines	Cytochrome P450 monooxygenase
**SubH**	37.1	AdhA (35.28%)	*Synechocystis sp. PCC* 6803 Kazusa (bacteria)	P74721	N/A	Aldehyde reductase
**SubI**	33.0	tRNA-NMT (42.86%)	*Parasynechococcus marenigrum* (bacteria)	Q7U9H3	N/A	tRNA (guanine-N7-)-methyltransferase
**SubJ**	56.4	ClaJ (43.02%)	*Penicillium crustosum*	A0A481WPJ6	Clavatols	Cytochrome P450 monooxygenase
**SubK**	146.2	Hkm11 (35.18%)	*Aspergillus hancockii*	P0DUL9	Hancockiamides	Nonribosomal peptide synthetase

Table S3. gDNA sequences and predicted protein sequences.

Protein	gDNA (predicted orf underlined)	Predicted protein sequence
**SubA**	**ATG**ACCGTGACAAGCGAAGACCATCCACTGGACACTTTTCTGGGTCACAAACTCCTTGAGCAACATCACATTCGCGATGCTGATGAACAGCTGGTGCGCCGTCCCACGACTGAGGAGAT<br>GGCCCGACTTGAAGAGATGGCCATACCGGGAACACCTCAAGACCTGGAAAAAGTCATCGCACACACCCGAGAGATTCTCTCATACCGTATCGCCCTCGGCCATACTCGATTCTTCTCAT<br>GTATACCCTCTCCCGCCTCTCCAGTTTCTTGGTTTGGAGAGGCCTTGACAAATGCCTTCAACCCCTTTGCTGGCAATCTATCGGCCGGTCCAGGAATCTGTGCCATTGAAAGGGCGCTG<br>GTTAAGTGGATCGCAAATCAATTTGCGCTTCCACCTACTGCCGGAGGGCAATTCGTCTCGGGCGCTTCAATCGCCAACATGACTGCCATGACTGTTGCCCGGGATCATAAGCTCGCGGA<br>ACACACTCGCTCGCAAGGGGTCGTATATATATCAGAGCAGGCCCACTTTTGTGTGCGGAAAGCACTCCGCATCATTGGATTTTCAGACAGGCAGATCCAGAGCATTCCAGTCGATTCAA<br>ACTTTCGCATGGACGTTCGCATACTACAGCAAGCAATCACCTCAGACCTAGATGAGGGCCTACGTCCGTTTCTGATTGTGGCAACGTTTGGAACGACCAATACGGGTACCATTGACCCC<br>CTCTTGCCGATCATCGACATTGCAAAGTCGCACAATTTGTGGTTTCACGTCGATGCCGCATACGGAGGCGCTGTGGCCTTCTCCACGAAGCATCGTTACAAGATTGAAGGTCTTGGACA<br>GGTAGACAGTATTGCATGGGATGCCCACAAATGGATGTTCCAGACGCATGGGTGCGGGCTCGTCCTCTTTCGCGACGGGACTCATCCACTCAAAAGCTTCGCCGCTGGGGGCGAGTATG<br>TCCGAGACGTGAGCGTGGTGCGAGATCCCTCCAATTACGGAATTGAACTTACCCGGCCCGCTCGCCATATGAAGCTTTGGTTCACTTTACAAGTGCTGGGAACGGAAGCCATTGAGAAC<br>ATGATCAACTGGGGAATTGAGCTTGCCATGCACGCCGAAGATAAACTTCGATCATTGGATCACTGGGAAATTACCTCTCCTGCAAGTTTGGCCATACTCACTTTCCGCTACGCGCCTCC<br>CCATTTGGACGAAATTATGTTTGACAGGTTGAATGAGACCATTTCCAAAGAGATGATCGCCCGTAACGTTGCTGTCATGTTCACGACTATACTTGACGGCAAAGTATGTTTGCGAATGT<br>GCACAATCAACCCGAATACCACAATGAACGAGGTCACATACGTTATTCAGGTCGTGGAAGATGTTGCTGCCAGTTGCCTTCAGCAAAGCGCGCACTGTAGAGACGATTGCGCAGACAAG<br>CTGTCGACCTGCGGACCAGAAAGACGACAGCTTCCGCTACATTCT**TGA**TATAGATAGGACAACGCTATAATCTCAAATTTAATTGCCAGAAACTCTGGGAGTCTTCTAACTTGCTGGTC<br>TTCCGTCCCTCCTAAGCGAGCAGCTCGAAGACACGACACACGGCAACGCGCTATCTACTCAAGCTGTCTCCTGGTGAACTAGGTACCCGTCTATCGAAGGCAAGATGTTCTACTTCCTA<br>TGATTTAGTAAATTTTGGTCTCGGTCGATACTGGATATGAC	MTVTSEDHPLDTFLGHKLLEQHHIRDADEQLVRRPTTE<br>EMARLEEMAIPGTPQDLEKVIAHTREILSYRIALGHTR<br>FFSCIPSPASPVSWFGEALTNAFNPFAGNLSAGPGICA<br>IERALVKWIANQFALPPTAGGQFVSGASIANMTAMTVA<br>RDHKLAEHTRSQGVVYISEQAHFCVRKALRIIGFSDRQ<br>IQSIPVDSNFRMDVRILQQAITSDLDEGLRPFLIVATF<br>GTTNTGTIDPLLPIIDIAKSHNLWFHVDAAYGGAVAFS<br>TKHRYKIEGLGQVDSIAWDAHKWMFQTHGCGLVLFRDG<br>THPLKSFAAGGEYVRDVSVVRDPSNYGIELTRPARHMK<br>LWFTLQVLGTEAIENMINWGIELAMHAEDKLRSLDHWE<br>ITSPASLAILTFRYAPPHLDEIMFDRLNETISKEMIAR<br>NVAVMFTTILDGKVCLRMCTINPNTTMNEVTYVIQVVE<br>DVAASCLQQSAHCRDDCADKLSTCGPERRQLPLHS*
**SubB**	**ATG**ATAAATCTACTAGCTGTTACGTTTCTTGTGTGGGTTGGGGTCAAGTTTATATATCGCCTCCACTGGCATCCCCTGTGCCACATACCTGGACCAAAATGCGCCGCAATCAGTCATCT<br>TTATGAATTCTATTACGATGTCGTCAAAGGCGGAACTTACCTGTTTGAGGTTCAGAAGATGCATCGGAAATATGGTGAGCCATCACGGTGTTTTAGACCTGAAATCATCACCCAAGCTA<br>ATGTCGGTCGCTTTATGACAGGCCCTATAGTGCGTATCAACCCACGAGAAGTTCATATCGCGGATCCAGATTTCTATGACGATATATACTCGTCGGCAGCGAGAACAAGATGGAAAGAC<br>CCGAAGCAGGTCCCCATGATCGGCGCGCCATTTTCCATGGGTGCAACATGGCATCACGACCATCATAAATTTCGCCGTGGCCTTATCAAGAATCACTTTTCACGCCAGTCCATCAACAG<br>GATTTCCCCGAAAATCCGTCAGAAAGTGGATAAATTTTGTGATCGTCTCGAAGAAGCCTCGAAAAGTGCTCGTCCGGTCAACATGAGCGACGCCTTTGCCGCTTTGACCTCCGATATCA<br>TCGCTGGATATGCGTTCGACCTAGAGCTCGAATTCCTTAGTGATCCTCAGTTCAAGAACGATATCTACCGATCTGCACTGGAACTAGACATGCTGTGCCATGTTCTCAAGTTCTTTCCA<br>TTCATTTTGAAGCCTGTACGATGGCTTCCCTCCCAGCTGACTATCTTCTTGGGGCTACAAAACAAGTCTTTGCGCTCGCTCCAAGACATAATCGATAAGCAGATAATTTTGAAATCTTT<br>TCAAGCTGATCTTTGTGAGAAAGACGAGAACCGCACGACTGAGAACATTTTTACCTCACTCTGCAATCCAAGAGTCCCAGCAGAGGAGCGCACGCTTGACAGGCTGCGAGATGAAAGTT<br>TGGTGCTTCTCATTGCTGGCGCTGAAGCCACCGCTCGAGCTCTGGCTGTCTTCGCTTATTATGCAGCAAGTGGTTCTATCTGCCTCAATAGGCTCCGGAAAGAGCTCCGAGAAGTCTTA<br>CCAGATGCAAAATCGCATGCATCCTGGCCCCAGCTGGAGCATCTCCCCTATCTGGTATGTTGGCCGCTGATCTCCCTCTTCTGGGGCTGAAGGCATGGCCTCGATTGACATTTGAATAG<br>AAAGGGGTCGTGAACGAATCACTTCGACTCTCTTTCGGTGCCGTGGGTAGACTATCTCGCATGGCGCCAACGGAAGACCTGAATTACAAAGGCATCAAAATTCCGAAGAAGGTAACCGA<br>TAATAAAACTCTGCATCATCATCTTGATTAATTTTTGGGCTAATCTGACATTCTATCAGACTCCAATGAGTTCGTCTGCATATTTGATTCATATGAACCCCGACGTGTTCCCAGAACCA<br>GAGCGATTTTCACCCGACCGATGGCTCAAAGCTGCCGCGAGTGGGATCAATTTAGAACGCTACTTGGTGGCCTTCTCTCGAGGAACCAGGCAATGTATCGGAATCAAGTAAGCATCCGG<br>GGTCTGCCCGAGCATTATCTTGTTGAGAAATACAGGTTGACGTCTAATGACACCATGGTAGCTTAGCTCAATGCCAGCTTCATTTCACCATTGCGGCAGTCGCACAGCGATTTGATTTC<br>GATCTTTTTGACACCAAGTTGGAAGATATCCAAGTGACCAGAGATTATATCACTGCCTTCCCTCAGGATGGCCTCTTTGATGTGAAGTGTACGATTCGAAGCAGTACAGTGGAATATGT<br>GCGAGAGGTATAT**TGA**CAACGTACTTTCGACTCCTAAACCTGCACTGCTATGAGTACGCAAGTTTGATTTTACGTCTATTCCAGTATAATTTACTCAGCCACGACCGGGCTATTGGTAC<br>TGTACATGTGTCTTTGCACTGGAGAATTGCTTTGGGCTGTGGTTTTATGGTATTGGGAATATCAGCTTCGGGTGAATCTGGGTTATTAGAATGTGTTTTAGTAAACATCCTATTTGGGA<br>AACAGAATAGTGCTACCGAATTCTAAAGAAGAGTAAGGGTTCACGTAGAGCAGATGGTGACCCGATCAGGAGAGGTGATGAGCTCGACCAAGCTTCGGGGAATCGGTCTGAGGGGACTG<br>GCGAAAAGAGCGACGAGGGGCATCCATGGAGGGATGGCGGGTAGTAAGCGTCCAAGAATGAAAGCTGCTTTCTGCAATCAATGCTACGAACATGTCTTATCTCAGCCATCGTCCAGCAA<br>AGCTTTTGTGGGTGCATCTTGAGGCAGTATTAAAGTTGGGTATGGGAATGAACCACAGGGAATTTCGTTTGGTTTTTTCAGGTCAGGCGATAGACTTCACTGCAGCATGCACTACTTGA<br>AATC	MINLLAVTFLVWVGVKFIYRLHWHPLCHIPGPKCAAIS<br>HLYEFYYDVVKGGTYLFEVQKMHRKYGPIVRINPREVH<br>IADPDFYDDIYSSAARTRWKDPKQVPMIGAPFSMGATW<br>HHDHHKFRRGLIKNHFSRQSINRISPKIRQKVDKFCDR<br>LEEASKSARPVNMSDAFAALTSDIIAGYAFDLELEFLS<br>DPQFKNDIYRSALELDMLCHVLKFFPFILKPVRWLPSQ<br>LTIFLGLQNKSLRSLQDIIDKQIILKSFQADLCEKDEN<br>RTTENIFTSLCNPRVPAEERTLDRLRDESLVLLIAGAE<br>ATARALAVFAYYAASGSICLNRLRKELREVLPDAKSHA<br>SWPQLEHLPYLKGVVNESLRLSFGAVGRLSRMAPTEDL<br>NYKGIKIPKKTPMSSSAYLIHMNPDVFPEPERFSPDRW<br>LKAAASGINLERYLVAFSRGTRQCIGINLAQCQLHFTI<br>AAVAQRFDFDLFDTKLEDIQVTRDYITAFPQDGLFDVK<br>CTIRSSTVEYVREVY*
**SubC**	**ATG**GGATCCGTCAGCAAAGCATACATTGTCACTGGGGGAAGCTCAGGAATCGGCCTCGCTGTTGTCCAGGCCCTTCTCGCCAAAGATGCTGTTGTTCATGTTATCGATATAGCTCCAAA<br>TATTCCCAAACCTCTTCTGGAAGCCGAGCAGAGCGGTAGAGTCTACGTTCACCAAGGCATCGACGTTAGCTCGCGCGCACAAGTTGACAAGACTTTCAAAGCAATCCTCGATCGCTCCC<br>CCCGGATTCACGGCCTCGTGAACAGCGCTGGAATTATTTCAGAGAGCCTCGACGGTTTGAATCTCCCGGAATCTGATGAATCGTTTCGGCGCGTGATGGAGGTCAATCTTTTCGGAACC<br>TGGATTGCAGGGACCGCGTATCTGAACCACATCTTGGATTGCAATCCAAGCCTGCGCAGTTCACCCGACGAAATGGAAGTGAGAGAAGGACTTGGGAGCATCGTGAATCTGGCTTCTGA<br>ATCGATCCTCAGAACAGATCCAGGAATGGCTTCGTATAATACGAGCAAACACGCCGTGGTCGGCTTGACGAAGTCGTGGGCGAAAGACTTTGTCAGGCGCGGAGTGCGTGTCAATTGTG<br>TTGCTCCTGGGATGACAGACACTCCAATGATTCAAAATGAGAGTATTCCTCCGGAGATGGTGGAAGAGTTCATCAATACGGTGCCCTTGAAGCGGCCTGCTAGGCCGGAAGAGGTTGCT<br>CAGCTCATCGTCTTTCTGCTGGAGCCGACGTCGTCTTACATGACCGGACAGACTATCCCAATCGAGGGTGGTATTACCATT**TAG**ATAAATACCTGGAAGGTGCCTCTTGAGGATCTGAG<br>AAGTGACTCATCTATATAGACTATTAGCAGGCCAAATGCATTCACATCAATACAAACAGTGGCACTGTCACGTCCGGCATAGCTGTCCGTTCCAGCTACTCCTTAACCTACAAGACACC<br>TGTGAAATTACCTGGCTCAAAACAACAGTTAAAGTATTCCAGGTAG	MGSVSKAYIVTGGSSGIGLAVVQALLAKDAVVHVIDIA<br>PNIPKPLLEAEQSGRVYVHQGIDVSSRAQVDKTFKAIL<br>DRSPRIHGLVNSAGIISESLDGLNLPESDESFRRVMEV<br>NLFGTWIAGTAYLNHILDCNPSLRSSPDEMEVREGLGS<br>IVNLASESILRTDPGMASYNTSKHAVVGLTKSWAKDFV<br>RRGVRVNCVAPGMTDTPMIQNESIPPEMVEEFINTVPL<br>KRPARPEEVAQLIVFLLEPTSSYMTGQTIPIEGGITI*
**SubD**	**ATG**AGAGGTGTGCGAGAAACCCCCGTGCGAACACTGCGTGAAAGTCTGAAGCATTTGACTCGCGCAGTCCATGTGTTCGCCGCGGAACTAGATCAGGCTGGTTACAGCGACTACAGCTT<br>CGCACCAGACAATTCCCCTTATGATCTGTCCTTTCTTAGTTCTCGGGGCCTCGCCTCTCGTGGAGAGATAATCACTGCCGCGGAGACCATTATCAGACTGGCCAAAGGCCCACAGGAAG<br>CTCTTGCGGTGTTATCTGAAAAGGTACGCCCAATTGAATGACCAGCAGTTGCCAACGGAACTGACAAGTTGAAATCAGAGTGTCGAGCATGGGACTCTCCAAGCCTTGATTCAAATGGG<br>TATCCCGCAGAAGGTGCCTCTCGATGGCTCTGTTACATACAAGGAGCTCGCTGGTCCGGCGCAAATTGCACCCGACTTGCTGCAACGACTCGTTCGCCTAGCCGCGATGGGTGGGTTCC<br>TTTTAGAGGATGCGACAGGCAAAGTTCGACACACTGCCATGTCCGCTGTGTTCCATCTAGATCGAGACGTTGCAGACATGACGCATTTCATGTGCGACATTGACATGCGCGCGGGGACC<br>TATTTCTATGACTCGATTCGGCTTGACTCAAGCGGCAAGACAGCATCGCAAGGTCCAGCAGCATTAGCATTGCATGCTAGAGACTATAATAACGAGACGCGACGAACAATCTGGGAAGT<br>GCTGGAGAATGACCCGGTACAGAGTGCTCGATTTCATTCGAGCATGACTGCTTTACTTGCTTCTCCTTCTCACTCTCTCAAGCATATCGTAGGTGCCATGGACTGGAGTCAGTTCCCCA<br>CCGTGGTCGACGTACGTTATGAATCCACCTCCAATCAGTCATTTACTTCCTCGTCCTCACTGGAATTCTCTATATATATCAGCTGATACGTGCACAGGTTGGCGGTTCATTGGGTCAGA<br>TCGCCCTTGCAATCGTGGAGAAAAACCCCAACATCACCATCATTGTGCAGGACCTTCCCGACGTCATCAAGCAAGCGAAAGCTTCGCTACCATCTGCACCATGGGTCAGTCAGATCACC<br>TTCGAGCCGCATGATTTCTTTACAATCCAGCAGACCAGAGCGCCTGCCTATCTCTTGAGGCAAATACTCCATGACTGGCCGAATGAAGATGCTCAGCGTATTGTGAAGAATTTATTGCC<br>AGCCTTTGAAACTGGAACTCGCTTACTCGTCGCGGATATCGTCCTTCCCCAAAGTGGATCTATTTCACCGCATACGGAGAAGCTACTCCGCATCTATGATGTCTCGATGTTTTCCATTT<br>TTTCTAGCACCGAACGCACACTCGAACAGCTGAGGAACCTTGTTGAGGGATGTGATCCGCGGCTGCAGTTTGAGGGTGCTCACTATCCCGAAGGTAGCGCACTGAGCCTCGTGTCTTGG<br>ATCTATAATTCTGAG**TGA**CAAGAGAACGAAATTGTGGAGTGAAAATGATGGTGTGACCACGCCGGCGTGAAGGAAGGAAGCAGGGGAATGAGATGGTGGTGGGG	MRGVRETPVRTLRESLKHLTRAVHVFAAELDQAGYSDY<br>SFAPDNSPYDLSFLSSRGLASRGEIITAAETIIRLAKG<br>PQEALAVLSEKSVEHGTLQALIQMGIPQKVPLDGSVTY<br>KELAGPAQIAPDLLQRLVRLAAMGGFLLEDATGKVRHT<br>AMSAVFHLDRDVADMTHFMCDIDMRAGTYFYDSIRLDS<br>SGKTASQGPAALALHARDYNNETRRTIWEVLENDPVQS<br>ARFHSSMTALLASPSHSLKHIVGAMDWSQFPTVVDVGG<br>SLGQIALAIVEKNPNITIIVQDLPDVIKQAKASLPSAP<br>WVSQITFEPHDFFTIQQTRAPAYLLRQILHDWPNEDAQ<br>RIVKNLLPAFETGTRLLVADIVLPQSGSISPHTEKLLR<br>IYDVSMFSIFSSTERTLEQLRNLVEGCDPRLQFEGAHY<br>PEGSALSLVSWIYNSE*
**SubE**	**ATG**GCGATCGGAAATCAAGCGATTGCGGTGCCAAAGCGAGTGGCAACTACCTTTGACACGCTCTCTCTGAGTCTTGACTTCCCAAACGAAGACCAAGCACTCTGGTGGAAAGACACTGC<br>TCCTTTGCTTGAGAGACTGCTTTCGGCCACCGGATATGACGTTCATTTGCAATACCTGCATTTGATCTTTCACTATCGTTATGTTCTTGCAGCGCTCGGACCGTATCCGAAAGCAAAAT<br>CAGACAGCATTGTCCCAGGCGGCTGCCCATATGAGCTCAGTGTCAACTTCCAGCAAGGCAAGACTGTTGTTCGATTCGACTTTGCGCCAACGACTGCAATTGGACAGACTGGTCGAAGC<br>CATGATCAATATTGCCCCTGGCCAACGGTCAACAAATTACTGGCAGAGCTGTCGAATGATGGCCGCGAGATCGACACCAGGCTGTACAATCATTTCATCAATGCGCTCGCTGTGACAAA<br>GAAGGATCTTTCGGCCCTCCAGAAGTGTCCGACCATGCATTTGAGTACGCAATCTATTCTTTCTTGGGACTTGAAGAGCTGCTTCAATGATTTGAAGATGTACTTCTTTCCGCTGGTGA<br>AGGCAGACGCAGTCAGGGCTCCTTCTGGTCAACTTGTATTAAACGCTGTTCGCGGGGCAGATGGTGGACGATTCATCTCTCCAGCATTCAACTTGGTAGAGACTTCCCTGGGAGAAATG<br>GGGCTTCTTTCCAATGTGTGCATACTTGGATGGGATTGTGTGCCCGACGAGGGCCGGGTGAAGATATATCTTGCCGAGCCGAATGTGACTAAAGCAAATGTTCACCATCTATGGACCCT<br>CGGAGGACGGCTCAACAGTTCTACCATTAACAAAGGGTGGGAACTGCTTTCAAGGTTATGGGATTATTTAGGTATACCGGAAGGAGTGCGGTCCACTACTGAACTCGGCCAGCCCGAGC<br>AAGAGGATGGTCGAAGTGGATTGACCTTTTCCTACGAATTGCGACCCGGAAGCCTGGAACCCGAGCCAAAGATCTATATTCCACTCGTTGGGGAAAATGATATGGCTGTAGTCAGAAGC<br>CTGGCTCAGTTCACCTCGGACCTTGGCTGGACAGATCTTGATAGCTACCCGTCCAAGCTGGCAAATGTCTTGTACGTGTACTCACTCTTGAATTCGGAACTGACAAGGTACTGACCAAC<br>CATCCGTTTCAGCTCTCATGTTGACATGAACATTTCCACTCGTCTTATCACCTCCCTTTCTTTTGCCTACACAGAGGAAAAAGGGCCGTACATGTCGGCCTATTACTTTTCATCGGGGC<br>GATACCCCTGGGAAGGTTCTCGACTGTTG**TAG**TTGCAGCAGTGATCTAATTGTGAATACAAGCTAAGAGATGGAAAGCGAAGAATGGTCCTCAATGTCATGTTTCAATTTAGAATAAAC<br>TCCGATCGATATTTCTGTAATCCTGCGTACTTTCTACGGAGCAGACATCCATTGCACTCGACATGGCACACCGCAGGTGCCCAAATTTCTTCTTTAATTAAATCATTCCTCGCAGAATG<br>ACGTACGATTC	MAIGNQAIAVPKRVATTFDTLSLSLDFPNEDQALWWKD<br>TAPLLERLLSATGYDVHLQYLHLIFHYRYVLAALGPYP<br>KAKSDSIVPGGCPYELSVNFQQGKTVVRFDFAPTTAIG<br>QTGRSHDQYCPWPTVNKLLAELSNDGREIDTRLYNHFI<br>NALAVTKKDLSALQKCPTMHLSTQSILSWDLKSCFNDL<br>KMYFFPLVKADAVRAPSGQLVLNAVRGADGGRFISPAF<br>NLVETSLGEMGLLSNVCILGWDCVPDEGRVKIYLAEPN<br>VTKANVHHLWTLGGRLNSSTINKGWELLSRLWDYLGIP<br>EGVRSTTELGQPEQEDGRSGLTFSYELRPGSLEPEPKI<br>YIPLVGENDMAVVRSLAQFTSDLGWTDLDSYPSKLANV<br>FSHVDMNISTRLITSLSFAYTEEKGPYMSAYYFSSGRY<br>PWEGSRLL*
**SubF**	**ATG**ACGAAGGATTCGAAGCTTTTCAAAAGTATTCGCTTGGGGCCTTGCAACCTCCACCACCGGGTGGTGATGGCACCGCTCACCCGGCTTCGTGCCGATGATCTTTACATCCAGCTCCC<br>GTTTGTCAAGGACTACTATGGGCAGCGTGCCTCGGTACCCGGGACCTTGCTCATCGCAGAGGCAACCGCGATCAGTCCCCAGGCTGTCGGGTTTTCGAACACTCCGGGTATCTGGAGTA<br>CGGATCAAATACATGCATGGACAGAGGTGACCGAATGCGTCCACCAAAAAGGCTCCTATATCTTTTTACAGCTGTGGGCATGTGGCCGTGCTGCCGAACAGGAGGCTCTTGACAAGGAC<br>GGGATTCGATTCGTGTCAAGCAGTGCGACTGCTGTTGACCCTTATCAGCGATGCCCTCATCCGCTTACTGAGGGAGAGATTCAAGACTACATTACCGACTTTGCAACCGCAGCCAGGAA<br>TGCCATTACCGCAGGGTTTGATGGCGTGGAAATTCACGGCGCACACGGCTTCCTGATCGATCAATTCACTCAGGAGATCTGTAACAAGCGAACGGATCAATGGGGTGGAGCATCGAAA<br>ACAGATCCCGCTTTGCCCTTGAGGTGACCAAAGCCGTTGTGGACGCTGTCGGCGCAGACCGAGTTGGAATTAAACTCAGTCCCTGGGCCGAGGAGTTTGGCATGAAGATGACGCGTGAT<br>ATAATACCGCAATTTGAGTTTCTCATCAAAGCTCTTGCGGGATTGCAATTGGCGTATCTGACCTTGTCCAATCCACGATGGGAACAGGGAGATCTTGCAGACGATAAAGAATACAACGG<br>CATCTTTGTTCGGGCCTGGGGAAAGTCGAGACCTGTGATTCTCTGTGGCGGTTACACTCAAAACTCGGCCCGTCTGGCTGTAGACGAGCAGTTTGCTGAATATCAGATACTGATTGCCT<br>TTGGTCGTTTCTTCATCTCGACACCGGACCTTCCATTCCGTGCAAAGAAAGGACTTGAATTCAGCAAATACGACAGGGACACTTTCTACACCCCGTGCTCCAAGGCAGGGTATATTGAT<br>TACCCTTTTAGTGAGGAATTCGTGGCCCAGTCG**TAG**GTCCAGTGGTGGAACAGTAGGATATTATTATTTGGAAGACATTCAATTAGATTACAAGATCCGGAAGACAGACCTTCCCTATT<br>CCGCATTACTGCCCTTGTGACAGATTATTGAACTCAAATCATTGCCATTCCATCCCTTCCTGGTGATGAAAAATAGTGTCTGCCTTCACGAGC	MTKDSKLFKSIRLGPCNLHHRVVMAPLTRLRADDLYIQ<br>LPFVKDYYGQRASVPGTLLIAEATAISPQAVGFSNTPG<br>IWSTDQIHAWTEVTECVHQKGSYIFLQLWACGRAAEQE<br>ALDKDGIRFVSSSATAVDPYQRCPHPLTEGEIQDYITD<br>FATAARNAITAGFDGVEIHGAHGFLIDQFTQEICNKRT<br>DQWGGSIENRSRFALEVTKAVVDAVGADRVGIKLSPWA<br>EEFGMKMTRDIIPQFEFLIKALAGLQLAYLTLSNPRWE<br>QGDLADDKEYNGIFVRAWGKSRPVILCGGYTQNSARLA<br>VDEQFAEYQILIAFGRFFISTPDLPFRAKKGLEFSKYD<br>RDTFYTPCSKAGYIDYPFSEEFVAQS*

**SubG**	**ATG**GTAGCGTCTATTCGTTCCGAGTCGCCCATCTGGCAAAATCAGGTAGATGCTGCGAGATGGACATGGGTATTGCCGTTATTGGCCACCAGTCTCAGTCTCTTGTGGATTTTAGTACA<br>ACGATACTACCGTGCCGGACTGTCCAAATTCCCAGGACCGGCATTGGCCGCTGTGAGTGGCTTCTACAAGACTTATGTTGAGGTCTTCCAACAGGGTTGCTTTGTTCACAAATTGGAAG<br>AGCTACATGCCAAGTATGGTAAGATCATGCTCCGCAGCCAATCGTTGTTCCTGGCAGCACACTAAGTCGCAAGCCGATATTAGGAGATGTTGTGCGCACTGGGCCAAATGAGGTTCGTT<br>TACCATGGCCCTGAAGAGATCACCACTGCTACAACGGTCCCCTGATAACCTGATAGTTACATTTCCGAAATCCCTCGGCGTATGCAGACATCTACAACATGAAGAATCGCTGGGATAAA<br>GACCCCAAGCTTTATGAAGCATTCGGTGAATCCGACTCTTCCTTTGGCAGGCTGTCTTATCGCGCGGCCAAGGAGCGCAAGAACGTCTTGCAGTCGTTGTTCTCCAAAAAAGCTGCCGT<br>CCAAACCCACGAGCTGATCGAAGCCAAGGTACATTCAATGATAGCCCGGAGCGACTGGCTGCCAGTATGTGCTCACCACCAAGCTGCTTCGTAGGTAGTTGAGCTGTGTAACGCATTCG<br>AGAAATCATCAGGTGCTCCGGTCGATCTCCTCGGGGCCTTCAGATGCTTGTCAATTGAGACTATTGCATGTATACCTTCCCCGCCGCATCTACAGCACGCTGGGATAACCTGACACAGA<br>CGATTCGTGACTCTAGATATCTTCTGCGGTCAGTCCGTGGGTACTTTGCAATCACTCGATTTTGATCCACCTCTTTTGCAAGCAATGGACGAGTCTGTCAACTATATCATGCTTATGAA<br>GCACTTTCCGATCCTCCAAACTCTTATGCAACGTTGCCCCCCGTGGCTAGCTAAAATTCTGAGCCCGAAGGCAAGCGGAATGGTTGACTTCCGGGAGGTAGAGTCCCTTGGTTCTCCTC<br>GGTTGGTCGATAATGTATGACAATTTCAAGCGCTGACTTGGAGTGTCTTGTCTTTCATTCCAGTTTTTGGATCAGCAAGTCAGCACCCTTATGGCAGACAGAAAAAAGCTCGACAATCT<br>TCCGCATTCTTCCACCATCTACCATCTTCTACTAGGCCCGGAGGTCTCTGCACCACCGTCACACCTTAGTATTCTTGAAGAAACACAGACCCAGGTTTTTGCTGGAGAATCTTCGGGGC<br>TAACGCTGATGCATGGATCGTTTCATATTGTTCAGACACCGCGAGTATATCAAAAGCTCAAAGAGGAGCTCATTGCTACGTGGCCGAATCTTGATCAGCCACCAAGTCTTGCGACGCTG<br>GAGCGATTGCCCTACCTCGTAAGTCTTTCGATGTGACATGTGACTGCTCAGTCAACTTAACATTCTTCAGAATGCTGTCGTTAAGGAGTCTTTACGAATGGGTGGAGGAGCAACTTCTC<br>CTCTCCTCCGTGTTGTTCCCGAAACTGGCGCCATCATCGATGGTGTACTTATTGCTCCGGGAGTAAGTCCGTCCCCTTGTCTTTTCCGCGAATATACGGACACTTTGACAATCTTACAA<br>CGTCAGACTGTTGTTGGCTCGAGCAGTCACTTTGTCCATCGAAACGGGGATATCTTCCATGACCCCGATGAATACATTCCGGAAAGATGGATGGATGGGCGCGGAGATGAGCTTGAGCA<br>ACATCTAGTTGCATTCGGACGAGGTCCACGACGCTGTATTGGAGAAAAGTTAGTATTCCGTCTATTTTAATCTGTTGCCTGATAGCACAGCGTCTTGAAGCTAAAGGTGACGACAGTTT<br>TGCTTGGGTCATTCTCTTAACATGTATCGCCCACTTTTATCGCAAGTTCAACGTTGAGCTTGATCCCTCCAGGTAATGCACCATTTCGCGTAAATATTCTTGGGTGGTAGATCTTGAGC<br>GTATTTGCTGACAATAATCTCTATCTAGTCCCACAAAGTTGGACTGGAAGGATAGATTTGTTCCAGTGTATTCCACACCGCACCTCACTGTGAGAGTGAGCAAAGTGATCCTA**TAG**ATC<br>TTTCAACAATCCATTCGTTGCCTTCAACTTTGAAGTAACTACAGAGAACGTGAATCTGTTTATTTTGATACAGTGAGCTTCAAGGTCCAGTAGCATAGTGATCCTTGTACCTGTAATAA<br>TGTTCTATATGGTCTGCTAGTGGTCAGTGCTTTATTGAAGGCGGCCTTTGACGAAAGTTGGCTTTA	MVASIRSESPIWQNQVDAARWTWVLPLLATSLSLLWIL<br>VQRYYRAGLSKFPGPALAAVSGFYKTYVEVFQQGCFVH<br>KLEELHAKYGDVVRTGPNELHFRNPSAYADIYNMKNRW<br>DKDPKLYEAFGESDSSFGRLSYRAAKERKNVLQSLFSK<br>KAAVQTHELIEAKVVELCNAFEKSSGAPVDLLGAFRCL<br>SIETIAYIFCGQSVGTLQSLDFDPPLLQAMDESVNYIM<br>LMKHFPILQTLMQRCPPWLAKILSPKASGMVDFREFLD<br>QQVSTLMADRKKLDNLPHSSTIYHLLLGPEVSAPPSHL<br>SILEETQTQVFAGESSGLTLMHGSFHIVQTPRVYQKLK<br>EELIATWPNLDQPPSLATLERLPYLNAVVKESLRMGGG<br>ATSPLLRVVPETGAIIDGVLIAPGTVVGSSSHFVHRNG<br>DIFHDPDEYIPERWMDGRGDELEQHLVAFGRGPRRCIG<br>ENFAWVILLTCIAHFYRKFNVELDPSSPTKLDWKDRFV<br>PVYSTPHLTVRVSKVIL*
**SubH**	**ATG**CTCAAAGCCATACAATTCTGTGGCTCTCCGTCGCAGCAAATAGTACAGAGCAATTTTGATCTACCAGCCCCGCAAGGTAATGAGGTGCTCATTCGAGTCACCCATAGCGGAATTTG<br>CGGCAGCGAGCTTCATATGTTGAGCCGGCCACTTGTACTTGGACACGAGGGCGAGTGGATTGACCCAATTTCCAAGGCTTGCTTTTGTGAGAGTACTGACTATGAGGAAACAGGCATCG<br>GAATAGTCGAAGCAGTGGGACCAGGCTGTATACGCGTACAGGTTGGGGACCGAGTTGGCTGGGGTCCAATCAATGCTACCTGCGGGACCTGCGAGTTCTGCTGCAACGGCAGAGACAAT<br>TACTGCGAGGATATAAAGCTCTATGGCTTTGACTCCTATCGTCTTTCTGGGTCACTGTGTAGCCATGCATTACGCAAGGAACAATGGCTCTTTCGCATTCCCTCGGCCCTGTCGTCTAT<br>TGATGCAGCACCTTTGATGTGTCAGTTTCTTTCAGGCTGCTCCTTGGAGCTGAGATTTGTTTGATGAATGCTGATTCTCGCTCGACGTAGGTGGAGGCGGGACAGTCTGGGCCGCCTTG<br>ATGGAGCATTGCCGTCCCTTCCATAGGGTGGGAATCGTTGGCATTGGGGGACTCGGCCATCTAGCGATACAATTTGCCGCTAAAATCGGATGCCATGTCTCTGTTTTCTCTACGACGCC<br>GGAAAAGGAGGAAGAAGCACGTTGCCTAGGCGCTCATCAGTTCTTTTGTAGCAGTACCGGCGCTGAGCGCGTGTCGCTATGCGGCGATGGTTCGATCGACCGTCTTCTCATAACATCCT<br>CCTCGAAAGCTGATTTAGGATTGTTCTATTCCACGCTTCACCGCAATGCAGTTGTCGTGTTGCTATCAGTCGGAGATGGCGATCTTTCTGCTCCTTACATGCCAACTCTTCTCTCTGGA<br>ATGAGTATTGCCGGCAGCTGCATGTGCAGCCGCTACATGCAGTATGTTAATGACCTATACCCTTCCGGTGAGCCAAGATTTAATCCCTTGCAGGACCAAAATGCTCGACTTTGCTGCAC<br>GCCACGCCATAGCATGTACTGTGGAGCACTATCCTATGACATTGAAGGGTATTCAAGACGCGATAGAACGACTACGAAACGGCCAAATCCGGTACCGTGCGATTTTGAGCTGGGATAGC<br>GCTAGCAGTGTCGGTGTGCATGAAAAA**TGA**TCATATCCACATGTTTGCAAGTTCTAGACAAGCCAAGTGAGAACAATATTAGAGACCTTGAGTCGCCAGTCATAAGAAGAGGACAGGAG<br>CGGTGTGATAAAAGCCTAGATCCGGATCCTGCCAGGACAACCGAAGTAGTCACCCAGGAGCGTAGTGAATTGCTGCATCG	MLKAIQFCGSPSQQIVQSNFDLPAPQGNEVLIRVTHSG<br>ICGSELHMLSRPLVLGHEGIGIVEAVGPGCIRVQVGDR<br>VGWGPINATCGTCEFCCNGRDNYCEDIKLYGFDSYRLS<br>GSLCSHALRKEQWLFRIPSALSSIDAAPLMCGGGTVWA<br>ALMEHCRPFHRVGIVGIGGLGHLAIQFAAKIGCHVSVF<br>STTPEKEEEARCLGAHQFFCSSTGAERVSLCGDGSIDR<br>LLITSSSKADLGLFYSTLHRNAVVVLLSVGDGDLSAPY<br>MPTLLSGMSIAGSCMCSRYMQTKMLDFAARHAIACTVE<br>HYPMTLKGIQDAIERLRNGQIRYRAILSWDSASSVGVH<br>EK*
**SubI**	**ATG**ACTATCGAGAGTCATCCTACCGCTTCTGTCGAAAAGAACACCGATCTTTTTGACACTCCATTCCTTAGCGAGCATTATGATCTTCTGCTCGCGGATTACTTCAAAACGAAGCTGCT<br>TGATGTGCCTGTATACTGGCAGCTATTGAAGAAATGCACCCAGCCCAATCCGATCCTCCTGGACATTGGTACCGGCACTGGTCGGATTATAAGTGGCTTGATAGCAGAGGCCAAACGCG<br>AGAAGCTGGACATCTCAAATTGGACTTTCCTCGGAGTCGATATCTCGCAACACATGTTGGAACGAGCAAAAACCGTTACCTCAATCCCTCCGAAAAGCCCAACAGTGCAATGGATTAAG<br>GGGTCCGCCCAAGAGCTTGAAAAACTTCCACCACTGTGCAATGGCGCACAAAAAGTTGGCTTCATACTCTTCTCTGCTGGTGCGTTCAGCCACCTCTACGAGAAAGGTCAGCCAGAGCA<br>ATTTTTTTCGCAAGTCGCAAAAGTGATTGAACCCCGGACCGGAATGGCTTGCATCTCCATCGCAAAATCCTTCATGGTAACCGAGACTGGGAAGGAGTTTGCTTTACCTCAGATCGATG<br>GACCAAAGCAGATTGAGAGCAAAATATTCCCGGGAGTGATCTACGAGAATAGAATATTGTCCAACGAGACAGACGGTAACATCTTGACCGAGAAGCGGATCATTGATGTCATGCGAAAG<br>GATGACGGGCAAGAGAAAGTTATTGAGCGAAACCATGTCTCCAGTATGTTCAGACTTTGGAGTCGAGAAGGCTTCACCAAAATGGCCACTCATTTCGGGTTTGAAATCACGGACTTGAT<br>GGAGGGAACTGAGGAGACCTACTTCACACTGCAACTAAAGCAG**TAG**CCGCGACTTGGCCTACCACGCGTTCATCTTGTTTGCGACTAGCATTAATTGCCCGTTGAAGAAAACTTTTAGA<br>TAACCAACTAAGTACACTTGGCGGTAATGGATACCTGATTCAGGAACTGTATTTAGTTACGAATTCAATGTTTATCGGTTTTTTTACTCAGTAGAAAGGTCTTAGGCATTGCCAGTAT<br>CTCCCGCGAGATCAGAAACATCGAGGATGCAA	MTIESHPTASVEKNTDLFDTPFLSEHYDLLLADYFKTK<br>LLDVPVYWQLLKKCTQPNPILLDIGTGTGRIISGLIAE<br>AKREKLDISNWTFLGVDISQHMLERAKTVTSIPPKSPT<br>VQWIKGSAQELEKLPPLCNGAQKVGFILFSAGAFSHLY<br>EKGQPEQFFSQVAKVIEPRTGMACISIAKSFMVTETGK<br>EFALPQIDGPKQIESKIFPGVIYENRILSNETDGNILT<br>EKRIIDVMRKDDGQEKVIERNHVSSMFRLWSREGFTKM<br>ATHFGFEITDLMEGTEETYFTLQLKQ*
**SubJ**	**ATG**TGGGCCGAATCGATTATCGCTCGATTACCCTTTGCTCTCGTGATATGGGTATTATTTTTGGGCTTGCGGTCAATTTACAGACTCAGTTTCCATCCTCTACGGCATATTCCAGGTCC<br>GAAACTTGCGGCTGTCACGCATCTCTACGAATTCTACTTTGACGTGATCTGCCGTGGCCGGTATATCTGGCAAATACAAAAGCTTCACGCAAAATACGGTTTGGGATTGTCATCCAGCG<br>TTCGTGTACTGAAAGCTAATAAAGACTAGGTCCGATCATAAGGATCAGCCCTCGCGAGATCCATATTGCGGACGCTTCATTCTATGATGAGATCTATGCTCCAAGTAGCCGTATACGAG<br>ACAAGGACGCAAAATGGGTGCCAACTTTTGGTCTGTCACAGTCCATGTTGAGCACAGTCGGGCATAGACAGCATCGAGTTCGACGTTCACTGCTATCGAGCTTCTTCTCAAAGAAGTCA<br>GTACTGGAAATCGGACCGCTGATTGAGAGACAAATTGCAAAATTGTCTCATCGCCTGAAGAATCTCCACAGCACACAGGAGGTGGTACAACTTGATGCCGCGTTTGTCGCACTCACTTC<br>TGACATTATCTCTGCCTACTGCTATGGCGAGTCAGGTAACTTCTTAGACAACCCAGGCTTCCGAAGCGACATTCGGGCTTCGGTGATCGATGCCACTGCCATTTGCCACTTGGTCCGGT<br>TTTTCCCTTTCCTTCCGCCCCTCCTGCAGCAGATTCCAGAGACCCTCATGCGGAAGTTCATGCCGGGGAAGATAGCTATAATTAACTTTCAACGTTCTTTGGCGGCAAATGCTGTTCAA<br>GCTCTGCGCGGAGAAAGGAAGGTTGGAACTGACCAAGACACAATGTACGACAGGCTGATCAACGAAGCCGTTCCCTGCGAAGAGAGGACACTCGGTCGTATTCAAGATGAGAGCTCGCT<br>CGTCCTCGCGGCTGGAACAGAGACAACTGGGCGCATCTTGACAGTTGCCATATATTATTTGAGCAAGGATCAGTCGATCCTTCGCAAGCTACGCGCAGAGCTCAAGACAGTCCTCCCTT<br>CGCCGACCGCTTCCGTAACCTGGGTGCAGCTCGAGCAACTTCCCTATTTGGTACGTCAAATCTTGTTCCTGTTCACATTTCTTTCTTTTCGCCATCAACGACATTAACTTAAAATTTCA<br>GACAGCTGTCGTTCGTGAATCCTTACGCCTTGGAATGGGTATTACTTGCCGTCTTCCTCGCGTGGCTCCAAACGAGGTCTTGCAGTACGGGAAGTACCACATTCCAGCTGGGGTGAGTA<br>CAATTTCTCGTGGAGGATTAATGGACGAAGCATACTCTGTTACCCACAGAATCTGATACGATAGTTCGCCCACAGACACCGATGAGCACGTGTACTATGCTCGTCCAACGTAACCCAGA<br>CATGTTTCCTGATCCGGATAGATTTGACCCGGAGCGGTGGATCACCGACGGTCAAGTCGATAACAAGCTTATGAAATATCTTGTCTCATTTACTCGAGGGAGCAGAATGTGCTTGGGGA<br>TTAAGTGAGTCACATCAATCAACCATTGAAGCAATTGCTCTGTACTGATAACAGAGCCACAGCCTGGCATACAGCGAACTCTATCTTCTGATATCCCACCTGGTTCGGCAGTTCACTTT<br>CGAGATCTGTAACACAAGTGACGATGACGTTCGAATTACTCGTGATATGATGGTCGGGTACACCCGACGAGGAGATCTCCAAGTATATGCGAAGGTGGCGAAGGTTATTGAGGAG**TAG**T<br>AGGCCAGTGAATCATCATGTTTCGACGAAACGTGGTGAAATGGAAACAAACATATTTGTCTAGCTCTTACCTAGTGGTCAGTTTAGCCTTCTACCCTAGATCTGATGGCATTCACCTTC<br>GTGTCTGGGAGTATCTTTCCTGCAGGATATTGAAACACATCAGCCCTCATAGGCCGTTTTTGGTTGATTCCCTCTCTACGGATTTAACTTCGGTTTTTTGGATCTGCATCCTGCGAATT<br>AACGGAAAGGGTGACGATGGAGGGCATCGAATGCACGCGTAGATAGATCTTCTCCTTTTCGTCGAATGCACCATTGAATTACTAATCTTCACGATTGGCTGTTCCGCTCTGGTCCATTT<br>TGAAAGTCACGAATCATGCCGAGTATAGGTT	MWAESIIARLPFALVIWVLFLGLRSIYRLSFHPLRHIP<br>GPKLAAVTHLYEFYFDVICRGRLGPIIRISPREIHIAD<br>ASFYDEIYAPSSRIRDKDAKWVPTFGLSQSMLSTVGHR<br>QHRVRRSLLSSFFSKKSVLEIGPLIERQIAKLSHRLKN<br>LHSTQEVVQLDAAFVALTSDIISAYCYGESGNFLDNPG<br>FRSDIRASVIDATAICHLVRFFPFLPPLLQQIPETLMR<br>KFMPGKIAIINFQRSLAANAVQALRGERKVGTDQDTMY<br>DRLINEAVPCEERTLGRIQDESSLVLAAGTETTGRILT<br>VAIYYLSKDQSILRKLRAELKTVLPSPTASVTWVQLEQ<br>LPYLTAVVRESLRLGMGITCRLPRVAPNEVLQYGKYHI<br>PAGTPMSTCTMLVQRNPDMFPDPDRFDPERWITDGQVD<br>NKLMKYLVSFTRGSRMCLGINLAYSELYLLISHLVRQF<br>TFEICNTSDDDVRITRDMMVGYTRRGDLQVYAKVAKVI<br>EE*
**SubK**	**ATG**CAAGCTAGCAAGACTGTCATCCTCGCCGAGCGCGTTTCAGACTGTGGGCGCCCTAAACCTCTAGTCGAGTCGCTTTCATTTCTGTCGACTGGTAGCAATAAACAGATTTCATCTGC<br>GAAGAATATGAACCGACTTACAGTGGATGCCATTCTTGAACCATCCTCGCAAACGGTTCTCCTTCAACTTTGCTGGGCCGCTGTATTAGCCAGATATGCGGAAACTCCCGATATTCAAT<br>ATGCATACGTTCTGGAGGACTCGGAGGAAGTCAGGTTATTTCACTCACAGATCTCTTCCGGTCAAGACGTGTATGACGCGCTGGAATCGGCTGCGTATGTCACGTCTCCTTTGGGGCTC<br>ATTGGCGATTCTCTTTCCCAGGAAGCAATCAGCAGCCAGACTGGAAATCTCGCCTTCGGCACCATCCTACTGGTCAAAGGCAAAAATAGCCCTTCTAGGATATCTCTCACCAAAGAACT<br>CTTCACTGGGACCGCTTTATTACTGGAATGCGCTGTTGCGGCCCAACATGTGCAAGTTGAATGGACATGCAGAAAACCTCTCGCGCCCGAGTATGTGTACATGATCATGACACAATTCG<br>CCCATGCTCTGCGGGTCAGCACGAAGAGTAGAAAAGAGAATATTCGTTACCGCGATCTCCAAGAAATTAGCCCCGAGGGATTCAGTCAAATCATCGAGTGGAATGGCGGACGCGAATAC<br>GTCACGCGGGAAACCTTTGCGCATCACCTGTTTGAGAAGATGGTCATAAAACAGCCCCATGCGCCTGCGATTTGTGCTTGGGATGGTGACATGACCTACGGCGAGCTCGACTCATACGC<br>CGAATTTGTGGCATCGCAAATCATGGCATCATGTCAAATCGAACATGAAGGCGTCTTGGGTCTTTTTTTTGCAAAGTCGAAGTGGACAGCTGTGGCCATGCTAGGAGCGCTCAAGGCTG<br>GTAGGGGGTTCATCATGTTTCATACCTCTCAGCCGCCCACACGCCTGCGAACAATCTGTGAGCGCCTAGGAGTCCTTGCCGTCCTTTCGCTGCCGGGATTCAAAACCGCCCTGGATGAG<br>TTGGACGTCCCCAAGATCATCCTCTCTACTGACAAAGATGAGGGTTTCCTACCGAACACCGCCAGCCAAAAGCCCGCCCCTTTACCTTCTGAATCAAATAGCATTCTCTATGCTGGATT<br>CACGTCGGGTTCTACTGGTGAGCCCAAGATATTCGATGTTCGCCATTCTGCATTCTGCAGCGGTATCTCTGCCTGGGGCGTGGCAATGAACGTTAACGAGCGGTCCCGGGTCTTTCAAA<br>GTGCGTCATATAACTTTACGGTCAGCGTGATAGAGCAATTAGCCCCCTTGTGTCTCGGTGGGTGTGTGTGCGTCCCTTCCGAGGAAGCGCTGCAAAACGACATCCCCGGAGCCATTCGT<br>GATCTCGGTGCCAATTGGGCTGAATTTACCCCGAGTTTGGCGCGATTGTTGGATCCCAGCACTTTGTCTGGAGTGAAAACGCTGGTTCTGACCGGCGAAGCTTTGGCCAGAGCAGATGT<br>CGAGAAATGGCACTGTCATCTTGATTTGCGGGTGTATTATGGACAATCTGAGCATTCCCTGGGTGCCACGGTTGGGAACACCGCAGACCTCGTCGCGGACCACCGCAACATCGGCCGCA<br>CCTTTTCGGCCAGGGAATGGATCGTTGACCCAGAAGACCATAACCGGCTCGCTCCACTTGGAGCTGAAGGCGAGCTCTTGTTAGAAGGGCCCTGTGTCGGAGTGGGTTATCTCAATAAC<br>CCAAAGGAGACAGATGCCATCTTCGTACGGAACCCTGCGTGGTTCCTATCTCAGCGATTGACTCCAGTGACTGACGGGCGATTTCTCAAGACCGGTGATCTTGTCTGCTACGATATGCA<br>CCGAGGTTGCCTTCAGTATCGTGGTCGCAGAGGAACCCAGGTCAAGATCCGCGGTCAACGTGTCGACCTCGGTGAGGTTGAGTATCATGCAGGCAAGCATTTGTCTTCGGCACAAGGTA<br>AGCCGGTAGCGGAGGTAATTTGCCCCAAAGTATATGCCCAAAAGTCAGATCCTGTGATGGTCGTTTTTCTTGTGACAGATACGCCAGTGGAAATGGCAGTGACATCCTACGAAGATTTT<br>GATTCTCCCATCATCTTCCCCCCAAACGACAAACTCAGGGCACAGGCGAAAGCTGCATTGGCTCGAATGAAAGATGTTTTGCCAGACTATATGATGCCAAGCGCCGTTATTCCAATCTC<br>GCATCTTCCACGGACGCCTTCTGGCAAGCTCTTTCGACGCAGGCTCCGCGACATCGGCTCACAGCTTGCTCTAGACGAAATTGTGGCCTATGTGAATCTTCGAAAACCACACCAAGCTC<br>CAGAAACTGCCAACGAAGAGAAGTTGCAGCAGATATCTGCAAGTATTCTTGGCTGCTCCGCGAGTCAGATCGGCATGAACGACACCTTCCTAGAGCTGGGAGGCGACTCGTTGAAAGTC<br>AGGCACTTGGTGCAAGCCGCGCGTACTGAAGGCGTGACACTGTCTGTCGGTGACATTTTCAAAAACTTGACCCTGGCAGATCTCGCTCGAATTGCTCAGGAAGGTCCCATGTCTGTACC<br>TGCTCATGAGAAACAACCAGAGGAGCCGGTCGTAAAGAGCTTCCTTCAGAGCAAGCCCGAATTCGCAATTGGATCGCGCATCGAGAGTGTCTCGCGCACACACGATATAGTCAAAGCCA<br>CCGGTAACGAGAACGCTGTGGAGTATTTCCTCTTCAGCCTGTATGGCCCTCTAGACGTCGAGCGGCTCGATGTCGCCTGTCAAGCCCTTGTGGCTTGTCATGCAGTTTTGCGGTCCGTG<br>TTCATCCCGTACGAGGAGGAGATCATGCAAGTCACATTTCGCTCATTGAAAGCCTCGTTTGAAGTTATCGAGGCGTCTCCAGGTGTTGATCCAAAAGTACTCACCGACCGACTTTGCCA<br>TGAGGATAAGATGCAGTCACCCTTCGGACCGACCCCAGTGATGGCATTTACCGTCGTTAGATCAAGTCCCGACAATCACATCCTTGTTTTGCGACTGTCTGAAGCTCAATGTGATGCCC<br>ATAGCTTGAAGAGACTGATCTCAAACCTGATGCTGCTCTACGATGAAACGTCAATCCATGTCCAACTCGACTACCGTGACTATCTGGCTATCTGTGCATCACAAAGGACTCCAGAAGCG<br>TTTGCCTATTGGAGGACACTTCTGAAAGGTGCCACTCCTCTCCATCTTGACAGCATACGACTCAATACCCAATCATCTTGGATGTGTCATGGGCAGCTCACTGGGAAAACCTACCACCA<br>CGATTCTCCTCCCAGTCTCACGTTCTCTCGTACAATACCTCCCGTCACCGCTCGCCGAGGTATTACCCTCGCCACCACGATCAAGGCTGCCTGGTCGTACGTACTGTACAAGAAATTGC<br>ATTGCAATGACATTCTTTTCGGTCAGCTCGGAAGCTGCCGCACCATGAATGTACCTGGTATCGAGGATACCATTGGCCCATGTACTAACGTGACGCCAGTCCGCGTAAAATATGTGCCC<br>CAGTGGGATCCCAGCGAGATCCTGCAGACGATTCAAGAACAGCACGCACAGTCCCTTCCCTACCAGACAATAGGCTGGCGGGATATTGTTAACCATTGCACCACGTGGCCCGATGGTGC<br>TTCCCTCCAGACAGTGGTGTTGCACCAGGGCGAGCAAGAGGACACAGAGATCCAAACTCGGGGTGGACTGAAATGCCGTGCCCTTGACTGGAGCGCATCAAGTATTCTTGGTCGTCTGT<br>ATTTGATGACTGAGAGTACCTCATCAGGGTTGAACATTGAACTCTGTGTGCCTCGTGGCGCTATTGACAACTCGGAAGCAGAAGGGCTCTTCGATCTCTTCTGCAGCGCAATAGGCACC<br>TTCTGCAGCTGGAAAAACCGCGACAGCCATGCTACAAGCCGAATCATC**TGA**TGGCGTCGTGGTCGCCACTGAGCAAAGGAAGCAGGAAAAAGAGAGGGGAAAATCGGTCTTGGTCAATC<br>TCGCGATCTCCAACGAAGCAGAACAGCAAAGGGGAAGCAGAGGCCTCTTGTAGGCCGACAGATCTAGGCGCAGGGCCTGGACAAATAAGGAATCTGCCGCCGATGGGAGGAATCTCCAT<br>CATCATCGCTTTCA	MQASKTVILAERVSDCGRPKPLVESLSFLSTGSNKQIS<br>SAKNMNRLTVDAILEPSSQTVLLQLCWAAVLARYAETP<br>DIQYAYVLEDSEEVRLFHSQISSGQDVYDALESAAYVT<br>SPLGLIGDSLSQEAISSQTGNLAFGTILLVKGKNSPSR<br>ISLTKELFTGTALLLECAVAAQHVQVEWTCRKPLAPEY<br>VYMIMTQFAHALRVSTKSRKENIRYRDLQEISPEGFSQ<br>IIEWNGGREYVTRETFAHHLFEKMVIKQPHAPAICAWD<br>GDMTYGELDSYAEFVASQIMASCQIEHEGVLGLFFAKS<br>KWTAVAMLGALKAGRGFIMFHTSQPPTRLRTICERLGV<br>LAVLSLPGFKTALDELDVPKIILSTDKDEGFLPNTASQ<br>KPAPLPSESNSILYAGFTSGSTGEPKIFDVRHSAFCSG<br>ISAWGVAMNVNERSRVFQSASYNFTVSVIEQLAPLCLG<br>GCVCVPSEEALQNDIPGAIRDLGANWAEFTPSLARLLD<br>PSTLSGVKTLVLTGEALARADVEKWHCHLDLRVYYGQS<br>EHSLGATVGNTADLVADHRNIGRTFSAREWIVDPEDHN<br>RLAPLGAEGELLLEGPCVGVGYLNNPKETDAIFVRNPA<br>WFLSQRLTPVTDGRFLKTGDLVCYDMHRGCLQYRGRRG<br>TQVKIRGQRVDLGEVEYHAGKHLSSAQGKPVAEVICPK<br>VYAQKSDPVMVVFLVTDTPVEMAVTSYEDFDSPIIFPP<br>NDKLRAQAKAALARMKDVLPDYMMPSAVIPISHLPRTP<br>SGKLFRRRLRDIGSQLALDEIVAYVNLRKPHQAPETAN<br>EEKLQQISASILGCSASQIGMNDTFLELGGDSLKVRHL<br>VQAARTEGVTLSVGDIFKNLTLADLARIAQEGPMSVPA<br>HEKQPEEPVVKSFLQSKPEFAIGSRIESVSRTHDIVKA<br>TGNENAVEYFLFSLYGPLDVERLDVACQALVACHAVLR<br>SVFIPYEEEIMQVTFRSLKASFEVIEASPGVDPKVLTD<br>RLCHEDKMQSPFGPTPVMAFTVVRSSPDNHILVLRLSE<br>AQCDAHSLKRLISNLMLLYDETSIHVQLDYRDYLAICA<br>SQRTPEAFAYWRTLLKGATPLHLDSIRLNTQSSWMCHG<br>QLTGKTYHHDSPPSLTFSRTIPPVTARRGITLATTIKA<br>AWSYVLYKKLHCNDILFGQLGSCRTMNVPGIEDTIGPC<br>TNVTPVRVKYVPQWDPSEILQTIQEQHAQSLPYQTIGW<br>RDIVNHCTTWPDGASLQTVVLHQGEQEDTEIQTRGGLK<br>CRALDWSASSILGRLYLMTESTSSGLNIELCVPRGAID<br>NSEAEGLFDLFCSAIGTFCSWKNRDSHATSRII*

Table S4. Primers and gene fragments used in this study.

Primer/Gene Fragment	Sequence	Desscription
pYTU.ANgpdAp_F	TAACCTCGCGGGTGTTCTTGACGATGGCATCCTGCACTCCGGTGAATTGATTTGGGTGAC	Amplification of ANgpdAp with homology to pYTU
pYTP.ANgpdAp_F	CTCGCGGGTGTTCTTGACGATGGCATCCTGCGGCCGCACTCCGGTGAATTGATTTGGGTG	Amplification of ANgpdAp with homology to pYTP
ANgpdAp_R	TGTTTAGATGTGTCTATGTGGCGGG	Amplification of ANgpdAp
PEgpdAp_F	GATTCGTCCAGGGCTTCCCAAG	Amplification of PEgpdAp
PEgpdAp_R	GATTGCGGTTTACTAGAAGTTGTTAGATC	Amplification of PEgpdAp
POgpdAp_F	TTTGCTCCAGGAATACATGTGAGC	Amplification of POgpdAp
POgpdAp_R	TTTTGCGATTGTTTGAAGTGTTCTGTATG	Amplification of POgpdAp
ANcoxAp_F	AATGCCTGATCCAGCCCCCAGGC	Amplification of ANcoxAp
ANcoxAp_R	TGTCCTGGTGGGTGGGTTGC	Amplification of ANcoxAp
ANgpdAp.subA F	ATTACCCCGCCACATAGACACATCTAAACAATGACCGTGACAAGCGAAGACCATCCACTG	Amplification of subA with homology to ANgpdAp
subA.pYTU R	TTCAACACAGTGGAGGACATACCCGTAATTTTCTGGTCATATCCAGTATCGACCGAGACC	Amplification of subA with homology to pYTU
ANgpdAp.subB F	ACCCCGCCACATAGACACATCTAAACAATGATAAATCTACTAGCTGTTACGTTTCTTGTG	Amplification of subB with homology to ANgpdAp
subB.pYTU R	TCAACACAGTGGAGGACATACCCGTAATTTTCTGGATTTCAAGTAGTGCATGCTGCAGTG	Amplification of subB with homology to pYTU
ANgpdAp.subE F	TTGACTAACCATTACCCCGCCACATAGACACATCTAAACAATGGCGATCGGAAATCAAGC	Amplification of subE with homology to ANgpdAp
subE.pYTU R	GACTTCAACACAGTGGAGGACATACCCGTAATTTTCTGAATCGTACGTCATTCTGCGAGG	Amplification of subE with homology to pYTU
ANgpdAp.subJ F	GACTAACCATTACCCCGCCACATAGACACATCTAAACAATGTGGGCCGAATCGATTATCG	Amplification of subJ with homology to ANgpdAp
subJ.pYTU R	CTTCAACACAGTGGAGGACATACCCGTAATTTTCTGAACCTATACTCGGCATGATTCGTG	Amplification of subJ with homology to pYTU
ANgpdAp.subF F	CTTGACTAACCATTACCCCGCCACATAGACACATCTAAACAATGACGAAGGATTCGAAGC	Amplification of subF with homology to ANgpdAp
subF.pYTU R	ACACAGTGGAGGACATACCCGTAATTTTCTGGCTCGTGAAGGCAGACACTATTTTTCATC	Amplification of subF with homology to pYTU
ANgpdAp.subH F	TAACCATTACCCCGCCACATAGACACATCTAAACAATGCTCAAAGCCATACAATTCTGTG	Amplification of subH with homology to ANgpdAp
subH.pYTP R	AGTGATGAGACCCAACAACCATGATACCAGGGGATTTCGATGCAGCAATTCACTACGCTC	Amplification of subH with homology to pYTP
subF_F196Y F	ATTCACGGCGCACACGGCTACCTGATCG	Introduction of F196Y mutation into subF
ANgpdAp.subG F	GACTAACCATTACCCCGCCACATAGACACATCTAAACAATGGTAGCGTCTATTCGTTCCG	Amplification of subG with homology to ANgpdAp
subG.pYTU R	ACTTCAACACAGTGGAGGACATACCCGTAATTTTCTGTAAAGCCAACTTTCGTCAAAGGC	Amplification of subG with homology to pYTU
ANgpdAp.subC F	CTAACCATTACCCCGCCACATAGACACATCTAAACAATGGGATCCGTCAGCAAAGCATAC	Amplification of subC with homology to ANgpdAp
subC.pYTP R	AACAACCATGATACCAGGGGATTTCTACCTGGAATACTTTAACTGTTGTTTTGAGCCAGG	Amplification of subC with homology to pYTP
subC.PEgpdAp R	ACCTTGGGAAGCCCTGGACGAATCCTACCTGGAATACTTTAACTGTTGTTTTGAGCCAGG	Amplification of subC with homology to PEgpdAp
subC.ANcoxAp R	GGCCTGGGGGCTGGATCAGGCATTCTACCTGGAATACTTTAACTGTTGTTTTGAGCCAGG	Amplification of subC with homology to ANcoxAp
ANcoxAp.subF F	TCATTGCAACCCACCCACCAGGACAATGACGAAGGATTCGAAGCTTTTCAAAAGTATTCG	Amplification of subF with homology to ANcoxAp
subF.PEgpdAp R	TATTCCAACCTTGGGAAGCCCTGGACGAATCGCTCGTGAAGGCAGACACTATTTTTCATC	Amplification of subF with homology to PEgpdAp
PEgpdAp.subH F	ATACATGATCTAACAACTTCTAGTAAACCGCAATCATGCTCAAAGCCATACAATTCTGTG	Amplification of subH with homology to PEgpdAp
subH.PEgpdAp R	TAGAATCAGTAAGCTCACATGTATTCCTGGAGCAAATCGATGCAGCAATTCACTACGCTC	Amplification of subH with homology to PEgpdAp
POgpdAp.subI F	CAAGTGCATACAGAACACTTCAAACAATCGCAAAAATGACTATCGAGAGTCATCCTACCG	Amplification of subI with homology to POgpdAp
subI.pYTP R	GTGATGAGACCCAACAACCATGATACCAGGGGATTTGCATCCTCGATGTTTCTGATCTCG	Amplification of subI with homology to pYTP
subE.POgpdAp R	GTAGAATCAGTAAGCTCACATGTATTCCTGGAGCAAAGAATCGTACGTCATTCTGCGAGG	Amplification of subE with homology to POgpdAp
POgpdAp.subA F	GCATACAGAACACTTCAAACAATCGCAAAAATGACCGTGACAAGCGAAGACCATCCACTG	Amplification of subA with homology to POgpdAp
subA.pYTR R	CTAAAGGGTATCATCGAAAGGGAGTCATCCAATTTGTCATATCCAGTATCGACCGAGACC	Amplification of subA with homology to pYTR
subA.ANcoxAp R	GGTCCTCGAGGCCTGGGGGCTGGATCAGGCATTTTGTCATATCCAGTATCGACCGAGACC	Amplification of subA with homology to ANcoxAp
ANcoxAp.subB F	CGTCATTGCAACCCACCCACCAGGACAATGATAAATCTACTAGCTGTTACGTTTCTTGTG	Amplification of subB with homology to ANcoxAp
subB.PEgpdAp R	CAATATTCCAACCTTGGGAAGCCCTGGACGAATCGATTTCAAGTAGTGCATGCTGCAGTG	Amplification of subB with homology to PEgpdAp
subB.pYTR R	GCTAAAGGGTATCATCGAAAGGGAGTCATCCAATGATTTCAAGTAGTGCATGCTGCAGTG	Amplification of subB with homology to pYTR
subG.pYTR R	TCTGCTAAAGGGTATCATCGAAAGGGAGTCATCCAATTAAAGCCAACTTTCGTCAAAGGC	Amplification of subG with homology to pYTR
PEgpdAp.subG F	CTTATACATGATCTAACAACTTCTAGTAAACCGCAATCATGGTAGCGTCTATTCGTTCCG	Amplification of subG with homology to PEgpdAp
subJ.POgpdAp R	TAGAATCAGTAAGCTCACATGTATTCCTGGAGCAAAAACCTATACTCGGCATGATTCGTG	Amplification of subJ with homology to POgpdAp
POgpdAp.subD F	TTACAAGTGCATACAGAACACTTCAAACAATCGCAAAAATGAGAGGTGTGCGAGAAACCC	Amplification of subD with homology to POgpdAp
subD.ANcoxAp R	AGTTTTGGTCCTCGAGGCCTGGGGGCTGGATCAGGCATTCCCCACCACCATCTCATTCCC	Amplification of subD with homology to ANcoxAp
subD.pYTU R	TGACTTCAACACAGTGGAGGACATACCCGTAATTTTCTGCCCCACCACCATCTCATTCCC	Amplification of subD with homology to pYTU
subK.pYTU R	AACACAGTGGAGGACATACCCGTAATTTTCTGTGAAAGCGATGATGATGGAGATTCCTCC	Amplification of subK with homology to pYTU
pJBU2bb1 F	AACGAAGCATCTGTGCTTCATTTTGTAG	Amplification of pJBU2 backbone
pJBU2bb1 R	CAGTCAAATTGCAGTACTCTGCG	Amplification of pJBU2 backbone

pJBU2bb2 F	GGTTCCTTTGTTACTTCTTCTGCC	Amplification of pJBU2 backbone
pJBU2bb2 R	GGCGTTTTTCCATAGGCTCCG	Amplification of pJBU2 backbone
pJBU2.ADH2p F	TGCTCGTCAGGGGGGCGGAGCCTATGGAAAAACGCCGCAAAACGTAGGGGCAAACAAACG	Amplification of ADH2p fragment with homology to pJBU2 backbone
ADH2p R	GGTATTACGATATAGTTAATAGTTGATAGTTGATTG	Amplification of ADH2p fragment
ADH2p.subE F	CATACAATCAACTATCAACTATTAACTATATCGTAATACCATGGCGATCGGAAATCAAGC	Amplification of subE with homology to ADH2p
subE.PRM9t R	CTGGTAAAGTTGTGTGCTAGTGTCTCCCGTCTTCTGTCCTACAACAGTCGAGAACCTTCC	Amplification of subE with homology to PRM9t
PRM9t F	GACAGAAGACGGGAGACACTAGCAC	Amplification of PRM9t-PCK1p fragment
PCK1p R	GTTGTTATTTTATTATGGAATAATTAGTTGCGTG	Amplification of PRM9t-PCK1p fragment
PCK1p.subA F	CAACTAATTATTCCATAATAAAATAACAACATGACCGTGACAAGCGAAGACCATCCACTG	Amplification of subA with homology to PCK1p
subA.CPS1t R	AAAAAAAAAAAATCTTTGACTATTCAATCATTGCGCTCAAGAATGTAGCGGAAGCTGTCG	Amplification of subA with homology to CPS1t
CPS1t F	GCGCAATGATTGAATAGTCAAAGATT	Amplification of CPS1t fragment
CPS1t.pJBU2 R	TTGTTCTACAAAATGAAGCACAGATGCTTCGTTATTTGACACTTGATTTGACACTTCTTT	Amplification of CPS1t fragment with homology to pJBU2 backbone
pJBU2bb.2824up F	CGTTGCATTTTTGTTCTACAAAATGAAGCACAGATGCTTCGTTCTATACTGGCCGGGCGC	Amplification of 2 kb DNA upstream of A. terreus subF with homology to pJBU2 backbone
2824up.hyg R	GTCCGTCCGTCTCTCCGCATGGGTTGGAGTTCAGCAAGTATCACCGG	Amplification of 2 kb DNA upstream of A. terreus subF with homology to hydromycin marker
2824up.hyg F	CCGGTGATACTTGCTGAACTCCAACCCATGCGGAGAGACGGACGGAC	Amplification of hygromycin marker with homology to DNA upstream of A. terreus subF
hyg.2824down R	GACCTTGCCACCTTCAGCACTCGAGTGGAGATGTGGAGTGGG	Amplification of hygromycin marker with homology to DNA downstream of A. terreus subF
hyg.2824down F	CCCACTCCACATCTCCACTCGAGTGCTGAAGGTGGCAAGGTC	Amplification of 2 kb DNA downstream of A. terreus subF with homology to hydromycin marker
2824down.pJBU2bb R	GTGATGCTCGTCAGGGGGGCGGAGCCTATGGAAAAACGCCTTATACGCGGGCAACGTACC	Amplification of 2 kb DNA downstream of A. terreus subF with homology to pJBU2 backbone
ANcoxAp.subK F	TTGTCTGCCGTCATTGCAACCCACCCACCAGGACAATGCAAGCTAGCAAGACTGTCATCC	Amplification of subK with homology to ANcoxAp
2824_gRNA F	ATAAGATCTGCGTAAGCTCCCTAATTGGCC	Amplification of pFC330 insert Δ02824_guide_RNA
2824_gRNA R	ATATTAATTAAGAGCCAAGAGCGGATTCCTC	Amplification of pFC330 insert Δ02824_guide_RNA
pET28a subEa F	TAATGCTAGCATGGCGATCGGAAATCAAGC	Amplification of subE fragment A
pET28a subEa R	GGTGATAAGACGAGTGGAAATGTTCATGTCAACATGAGAGAAGACATTTGCCAGCTTGGACG	Amplification of subE fragment A
pET28a subEb F	CGTCCAAGCTGGCAAATGTCTTCTCTCATGTTGACATGAACATTTCCACTCGTCTTATCACC	Amplification of subE fragment B
pET28a subEb R	CGAGTGCGGCCGCTCTACAACAGTCGAGAACCTTCC	Amplification of subE fragment B
XP_001212002_ko	GCGTAAGCTCCCTAATTGGCCCATCCGGCATCTGTAGGGCGTCCAAATATCGTGCCTCTCCTGCTTTGCCCGGTGTATGAAA<br>CCGGAAAGGCCGCTCAGGAGCTGGCCAGCGGCGCAGACCGGGAACACAAGCTGGCAGTCGACCCATCCGGTGCTCTGCAC<br>TCGACCTGCTGAGGTCCCTCAGTCCCTGGTAGGCAGCTTTGCCCCGTCTGTCCGCCCGGTGTGTCGGCGGGGTTGACAAGG<br>TCGTTGCGTCAGTCCAACATTTGTTGCCATATTTTCCTGCTCTCCCCACCAGCTGCTCTTTTCTTTTCTCTTTCTTTTCCCATC<br>TTCAGTATATTCATCTTCCCATCCAAGAACCTTTATTTCCCCTAAGTAAGTACTTTGCTACATCCATACTCCATCCTTCCCAT<br>CCCTTATTCCTTTGAACCTTTCAGTTCGAGCTTTCCCACTTCATCGCAGCTTGACTAACAGCTACCCCGCTTGAGCAGACAT<br>CACCGATATAGCTGATGAGTCCGTGAGGACGAAACGAGTAAGCTCGTCCTATATTGAGGACTATGCGCGTTTTAGAGCTAG<br>AAATAGCAAGTTAAAATAAGGCTAGTCCGTTATCAACTTGAAAAAGTGGCACCGAGTCGGTGCTTTTGGCCGGCATGGTCC<br>CAGCCTCCTCGCTGGCGCCGGCTGGGCAACATGCTTCGGCATGGCGAATGGGACTGATTTAATAGCTCCATGTCAACAAGA<br>ATAAAACGCGTTTCGGGTTTACCTCTTCCAGATACAGCTCATCTGCAATGCATTAATGCATTGGACCTCGCAACCCTAGTAC<br>GCCCTTCAGGCTCCGGCGAAGCAGAAGAATAGCTTAGCAGAGTCTATTTTCATTTTCGGGAGACGAGATCAAGCAGATCAA<br>CGGTCGTCAAGAGACCTACGAGACTGAGGAATCCGCTCTTGGCTC	XP_001212002_knockout_gRNA_expression_cassette (IDT gBlock), protospacer in the gRNA is underlined

Table S5. Plasmids used in this study.

Plasmid	Vector	Insert	Construction method
pJB265	pYTU	ANgpdAp-*subF*^{F196Y}	Yeast homologous recombination
pJB086	pYTP	ANgpdAp-*subC*-PEgpdAp-*subH*	Yeast homologous recombination
pJB087	pYTP	ANgpdAp-*subH*	Yeast homologous recombination
pJB112	pFC330	Δ02824_gRNA	Digestion, ligation
pJB142	pJBU2	Δ02824_repair_fragment	Yeast homologous recombination
pJB208	pYTU	ANgpdAp-*subA*	Yeast homologous recombination
pJB209	pYTU	ANgpdAp-*subB*	Yeast homologous recombination
pJB210	pYTU	ANgpdAp-*subE*	Yeast homologous recombination
pJB213	pYTU	ANgpdAp-*subJ*	Yeast homologous recombination
pJB215	pYTR	ANgpdAp-*subE*-POgpdAp-*subA*	Yeast homologous recombination
pJB216	pYTR	ANgpdAp-*subE*-POgpdAp-*subA*-ANcoxAp-*subB*	Yeast homologous recombination
pJB231	pET28a	T7p-N-His_6-*subE*-T7t	Splicing by overlap extension, digestion, ligation
pJB232	pYTU	ANgpdAp-*subJ*-POgpdAp-*subD*	Yeast homologous recombination
pJB233	pYTU	ANgpdAp-*subJ*-POgpdAp-*subD*-ANcoxAp-*subK*	Yeast homologous recombination
pJB234	pYTU	none	Previous study
pJB235	pYTP	none	Previous study
pJB236	pYTR	none	Previous study
pJB237	pYTR	ANgpdAp-*subE*-POgpdAp-*subA*-ANcoxAp-*subB*-PEgpdAp-*subG*	Yeast homologous recombination
pJB239	pYTP	ANgpdAp-*subC*	Yeast homologous recombination
pJB242	pYTP	ANgpdAp-*subC-ANcoxAp-subF*-PEgpdAp-*subH*-POgpdAp-*subI*	Yeast homologous recombination
pJB243	pYTU	ANgpdAp-*subG*	Yeast homologous recombination
pJB250	pJBU2	ADH2p-*subE*-PRM9t-PCK1p-*subA*-CPS1t	Yeast homologous recombination
pJB265	pYTU	ANgpdAp-*subF*	Yeast homologous recombination

ANgpdAp = *Aspergillus niger* gpdAp
PEgpdAp = *Penicillium expansum* gpdAp
POgpdAp = *Penicillium oxalicum* gpdAp
ANcoxAp = *Aspergillus niger* coxAp

Table S6. Strains constructed in this study.

FJBNNN/ YJBNNN	Parent	Modification	Subrubine genes	Selectable marker
FJB003	*Aspergillus terreus* NIH2624	ΔATEG_02824	ΔsubF	*hph*
FJB011	*Aspergillus nidulans* A1145ΔEMΔST	pJB234, pJB235, pJB215	subAE	*pyrG, pyroA, riboB*
FJB020	*Aspergillus nidulans* A1145ΔEMΔST	pJB237, pJB234, pJB242	subAEBGCFHI	*pyrG, pyroA, riboB*
FJB044	*Aspergillus nidulans* A1145ΔEMΔST	pJB234, pJB235, pJB236	none	*pyrG, pyroA, riboB*
FJB045	*Aspergillus nidulans* A1145ΔEMΔST	pJB208, pJB235, pJB236	subA	*pyrG, pyroA, riboB*
FJB046	*Aspergillus nidulans* A1145ΔEMΔST	pJB210, pJB235, pJB236	subE	*pyrG, pyroA, riboB*
FJB047	*Aspergillus nidulans* A1145ΔEMΔST	pJB209, pJB235, pJB236	subB	*pyrG, pyroA, riboB*
FJB048	*Aspergillus nidulans* A1145ΔEMΔST	pJB243, pJB235, pJB236	subG	*pyrG, pyroA, riboB*
FJB049	*Aspergillus nidulans* A1145ΔEMΔST	pJB213, pJB235, pJB236	subJ	*pyrG, pyroA, riboB*
FJB052	*Aspergillus nidulans* A1145ΔEMΔST	pJB243, pJB235, pJB215	subAEG	*pyrG, pyroA, riboB*
FJB053	*Aspergillus nidulans* A1145ΔEMΔST	pJB213, pJB235, pJB215	subAEJ	*pyrG, pyroA, riboB*
FJB054	*Aspergillus nidulans* A1145ΔEMΔST	pJB234, pJB235, pJB237	subAEBG	*pyrG, pyroA, riboB*
FJB055	*Aspergillus nidulans* A1145ΔEMΔST	pJB213, pJB235, pJB216	subAEBJ	*pyrG, pyroA, riboB*
FJB056	*Aspergillus nidulans* A1145ΔEMΔST	pJB234, pJB239, pJB237	subAEBGC	*pyrG, pyroA, riboB*
FJB057	*Aspergillus nidulans* A1145ΔEMΔST	pJB265, pJB235, pJB237	subAEBGF	*pyrG, pyroA, riboB*
FJB058	*Aspergillus nidulans* A1145ΔEMΔST	pJB234, pJB087, pJB237	subAEBGH	*pyrG, pyroA, riboB*
FJB059	*Aspergillus nidulans* A1145ΔEMΔST	pJB265, pJB239, pJB237	subAEBGCF	*pyrG, pyroA, riboB*
FJB060	*Aspergillus nidulans* A1145ΔEMΔST	pJB234, pJB086, pJB237	subAEBGCH	*pyrG, pyroA, riboB*
FJB061	*Aspergillus nidulans* A1145ΔEMΔST	pJB265, pJB087, pJB237	subAEBGFH	*pyrG, pyroA, riboB*
FJB062	*Aspergillus nidulans* A1145ΔEMΔST	pJB265, pJB086, pJB237	subAEBGCFH	*pyrG, pyroA, riboB*
FJB063	*Aspergillus nidulans* A1145ΔEMΔST	pJB085, pJB086, pJB237	subAEBGCF^{F196Y}H	*pyrG, pyroA, riboB*
FJB064	*Aspergillus nidulans* A1145ΔEMΔST	pJB233, pJB242, pJB237	subAEBGCFHIJDK	*pyrG, pyroA, riboB*
FJB065	*Aspergillus nidulans* A1145ΔEMΔST	pJB209, pJB235, pJB215	subAEB	*pyrG, pyroA, riboB*
FJB067	*Aspergillus nidulans* A1145ΔEMΔST	pJB213, pJB242, pJB237	subAEBGCFHIJ	*pyrG, pyroA, riboB*
FJB068	*Aspergillus nidulans* A1145ΔEMΔST	pJB232, pJB242, pJB237	subAEBGCFHIJD	*pyrG, pyroA, riboB*
YJB092	*Saccharomyces cerevisiae* JHY651	pJB250	subAE	*ura3*

Table S7. LC-MS chromatograms presented in this study.

Sample	Dilution factor	Solvent	Strain/Sample	Media	Time	Extraction protocol
102	1	MeOH	FJB011	CD solid	7 days	200 mg solid culture, 1 mL acetone extract, 250 uL dry, 0.05 mL MeOH resuspend, centrifuge
103	1	MeOH	FJB020	CD solid	7 days	200 mg solid culture, 1 mL acetone extract, 250 uL dry, 0.05 mL MeOH resuspend, centrifuge
104	1	MeOH	FJB044	CD solid	7 days	200 mg solid culture, 1 mL acetone extract, 250 uL dry, 0.05 mL MeOH resuspend, centrifuge
105	1	MeOH	FJB045	CD solid	7 days	200 mg solid culture, 1 mL acetone extract, 250 uL dry, 0.05 mL MeOH resuspend, centrifuge
106	1	MeOH	FJB046	CD solid	7 days	200 mg solid culture, 1 mL acetone extract, 250 uL dry, 0.05 mL MeOH resuspend, centrifuge
107	1	MeOH	FJB047	CD solid	7 days	200 mg solid culture, 1 mL acetone extract, 250 uL dry, 0.05 mL MeOH resuspend, centrifuge
108	1	MeOH	FJB048	CD solid	7 days	200 mg solid culture, 1 mL acetone extract, 250 uL dry, 0.05 mL MeOH resuspend, centrifuge
109	1	MeOH	FJB049	CD solid	7 days	200 mg solid culture, 1 mL acetone extract, 250 uL dry, 0.05 mL MeOH resuspend, centrifuge
110	1	MeOH	FJB052	CD solid	7 days	200 mg solid culture, 1 mL acetone extract, 250 uL dry, 0.05 mL MeOH resuspend, centrifuge
111	1	MeOH	FJB053	CD solid	7 days	200 mg solid culture, 1 mL acetone extract, 250 uL dry, 0.05 mL MeOH resuspend, centrifuge
112	1	MeOH	FJB054	CD solid	7 days	200 mg solid culture, 1 mL acetone extract, 250 uL dry, 0.05 mL MeOH resuspend, centrifuge
113	1	MeOH	FJB055	CD solid	7 days	200 mg solid culture, 1 mL acetone extract, 250 uL dry, 0.05 mL MeOH resuspend, centrifuge
114	1	MeOH	FJB056	CD solid	7 days	200 mg solid culture, 1 mL acetone extract, 250 uL dry, 0.05 mL MeOH resuspend, centrifuge
115	1	MeOH	FJB057	CD solid	7 days	200 mg solid culture, 1 mL acetone extract, 250 uL dry, 0.05 mL MeOH resuspend, centrifuge
116	1	MeOH	FJB058	CD solid	7 days	200 mg solid culture, 1 mL acetone extract, 250 uL dry, 0.05 mL MeOH resuspend, centrifuge
117	1	MeOH	FJB059	CD solid	7 days	200 mg solid culture, 1 mL acetone extract, 250 uL dry, 0.05 mL MeOH resuspend, centrifuge
118	1	MeOH	FJB060	CD solid	7 days	200 mg solid culture, 1 mL acetone extract, 250 uL dry, 0.05 mL MeOH resuspend, centrifuge
119	1	MeOH	FJB061	CD solid	7 days	200 mg solid culture, 1 mL acetone extract, 250 uL dry, 0.05 mL MeOH resuspend, centrifuge
120	1	MeOH	FJB062	CD solid	7 days	200 mg solid culture, 1 mL acetone extract, 250 uL dry, 0.05 mL MeOH resuspend, centrifuge
121	1	MeOH	FJB063	CD solid	7 days	200 mg solid culture, 1 mL acetone extract, 250 uL dry, 0.05 mL MeOH resuspend, centrifuge
122	1	MeOH	FJB064	CD solid	7 days	200 mg solid culture, 1 mL acetone extract, 250 uL dry, 0.05 mL MeOH resuspend, centrifuge
123	1	MeOH	FJB065	CD solid	7 days	200 mg solid culture, 1 mL acetone extract, 250 uL dry, 0.05 mL MeOH resuspend, centrifuge
124	1	MeOH	FJB067	CD solid	7 days	200 mg solid culture, 1 mL acetone extract, 250 uL dry, 0.05 mL MeOH resuspend, centrifuge
125	1	MeOH	FJB068	CD solid	7 days	200 mg solid culture, 1 mL acetone extract, 250 uL dry, 0.05 mL MeOH resuspend, centrifuge
126	1	MeOH	FJB011	CD solid	7 days	200 mg solid culture, 1 mL acetone extract, 250 uL dry, 0.05 mL MeOH resuspend, centrifuge
127	1	MeOH	FJB011	CD solid	7 days	200 mg solid culture, 1 mL acetone extract, 250 uL dry, 0.05 mL MeOH resuspend, centrifuge
128	1	MeOH	FJB020	CD solid	7 days	200 mg solid culture, 1 mL acetone extract, 250 uL dry, 0.05 mL MeOH resuspend, centrifuge
129	1	MeOH	FJB020	CD solid	7 days	200 mg solid culture, 1 mL acetone extract, 250 uL dry, 0.05 mL MeOH resuspend, centrifuge
130	1	MeOH	FJB044	CD solid	7 days	200 mg solid culture, 1 mL acetone extract, 250 uL dry, 0.05 mL MeOH resuspend, centrifuge
131	1	MeOH	FJB044	CD solid	7 days	200 mg solid culture, 1 mL acetone extract, 250 uL dry, 0.05 mL MeOH resuspend, centrifuge
132	1	MeOH	FJB045	CD solid	7 days	200 mg solid culture, 1 mL acetone extract, 250 uL dry, 0.05 mL MeOH resuspend, centrifuge
133	1	MeOH	FJB045	CD solid	7 days	200 mg solid culture, 1 mL acetone extract, 250 uL dry, 0.05 mL MeOH resuspend, centrifuge
134	1	MeOH	FJB046	CD solid	7 days	200 mg solid culture, 1 mL acetone extract, 250 uL dry, 0.05 mL MeOH resuspend, centrifuge
135	1	MeOH	FJB046	CD solid	7 days	200 mg solid culture, 1 mL acetone extract, 250 uL dry, 0.05 mL MeOH resuspend, centrifuge
136	1	MeOH	FJB047	CD solid	7 days	200 mg solid culture, 1 mL acetone extract, 250 uL dry, 0.05 mL MeOH resuspend, centrifuge
137	1	MeOH	FJB047	CD solid	7 days	200 mg solid culture, 1 mL acetone extract, 250 uL dry, 0.05 mL MeOH resuspend, centrifuge
138	1	MeOH	FJB048	CD solid	7 days	200 mg solid culture, 1 mL acetone extract, 250 uL dry, 0.05 mL MeOH resuspend, centrifuge
139	1	MeOH	FJB048	CD solid	7 days	200 mg solid culture, 1 mL acetone extract, 250 uL dry, 0.05 mL MeOH resuspend, centrifuge
140	1	MeOH	FJB049	CD solid	7 days	200 mg solid culture, 1 mL acetone extract, 250 uL dry, 0.05 mL MeOH resuspend, centrifuge
141	1	MeOH	FJB049	CD solid	7 days	200 mg solid culture, 1 mL acetone extract, 250 uL dry, 0.05 mL MeOH resuspend, centrifuge
142	1	MeOH	FJB052	CD solid	7 days	200 mg solid culture, 1 mL acetone extract, 250 uL dry, 0.05 mL MeOH resuspend, centrifuge
143	1	MeOH	FJB052	CD solid	7 days	200 mg solid culture, 1 mL acetone extract, 250 uL dry, 0.05 mL MeOH resuspend, centrifuge
144	1	MeOH	FJB053	CD solid	7 days	200 mg solid culture, 1 mL acetone extract, 250 uL dry, 0.05 mL MeOH resuspend, centrifuge
145	1	MeOH	FJB053	CD solid	7 days	200 mg solid culture, 1 mL acetone extract, 250 uL dry, 0.05 mL MeOH resuspend, centrifuge
146	1	MeOH	FJB054	CD solid	7 days	200 mg solid culture, 1 mL acetone extract, 250 uL dry, 0.05 mL MeOH resuspend, centrifuge
147	1	MeOH	FJB054	CD solid	7 days	200 mg solid culture, 1 mL acetone extract, 250 uL dry, 0.05 mL MeOH resuspend, centrifuge
148	1	MeOH	FJB055	CD solid	7 days	200 mg solid culture, 1 mL acetone extract, 250 uL dry, 0.05 mL MeOH resuspend, centrifuge
149	1	MeOH	FJB055	CD solid	7 days	200 mg solid culture, 1 mL acetone extract, 250 uL dry, 0.05 mL MeOH resuspend, centrifuge
150	1	MeOH	FJB056	CD solid	7 days	200 mg solid culture, 1 mL acetone extract, 250 uL dry, 0.05 mL MeOH resuspend, centrifuge
151	1	MeOH	FJB056	CD solid	7 days	200 mg solid culture, 1 mL acetone extract, 250 uL dry, 0.05 mL MeOH resuspend, centrifuge
152	1	MeOH	FJB057	CD solid	7 days	200 mg solid culture, 1 mL acetone extract, 250 uL dry, 0.05 mL MeOH resuspend, centrifuge
153	1	MeOH	FJB057	CD solid	7 days	200 mg solid culture, 1 mL acetone extract, 250 uL dry, 0.05 mL MeOH resuspend, centrifuge
154	1	MeOH	FJB058	CD solid	7 days	200 mg solid culture, 1 mL acetone extract, 250 uL dry, 0.05 mL MeOH resuspend, centrifuge
155	1	MeOH	FJB058	CD solid	7 days	200 mg solid culture, 1 mL acetone extract, 250 uL dry, 0.05 mL MeOH resuspend, centrifuge
156	1	MeOH	FJB059	CD solid	7 days	200 mg solid culture, 1 mL acetone extract, 250 uL dry, 0.05 mL MeOH resuspend, centrifuge
157	1	MeOH	FJB059	CD solid	7 days	200 mg solid culture, 1 mL acetone extract, 250 uL dry, 0.05 mL MeOH resuspend, centrifuge
158	1	MeOH	FJB060	CD solid	7 days	200 mg solid culture, 1 mL acetone extract, 250 uL dry, 0.05 mL MeOH resuspend, centrifuge
159	1	MeOH	FJB060	CD solid	7 days	200 mg solid culture, 1 mL acetone extract, 250 uL dry, 0.05 mL MeOH resuspend, centrifuge
160	1	MeOH	FJB061	CD solid	7 days	200 mg solid culture, 1 mL acetone extract, 250 uL dry, 0.05 mL MeOH resuspend, centrifuge
161	1	MeOH	FJB061	CD solid	7 days	200 mg solid culture, 1 mL acetone extract, 250 uL dry, 0.05 mL MeOH resuspend, centrifuge
162	1	MeOH	FJB062	CD solid	7 days	200 mg solid culture, 1 mL acetone extract, 250 uL dry, 0.05 mL MeOH resuspend, centrifuge
163	1	MeOH	FJB062	CD solid	7 days	200 mg solid culture, 1 mL acetone extract, 250 uL dry, 0.05 mL MeOH resuspend, centrifuge
164	1	MeOH	FJB063	CD solid	7 days	200 mg solid culture, 1 mL acetone extract, 250 uL dry, 0.05 mL MeOH resuspend, centrifuge
165	1	MeOH	FJB063	CD solid	7 days	200 mg solid culture, 1 mL acetone extract, 250 uL dry, 0.05 mL MeOH resuspend, centrifuge
166	1	MeOH	FJB064	CD solid	7 days	200 mg solid culture, 1 mL acetone extract, 250 uL dry, 0.05 mL MeOH resuspend, centrifuge
167	1	MeOH	FJB064	CD solid	7 days	200 mg solid culture, 1 mL acetone extract, 250 uL dry, 0.05 mL MeOH resuspend, centrifuge
168	1	MeOH	FJB065	CD solid	7 days	200 mg solid culture, 1 mL acetone extract, 250 uL dry, 0.05 mL MeOH resuspend, centrifuge
169	1	MeOH	FJB065	CD solid	7 days	200 mg solid culture, 1 mL acetone extract, 250 uL dry, 0.05 mL MeOH resuspend, centrifuge
170	1	MeOH	FJB067	CD solid	7 days	200 mg solid culture, 1 mL acetone extract, 250 uL dry, 0.05 mL MeOH resuspend, centrifuge
171	1	MeOH	FJB067	CD solid	7 days	200 mg solid culture, 1 mL acetone extract, 250 uL dry, 0.05 mL MeOH resuspend, centrifuge
172	1	MeOH	FJB068	CD solid	7 days	200 mg solid culture, 1 mL acetone extract, 250 uL dry, 0.05 mL MeOH resuspend, centrifuge
173	1	MeOH	FJB068	CD solid	7 days	200 mg solid culture, 1 mL acetone extract, 250 uL dry, 0.05 mL MeOH resuspend, centrifuge
174	1	MeOH	FJB004	CD solid + 20 mg/L tryptophan	7 days	200 mg solid culture, 1 mL acetone extract, 250 uL dry, 0.05 mL MeOH resuspend, centrifuge
175	1	MeOH	FJB004	CD solid +20 mg/L d5-tryptophan	7 days	200 mg solid culture, 1 mL acetone extract, 250 uL dry, 0.05 mL MeOH resuspend, centrifuge
178	1	MeOH	FJB003	CD solid	7 days	200 mg solid culture, 1 mL acetone extract, 250 uL dry, 0.05 mL MeOH resuspend, centrifuge
180	1	MeOH	FJB001	CD solid	7 days	200 mg solid culture, 1 mL acetone extract, 250 uL dry, 0.05 mL MeOH resuspend, centrifuge
182	1	MeOH	FJB004	CD solid	7 days	200 mg solid culture, 1 mL acetone extract, 250 uL dry, 0.05 mL MeOH resuspend, centrifuge
187	1	MeOH				1 mg/L compound **1** in MeOH
186	1	MeOH				1 mg/L compound **2** in MeOH
185	1	MeOH				1 mg/L compound **3** in MeOH
188	1	MeOH				1 mg/L compound **4** in MeOH
189	1	MeOH				1 mg/L compound **5** in MeOH
190	1	MeOH				1 mg/L compound **6** in MeOH
191	1	MeOH				1 mg/L compound **15** in MeOH
199	1	MeOH				1 mg/L tryptamine in MeOH
201	1	MeOH				1 mg/L 3-indoleacetic acid in MeOH
206	0.1	MeOH	YJB092	YPD	3 days	100 uL culture + 900 uL MeOH, centrifuge
208	0.01	MeOH	subE rxn, boiled enzyme			1 uL rxn + 99 uL MeOH, centrifuge
210	0.01	MeOH	subE rxn			1 uL rxn + 99 uL MeOH, centrifuge

Table S8. Spectroscopic data for subrubine A (**1**).

Subrubine A (1): brown amorphous solid; $[\alpha]_D$ −5 (c 0.2, MeOH); UV ($MeCN/H_2O/FA$, 45:55:0.1) λ_{max} 208, 222, and 312 nm. HRESIMS m/z 394.2131 (calcd for $C_{23}H_{28}N_3O_3$: 394.2125, $[M+H]^+$)

	**1** in methanol-d_6	
position	^{13}C	^1H (J in Hz)
1	–	–
2	100.8	–
3	69.6	–
4	114.3	6.57 (1H, d, 2.5)
5	154.1	–
6	110.8	6.64 (1H, dd, 8.3, 2.5)
7	108.0	6.44 (1H, d, 8.3)
8	147.7	–
9	137.3	–
10	41.6	2.01 (2H, m)
11	58.0	2.91, 2.42 (each 1H, m)
12	–	–
13	36.1	2.35 (3H, s)
14	56.6	3.70 (3H, s)
1'	38.3	2.06, 1.80 (each 1H, m)
2'	38.1	1.98, 1.60 (each 1H, m)
3'	50.9	–
4'	19.7	1.11 (3H, s)
5'	70.5	4.49, 4.35 (each 1H, d, 11.0)
6'	168.2	–
7'	131.6	–
8',12'	130.5	8.07 (2H, d, 7.5)
9',11'	129.7	7.50 (2H, t, 7.5)
10'	134.3	7.62 (1H, t, 7.5)

Selected 2D NMR correlations

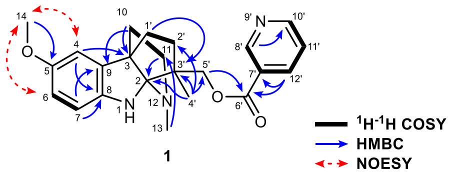

Selected NOESY correlations

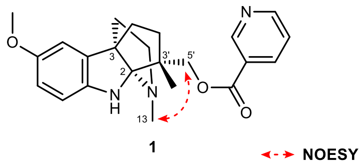

UV spectrum

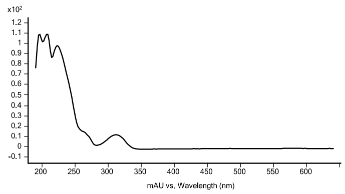

Table S9. Spectroscopic data for subrubine B (**2**).

Subrubine B (2): brown amorphous solid; $[\alpha]_D$ −13 (*c* 0.2, MeOH); UV (MeCN/H_2O/FA, 17:83:0.1) λ_{max} 202, 236, and 310 nm. HRESIMS *m/z* 416.2559 (calcd for $C_{23}H_{34}N_3O_4$: 416.2544, $[M+H]^+$)

	**2** in DMSO-d_6	
position	^{13}C	^1H (*J* in Hz)
1	–	5.68 (1H, brs)
2	98.9	–
3	67.6	–
4	109.9	6.58 (1H, d, 2.7)
5	151.7	–
6	112.6	6.47 (1H, dd, 8.4, 2.7)
7	106.0	6.32 (1H, d, 8.4)
8	146.5	–
9	135.8	–
10	40.8[a]	1.88 (2H, m)
11	56.9	2.79, 2.21 (each 1H, m)
12	–	–
13	35.7	2.15 (3H, s)
14	55.6	3.61 (3H, s)
1'	37.2	1.90, 1.69 (each 1H, m)
2'	36.7	1.74, 1.36 (each 1H, m)
3'	49.2	–
4'	19.2	0.91 (3H, s)
5'	69.1	4.16, 3.98 (each 1H, d, 10.8)
6'	171.5	–
7'	56.1	4.31 (1H, dd, 8.7, 5.6)
8'	30.1	2.09 (1H, m)
9'	18.0	0.87 (3H, d, 6.6)
10'	19.1	0.88 (3H, d, 6.6)
11'	–	8.45 (1H, brd, 8.7)
12'	161.5	8.09 (1H, brs)

[a] Detected by HMBC

Selected 2D NMR correlations

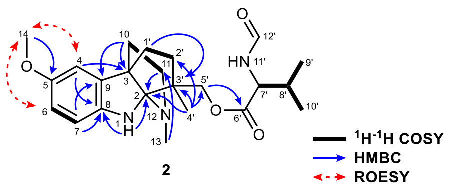

Selected ROESY correlations

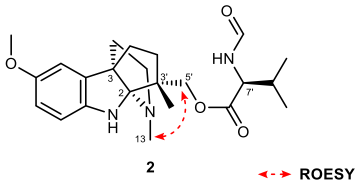

UV spectrum

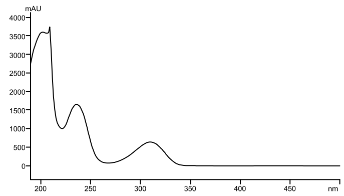

Table S10. Spectroscopic data for subrubine C (**3**).

Subrubine C (3): brown amorphous solid; $[\alpha]_D$ −8 (*c* 0.1, MeOH); UV (MeCN/H_2O/FA, 17:83:0.1) λ_{max} 204, 235, and 309 nm. HRESIMS *m/z* 393.2177 (calcd for $C_{24}H_{29}N_2O_3$: 393.2173, $[M+H]^+$)

	**3** in DMSO-d_6	
position	^{13}C	^1H (*J* in Hz)
1	–	5.81 (1H, brs)
2	98.9	–
3	67.8	–
4	109.8	6.61 (1H, s)
5	151.6	–
6	112.7	6.49 (1H, d, 8.4)
7	106.2	6.35 (1H, d, 8.4)
8	146.6	–
9	135.8	–
10	40.5	1.91 (2H, m)
11	56.8	2.28, 2.23 (each 1H, m)
12	–	–
13	35.7	2.21 (3H, s)
14	55.6	3.62 (3H, s)
1'	37.2	1.98, 1.73 (each 1H, m)
2'	37.0	1.89, 1.45 (each 1H, m)
3'	49.4	–
4'	19.2	1.03 (3H, s)
5'	69.5	4.41, 4.28 (each 1H, d, 10.9)
6'	165.1	–
7'	126.0	–
8'	150.3	9.19 (1H, brs)
9'	–	–
10'	153.9	8.84 (1H, brs)
11'	124.2	7.59 (1H, brt, 7.7)
12'	137.2	8.36 (1H, brd, 7.7)

Selected 2D NMR correlations

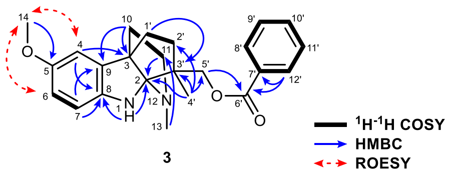

3

Selected ROESY correlations

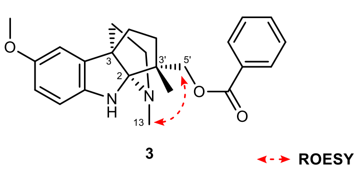

3

UV spectrum

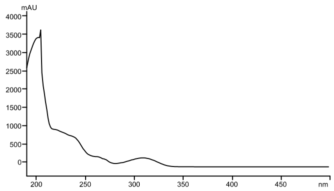

Table S11. Spectroscopic data for isolated and synthetic pensubrubine (**4**).

Pensubrubine (4): brown amorphous solid; $[\alpha]_D$ −8 (*c* 0.1, MeOH); UV (MeCN/H_2O/FA, 38:62:0.1) λ_{max} 205, 236, and 312 nm. HRESIMS *m/z* 289.1905 (calcd for $C_{17}H_{25}N_2O_2$: 289.1911, $[M+H]^+$)

	Isolated **4** in methanol-d_4		Synthetic **4** in methanol-d_4	
position	^{13}C	^{1}H (*J* in Hz)	^{13}C	^{1}H (*J* in Hz)
1	–	–	–	–
2	101.2	–	101.2	–
3	69.4	–	69.4	–
4	110.8	6.61 (1H, d, 2.6)	110.8	6.62 (1H, d, 2.5)
5	154.1	–	154.1	–
6	114.2	6.54 (1H, dd, 8.4, 2.6)	114.2	6.55 (1H, dd, 8.4, 2.5)
7	107.8	6.37 (1H, d, 8.4)	107.8	6.38 (1H, d, 8.4)
8	147.3	–	147.3	–
9	137.4	–	137.4	–
10	41.1	2.01 (2H, m)	41.0	2.01 (2H, m)
11	57.8	2.91 (1H, dd, 9.1, 5.0)	57.8	2.91 (1H, dd, 9.2, 6.2)
		2.31 (1H, m)		2.32 (1H, ddd, 12.3, 9.2, 5.5)
12	–	–	–	–
13	36.2	2.36 (3H, s)	36.2	2.37 (3H, s)
14	56.6	3.68 (3H, s)	56.6	3.69 (3H, s)
1'	38.1	2.02, 1.78 (each 1H, m)	38.1	2.03, 1.78 (each 1H, m)
2'	37.5	2.05, 1.40 (each 1H, m)	37.5	2.06, 1.41 (each 1H, m)
3'	51.3	–	51.3	–
4'	19.8	0.90 (3H, s)	19.8	0.91 (3H, s)
5'	68.1	3.65, 3.63 (each 1H, d, 11.2)	68.1	3.65, 3.63 (each 1H, d, 11.2)

Selected 2D NMR correlations

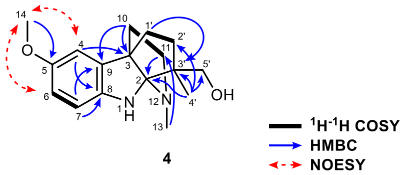

Selected NOESY correlations

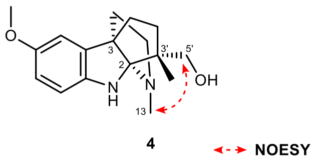

UV spectrum

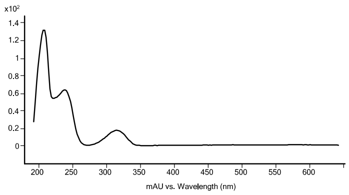

Table S12. Spectroscopic data for demethoxypensubrubine (**5**).

Demethoxypensubrubine (5): brown amorphous solid; $[\alpha]_D$ −10 (*c* 0.2, MeOH); UV (MeCN/H_2O/FA, 38:62:0.1) λ_{max} 204, 237, and 294 nm. HRESIMS *m/z* 259.1799 (calcd for $C_{16}H_{23}N_2O$: 259.1805, $[M+H]^+$)

	**5** in methanol-d_4	
position	^{13}C	^1H (*J* in Hz)
1	–	–
2	106.5	–
3	68.8	–
4	124.1	6.65 (1H, d, 7.7)
5	120.8	6.77 (1H, td, 7.7, 1.3)
6	129.9	7.08 (1H, td, 7.7, 1.3)
7	109.3	7.13 (1H, d, 7.7)
8	151.0	–
9	134.5	–
10	39.7	2.34 (1H, dd, 13.4, 5.4)
		2.16 (1H, 13.4, 6.2)
11	56.6	3.49 (1H, 11.0, 6.2)
		2.68 (1H, m)
12	–	–
13	35.8	2.86 (3H, s)
1'	36.8	2.20 (1H, m)
		1.90 (1H, td, 12.3, 6.5)
2'	35.3	2.28 (1H, m)
		1.52 (1H, ddd, 13.0, 6.5, 2.1)
3'	51.5	–
4'	19.5	0.89 (3H, s)
5'	65.2	3.80, 3.69 (each 1H, d, 11.9)

Selected 2D NMR correlations

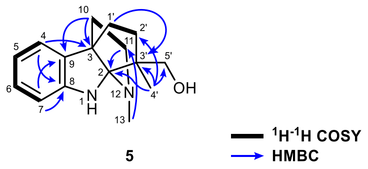

Selected NOESY correlations

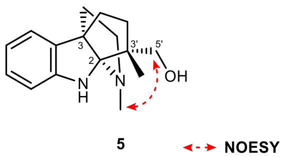

UV spectrum

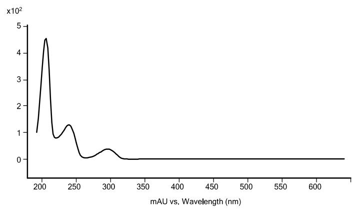

Table S13. Spectroscopic data for nordemethoxypensubrubine (**6**).

Nordemethoxypensubrubine (6): brown amorphous solid; $[\alpha]_D$ −10 (*c* 0.1, MeOH); UV ($MeCN/H_2O/FA$, 37:63:0.1) λ_{max} 204, 236, and 294 nm. HRESIMS *m/z* 245.1646 (calcd for $C_{15}H_{21}N_2O$: 245.1648, $[M+H]^+$)

	**6** in methanol-d_4	
position	^{13}C	^1H (*J* in Hz)
1	–	–
2	103.9	–
3	66.5	–
4	124.3	6.63 (1H, d, 7.9)
5	120.9	6.77 (1H, td, 7.9, 1.0)
6	129.8	7.07 (1H, td, 7.9, 1.0)
7	109.6	7.14 (1H, d, 7.9)
8	151.2	–
9	134.4	–
10	41.4	2.41 (1H, dd, 13.3, 5.5)
		2.14 (1H, m)
11	46.1	3.43 (1H, m)
		2.68 (ddd, 13.3, 11.2, 5.5)
12	–	–
1'	37.3	2.26 (1H, m)
		1.98 (1H, m)
2'	34.6	2.07 (1H, m)
		1.57 (1H, ddd, 12.7, 6.2, 1.5)
3'	51.4	–
4'	19.5	0.96 (3H, s)
5'	66.5	3.79, 3.69 (each 1H, d, 11.5)

Selected 2D NMR correlations

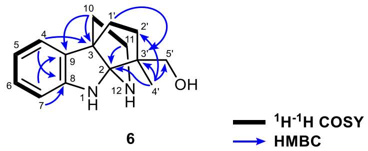

6

UV spectrum

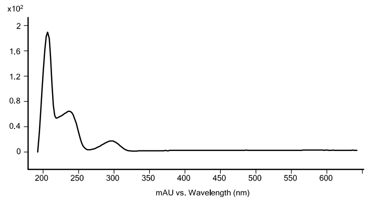

Table S14. Spectroscopic data for **15**.

Compound 15: brown amorphous solid; $[\alpha]_D$ −42 (*c* 0.2, MeOH); UV (MeCN/H_2O/FA, 46:54:0.1) λ_{max} 204, 231, and 292 nm. HRESIMS *m/z* 229.1691 (calcd for $C_{15}H_{21}N_2$: 229.1699, $[M+H]^+$)

	**15** in pyridine-d_5	
position	^{13}C	^{1}H (J in Hz)
1	–	–
2	82.4	5.54 (1H, s)
3	58.6	–
4	123.0	7.19 (1H, d, 7.4)
5	119.0	6.86 (1H, t, 7.4)
6	128.6	7.14 (1H, td, 7.4)
7	109.1	6.78 (1H, d, 7.4)
8	151.3	–
9	133.8	–
10	39.4	2.35 (1H, td, 11.9, 6.8)
		2.14 (1H, dd, 12.5, 5.6)
11	44.5	3.36 (1H, dd, 10.5, 7.2)
		2.94 (1H, td, 11.1, 5.8)
12	–	–
1'	37.4	2.69 (1H, dd, 14.5, 8.1)
		2.61 (1H, dd, 14.7, 6.5)
2'	120.5	5.25 (1H, t, 7.1)
3'	134.4	–
4'	25.8	1.54 (3H, s)
5'	18.1	1.54 (3H, s)

Selected 2D NMR correlations

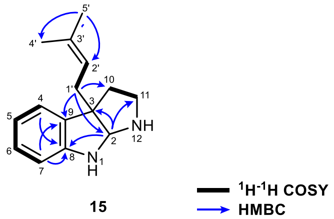

Selected ROESY correlations

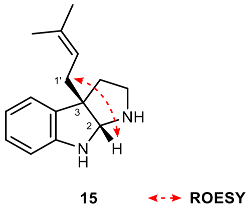

UV spectrum

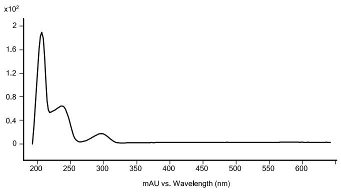

Table S15. Spectroscopic data for **16**.

Compound 16: brown amorphous solid; $[\alpha]_D$ −23 (*c* 0.1, MeOH); UV (MeCN/H_2O/FA, 80:20:0.1) λ_{max} 202 and 240, 296 nm. HRESIMS *m/z* 243.1864 (calcd for $C_{16}H_{22}N_2$: 243.1856, $[M+H]^+$)

	**16** in acetonitrile-d_3	
position	^{13}C	^1H (J in Hz)
2	NA[a]	–
3	58.3	–
4	124.2	7.09 (1H, d, 7.4)
5	120.1	6.75 (1H, t, 7.4)
6	128.8	7.06 (1H, t, 7.4)
7	110.5	6.66 (1H, d, 7.4)
8	150.3	–
9	134.1[b]	–
10	38.2	2.23, 2.12 (each 1H, m)
11	42.2	2.94, 2.50 (each 1H, m)
12	–	5.68 (1H, brs)
13	20.7	1.69 (3H, s)
1'	34.7	2.42, 2.38 (each 1H, m)
2'	120.0	4.96 (1H, t, 7.3)
3'	134.6	–
4'	17.8	1.48 (3H, s)
5'	25.6	1.62 (3H, s)

[a] Not assigned
[b] Detected by HMBC

Selected 2D NMR correlations

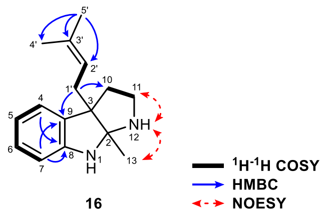

Selected NOESY correlations

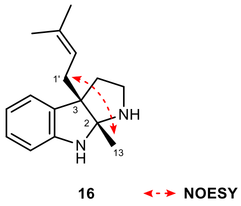

UV spectrum

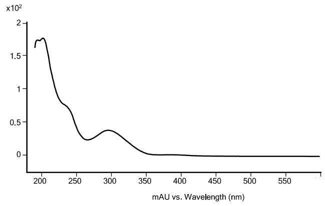

Table S16. Spectroscopic data for **17**.

Compound 17: brown amorphous solid; $[\alpha]_D$ −39 (*c* 0.5, MeOH); UV (MeCN/H_2O/FA, 17:83:0.1) λ_{max} 210, 240, and 314 nm. HRESIMS *m/z* 243.1865 (calcd for $C_{16}H_{22}N_2$: 243.1856, $[M+H]^+$)

	**17** in pyridine-d_5	
position	^{13}C	^1H (*J* in Hz)
2	82.2	5.64 (1H, s)
3	58.5	–
4	124.3	7.01 (1H, s)
5	127.9	–
6	129.2	6.91 (1H, d, 7.8)
7	109.1	6.76 (1H, d, 7.8)
8	148.7	–
9	133.3	–
10	38.6	2.43, 2.16 (each 1H, m)
11	44.0	3.50, 2.97 (each 1H, m)
12	–	–
13	20.9	2.23 (3H, s)
1'	37.0	2.69, 2.62 (each 1H, m)
2'	120.3	5.26 (1H, t, 7.1)
3'	134.6	–
4'	18.1	1.54 (3H, s)
5'	25.8	1.54 (3H, s)

Selected 2D NMR correlations

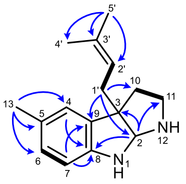

17

^1H-^1H COSY
HMBC

Selected ROESY correlations

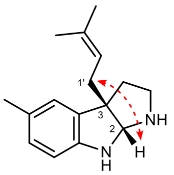

17 ROESY

UV spectrum

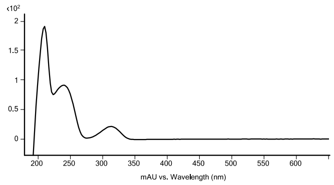

Table S17. Spectroscopic data for (–)-deoxypseudophrynaminol (**18**).

Compound 18: brown amorphous solid; $[\alpha]_D$ –42 (*c* 1.0, $CHCl_3$); UV ($MeCN/H_2O$/FA, 17:83:0.1) λ_{max} 209, 246, and 314 nm. HRESIMS *m/z* 243.1864 (calcd for $C_{16}H_{22}N_2$: 243.1854, $[M+H]^+$)

	**18** in chloroform-*d*	
position	^{13}C	^{1}H (*J* in Hz)
2	86.2	5.27 (1H, s)
3	58.4	–
4	123.1	7.07 (1H, d, 7.8)
5	120.5	6.83 (1H, t, 7.8)
6	128.8	7.11 (1H, t, 7.8)
7	109.9	6.73 (1H, d, 7.8)
8	148.4	–
9	132.3	–
10	36.7	2.50 (1H, m)
		2.19 (1H, dd, 12.9, 5.1)
11	52.2	3.42, 2.63 (each 1H, m)
12	–	–
13	34.5	2.74 (3H, s)
1'	36.9	2.58, 2.52 (each 1H, m)
2'	118.2	5.00 (1H, t, 6.9)
3'	136.1	–
4'	18.2	1.55 (3H, s)
5'	25.9	1.62 (3H, s)

Selected 2D NMR correlations

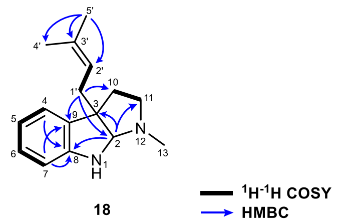

18

Selected NOESY correlations

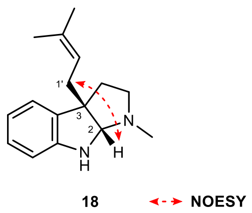

18

UV spectrum

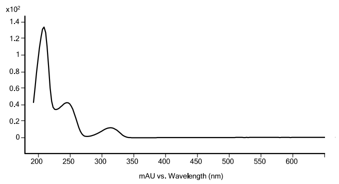

Table S18. Spectroscopic data for **19**.

Compound 19: brown amorphous solid; $[\alpha]_D$ −44 (*c* 0.05, $CHCl_3$); UV (MeCN/H_2O/FA, 80:20:0.1) λ_{max} 205, 240, and 314 nm. HRESIMS *m/z* 243.1867 (calcd for $C_{16}H_{22}N_2$: 243.1856, $[M+H]^+$)

	**19** in chloroform-*d*	
position	^{13}C	^{1}H (*J* in Hz)
2	76.1	4.67 (1H, s)
3	48.6	–
4	122.8	6.98 (1H, d, 7.7)
5	119.7	6.80 (1H, t, 7.7)
6	128.6	7.10 (1H, t, 7.7)
7	110.4	6.72 (1H, d, 7.7)
8	147.2	–
9	130.3	–
10	26.9	2.18, 1.83 (each 1H, m)
11	18.6	1.62, 1.53 (each 1H, m)
12	38.9	3.08 (1H, m)
		2.77 (1H, td, 11.5, 4.2)
1'	37.7	2.58, 2.52 (each 1H, m)
2'	117.9	5.00 (1H, t, 6.9)
3'	136.4	–
4'	17.8	1.55 (3H, s)
5'	26.0	1.62 (3H, s)

Selected 2D NMR correlations

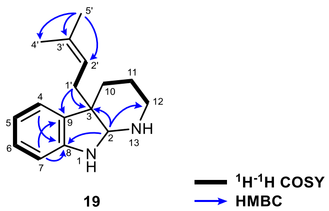

Selected NOESY correlations

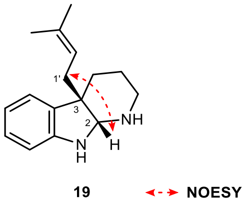

UV spectrum

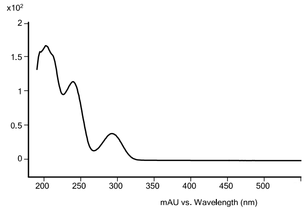

Table S19. Spectroscopic data for **20**.

Compound 20: brown amorphous solid; $[\alpha]_D$ −12 (*c* 0.05, MeOH); UV (MeCN/H_2O/FA, 22:78:0.1) λ_{max} 252 nm. HRESIMS *m/z* 245.1649 (calcd for : $C_{15}H_{21}N_2O$ 245.1648, $[M+H]^+$)

	**20** in methanol-d_4	
position	^{13}C	^1H (*J* in Hz)
1	–	–
2	182.7	–
3	53.0	–
4	124.5	7.25 (1H, d, 7.6)
5	123.7	7.07 (1H, t, 7.6)
6	129.7	7.25 (1H, t, 7.6)
7	111.1	6.91 (1H, d, 7.6)
8	143.1	–
9	132.3	–
10	34.7	2.17 (1H, m)
		1.97 (1H, m)
11	36.8	2.64 (1H, m)
		2.52 (1H, m)
12	–	–
1'	37.1	2.53 (2H, d, 7.6)
2'	118.2	4.79 (1H, t, 7.6)
3'	137.0	–
4'	26.0	1.54 (3H, s)
5'	18.1	1.50 (3H, s)

Selected 2D NMR correlations

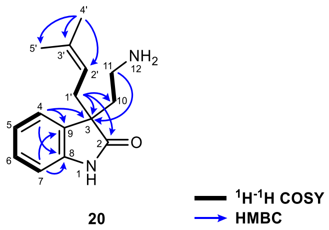

20

UV spectrum

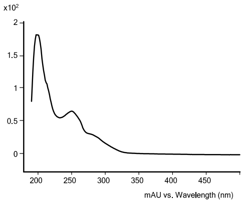

Table S20. Spectroscopic data for **22**.

Compound 22: brown amorphous solid; $[\alpha]_D$ −14 (*c* 0.2, MeOH); UV (MeCN/H_2O/FA, 15:85:0.1) λ_{max} 247 nm. HRESIMS *m/z* 275.1388 (calcd for : $C_{15}H_{19}N_2O_3$ 275.1390, $[M+H]^+$)

	**22** in methanol-d_4	
position	^{13}C	^1H (*J* in Hz)
1	–	–
2	183.2[a]	–
3	52.3	–
4	124.8	7.25 (1H, d, 7.5)
5	123.8	7.07 (1H, t, 7.5)
6	129.8	7.25 (1H, t, 7.5)
7	111.2	6.93 (1H, d, 7.5)
8	142.8	–
9	132.3	–
10	34.5	2.20 (1H, m)
		2.11 (1H, dt, 14.7, 7.3)
11	36.7	2.82 (1H, m)
		2.56 (1H, dt, 13.0, 6.4)
12	–	–
1'	37.8	3.01, 2.85 (each 1H, m)
2'	129.4	5.36 (1H, brs)
3'	136.4 [a]	–
4'	21.4	1.77 (3H, s)
5'	174.2 [a]	–

[a] Detected by HMBC

Selected 2D NMR correlations

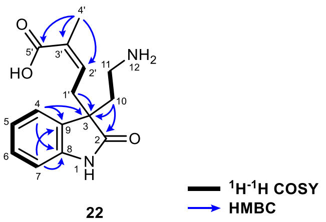

22

Selected NOESY correlations

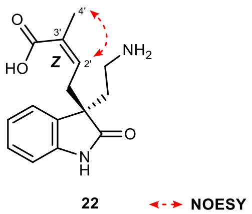

22

UV spectrum

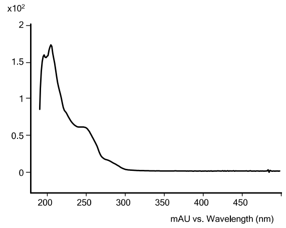

Supplementary Figures

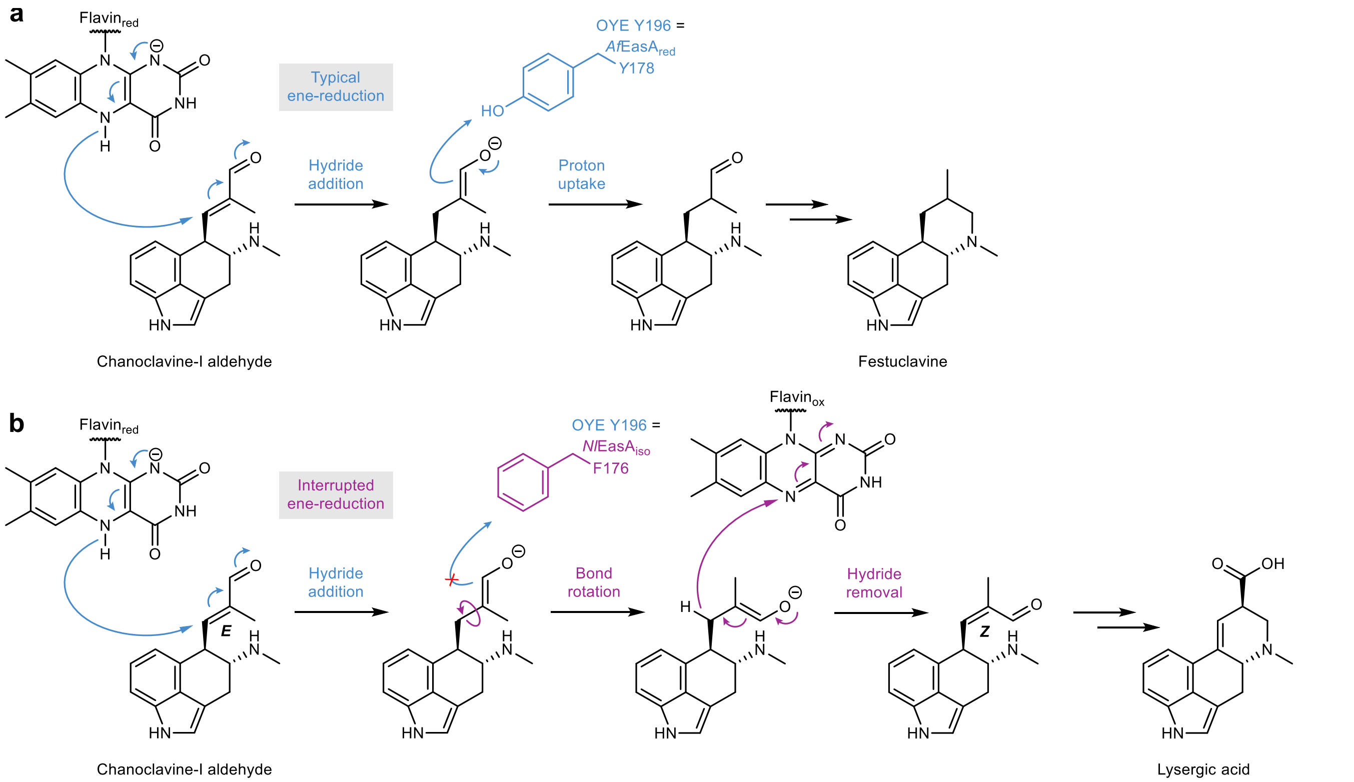

Fig. S1. The role of EasA in ergot alkaloid lysergic acid biosynthesis. a, In the festuclavine branch of ergot alkaloids, EasA$_{red}$ (ie. *Aspergillus fumigatus* EasA or *Af*EasA) functions as a canonical reductase, performing a typical ene-reduction reaction. **b,** In the lysergic acid branch (also referred to as agroclavine branch) of ergot alkaloids, EasA$_{iso}$ (ie. *Neotyphodium lolii* EasA or *Nl*EasA) functions as an *E*-to-*Z* isomerase. Following hydride addition to the chanoclavine-I aldehyde, the resulting enolate undergoes sigma bond rotation in the active site and transfer of the hydride back to the flavin, resulting in *Z*-alkene geometry. Mutation of the canonical tyrosine results in interruption of the proton uptake step. This allows for intramolecular formation of a cyclic iminium product, which is reduced and subsequently converted to lysergic acid.

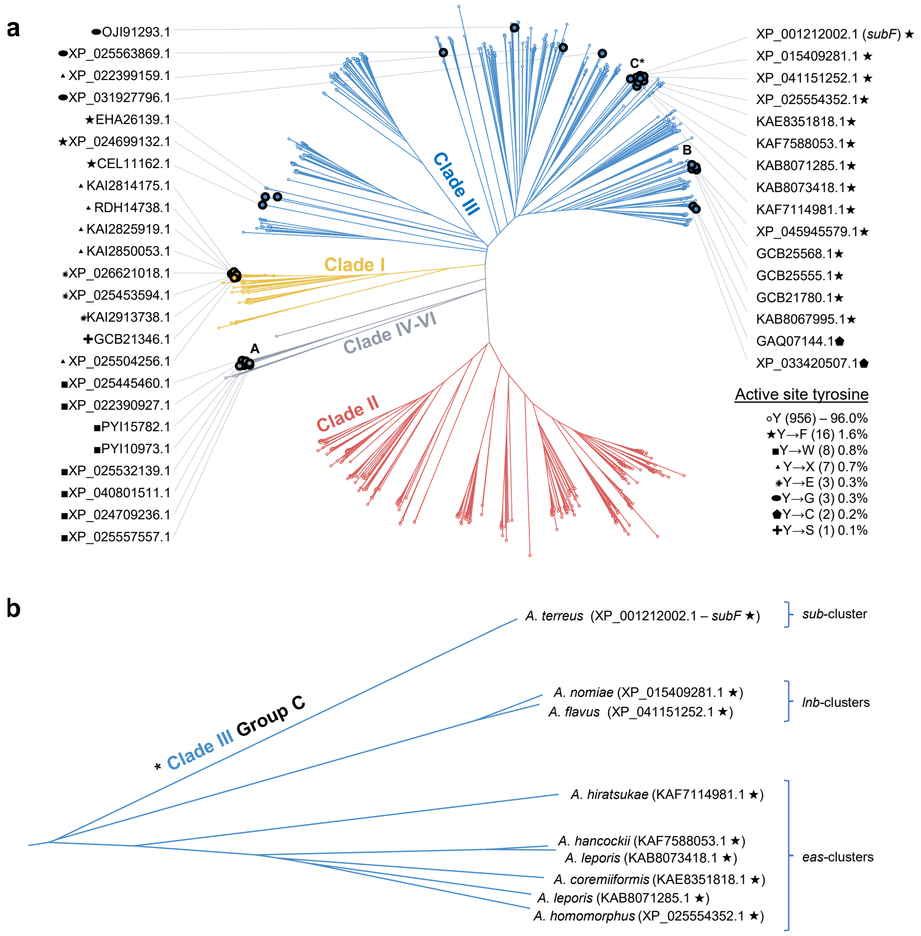

Fig. S2. Phylogenetic tree of *Aspergillus* OYEs. a, Tree was constructed using Clustal Omega (1.2.4) multiple sequence alignment and visualized using iTOL. Analysis of OYE Groups A–C (where a Group is defined as two or more closely related predicted noncanonical-OYEs sharing biosynthetic machinery) is shown in Fig. S1–S3. Among the 1000 hits analyzed, 40 OYE homologs (4.0%) contain a residue other than tyrosine at the conserved position, a subcollection that was designated as predicted noncanonical-OYEs (ψOYEs). Predicted ψOYEs were distributed throughout the different OYE clades (with the exception of Class II, classical OYEs, which strictly possessed active site tyrosines). Two of the three ψOYE BGC groups (Group A and B, Figs. S1 and S2) are not co-clustered with core-forming biosynthetic enzymes – polyketide synthase (PKS), nonribosomal peptide synthetase (NRPS), and terpene synthase (TS). (Note: all of the Clade I tyrosine mutations are derived from *A. niger* isolates, and occur on identical or highly syntenuous contigs, and are not defined as a Group). **b,** Expanded view of Group C (which includes the *sub* BGC) and corresponding *Aspergillus* species.

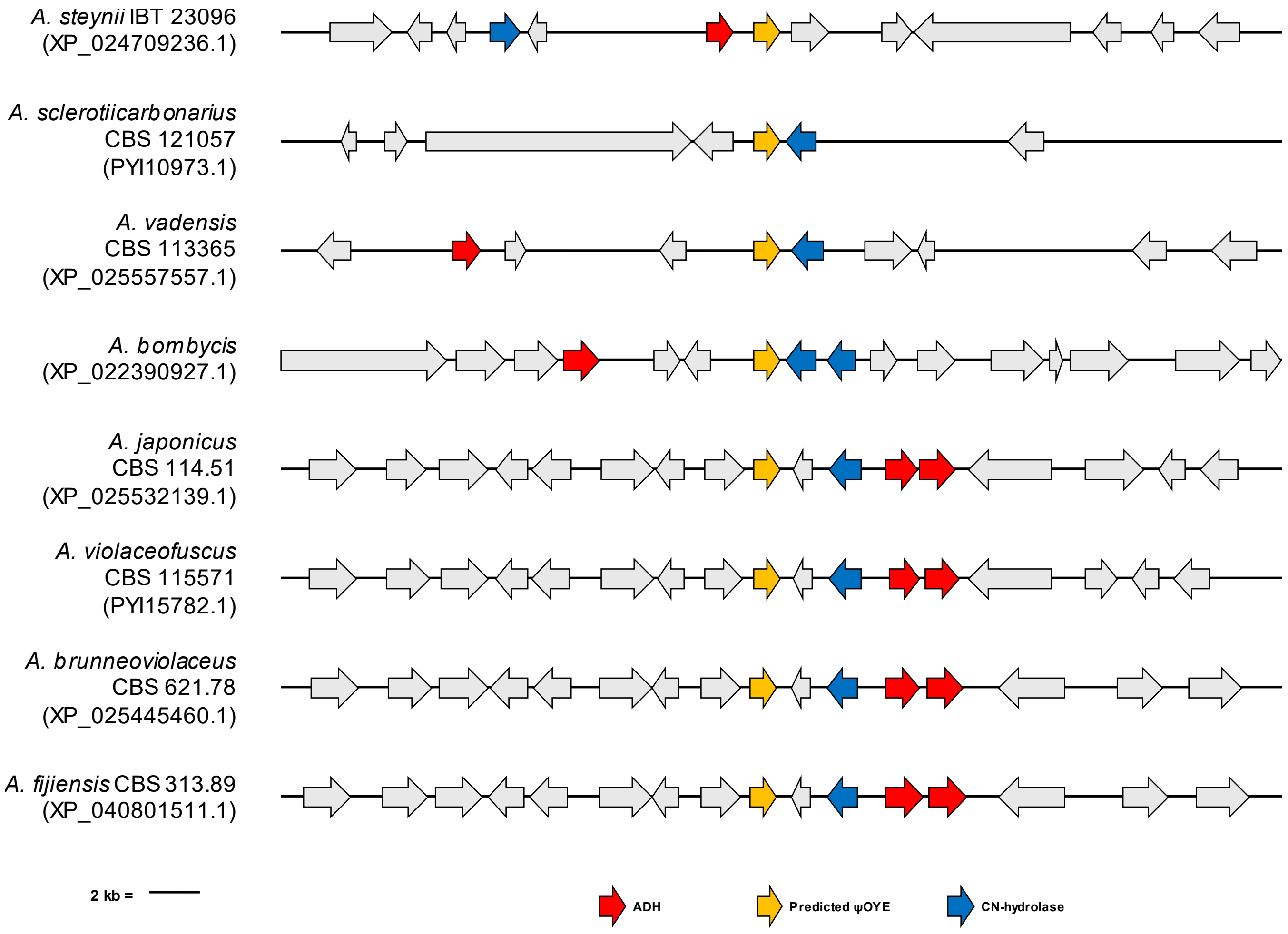

Fig. S3. *Aspergillus* ψOYEs - Group A. In addition to OYE (old yellow enzyme), shared biosynthetic machinery includes CN(carbon-nitrogen)-hydrolase, and ADH (alcohol dehydrogenase). Additional homologous clusters are present in non-*Aspergillus* genera.

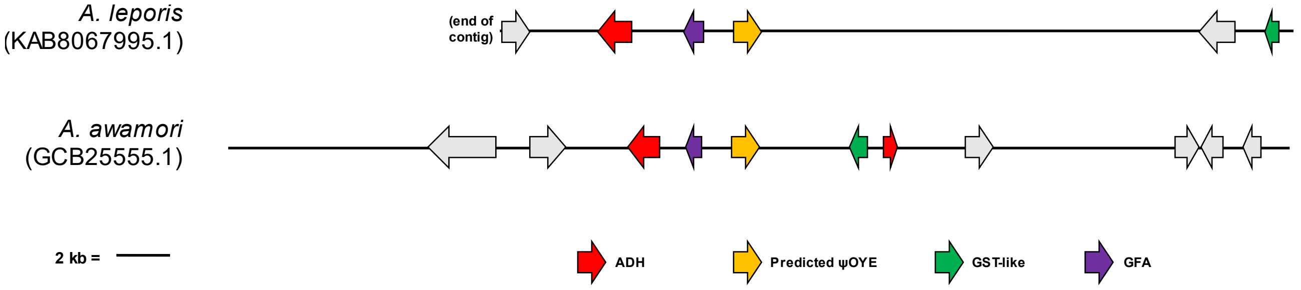

Fig. S4. ***Aspergillus*** **ψOYEs - Group B.** In addition to OYE (old yellow enzyme), shared biosynthetic machinery includes GFA (glutathione-dependent formaldehyde-activating enzyme), GST (glutathione *S*-transferase)-like, and ADH (alcohol dehydrogenase). Additional homologous clusters are present in non-*Aspergillus* genera.

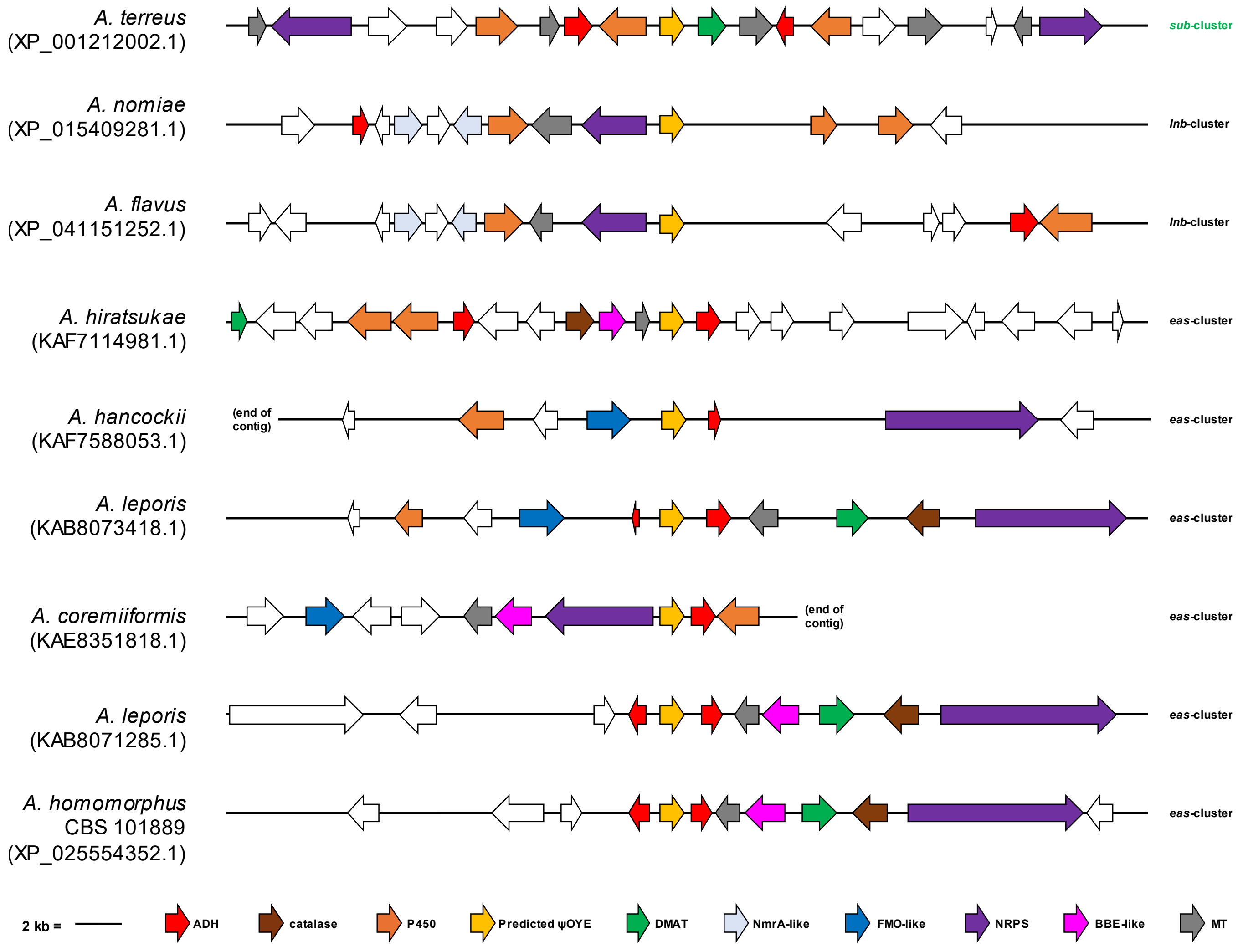

Fig. S5. *Aspergillus* ψOYEs - Group C. In addition to OYE (old yellow enzyme), shared biosynthetic machinery includes ADH (alcohol dehydrogenase), catalase, P450, DMATS (dimethylallyltryptophan synthase), NmrA-like, FMO (flavin-containing monooxygenase)-like, NRPS (non-ribosomal peptide synthase), BBE (berberine bridge enzyme)-like, and MT (methyltransferase). Additional homologous clusters are present in non-*Aspergillus* genera.

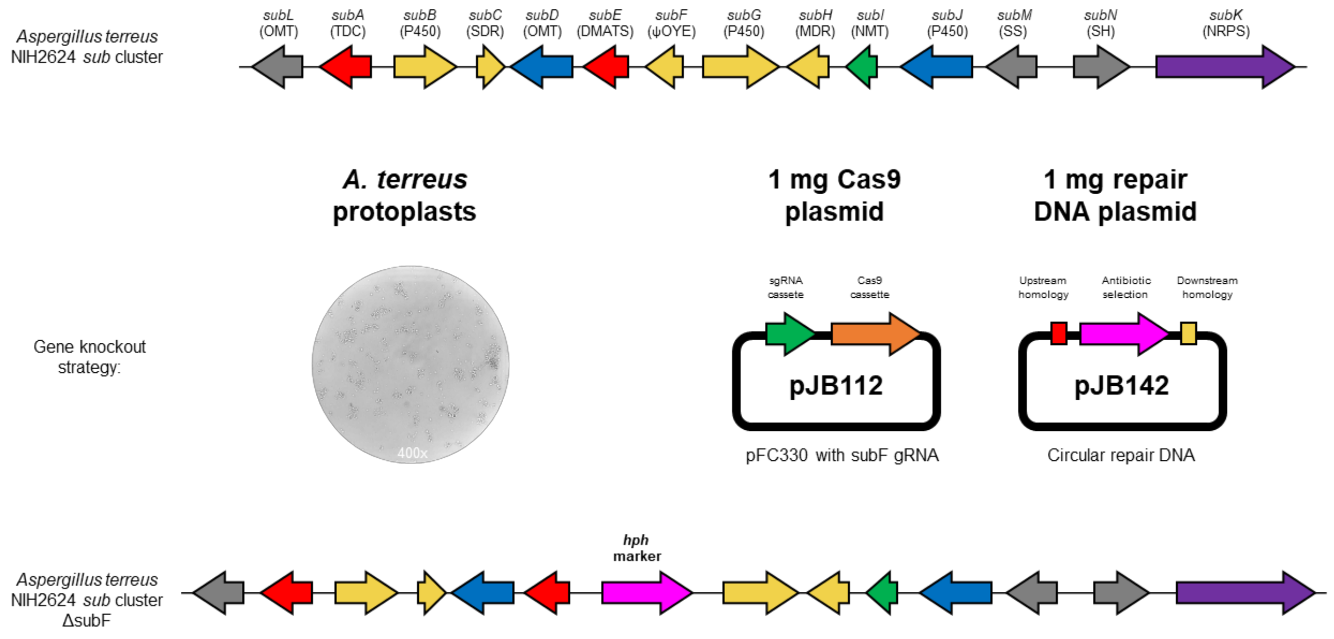

Fig. S6. Deletion of sub cluster from *A. terreus*. *A. terreus* locus architecture shown before and after Cas9-mediated *subF* replacement with the hygromycin resistance expression cassette (*hph*). Gene knockout strategy is depicted with image of generated *A. terreus* protoplasts and schematic of plasmids. OMT, O-methyltransferase; SDR, short-chain dehydrogenase/reductase; DMATS, dimethylallyl tryptophan synthase; OYE, old yellow enzyme; MDR, medium-chain dehydrogenase/reductase; NMT, N-methyltransferase; SS, salicylate synthase; SH, salicylate hydroxylase; NRPS, non-ribosomal peptide synthetase.

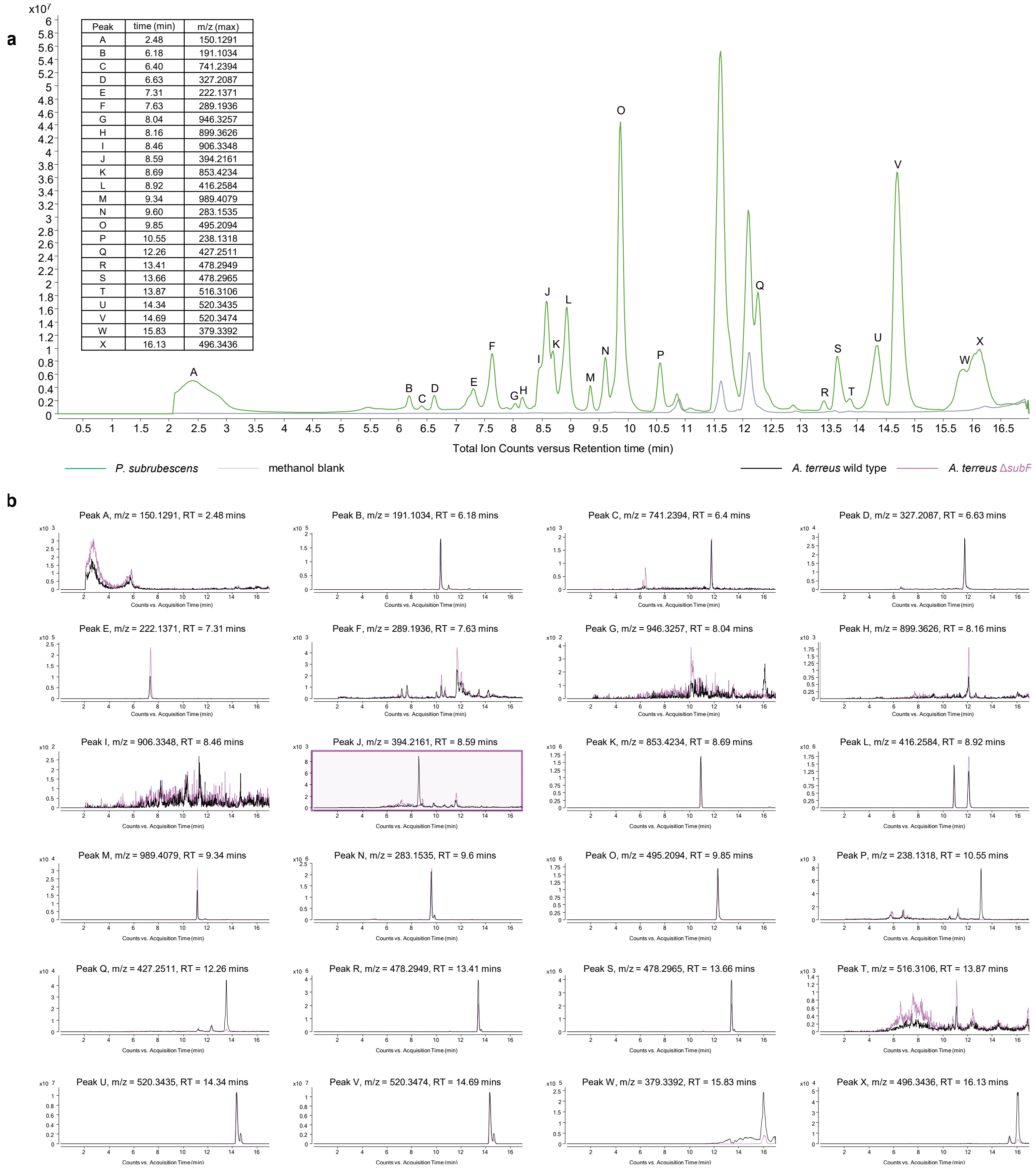

Peak	time (min)	m/z (max)
A	2.48	150.1291
B	6.18	191.1034
C	6.40	741.2394
D	6.63	327.2087
E	7.31	222.1371
F	7.63	289.1936
G	8.04	946.3257
H	8.16	899.3626
I	8.46	906.3348
J	8.59	394.2161
K	8.69	853.4234
L	8.92	416.2584
M	9.34	989.4079
N	9.60	283.1535
O	9.85	495.2094
P	10.55	238.1318
Q	12.26	427.2511
R	13.41	478.2949
S	13.66	478.2965
T	13.87	516.3106
U	14.34	520.3435
V	14.69	520.3474
W	15.83	379.3392
X	16.13	496.3436

Fig. S7. Identification of *sub* gene cluster product via comparative metabolomics. a, Metabolite profile of *P. subrubescens* grown on CD agar by LC-MS. All discernable peaks from the TIC chromatogram were extracted and the m/z (max) was recorded (see table). The grey trace is a methanol blank, with resulting peaks corresponding to background ions present in all samples. **b,** The m/z hits were used to filter TIC of the wild-type *A. terreus* (black) and *A. terreus ΔsubF* (purple). Resulting EIC were inspected for peaks with m/z and retention times matching *P. subrubescens* chromatogram profile. Peak J, which had m/z = ~394, and retention time = ~8.6 mins was present in both wild-type strains, but absent from the *A. terreus subF* knockout.

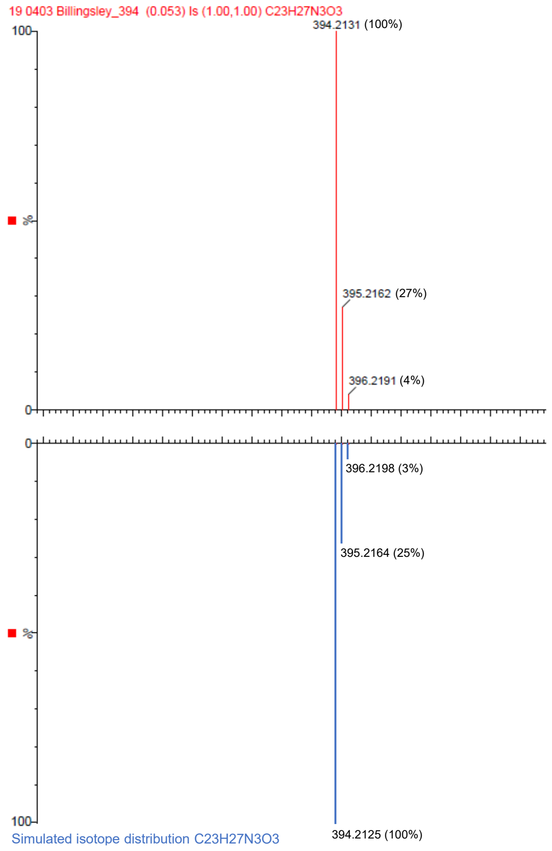

Fig. S8. Prediction of molecular formula of subrubine A (1) via HR-MS. The predicted formula of $C_{23}H_{27}N_3O_3$ hinted at an amino acid origin. Simulated isotope distribution (exact mass and abundance) is shown in blue and matched the formula predicted by high-resolution mass spectrometry.

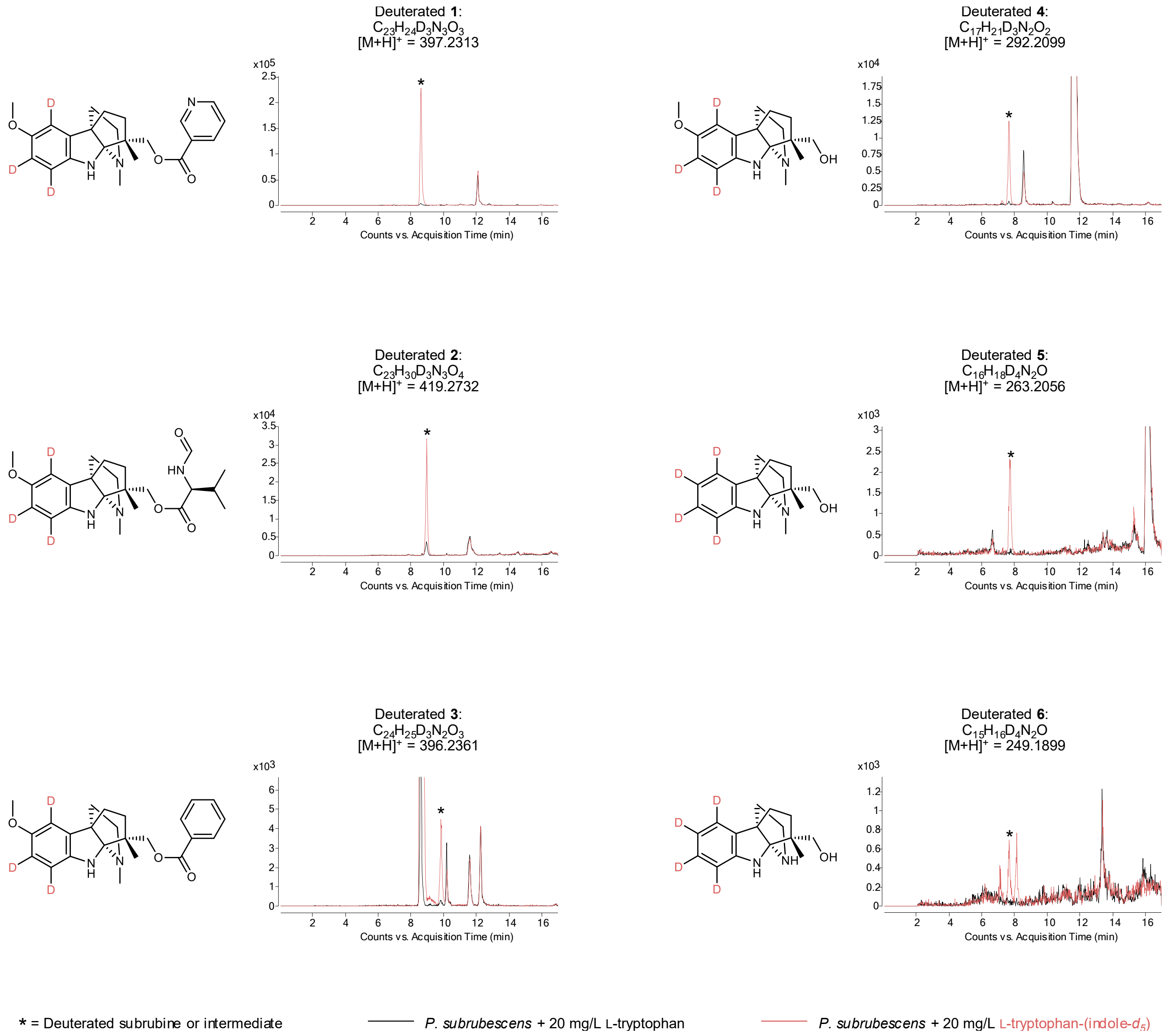

Fig. S9. Incorporation of L-tryptophan-(*indole*-d5) into subrubines. Tryptophan related genes in the cluster (TDC, DMATS) prompted feeding of deuterated (red) and non-deuterated (black) L-tryptophan. Inspection of mass spectra for isotope incorporation revealed six metabolites (**1–6**) with increased m/z values in cultures supplemented with L-tryptophan-(*indole*-d5), which confirmed the amino acid origin of the compounds.

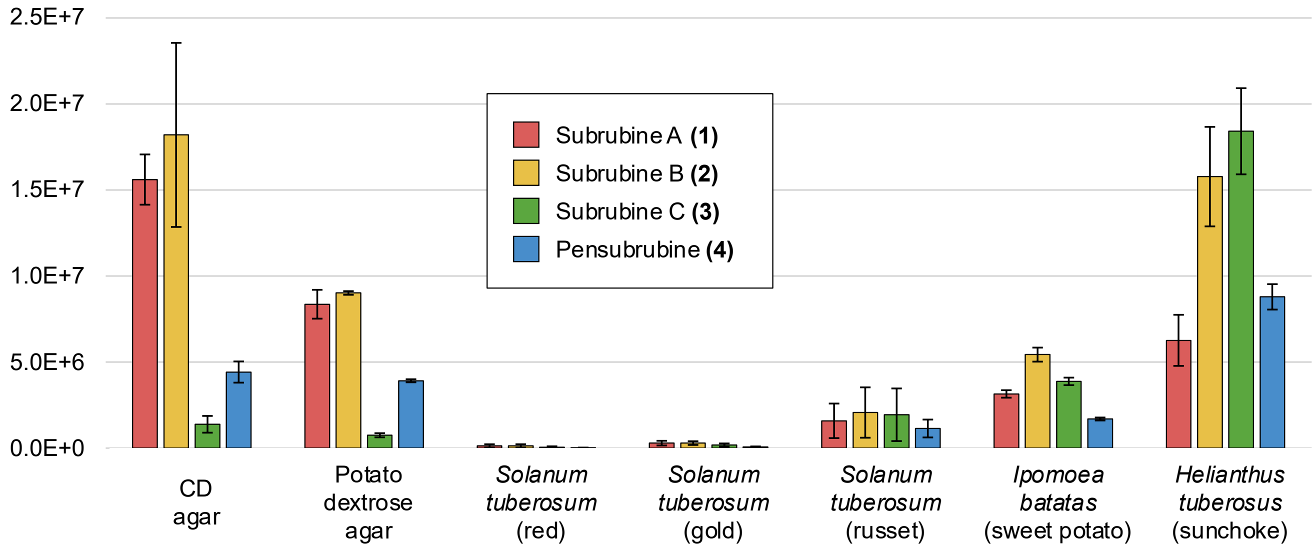

Fig. S10. Production of subrubines by *P. subrubescens* on a variety of fungal growth media. TIC values of four abundantly produced *subF*-associated metabolites. It was noted that growth on *H. tuberosus* (sunchokes), the plant from which *P. subrubescens* was isolated, resulted in the highest production of subrubines on a TIC per kg media basis as well as TIC per cost media basis. Error bars denote standard deviation across n = 3 biological replicates.

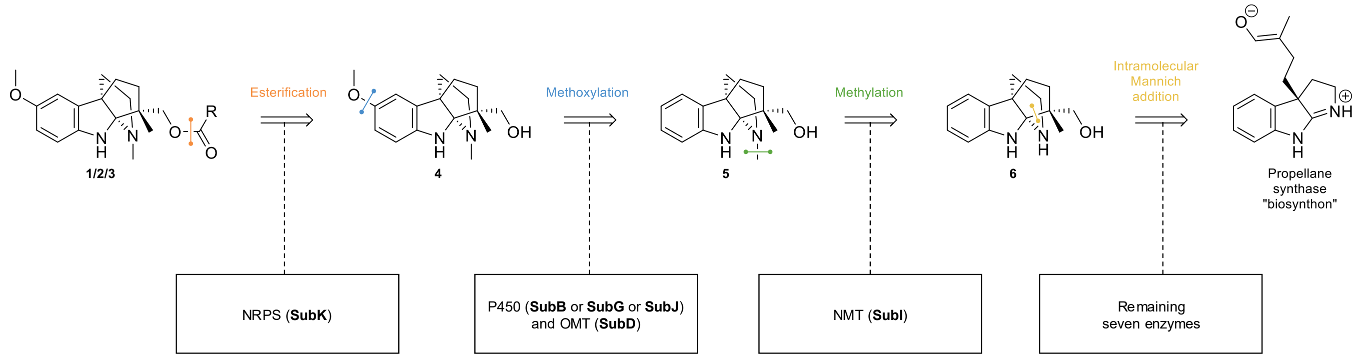

Fig. S11. Retrobiosynthetic analysis of subrubines. The function of 4/11 of the enzymes in the BGC could be speculated *a priori* based on isolated and predicted intermediates. SubK likely esterifies **4** to make **1/2/3**. Ring methoxylation was predicted to occur through the action of a P450 and the *O*-methyltransferase SubD. We also predicted that SubI was responsible for *N*-methylation to produce **5** from **6**. The remaining seven enzymes were therefore predicted to be responsible for **6** production from L-tryptophan.

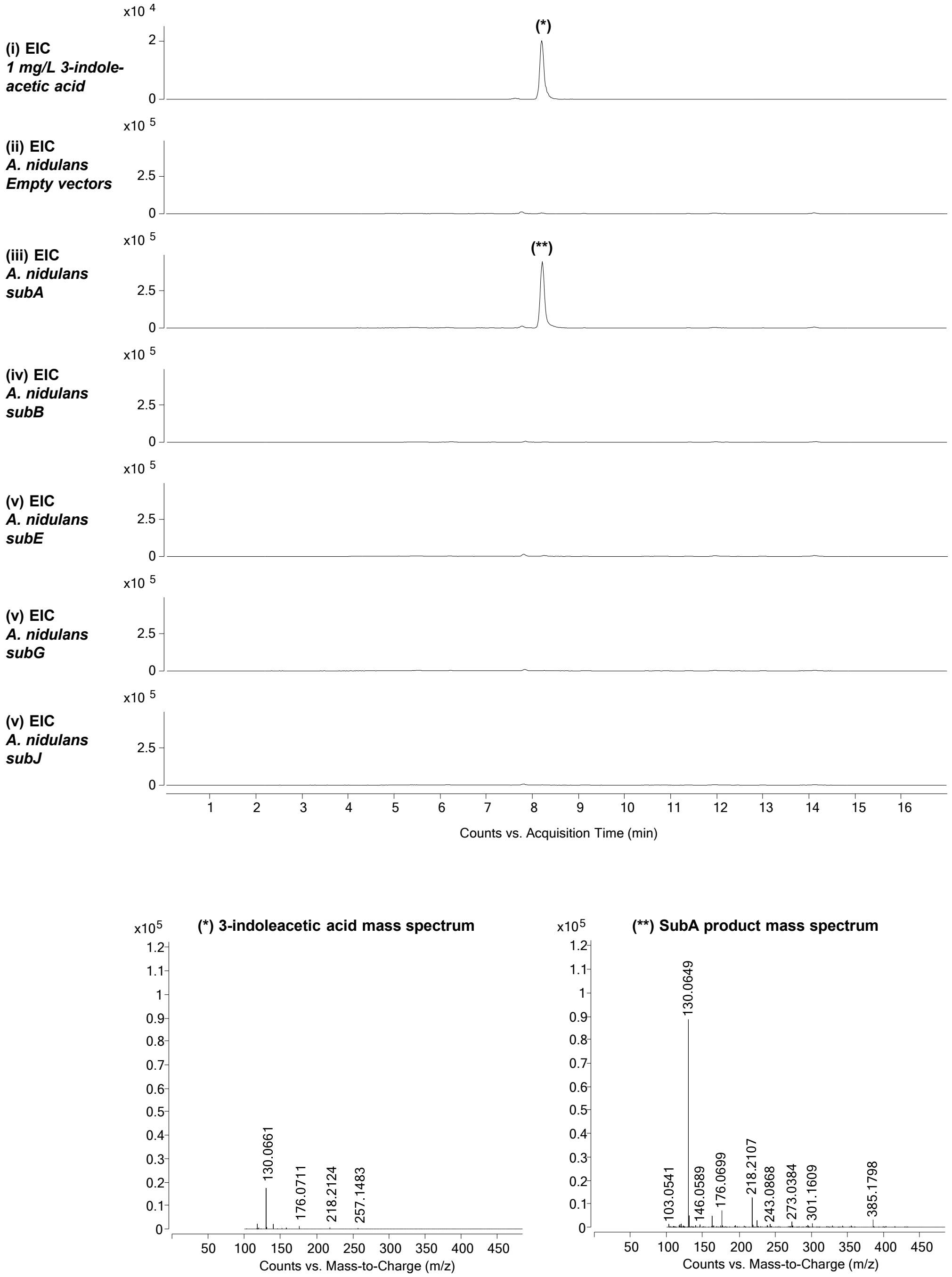

Fig. S12. *In vivo* characterization of *subA*. Reconstitution of early subrubine biosynthesis, in which *subA, subB, subE, subG,* and *subJ* were individually expressed in *A. nidulans*. LC-MS chromatograms of potential L-tryptophan derived metabolites were inspected. Accumulation of a metabolite in *subA* expressing strains had a retention time and exact mass matching 3-indole-acetic acid. EIC (merged): 130.0651, 144.0808, 159.0917, 161.1073, 174.055, 176.0706, 192.0655, 198.1277, 203.0816, 205.0972, 221.0921, 227.1543, 244.1332, 273.1598 – corresponding to prenylated, decarboxylated, and/or oxidized tryptophan (and corresponding water/ammonia losses).

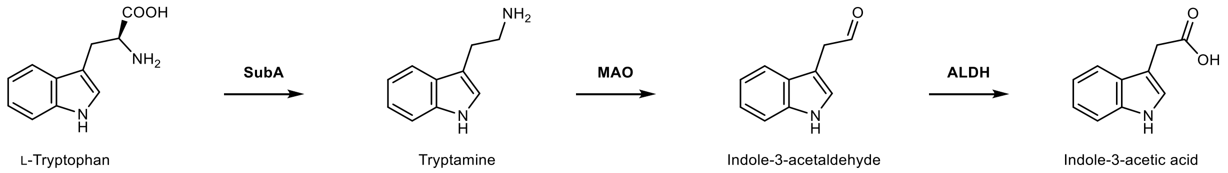

Fig. S13. Proposed formation of indole-3-acetic acid via TAM (tryptamine) pathway. Degradation of tryptamine produced via L-tryptophan has been reported in fungi. After decarboxylation by SubA, tryptamine may be converyed to indole-3-acetaldehyde by MAO (monoamine oxidase). An ALDH (aldehyde dehydrogenase) can then convert the aldehyde into indole-3-acetic acid.

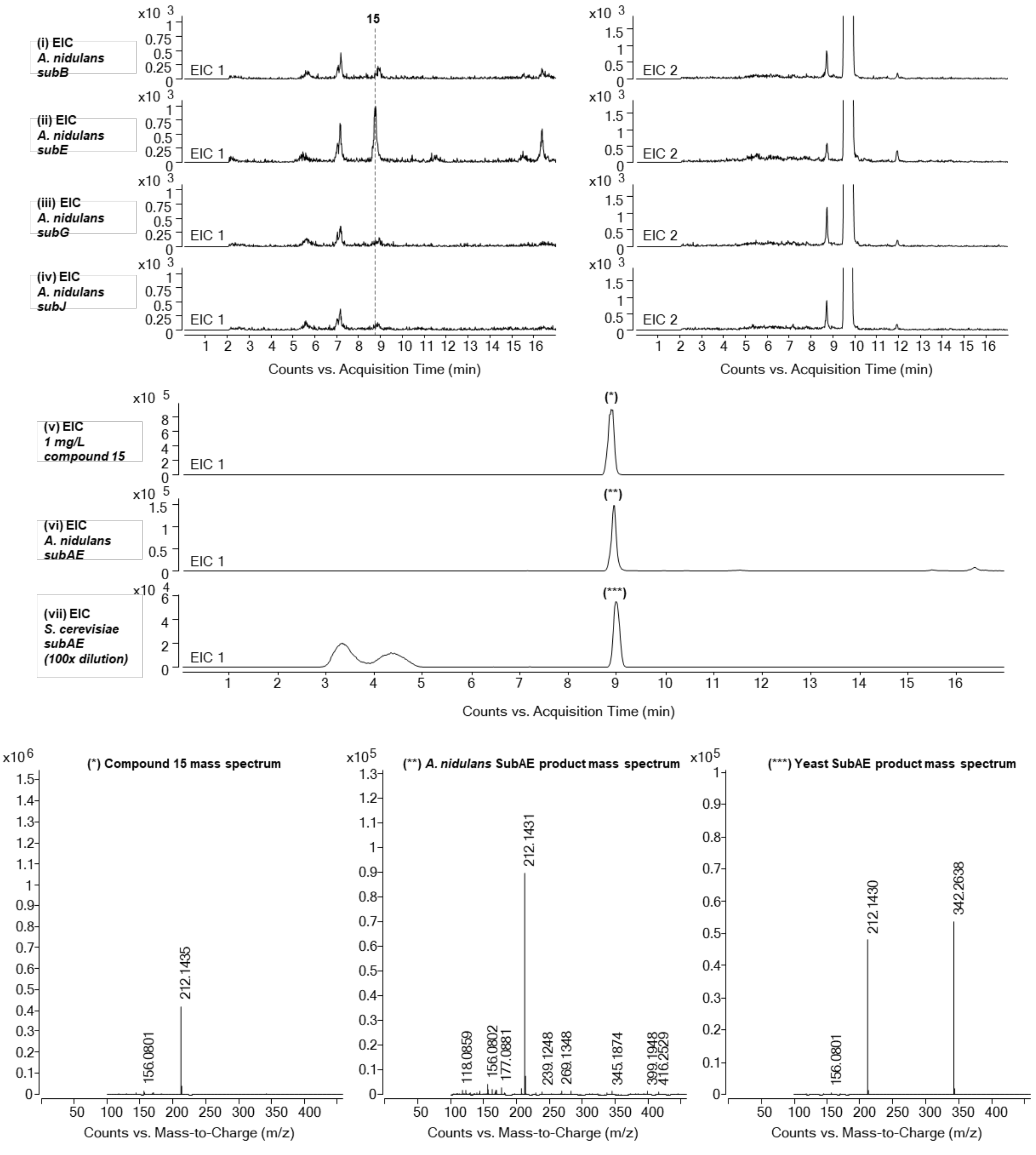

Fig. S14. ***In vivo* characterization of *subAE*.** Reconstitution of early subrubine biosynthesis, in which *subB, subE, subG,* and *subJ* were individually expressed or coexpressed with *subA* in *A. nidulans*. LC-MS chromatograms of potential tryptamine derived metabolites were inspected. Accumulation of a metabolite in *subAE* expressing *A. nidulans* and *S. cerevisiae* strains had a retention time and exact mass matching **15**. EIC 1 (merged): 212.1434, 229.1699 – corresponding to prenylated tryptamine (and corresponding ammonia loss). EIC 2 (merged): 160.0757, 177.1022, 194.1329 – corresponding to oxidized tryptamine (and corresponding water/ammonia losses).

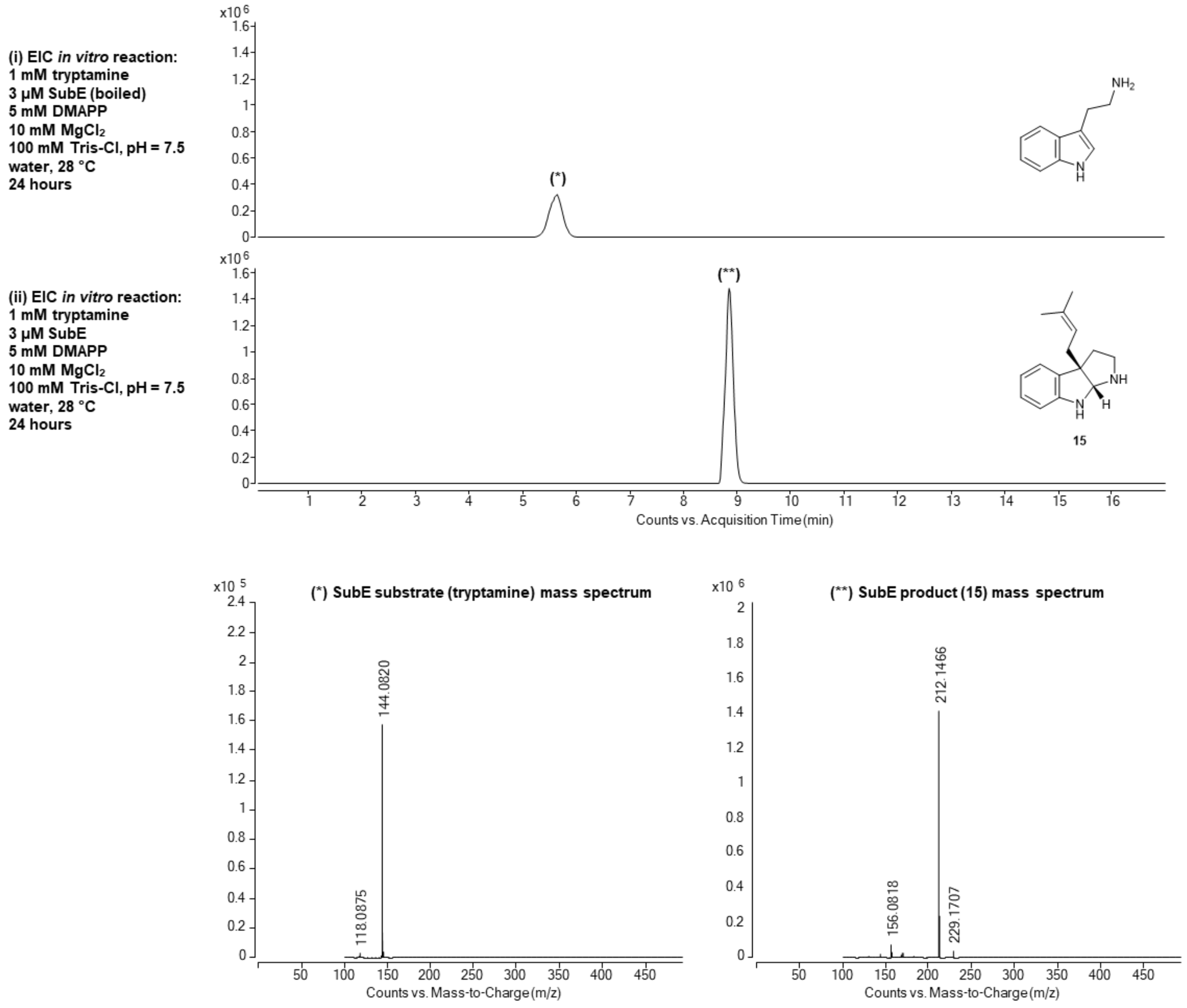

Fig. S15. *In vitro* characterization of SubE. *In vitro* conversion of 1 mM tryptamine to **15** in the presence of 5 mM DMAPP and 3 µM SubE was tested. Complete conversion in 24 hours was observed, no conversion was observed in the boiled SubE control. EIC (merged): 144.0808, 212.1434 – corresponding to the most abundant ion present in tryptamine and **15** mass spectra.

ComX pheromone pathway

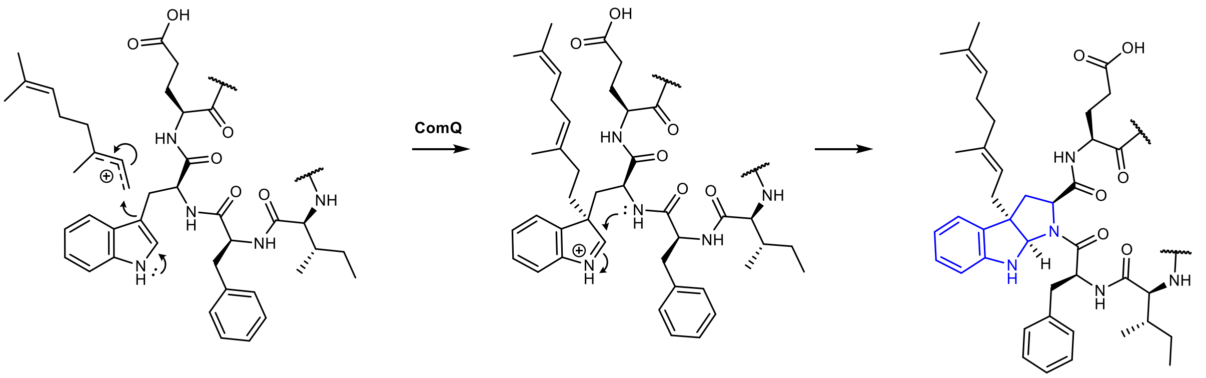

Aszonalenin pathway

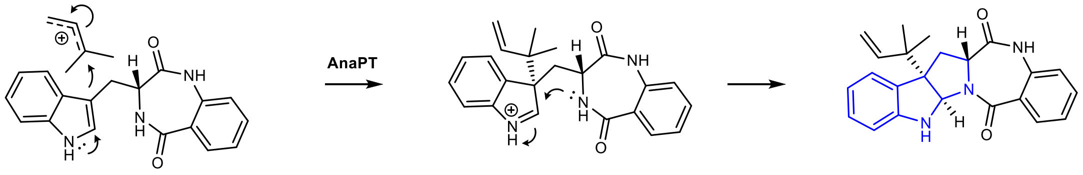

Subrubine pathway

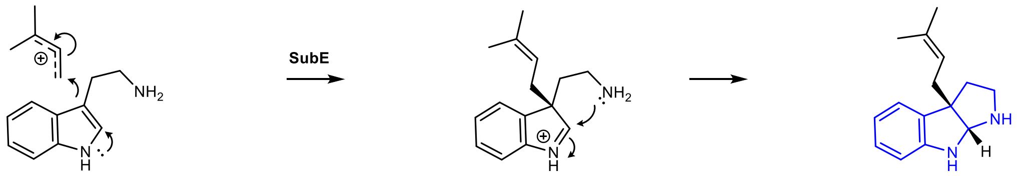

Fig. S16. Pyrrolidinoindoline forming prenyltransferases. Representative examples of DMAPP/GPP-utilizing PFTs are shown. In the absence of additional biochemical evidence of a Cope rearrangement type prenylation mechanism, direct forward and reverse prenylations are depicted. Reported PFTs have been shown to produce pyrrolidinoindolines with both stereochemical outcomes, but employ chiral/advanced biosynthetic intermediates.

Physostigmine pathway

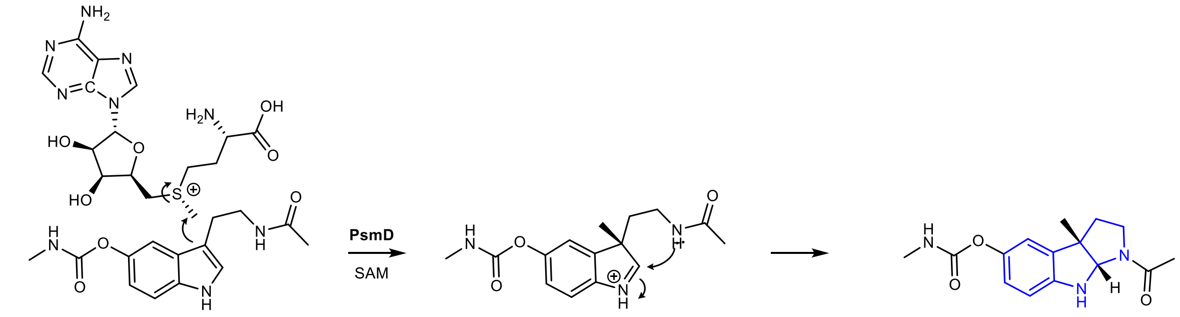

Nocardioazine pathway

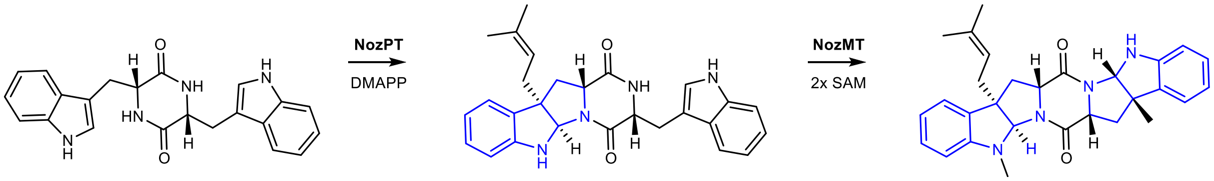

Fig. S17. Pyrrolidinoindoline forming methyltransferases. Representative examples of SAM-utilizing PFTs are shown. Both employ chiral/advanced biosynthetic intermediates. Nocardioazine biosynthesis employs both a DMAPP-utilizing PFT and a SAM-utilizing PFT.

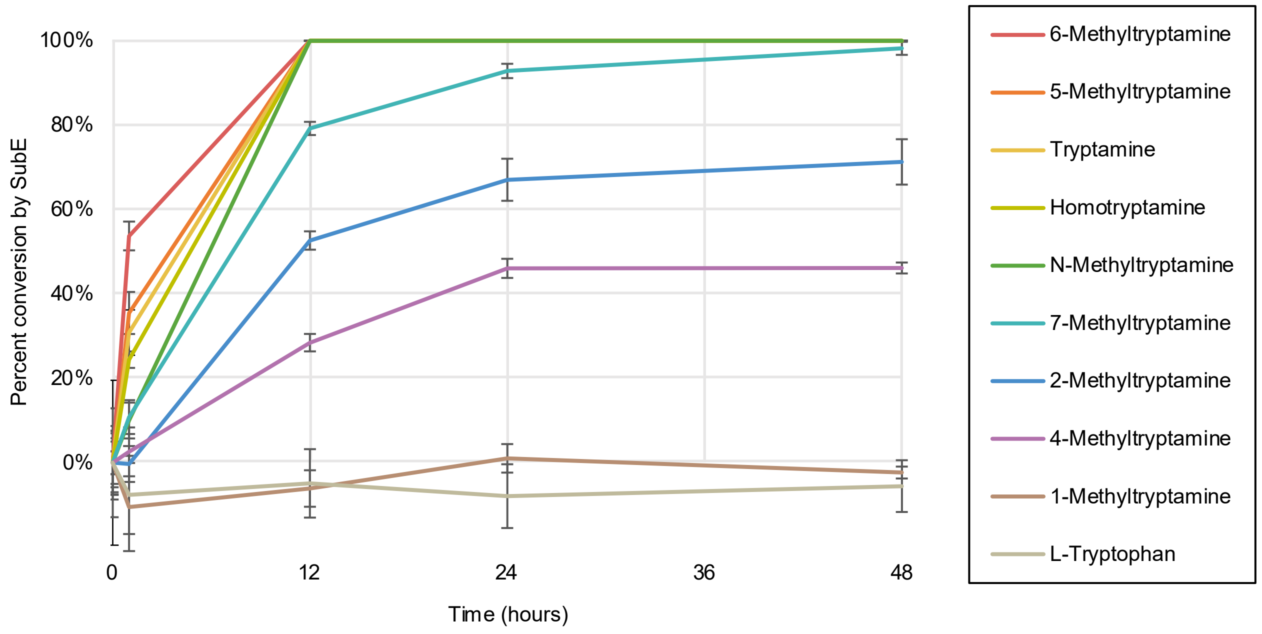

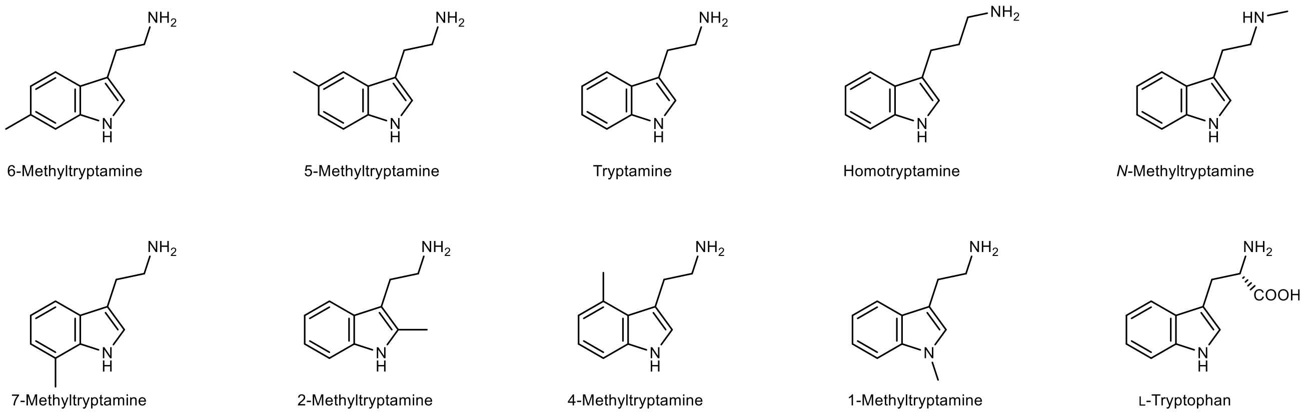

Fig. S18. SubE time course on modified tryptamines. A variety of tryptamine analogs were fed to SubE in the presence of DMAPP to probe its catalytic flexibility. The formation of corresponding methylated and "new-to-nature" **15** analogs was monitored by disappearance of substrate as determined by LC-MS. Four of the seven produced analogs were subsequently isolated and characterized (Fig. 3). Error bars denote standard deviation across n = 3 technical replicates.

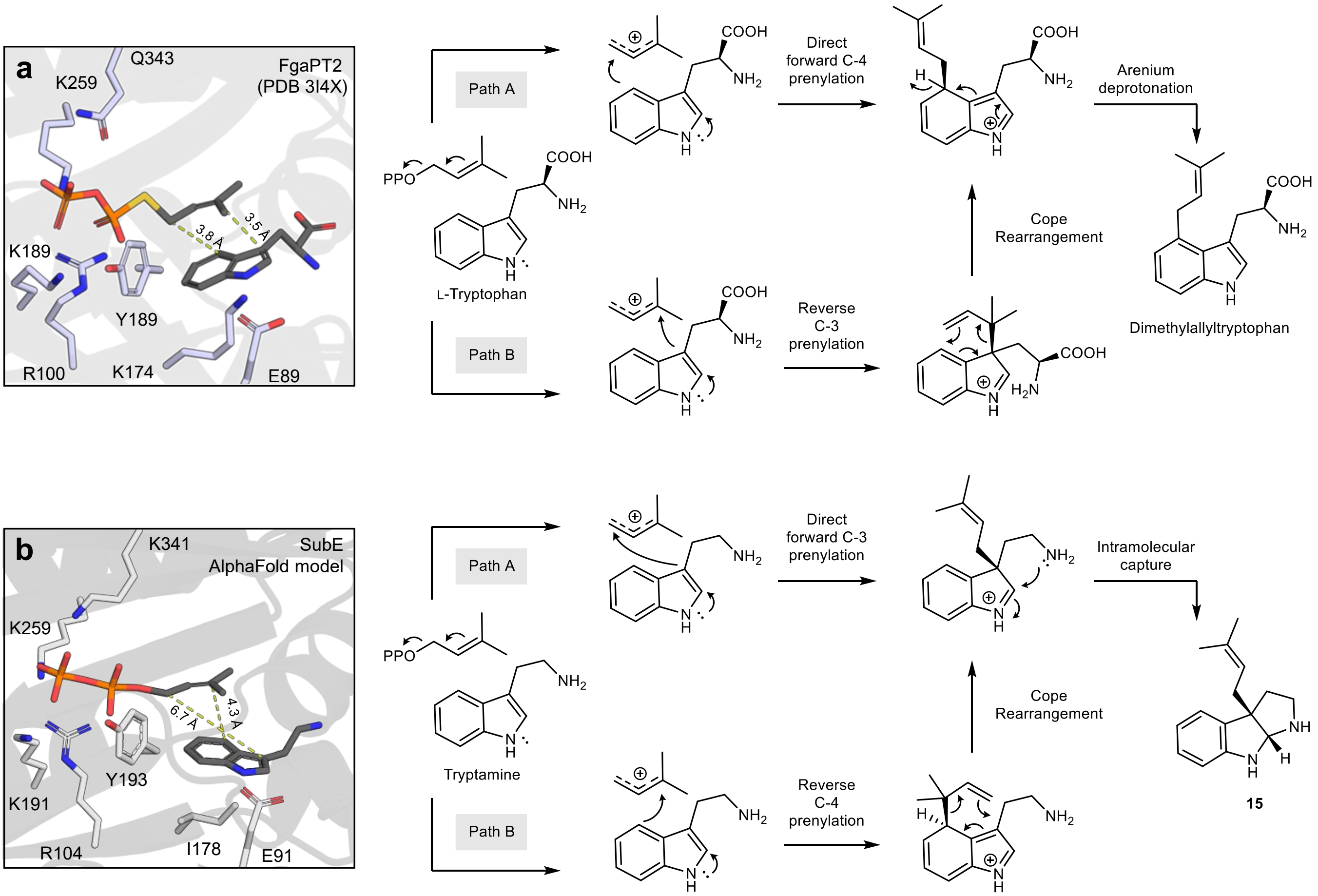

Fig. S19. Comparison of DMATS active sites and potential mechanism of prenylation. a, Crystal structure of FgaPT2, dimethylallyltryptophan synthase, complexed with L-tryptophan and DMASPP is shown. Two routes to dimethylallyltryptophan were previously identified by Tanner. Based on active site geometry, Path A and Path B were deemed equally plausible; mutational studies have provided evidence that Path B involving a Cope rearrangement is the favored route. **b,** AlphaFold model of SubE was predicted; ligands were placed using AlphaFill and SwissDock. Two potential routes to **15** are shown. The longer distance (6.7 Å) from indole C3 to DMAPP C1 and absence of the active site base (I178 SubE vs. K174 in FgaPT2) suggest that Path B may be favored, a possibility that warrants further investigation.

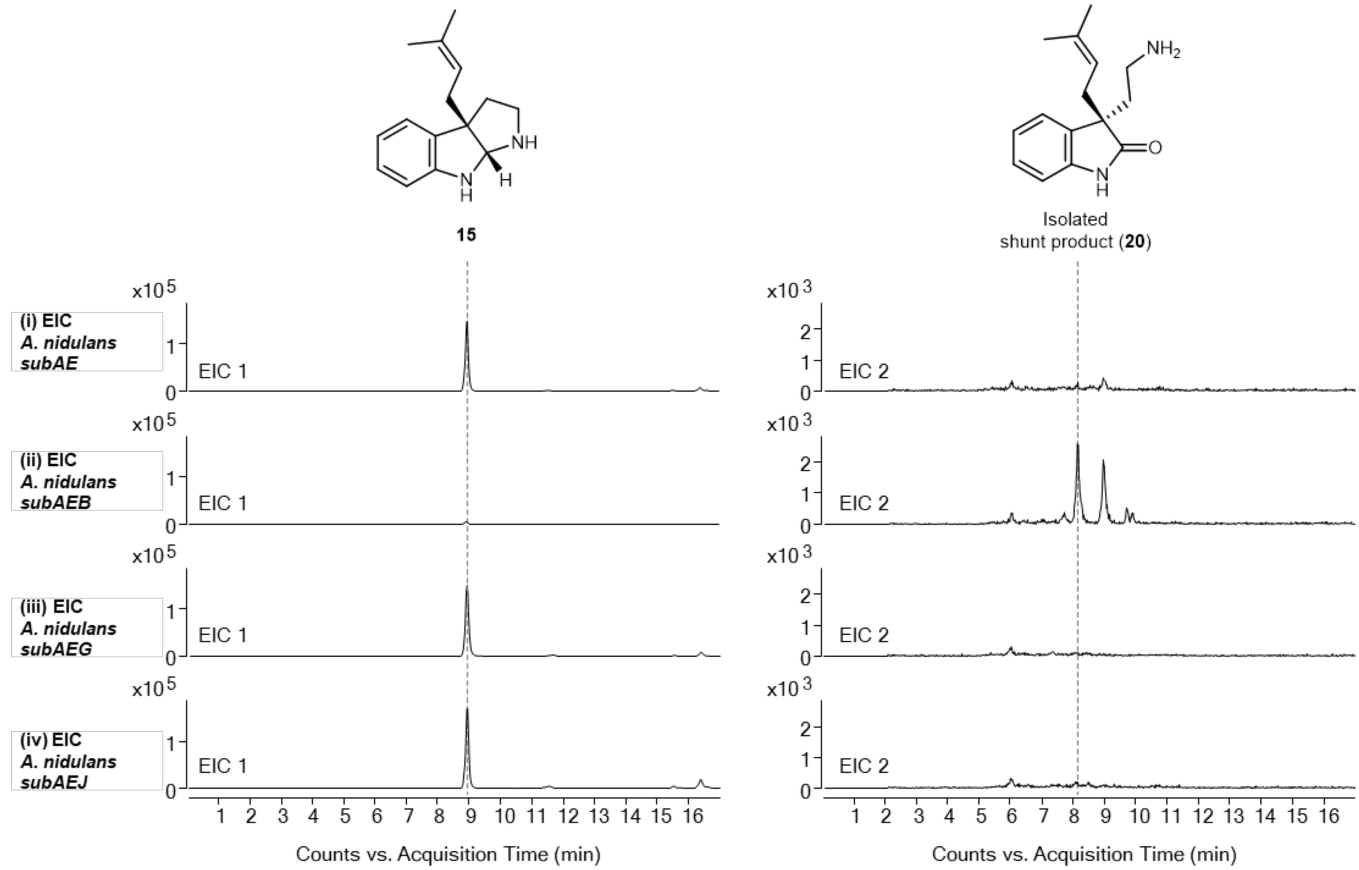

Fig. S20. ***In vivo*** **characterization of** ***A. nidulans*** **expressing** ***subAEB*****.** The **15**-producing *A. nidulans* strain (expressing *subAE*) was compared with strains expressing *subAE* and the clustered P450s (*subB*, *subG*, and *subJ*). Complete disappearance of **15** and concomitant appearance of **20** was observed in the sub*AEB* strain. Isolated compound **20** is presumed to be a “ring-open” oxindole shunt product of oxidized **15**. EIC 1 (merged): 212.1434, 229.1699 – corresponding to **15** (and corresponding ammonia loss). EIC 2 (merged): 227.1543, 228.1383, 245.1648 – corresponding to oxidized **15** (and corresponding hydrate and water/ammonia losses).

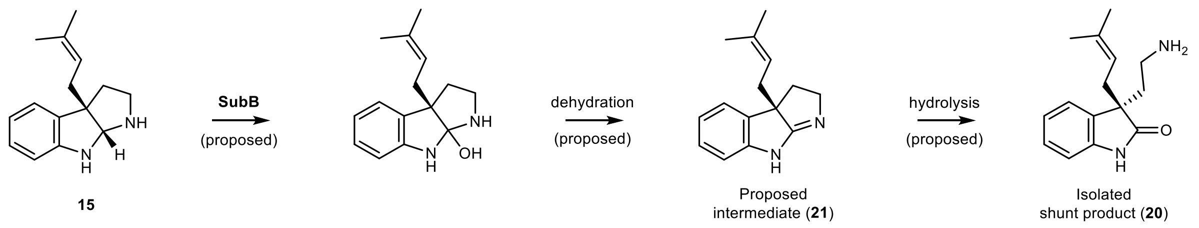

Fig. S21. Proposed formation of shunt product 20. Following P450-mediated hydroxylation of **15**, dehydration yields the imine intermediate **21**, which may readily hydrolyze to the corresponding oxindole **20**.

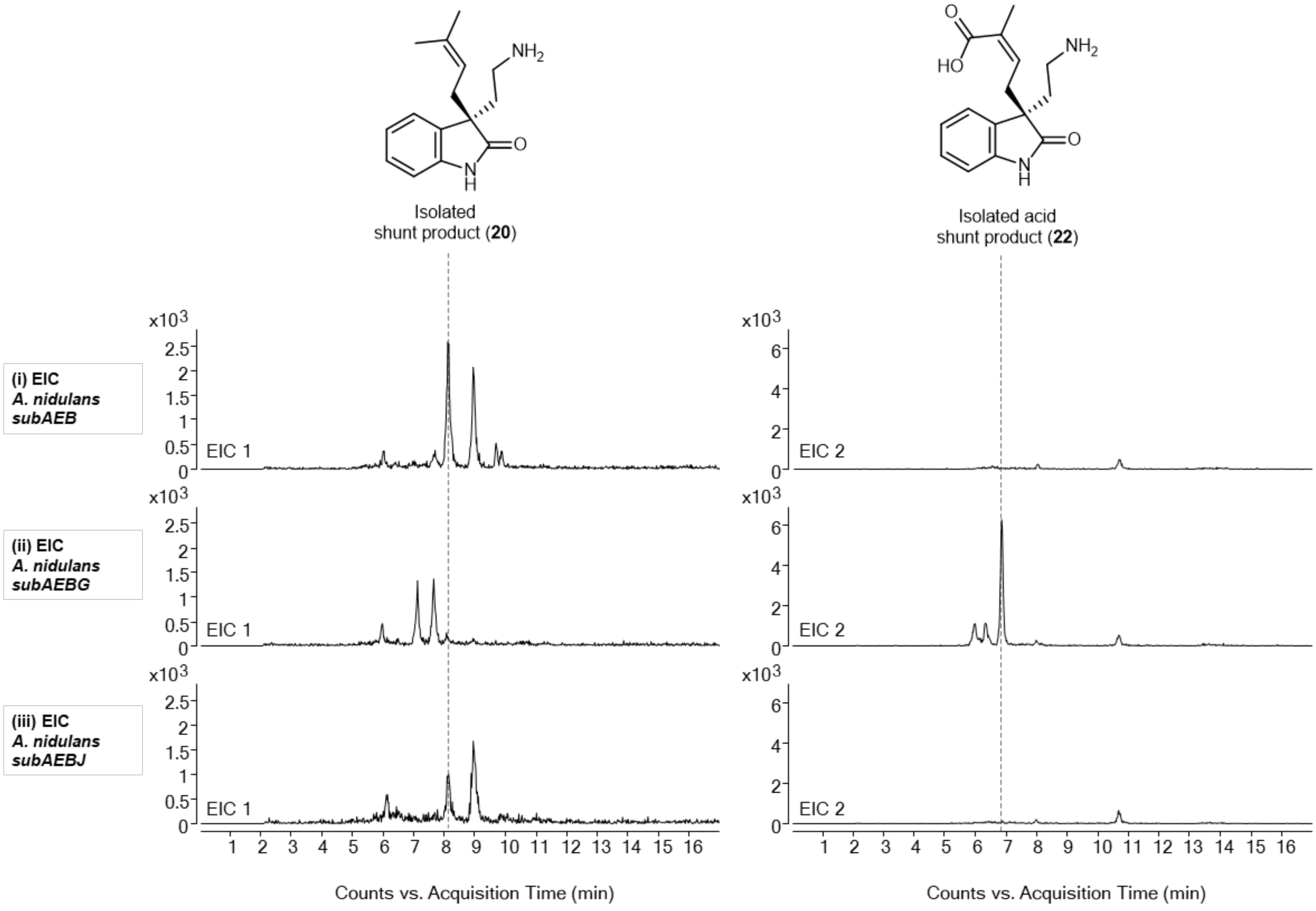

Fig. S22. ***In vivo*** **characterization of** ***A. nidulans*** **expressing** ***subAEBG*****.** The **20**-producing *A. nidulans* strain (expressing *subAEB*) was compared with strains expressing *subAEB* and the remaining P450s (*subG* and *subJ*). Complete disappearance of **20** and concomitant appearance of **22** was observed in the *subAEBG* strain. Isolated compound **22** corresponds to oxidized **20**. Although compound **20** was absent from *subAEGJ* traces, careful analysis of corresponding chromatograms did not reveal additional metabolites. Moreover, *subJ* shares significant homology with a number of benzoate 4-monooxygenase-type P450s, indicating that this enzyme may catalyze aromatic ring hydroxylation. (Based on our retrobiosynthetic analysis, this was predicted to occur after propellane formation.) Meanwhile SubG bears homology to P450s performing allylic oxidations, including trichodiene oxygenase (Tri4) and acoradiene epoxidase (FlvD). EIC 1 (merged): 227.1543, 228.1383, 245.1648 – corresponding to **20** (and corresponding hydrate and water/ammonia losses). EIC 2: 275.1390 – corresponding to **22**, a fully oxidized α,β-unsaturated acid.

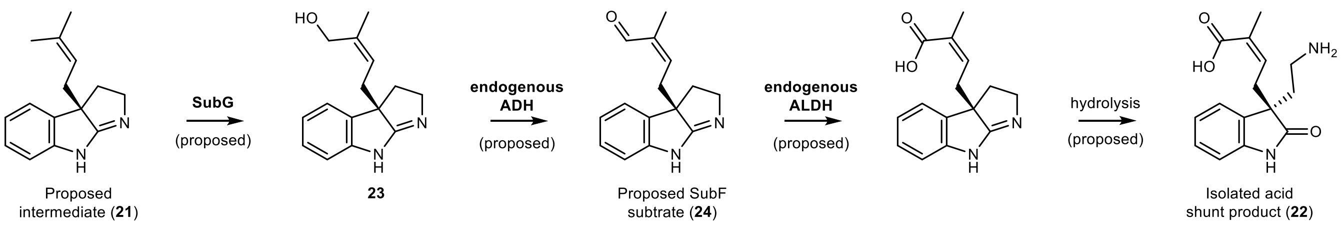

Fig. S23. Proposed formation of shunt product 22. Oxidation of α,β-unsaturated alcohols to their corresponding acids is driven by the toxicity of α,β-unsaturated aldehydes, which are considered potent mutagens known to include DNA-breakage. The well-established detoxification pathway involves alcohol oxidation by an alcohol dehydrogenase (ADH), followed by aldehyde oxidation by an aldehyde dehydrogenase (ALDH).

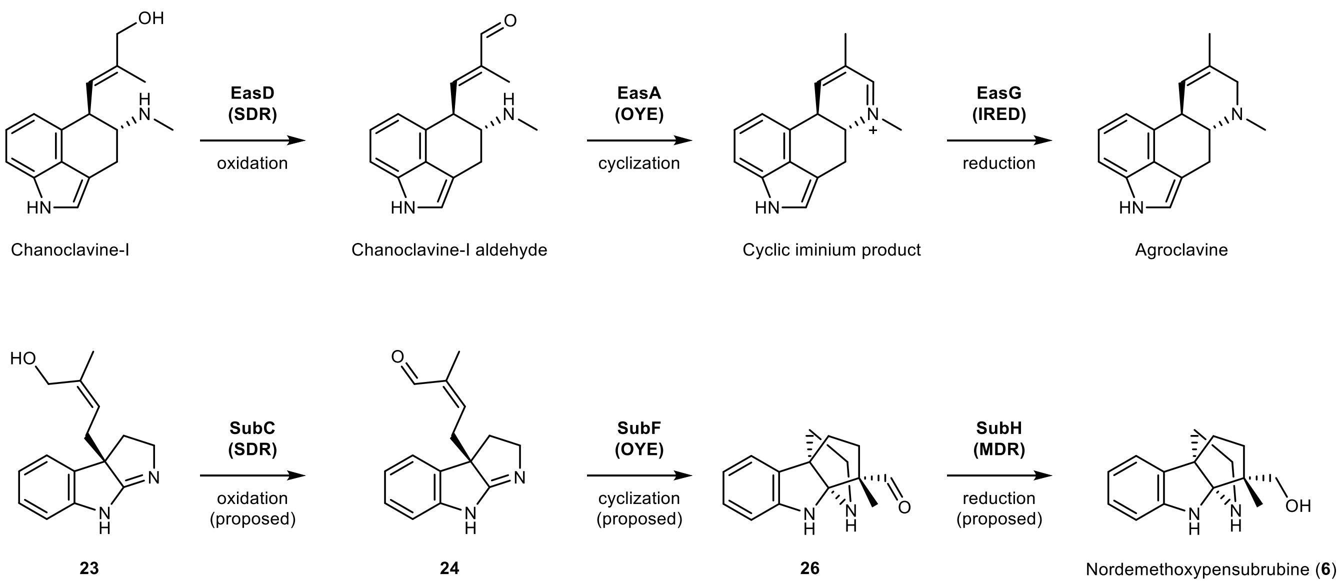

Fig. S24. Ergot alkaloid formation versus proposed propellane alkaloid formation. A comparison of the biosynthetic logic of the related ergot scaffold with subrubines is shown. The hypothesis is built around the role of the non-canonical OYE EasA, which facilitates chanoclavine-I aldehyde cyclization through α,β-unsaturated aldehyde isomerization. Both pathways involve activation of an α,β-alcohol by an SDR, followed by conversion of a tricyclic scaffold to a tetracyclic scaffold by an OYE, and subsequent reduction to a stable product (by either an IRED or MDR).

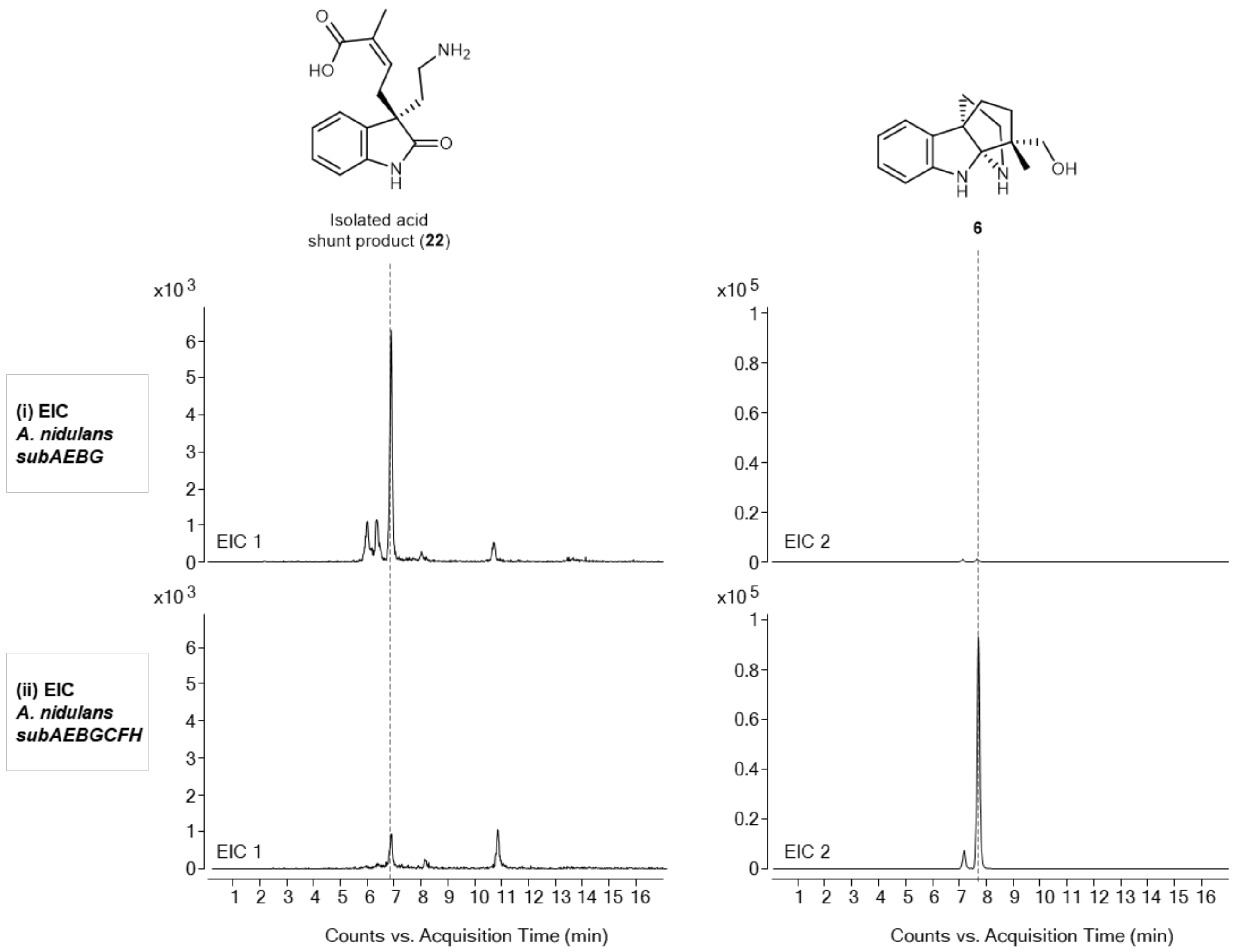

Fig. S25. ***In vivo*** **characterization of** ***A. nidulans*** **expressing** ***subAEBGCFH.*** The **22**-producing *A. nidulans* strain (expressing *subAEBG*) was compared to a strain expressing the remaining three genes (*subCFH*) which were not accounted for by our retrobiosynthetic analysis. (SubI was predicted to convert **6** to **5**, the remaining P450 (SubJ) and SubD were predicted to convert **5** to **4**, and SubK was predicted to convert **4** to **1**/**2**/**3**.) The disappearance of shunt product **22** and production of **6** was observed in the *subAEBGCFH* strain. Relative production of **6** upon individual and combinatorial expression of *subC/subF/subH* is shown in Fig. 4b. EIC 1: 275.1390 – corresponding to the α,β-unsaturated acid **22.** EIC 2: 245.1648 – corresponding to **6**.

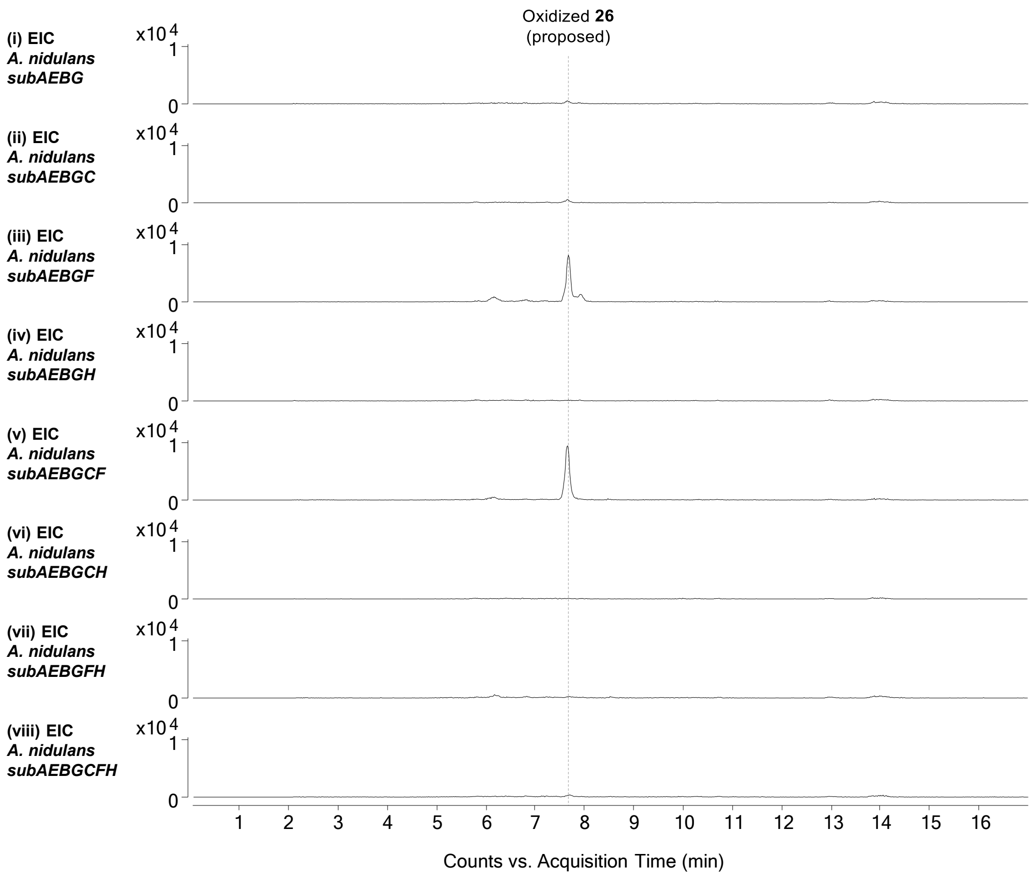

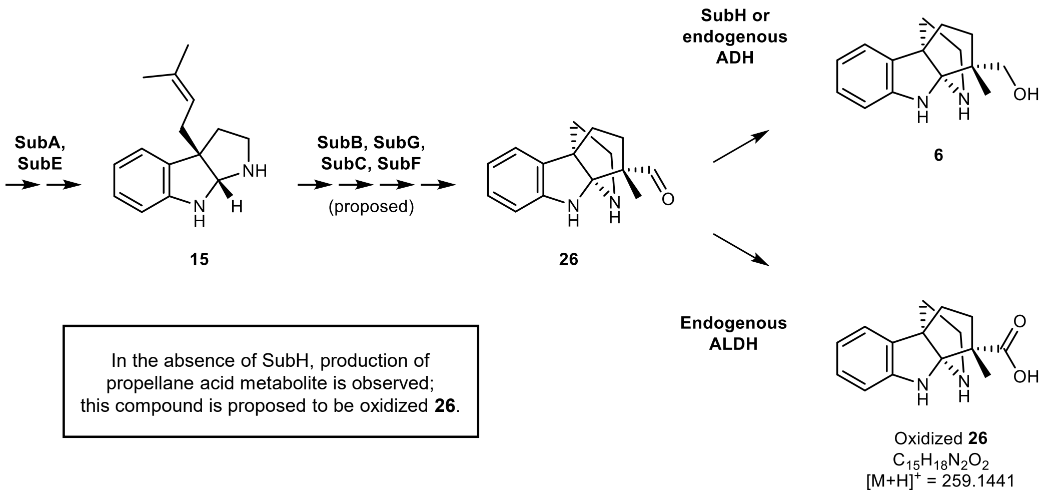

Fig. S26. *In vivo* detection of putative propellane acid in the absence of *subH*. The minimum set of genes in the proposed *subCFH* sequence required for **6** formation was investigated (Fig. 4b). *In vitro* analysis of the individual roles of each enzyme was hampered by the inability to synthesize/isolate proposed pathway intermediates. We therefore inspected the LC-MS traces for evidence of shunt products to bolster our proposal for the role of SubH as an aldehyde reductase. In *subAEBGC* and *subAEBGCF* strains (which both produce **6**) we observed a peak with an exact mass (EIC: 259.1441) matching a putative propellane acid. This compound disappeared upon addition of *subH*.

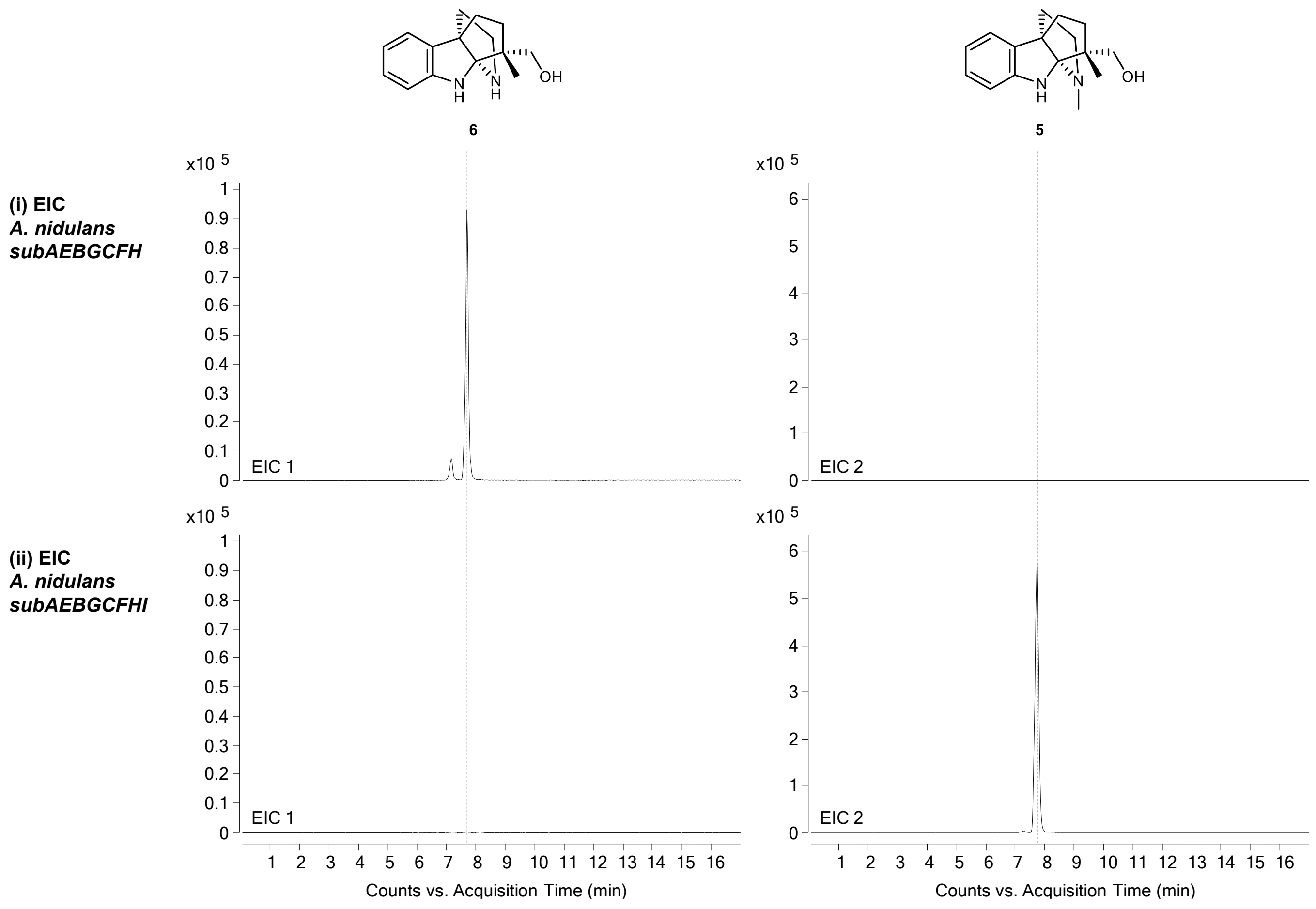

Fig. S27. ***In vivo*** **characterization of *A. nidulans* expressing *subAEBGCFHI*.** The **6**-producing *A. nidulans* strain (expressing *subAEBGCFH*) was compared to a strain expressing the gene encoding the *N*-methyltransferase SubI (*subAEBGCFHI*). The disappearance of **6** and production of **5** was observed in the *subAEBGCFHI* strain. We noted that *A. nidulans subEABGCFHI* makes 5–fold more **5** than *P. subrubescens* (and ~750-fold more than *A. terreus*). EIC 1: 245.1648 – corresponding to **6**. EIC 2: 259.1805 – corresponding to **5**.

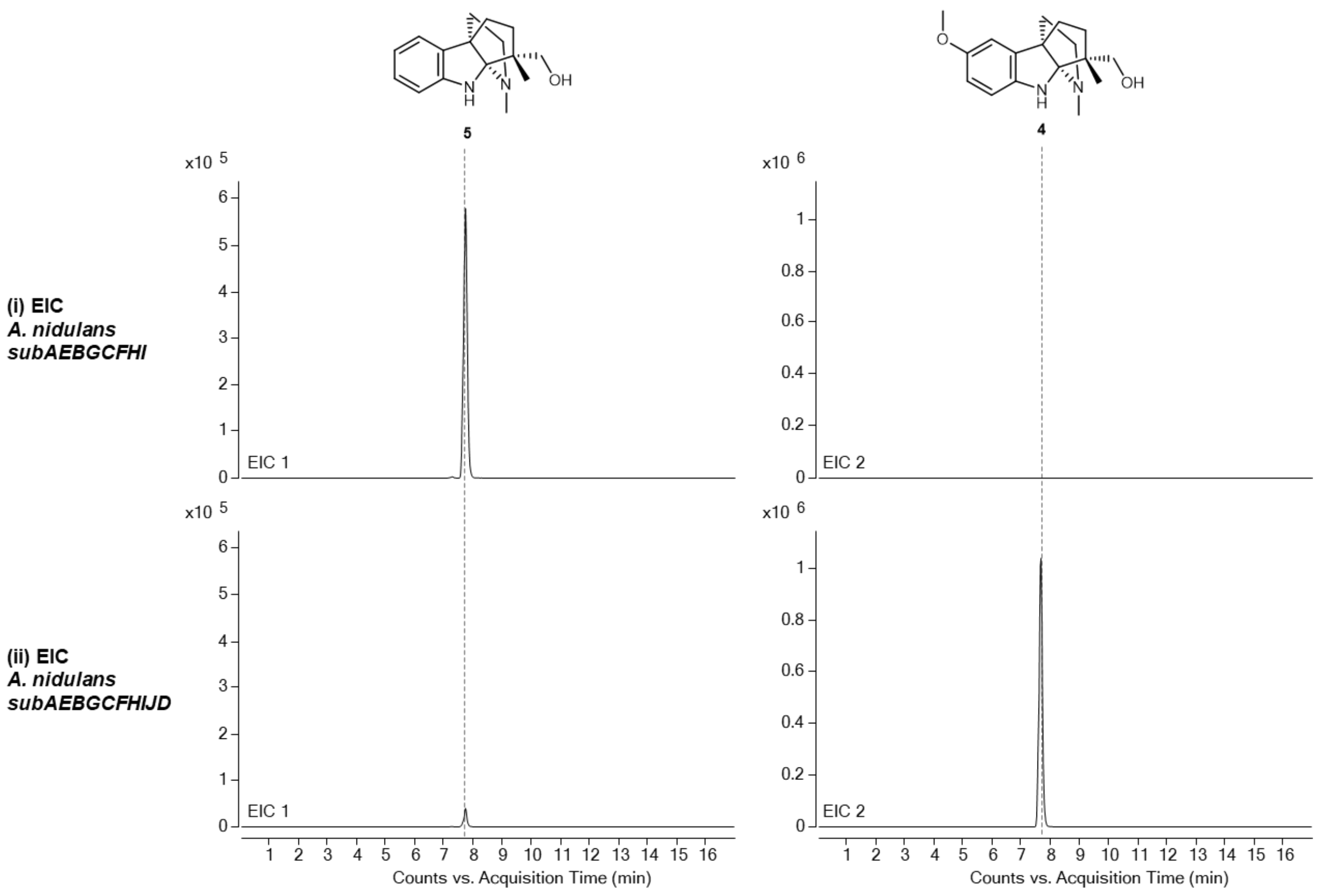

Fig. S28. ***In vivo*** **characterization of** ***A. nidulans*** **expressing** ***subAEBGCFHIJD*****.** The **5**-producing *A. nidulans* strain (expressing *subAEBGCFHI*) was compared to a strain expressing genes encoding the final P450 SubJ and the *O*-methyltransferase SubD (*subAEBGCFHIJD*). The disappearance of **5** and production of **4** was observed in the *subAEBGCFHIJD* strain. EIC 1: 259.1805 – corresponding to **5**. EIC 2: 289.1911 – corresponding to **4**.

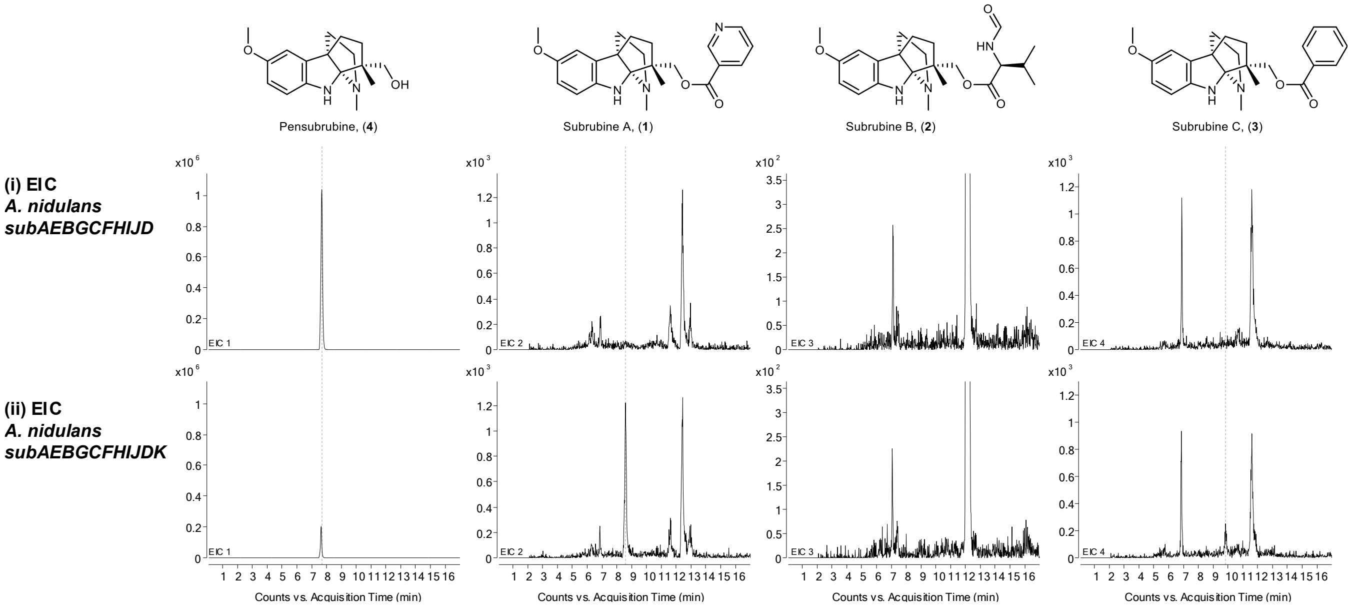

Fig. S29. ***In vivo*** **characterization of** ***A. nidulans*** **expressing** ***subAEBGCFHIJDK*****.** The **4**-producing *A. nidulans* strain (expressing *subAEBGCFHIJD*) was compared to a strain expressing the final gene encoding the NRPS, SubK (*subAEBGCFHIJDK*). The disappearance of **4** and low-level production of **1** and **3** was observed in the *subAEBGCFHIJD* strain. EIC 1: 289.1911 – corresponding to **4**. EIC 2: 394.2125 – corresponding to **1**. EIC 3: 416.2544 – corresponding to **2**. EIC 4: 393.2173 – corresponding to **3**.

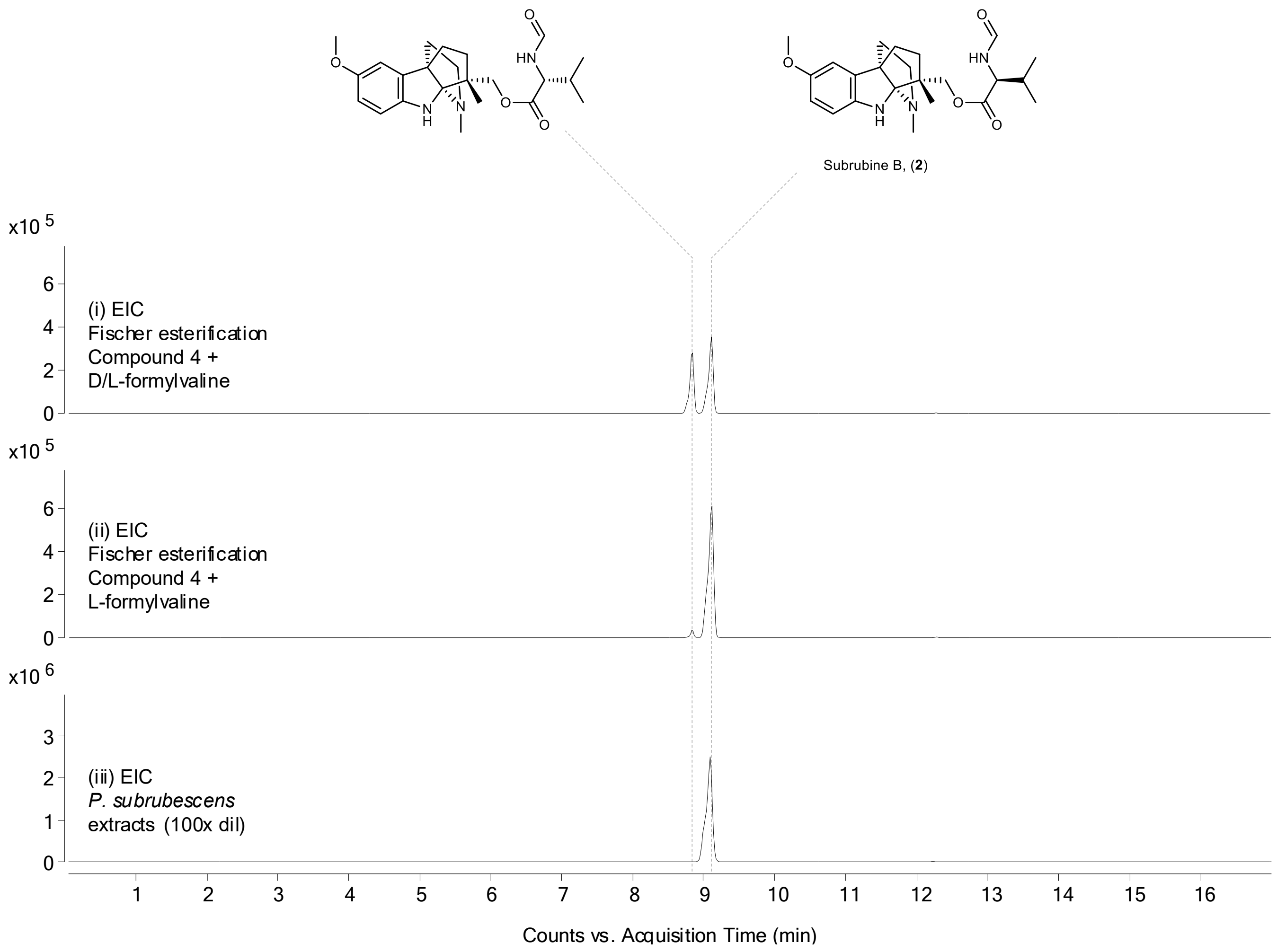

Fig. S30. Determination of *N*-formyl-valine configuration. Production of **2** (subrubine B, the pensubrubine *N*-formyl-L-valine ester), as well as a mixture of pensubrubine *N*-formyl-D/L-valine esters was carried out via Fischer esterification (D-formylvaline was not commercially available, however D/L-formylvaline and L-formylvaline could both be purchased). The resulting diastereomers were successfully resolved using standard LC-MS conditions. Comparison of retention times confirmed that pensubrubine *N*-formyl-L-valine (RT = 9.12 min) was produced by *P. subrubescens*, while pensubrubine *N*-formyl-D-valine (RT = 8.84 min) was not. EIC: 416.2544 – corresponding to **2**.

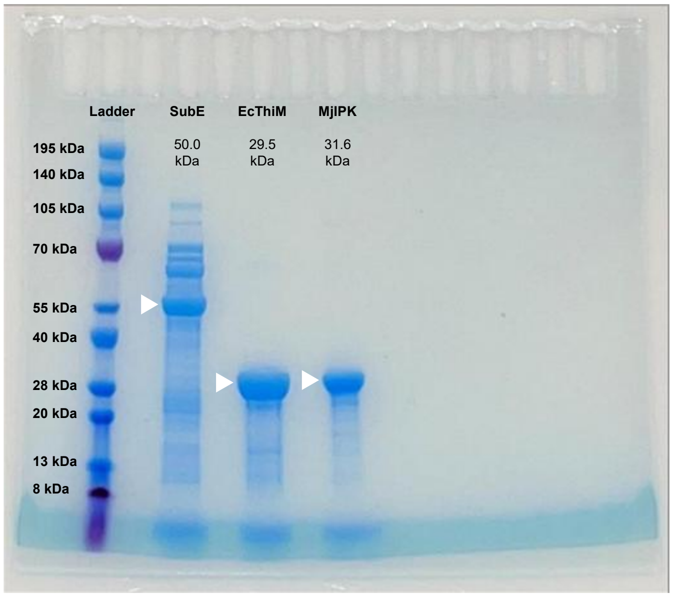

Fig. S31. SDS−PAGE analysis of EcThiM, MjIPK, and SubE expressed in *E. coli*. The isolated proteins were run on a gel to confirm size and assess relative purity. The protein of interest was the predominant band in all cases. Although SubE was the least pure of the proteins employed in our cell-free cascade, optimization of the affinity tag, expression conditions, or chromatography conditions could improve yields and purity.

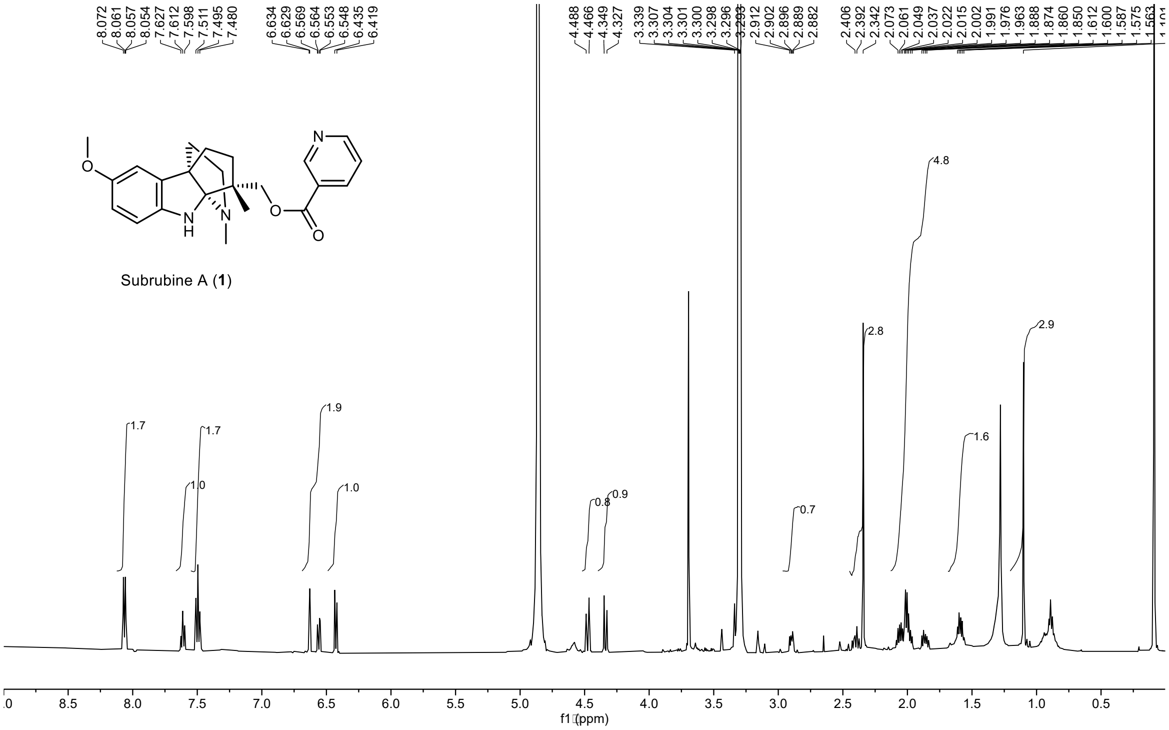

Fig. S32. ^{1}H NMR spectrum of subrubine A (**1**) in CD_3OD (500 MHz).

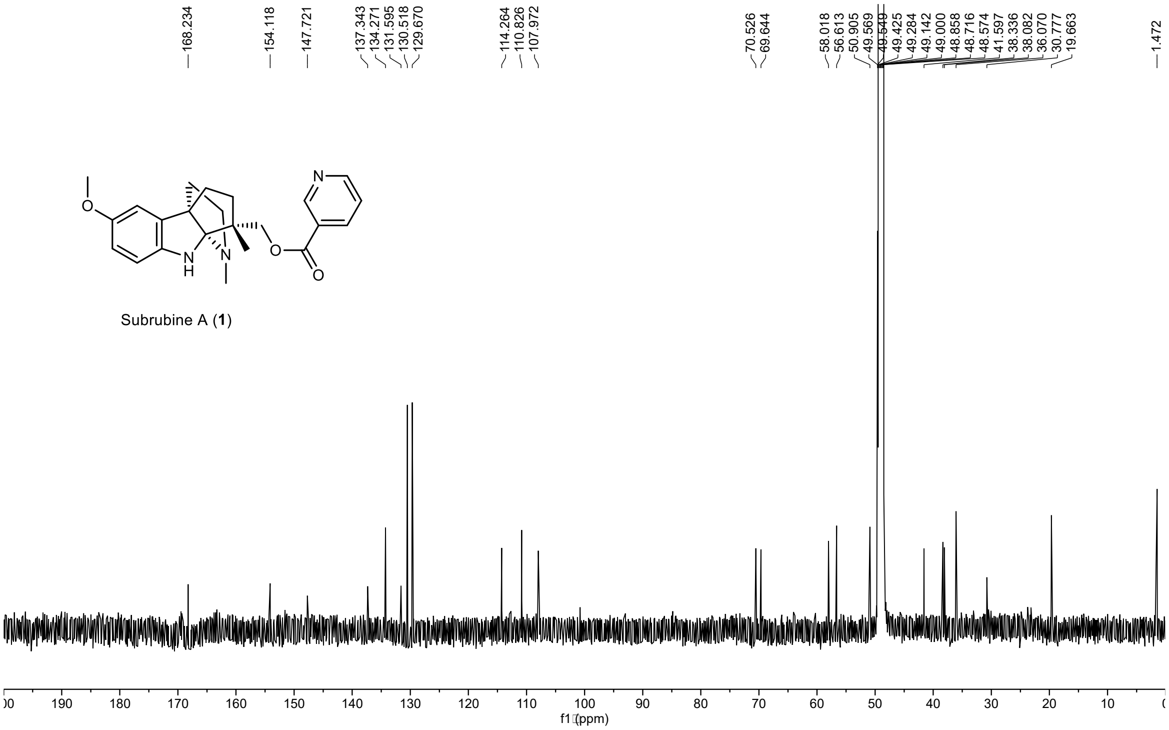

Fig. S33. ^{13}C NMR spectrum of subrubine A (**1**) in CD_3OD (125 MHz).

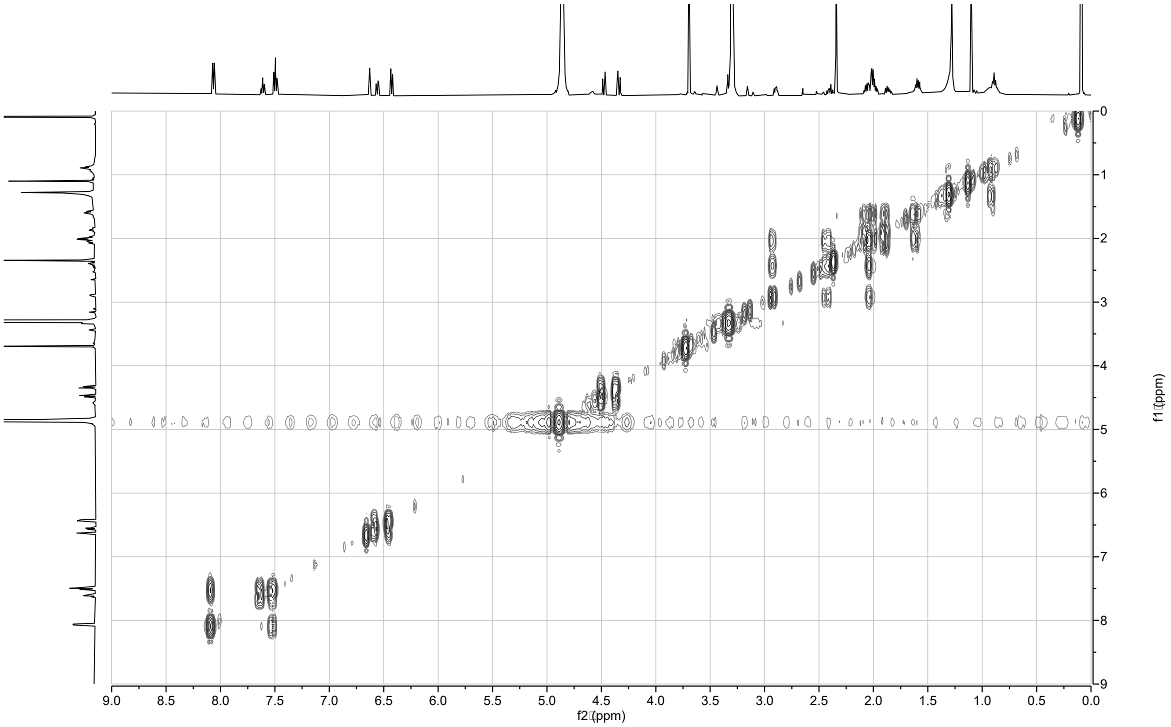

Fig. S34. ^{1}H-^{1}H COSY spectrum of subrubine A (**1**) in CD_3OD (500 MHz).

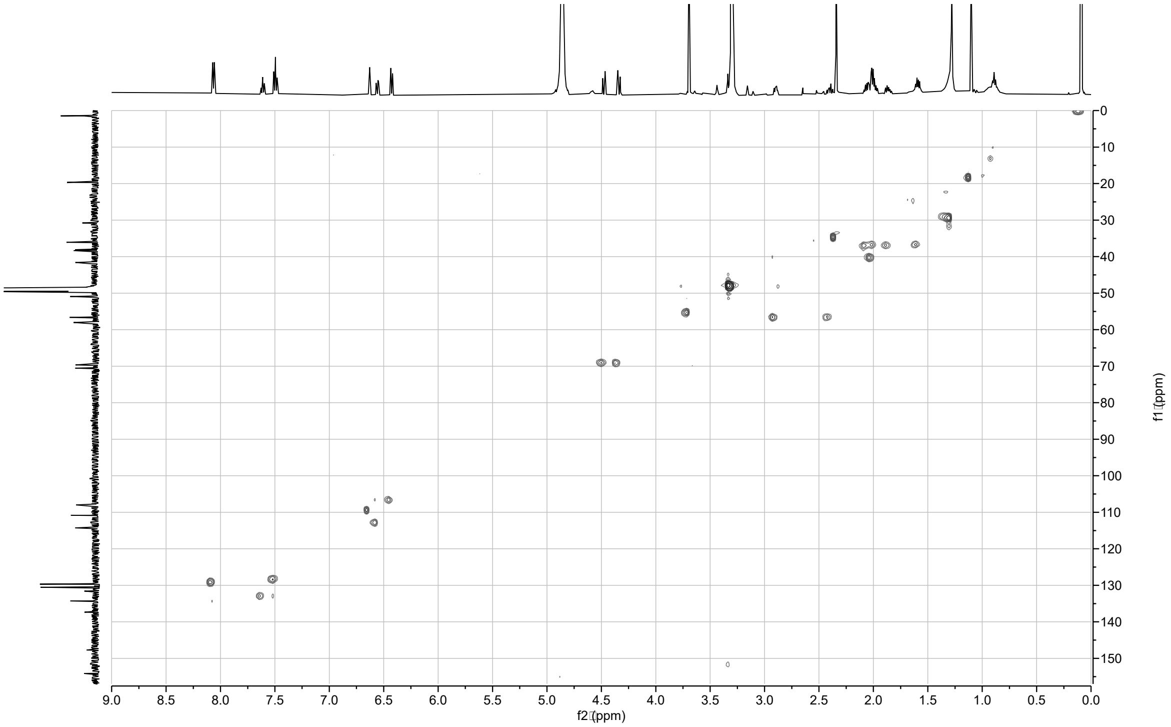

Fig. S35. HSQC spectrum of subrubine A (**1**) in CD_3OD (500 MHz).

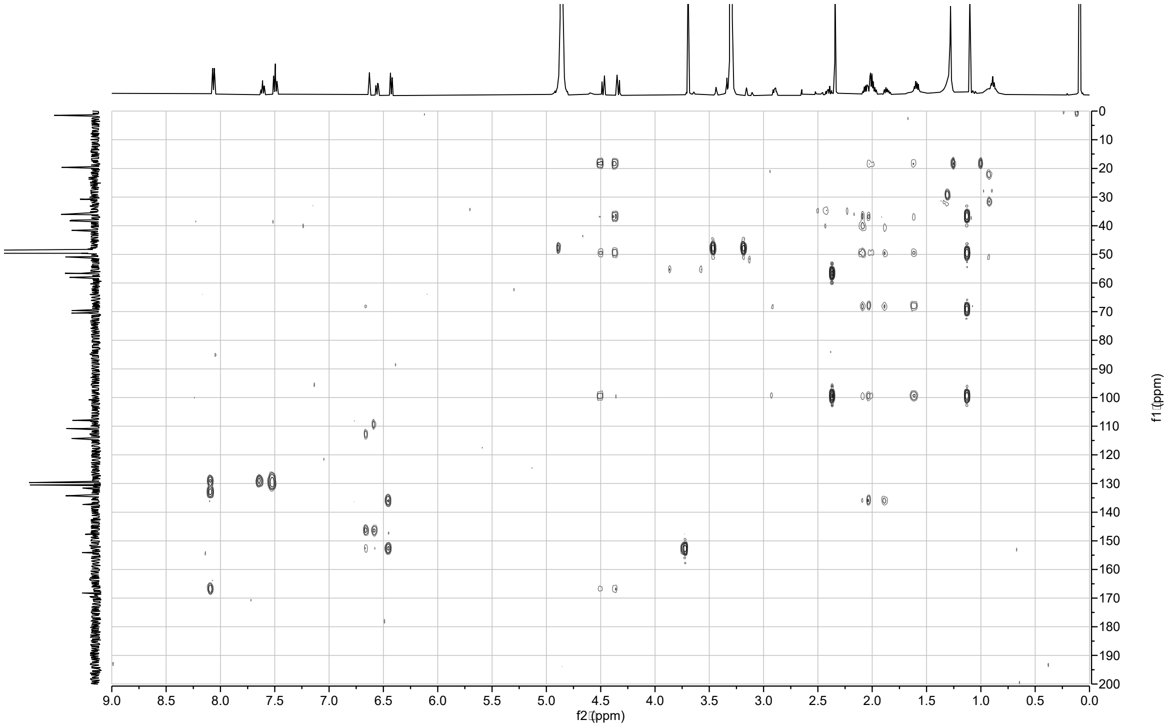

Fig. S36. HMBC spectrum of subrubine A (**1**) in CD_3OD (500 MHz).

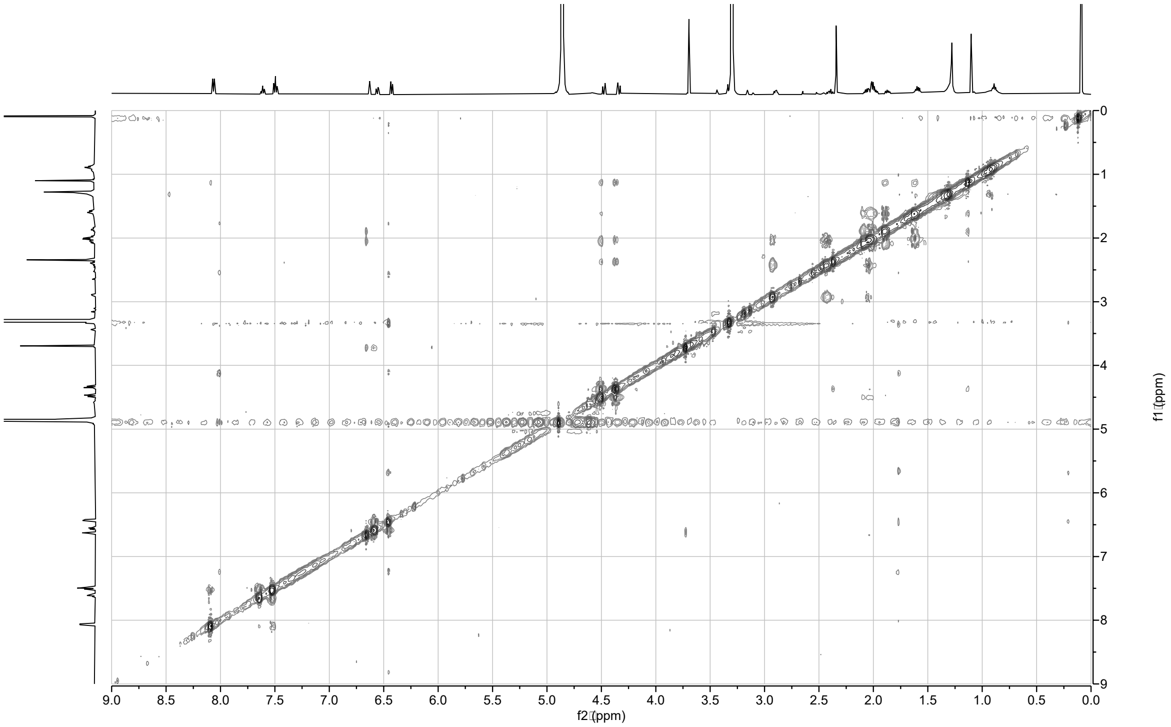

Fig. S37. NOESY spectrum of subrubine A (**1**) in CD_3OD (500 MHz).

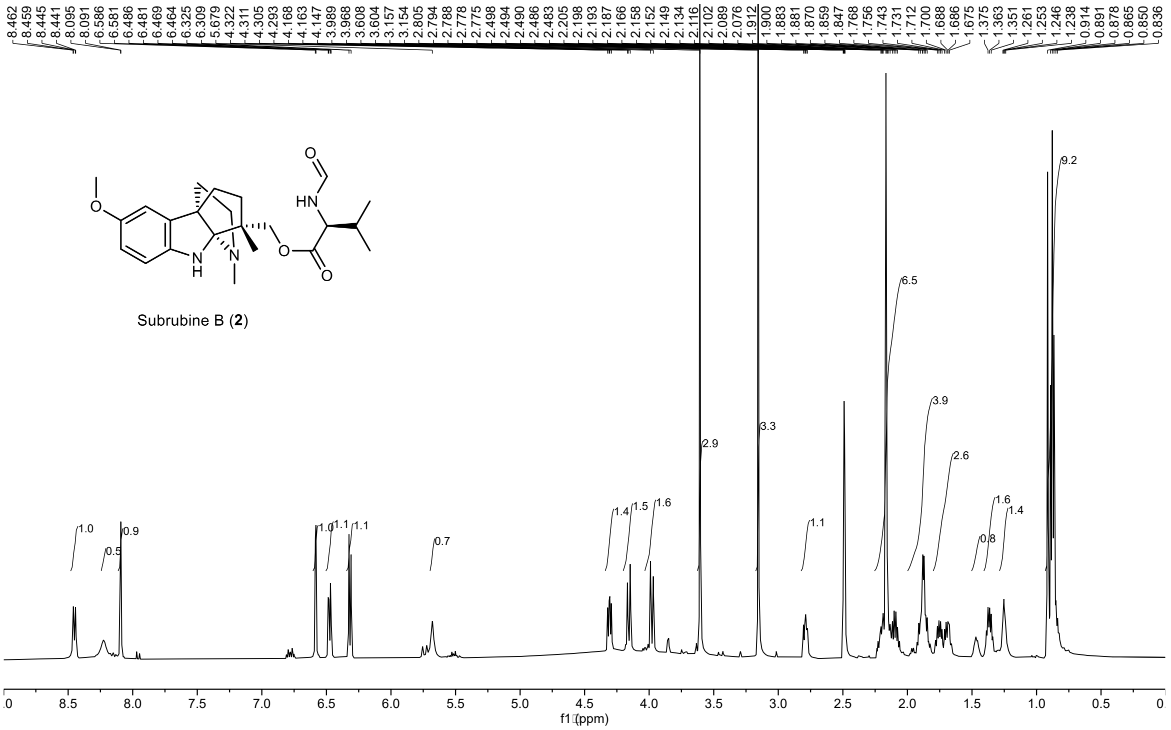

Fig. S38. ^{1}H NMR spectrum of subrubine B (**2**) in DMSO-d_6 (500 MHz).

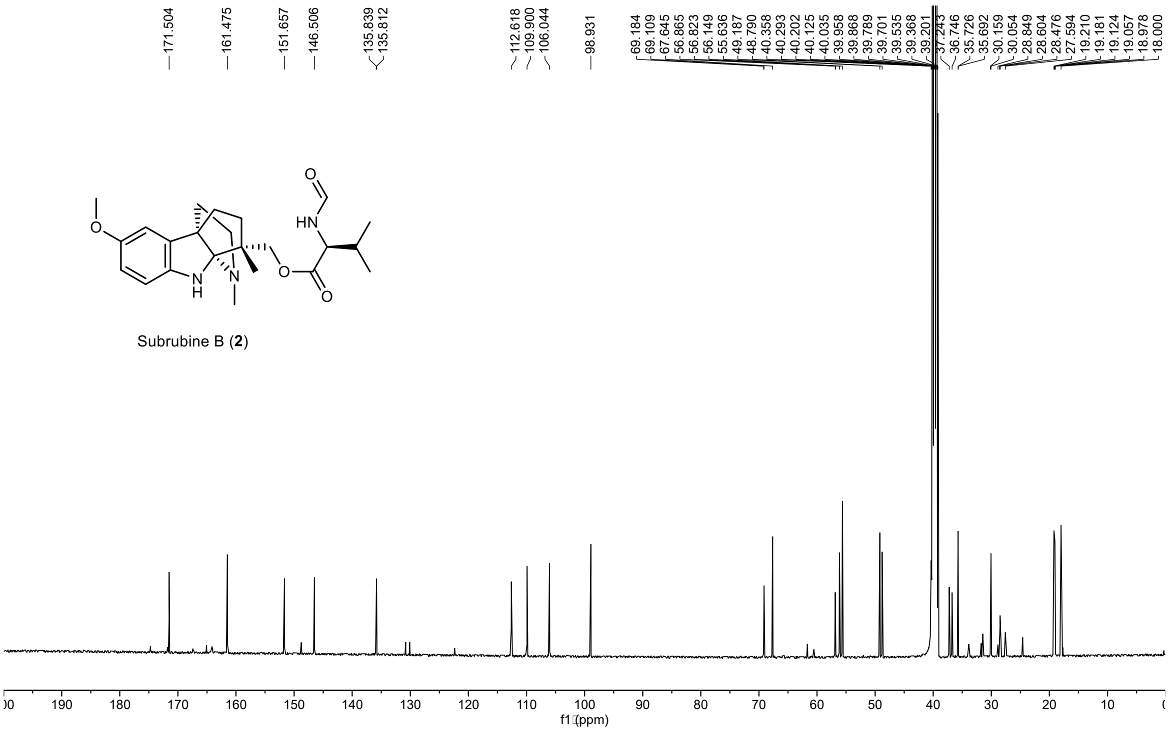

Fig. S39. ^{13}C NMR spectrum of subrubine B (**2**) in DMSO-d_6 (125 MHz).

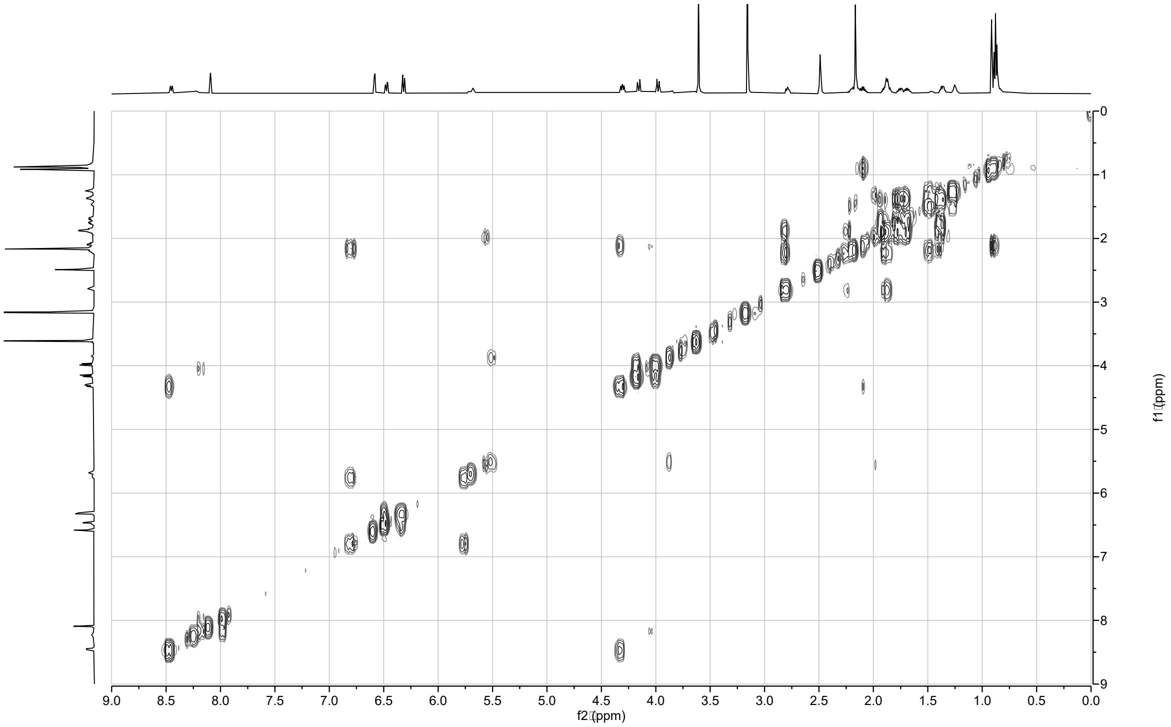

Fig. S40. ^{1}H-^{1}H COSY spectrum of subrubine B (**2**) in DMSO-d_6 (500 MHz).

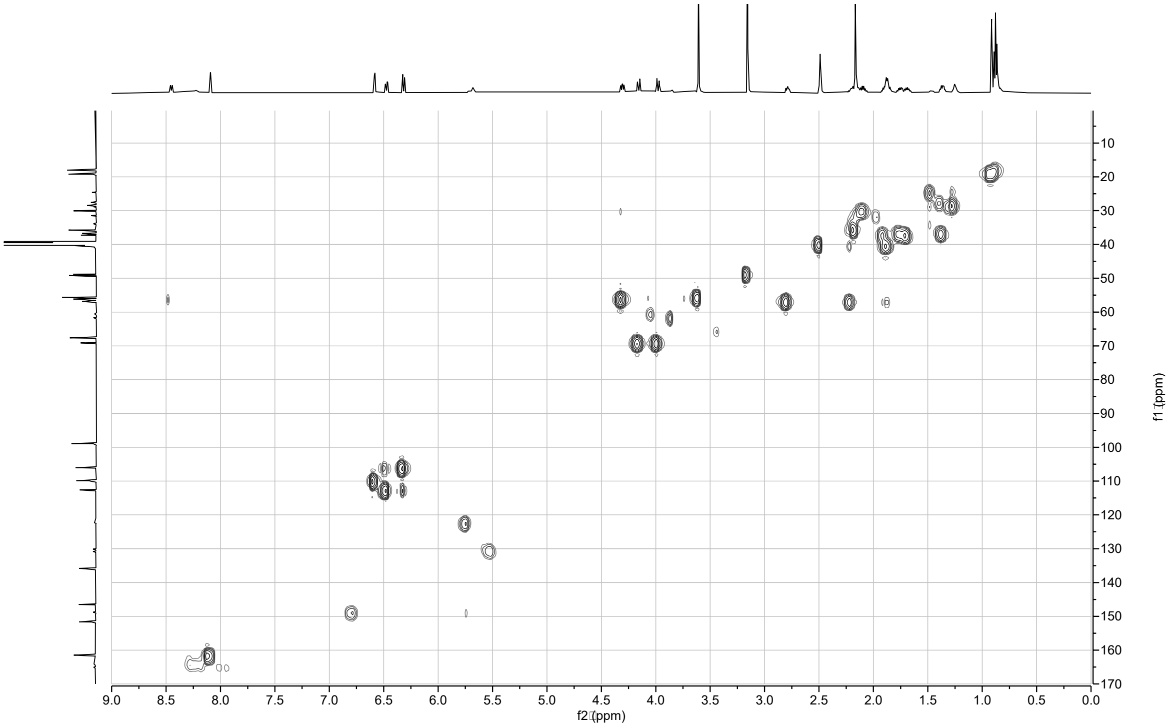

Fig. S41. HSQC spectrum of subrubine B (**2**) in DMSO-d_6 (500 MHz).

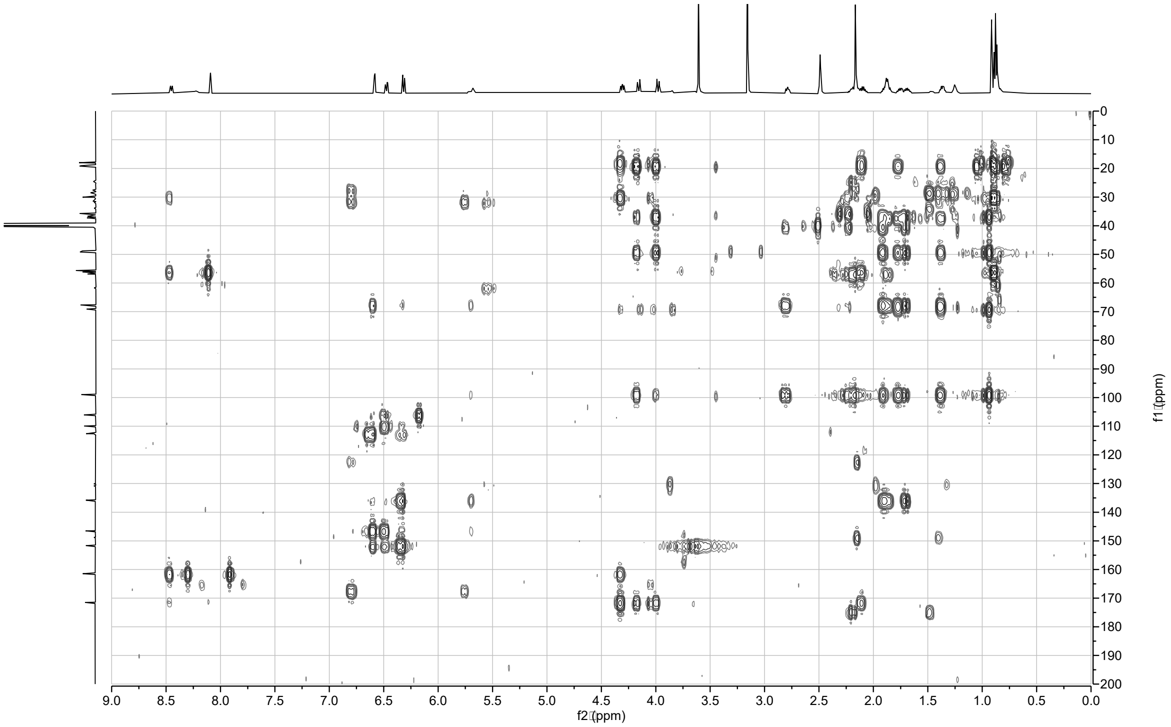

Fig. S42. HMBC spectrum of subrubine B (**2**) in DMSO-d_6 (500 MHz).

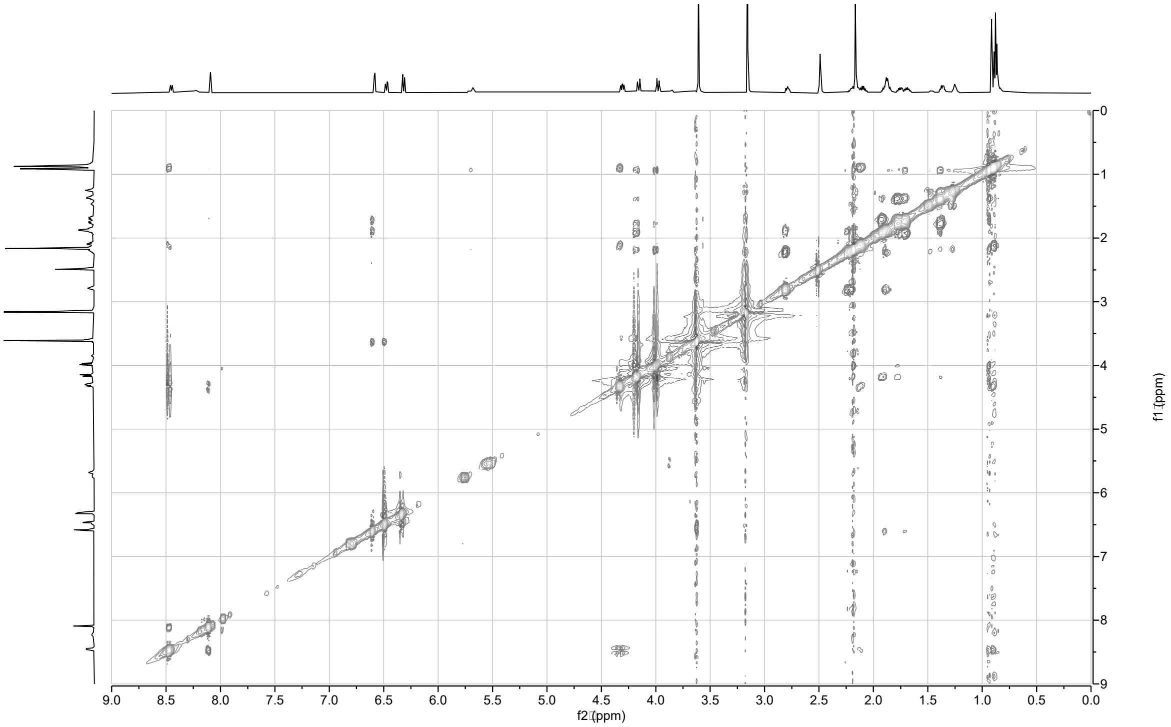

Fig. S43. ROESY spectrum of subrubine B (**2**) in DMSO-d_6 (500 MHz).

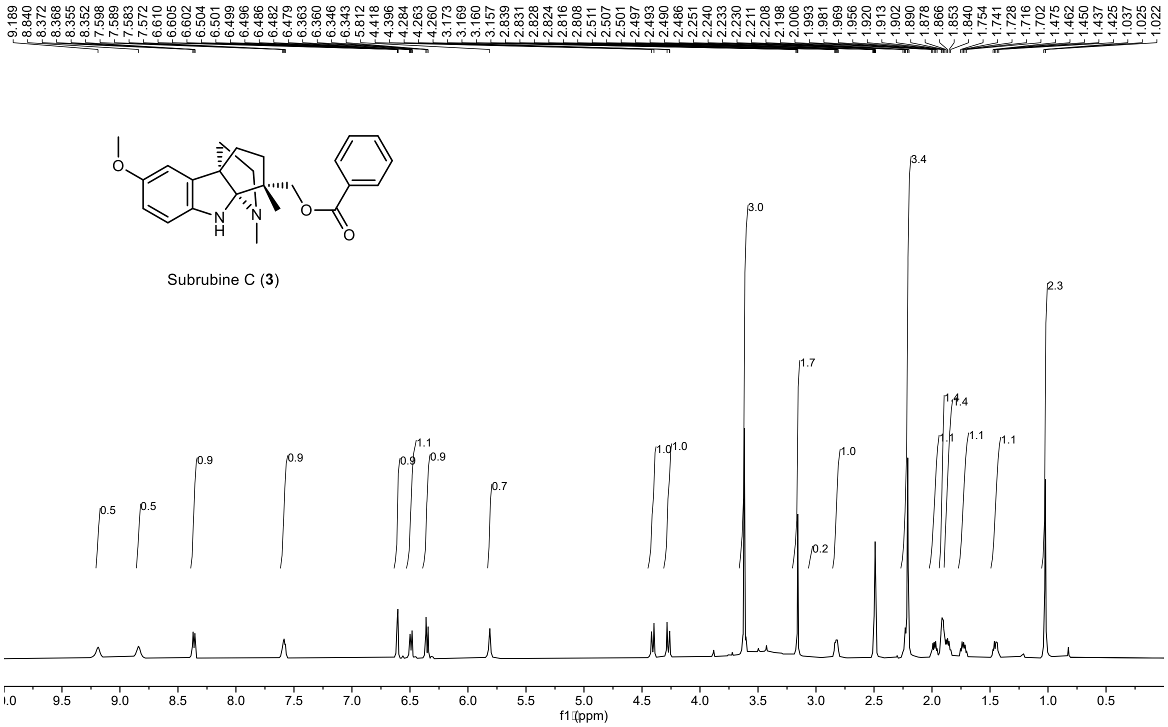

Fig. S44. ^{1}H NMR spectrum of subrubine C (**3**) in DMSO-d_6 (500 MHz).

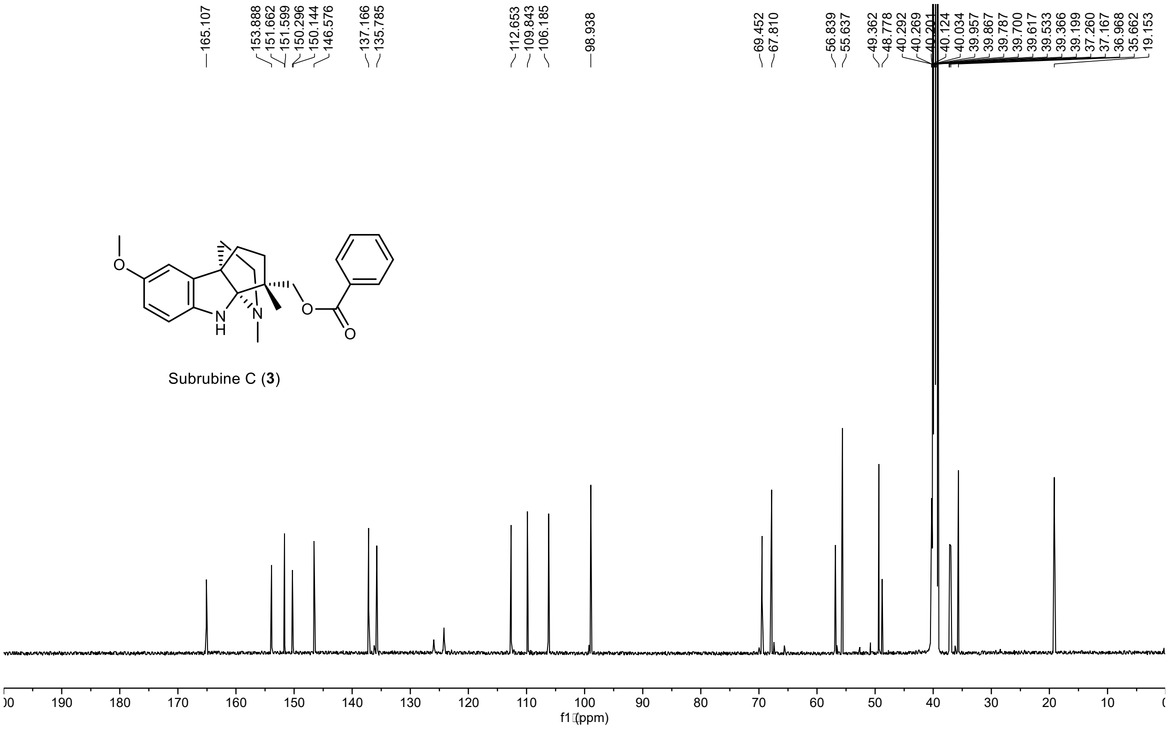

Fig. S45. ^{13}C NMR spectrum of subrubine C (**3**) in DMSO-d_6 (125 MHz).

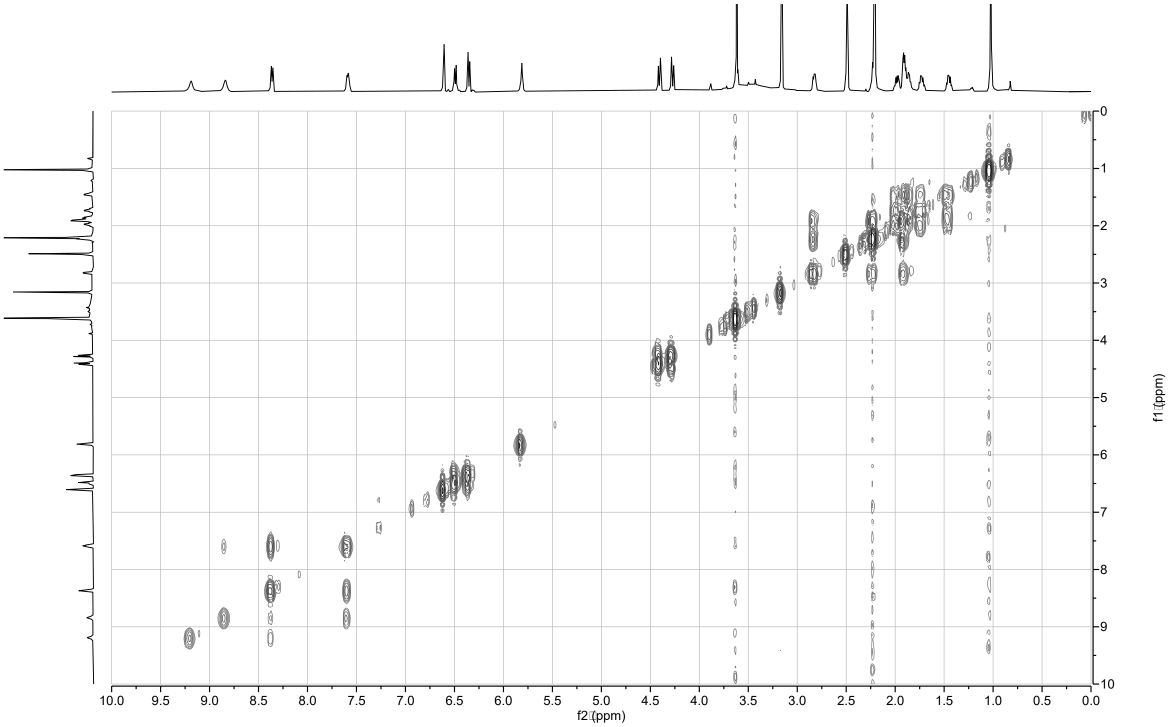

Fig. S46. ^{1}H-^{1}H COSY spectrum of subrubine C (**3**) in DMSO-d_6 (500 MHz).

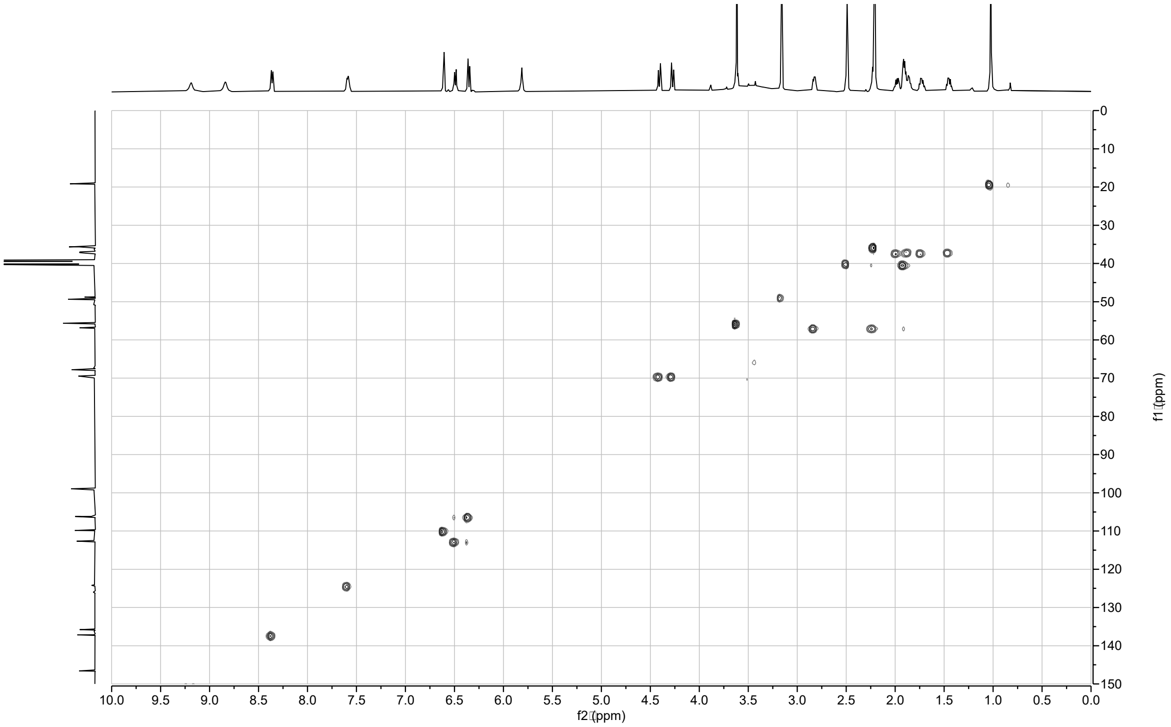

Fig. S47. HSQC spectrum of subrubine C (**3**) in DMSO-d_6 (500 MHz).

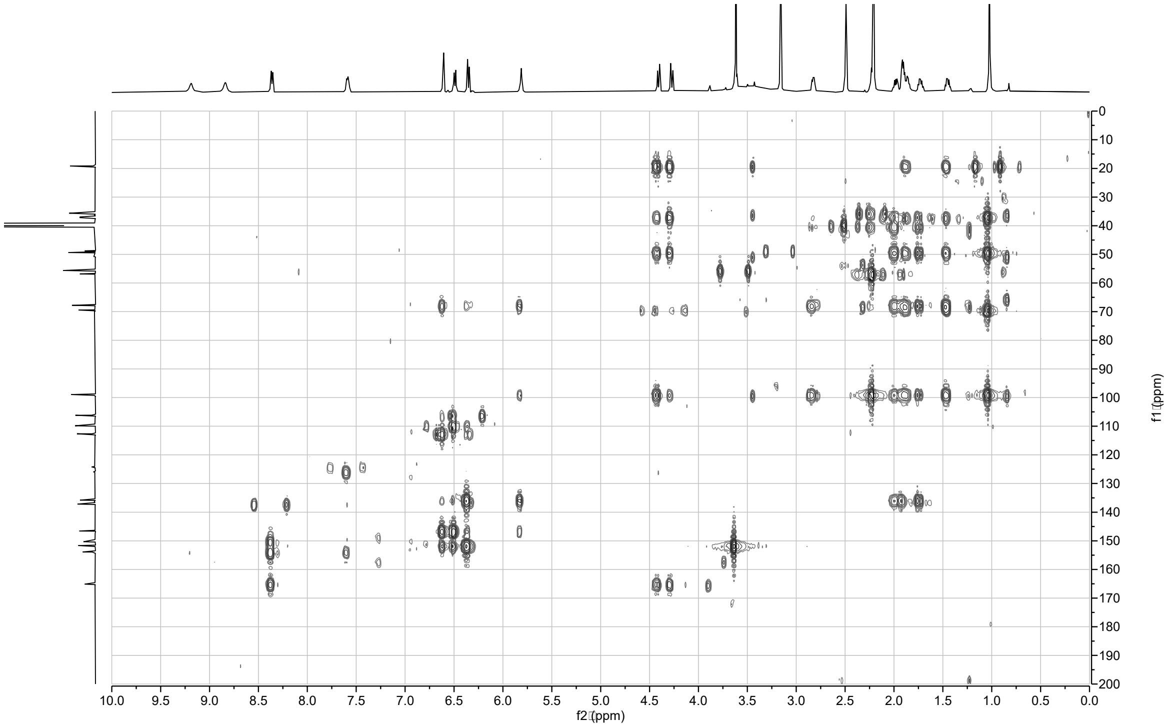

Fig. S48. HMBC spectrum of subrubine C (**3**) in DMSO-d_6 (500 MHz).

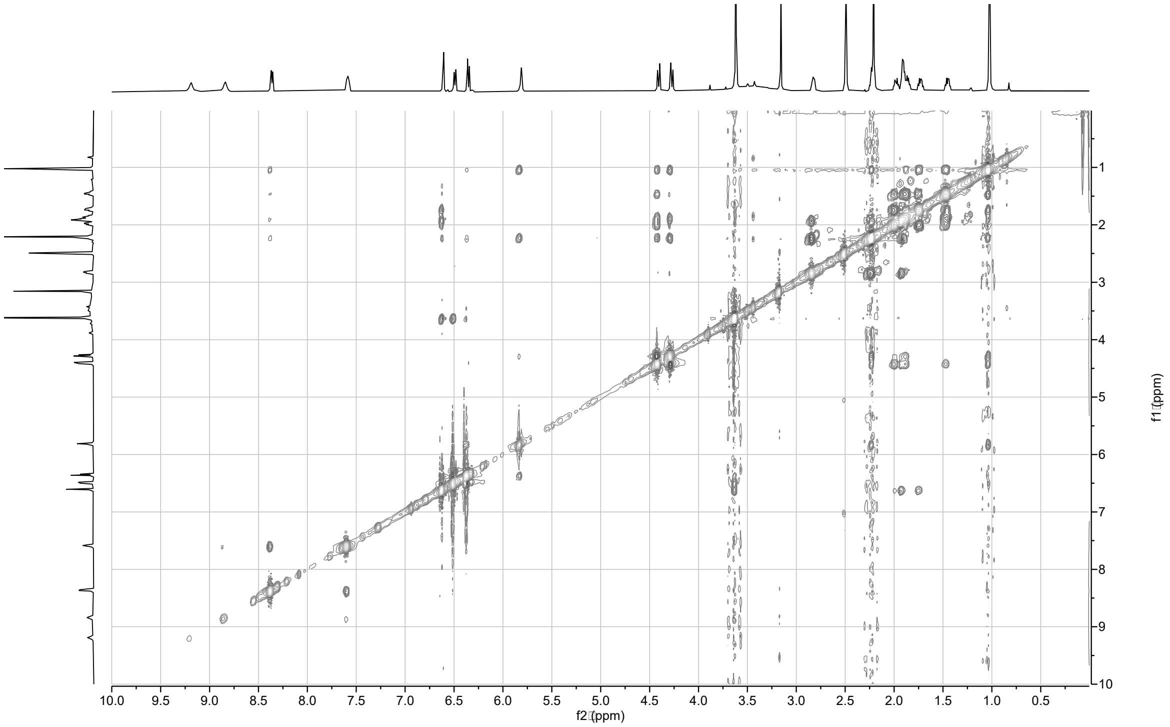

Fig. S49. ROESY spectrum of subrubine C (**3**) in DMSO-d_6 (500 MHz).

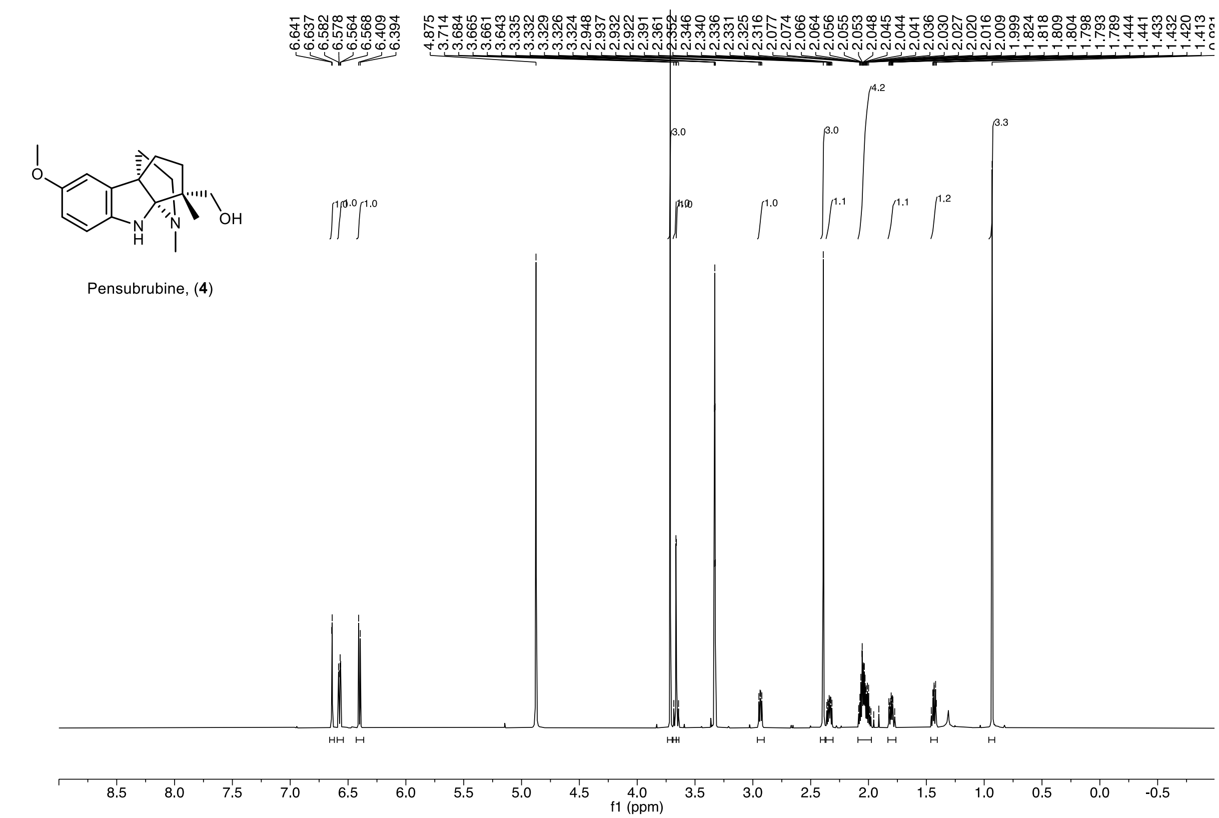

Fig. S50. ^{1}H NMR spectrum of pensubrubine (**4**) in CD_3OD (600 MHz).

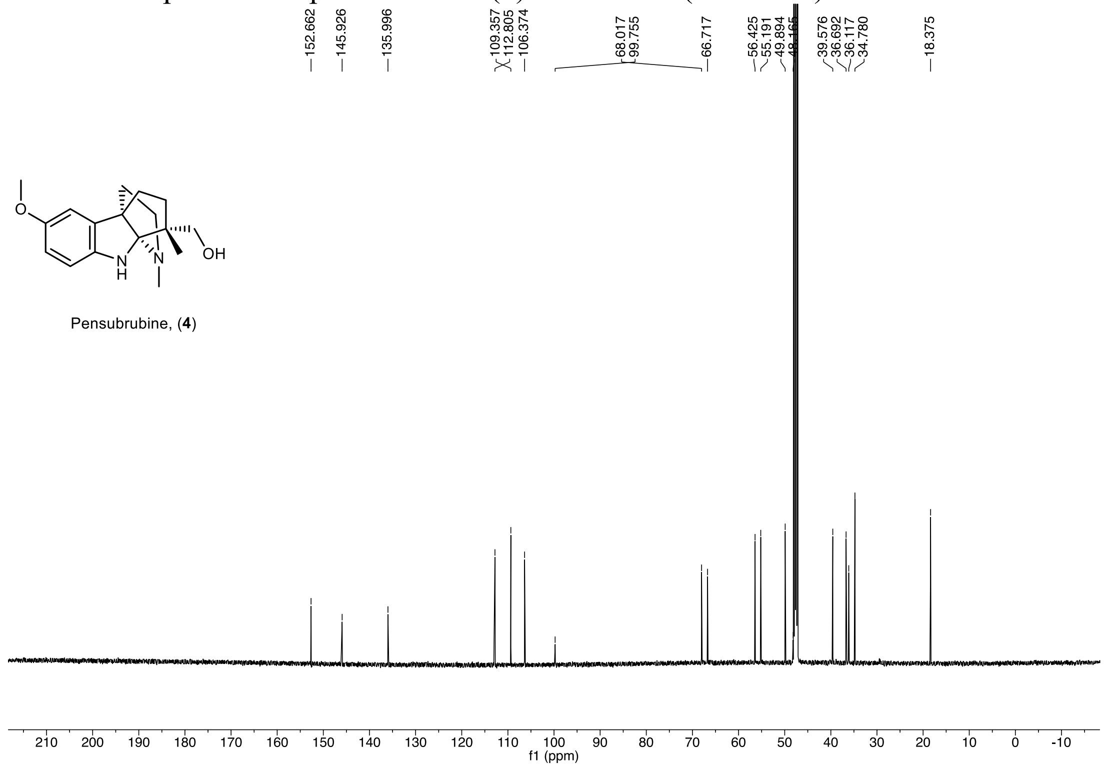

Fig. S51. ^{13}C NMR spectrum of pensubrubine (**4**) in CD_3OD (151 MHz).

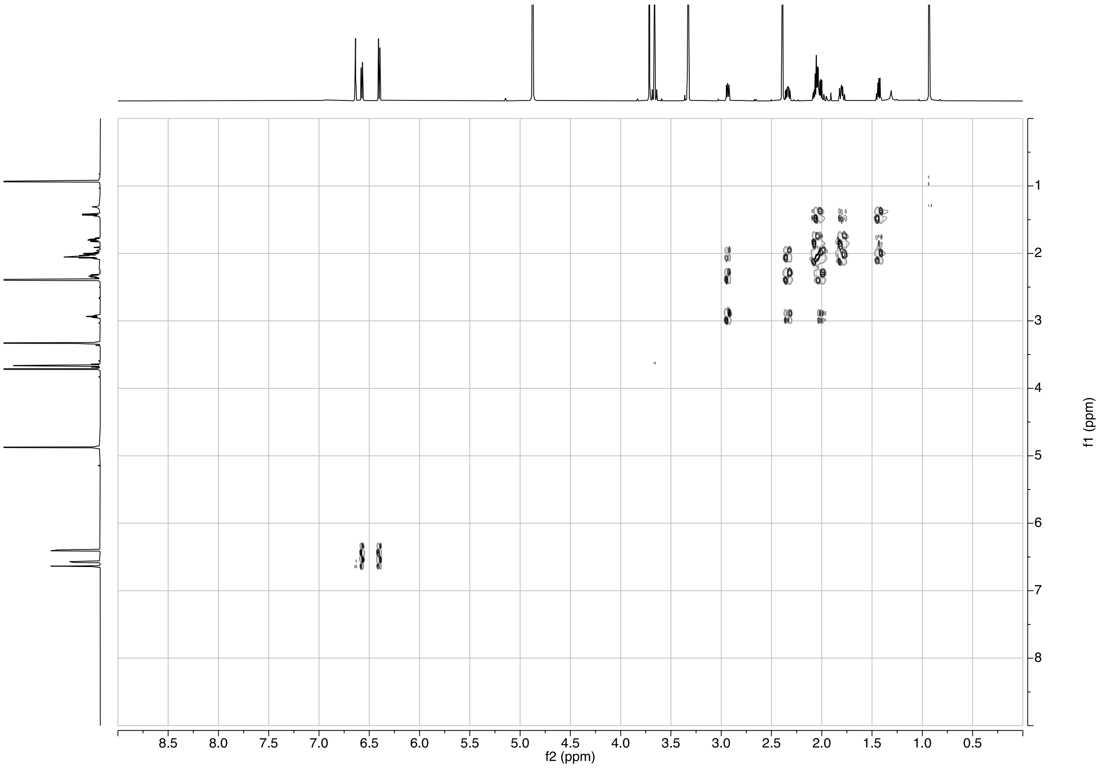

Fig. S52. ^{1}H-^{1}H COSY spectrum of pensubrubine (**4**) in CD_3OD (600 MHz).

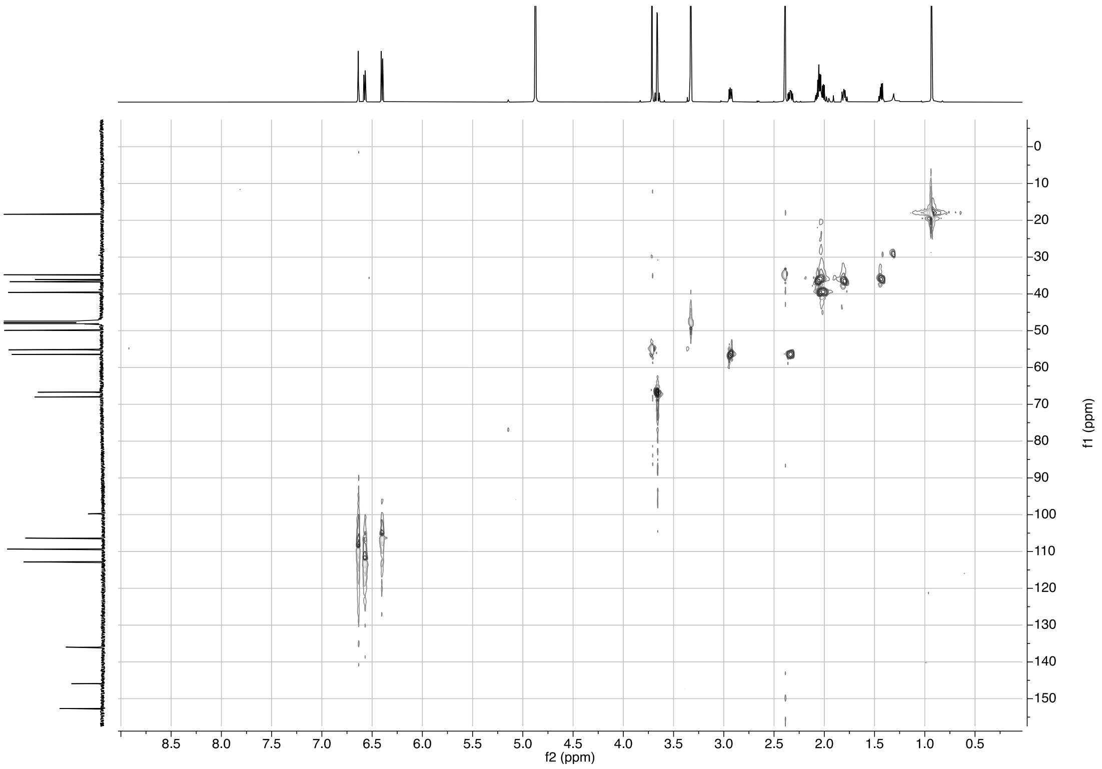

Fig. S53. HSQC spectrum of pensubrubine (**4**) in CD_3OD (600 MHz).

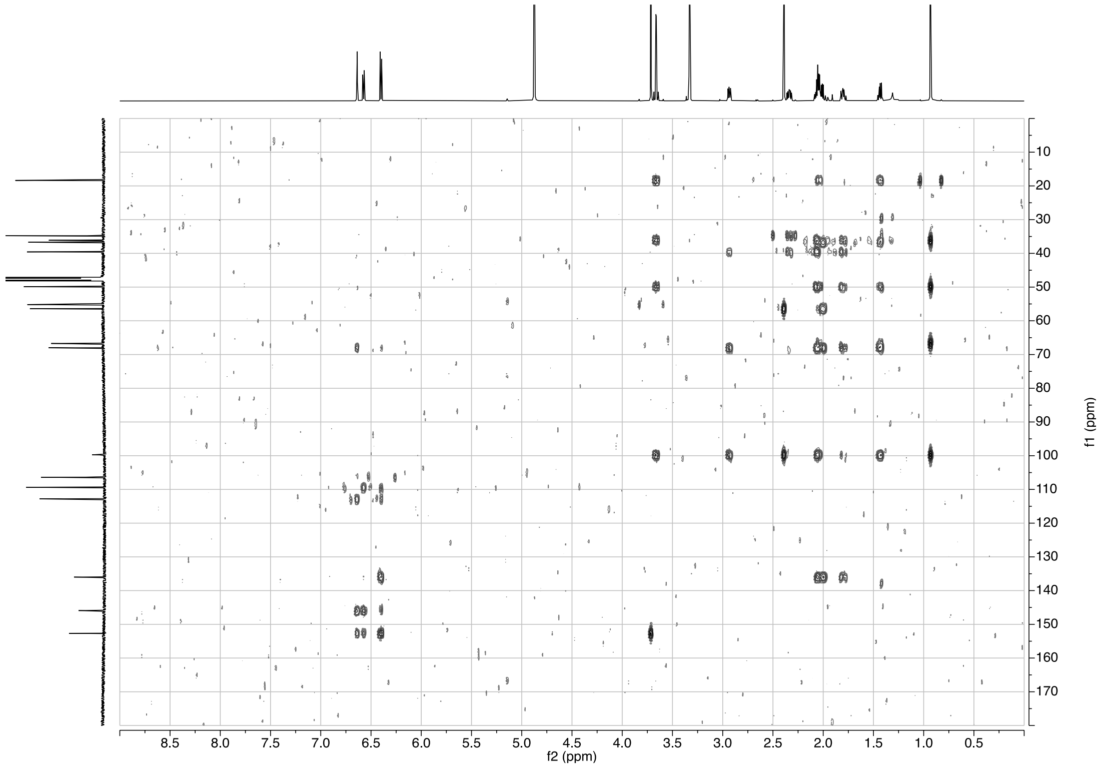

Fig. S54. HMBC spectrum of pensubrubine (**4**) in CD_3OD (600 MHz).

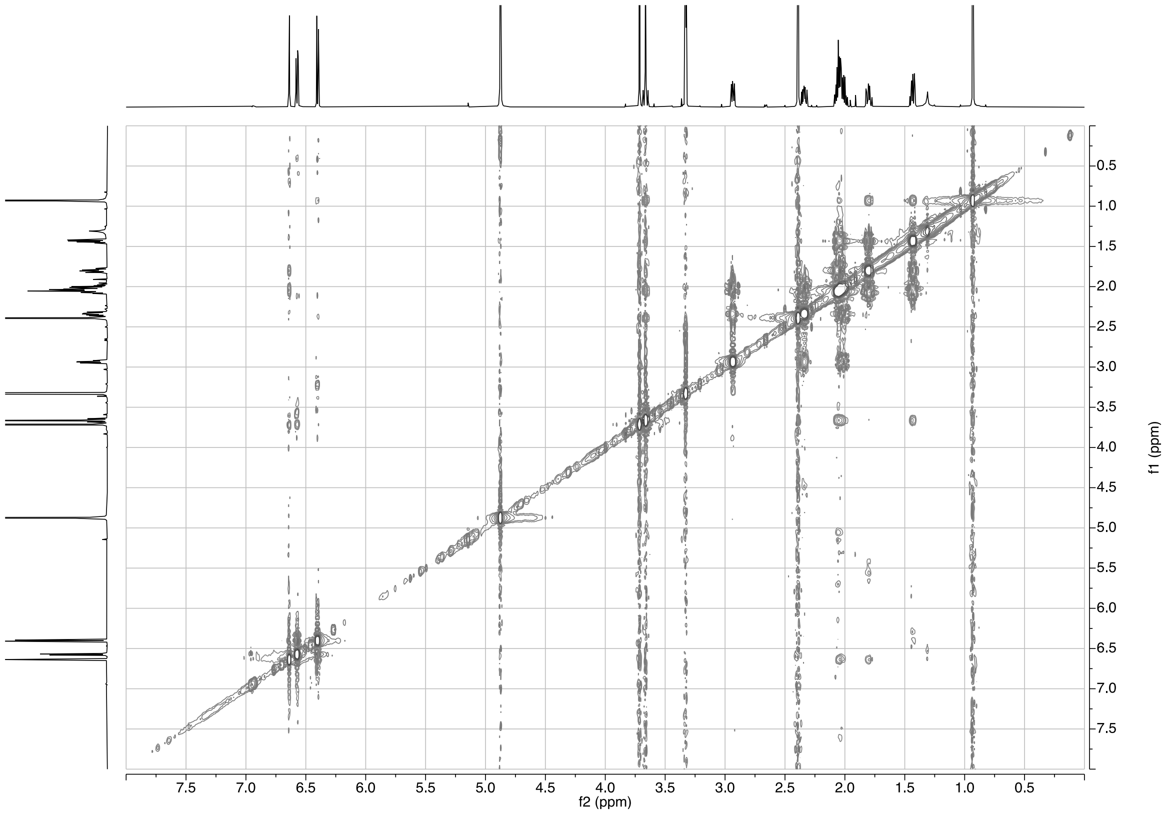

Fig. S55. NOESY spectrum of pensubrubine (**4**) in CD_3OD (600 MHz).

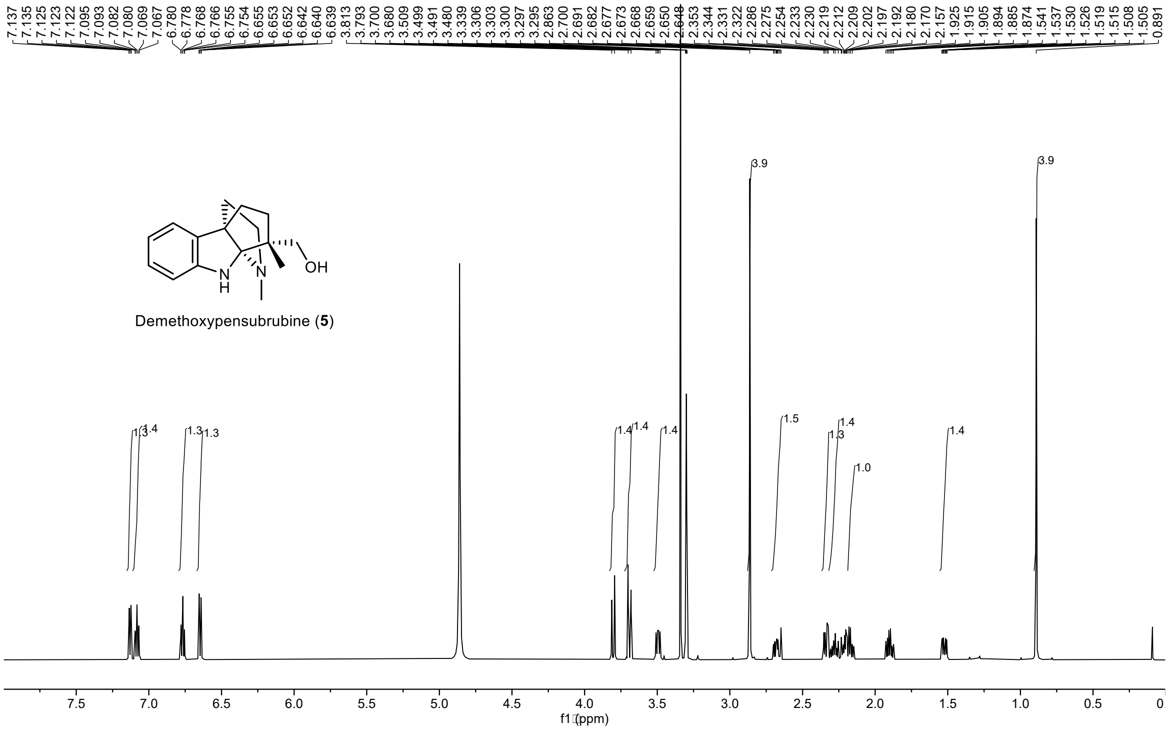

Fig. S56. ^{1}H NMR spectrum of demethoxypensubrubine (**5**) in CD_3OD (500 MHz).

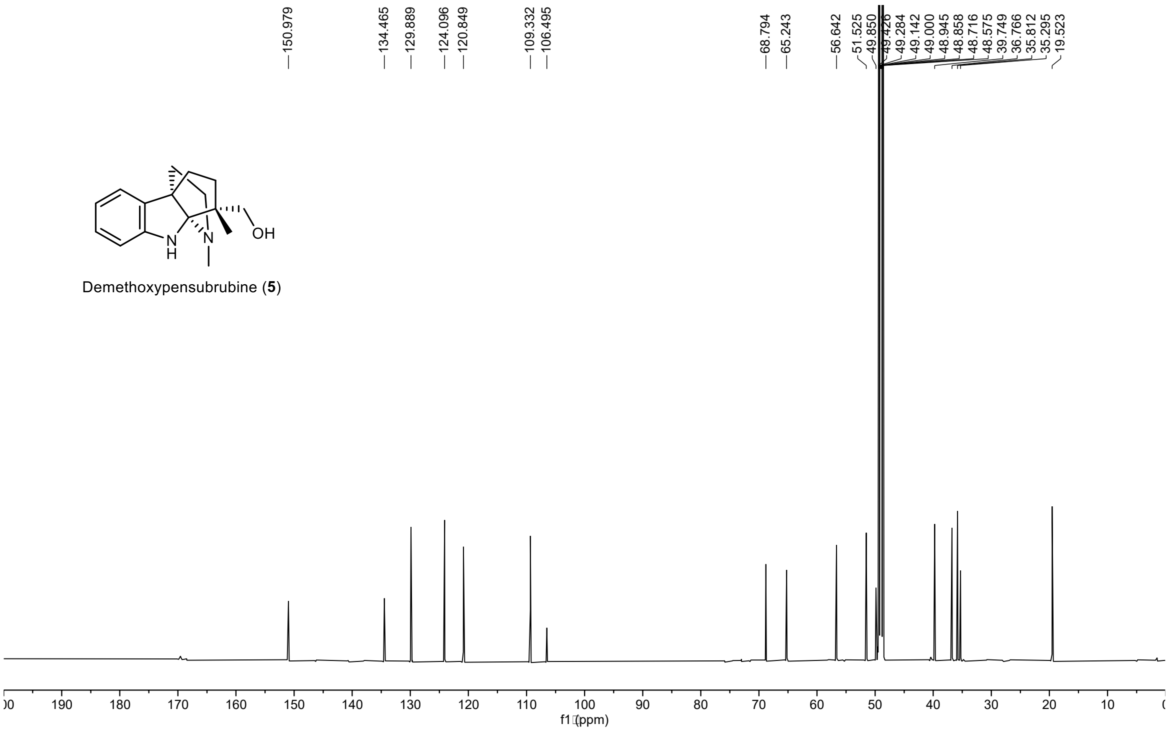

Fig. S57. ^{13}C NMR spectrum of demethoxypensubrubine (**5**) in CD_3OD (125 MHz).

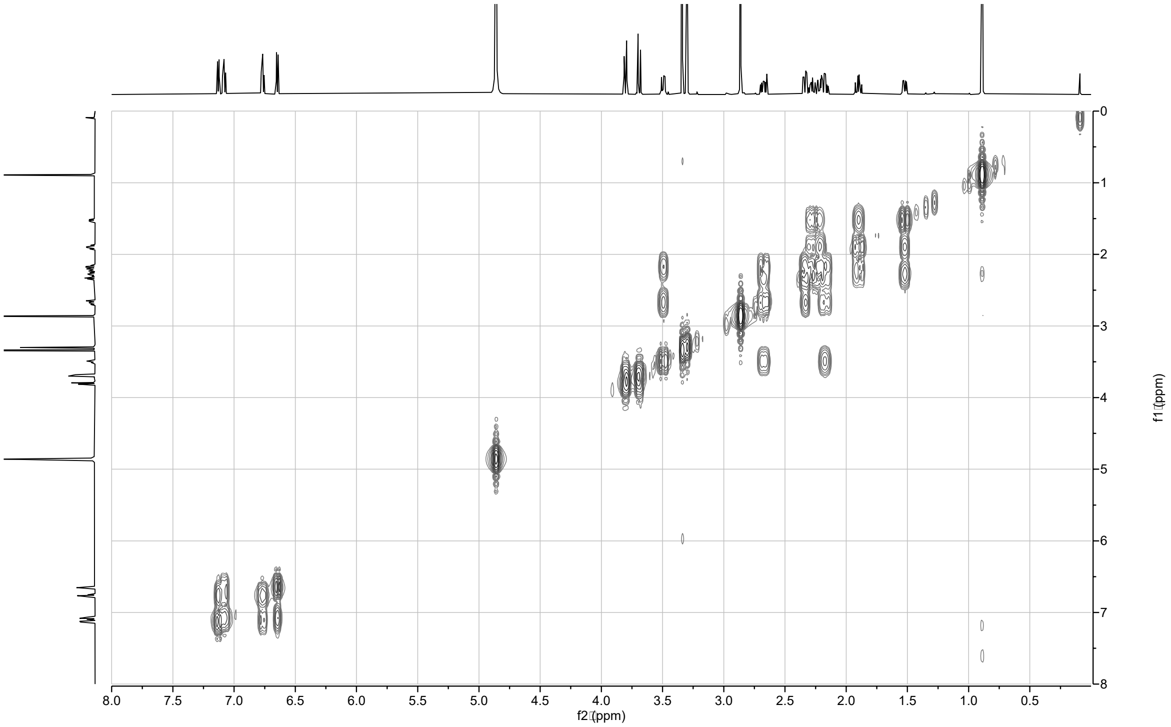

Fig. S58. ^{1}H-^{1}H COSY spectrum of demethoxypensubrubine (**5**) in CD_3OD (500 MHz).

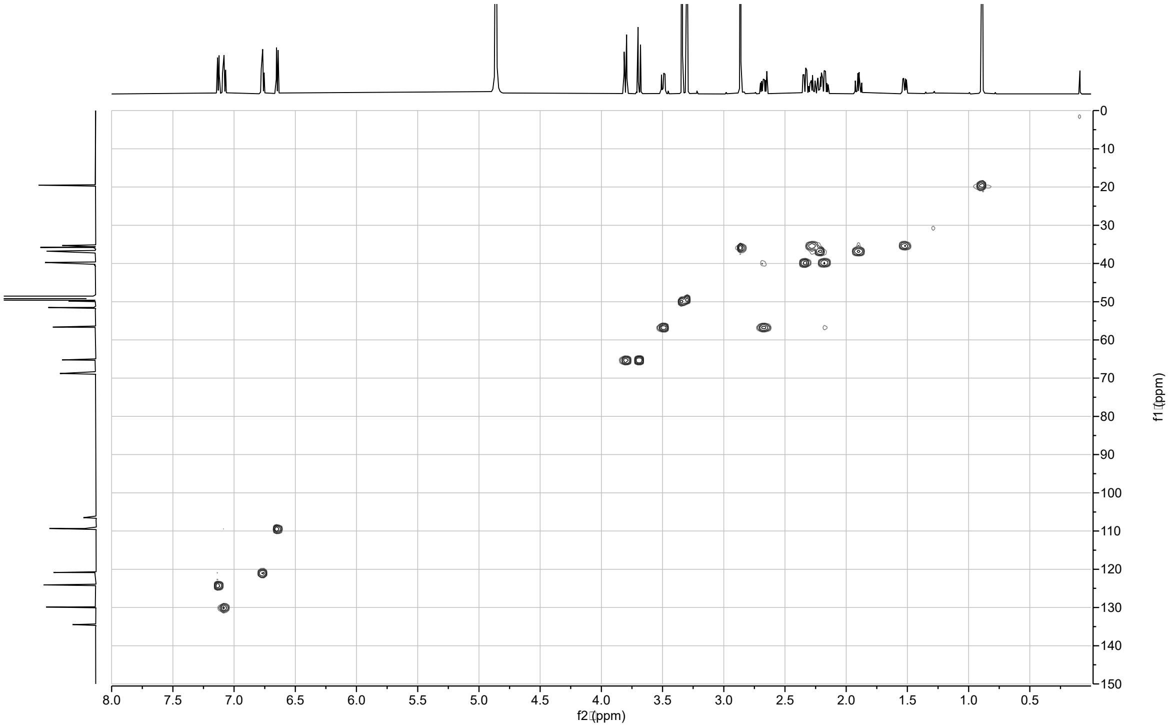

Fig. S59. HSQC spectrum of demethoxypensubrubine (**5**) in CD_3OD (500 MHz).

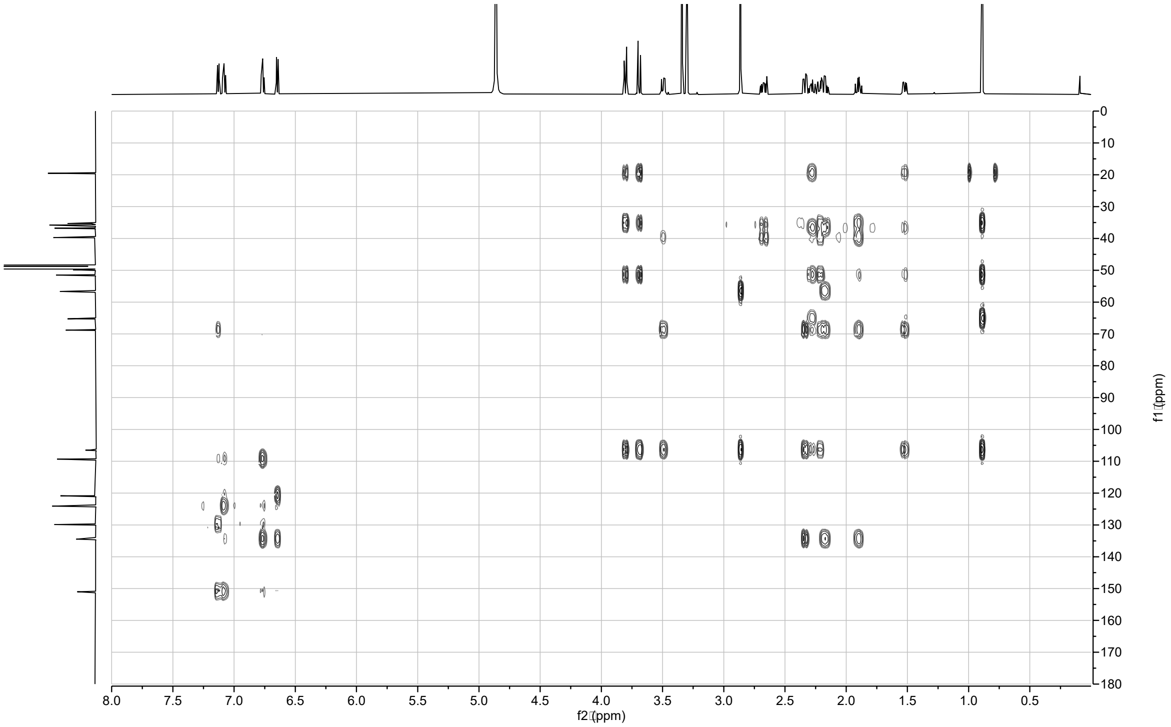

Fig. S60. HMBC spectrum of demethoxypensubrubine (**5**) in CD_3OD (500 MHz).

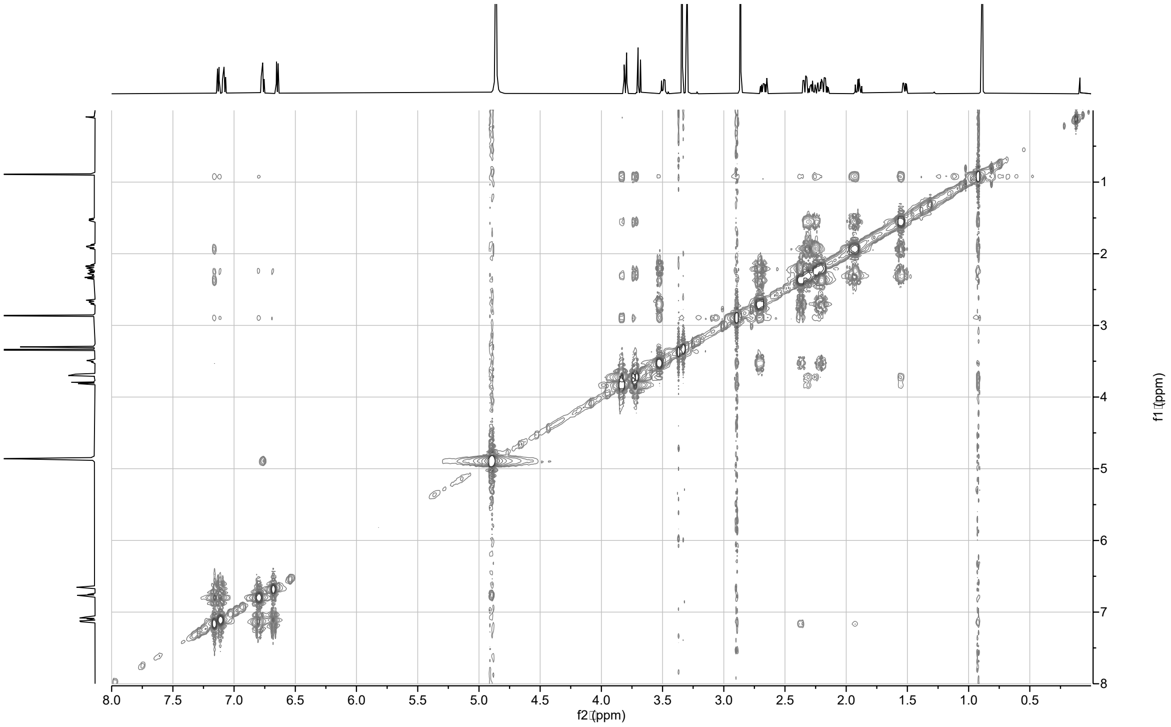

Fig. S61. NOESY spectrum of demethoxypensubrubine (**5**) in CD_3OD (500 MHz).

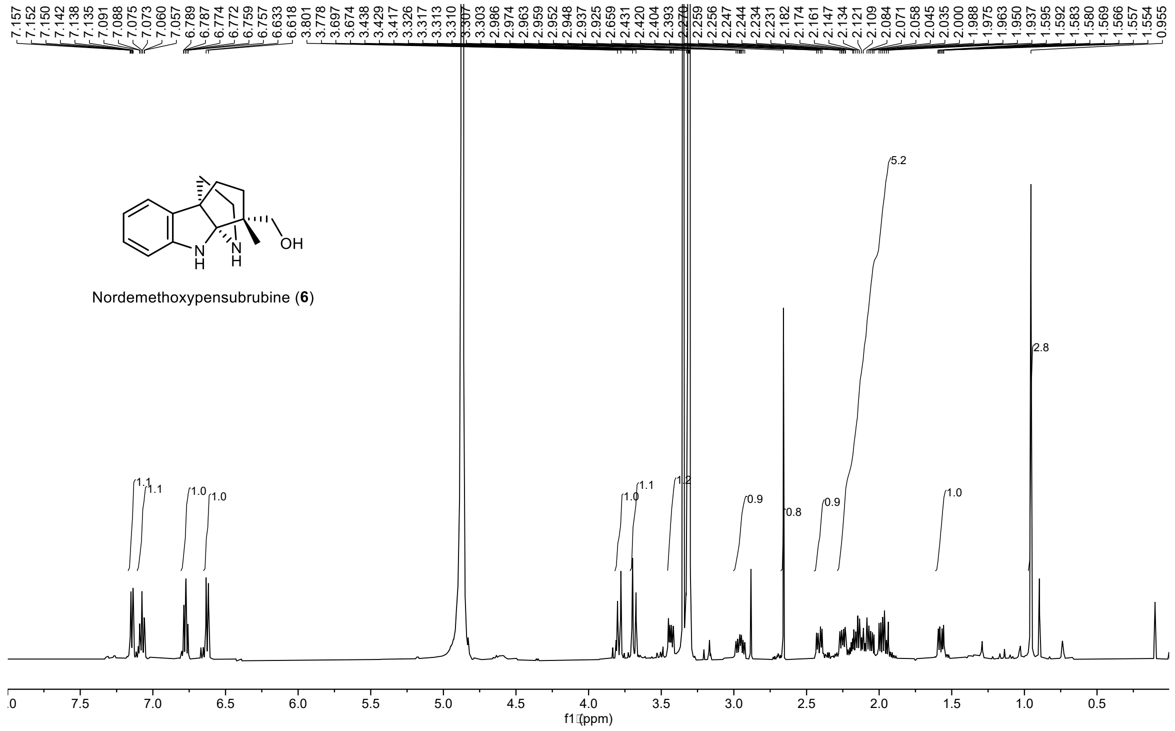

Fig. S62. ^{1}H NMR spectrum of nordemethoxypensubrubine (**6**) in CD_3OD (500 MHz).

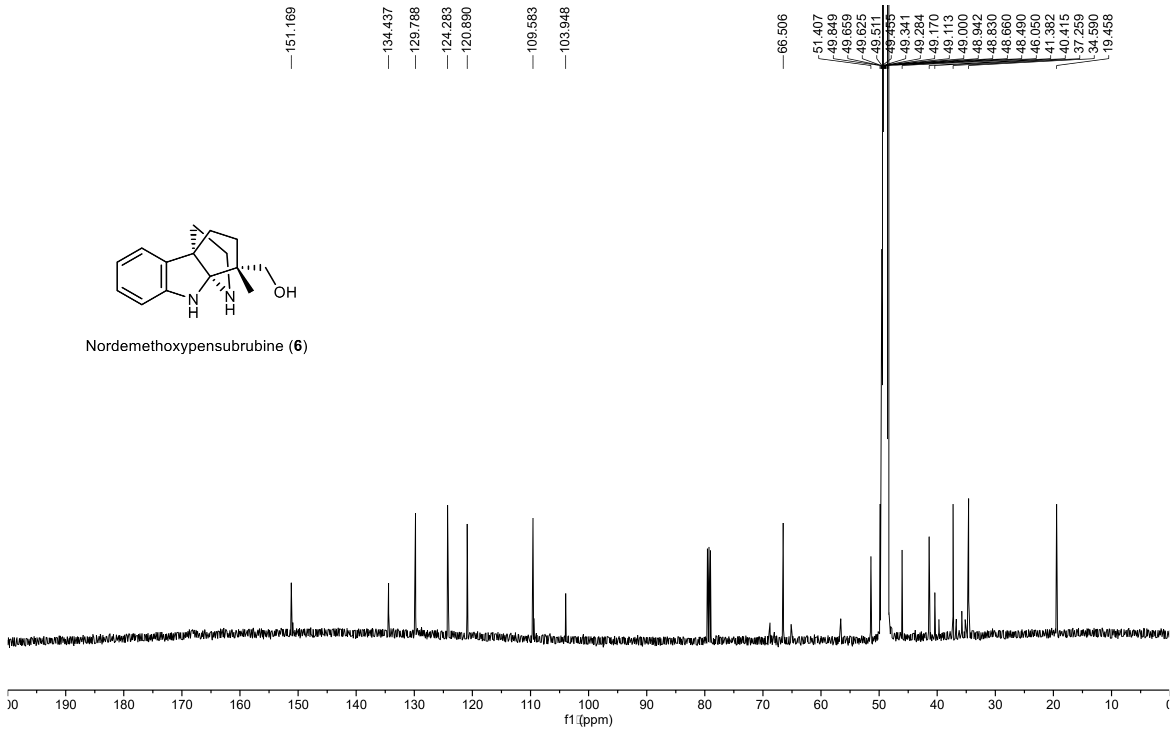

Fig. S63. ^{13}C NMR spectrum of nordemethoxypensubrubine (**6**) in CD_3OD (125 MHz).

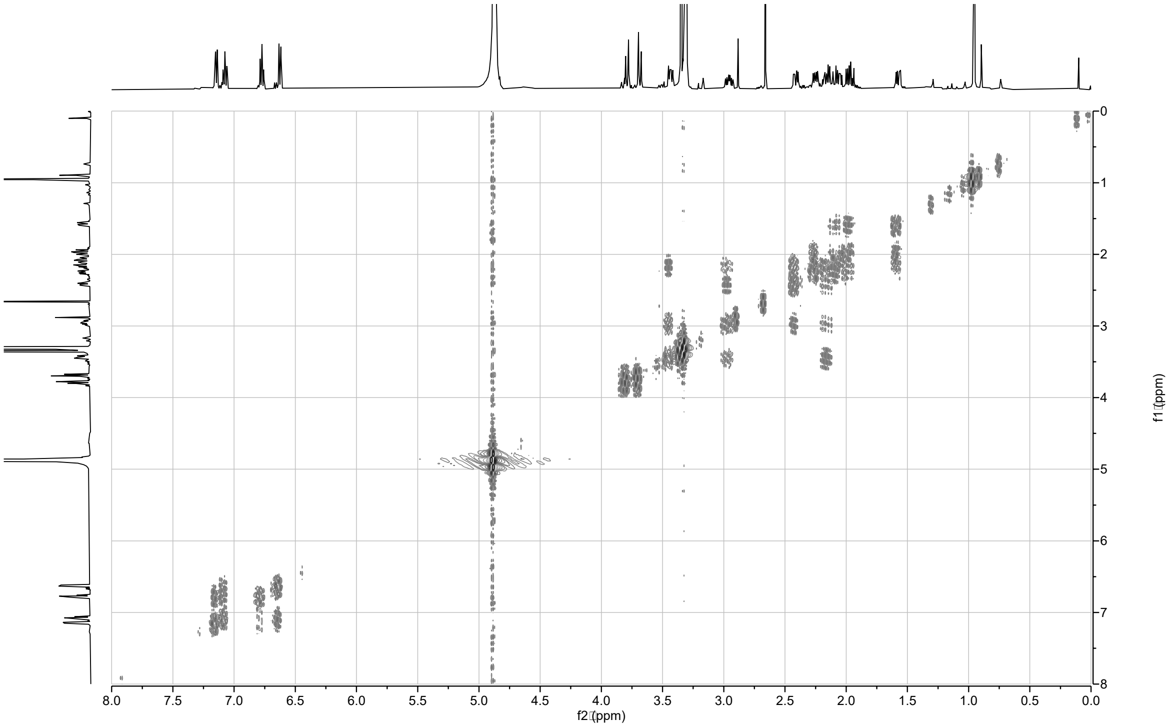

Fig. S64. ^{1}H-^{1}H COSY spectrum of nordemethoxypensubrubine (**6**) in CD_3OD (500 MHz).

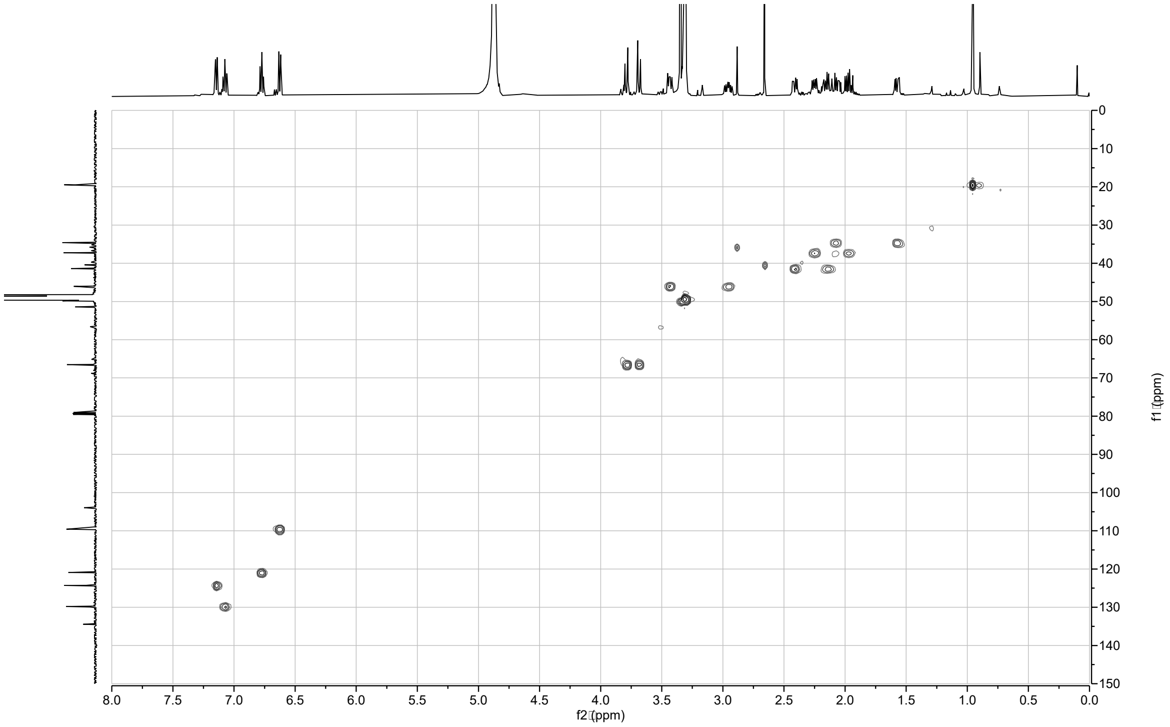

Fig. S65. HSQC spectrum of nordemethoxypensubrubine (**6**) in CD_3OD (500 MHz).

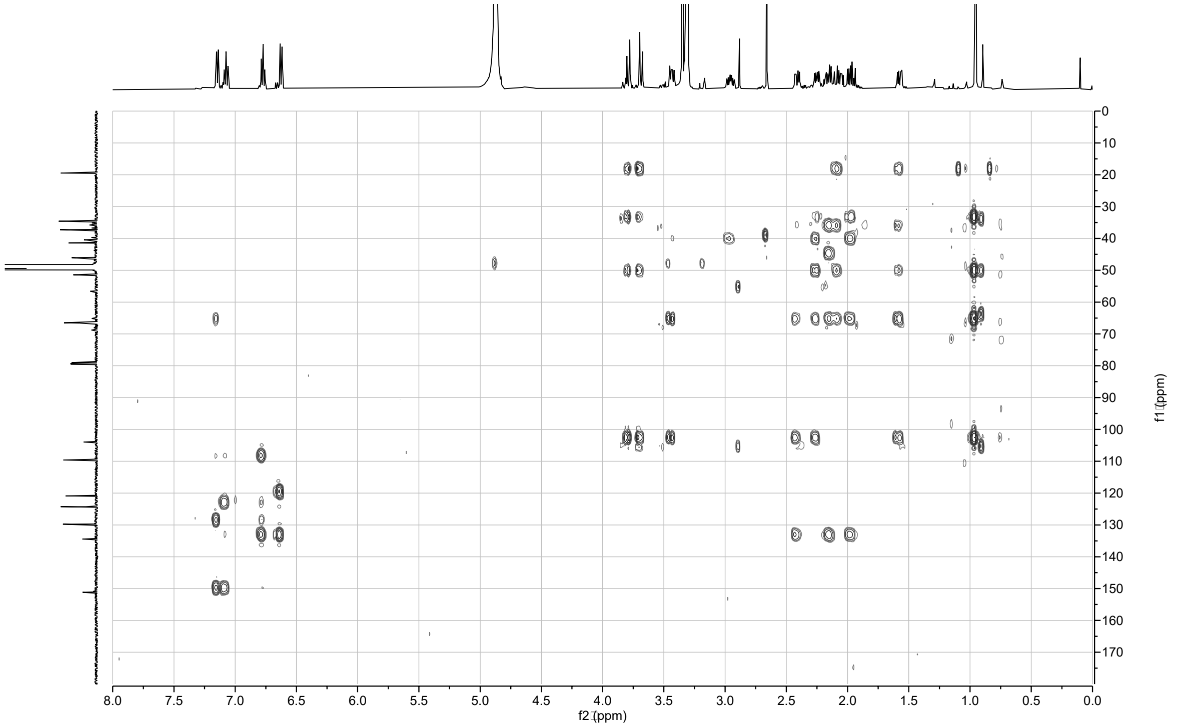

Fig. S66. HMBC spectrum of nordemethoxypensubrubine (**6**) in CD_3OD (500 MHz).

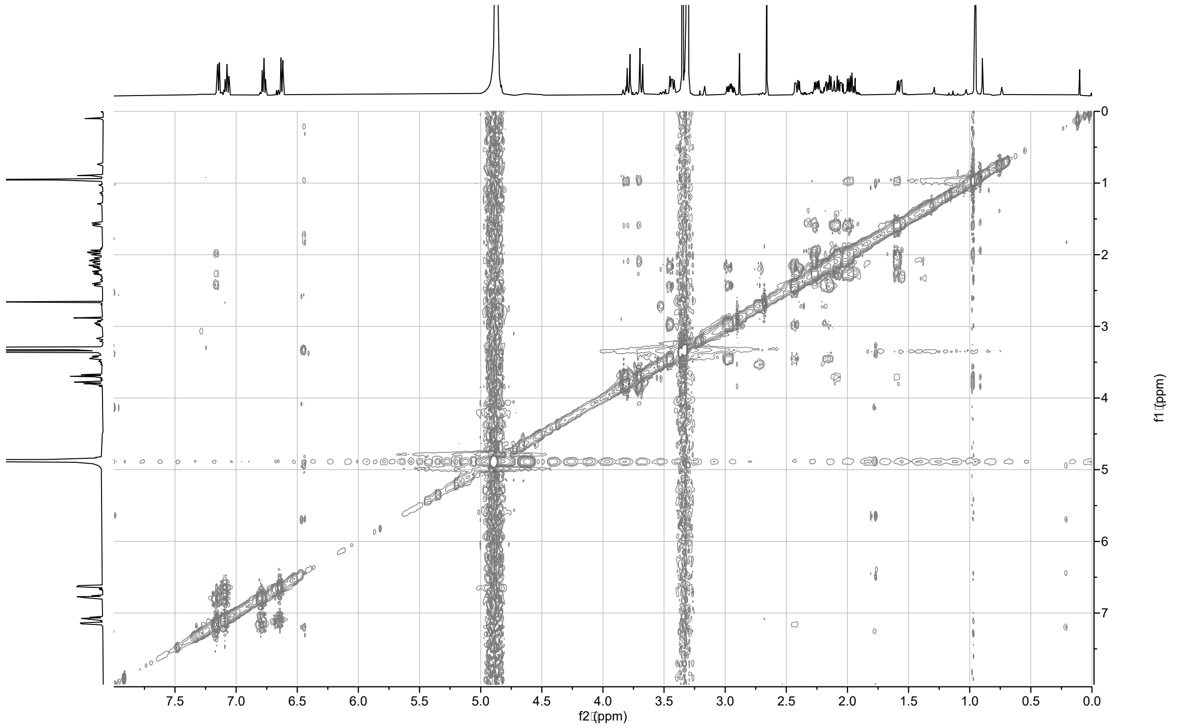

Fig. S67. NOESY spectrum of nordemethoxypensubrubine (**6**) in CD_3OD (500 MHz).

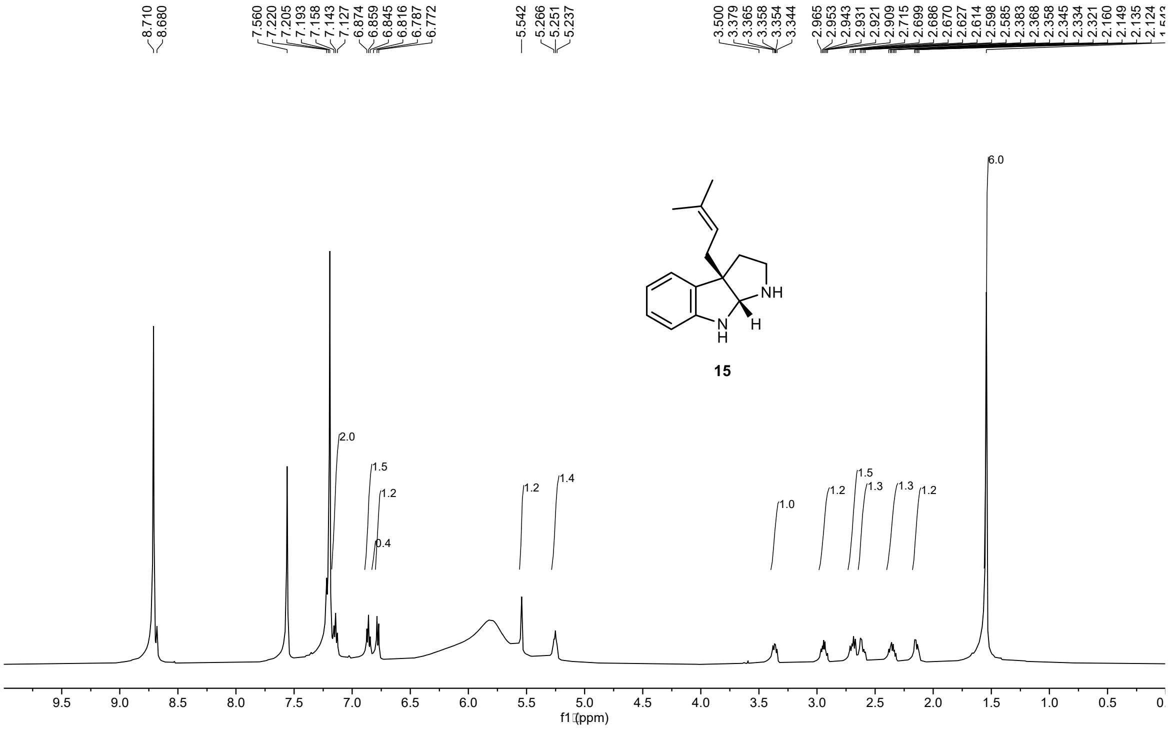

Fig. S68. ^{1}H NMR spectrum of **15** in pyridine-d_5 (500 MHz).

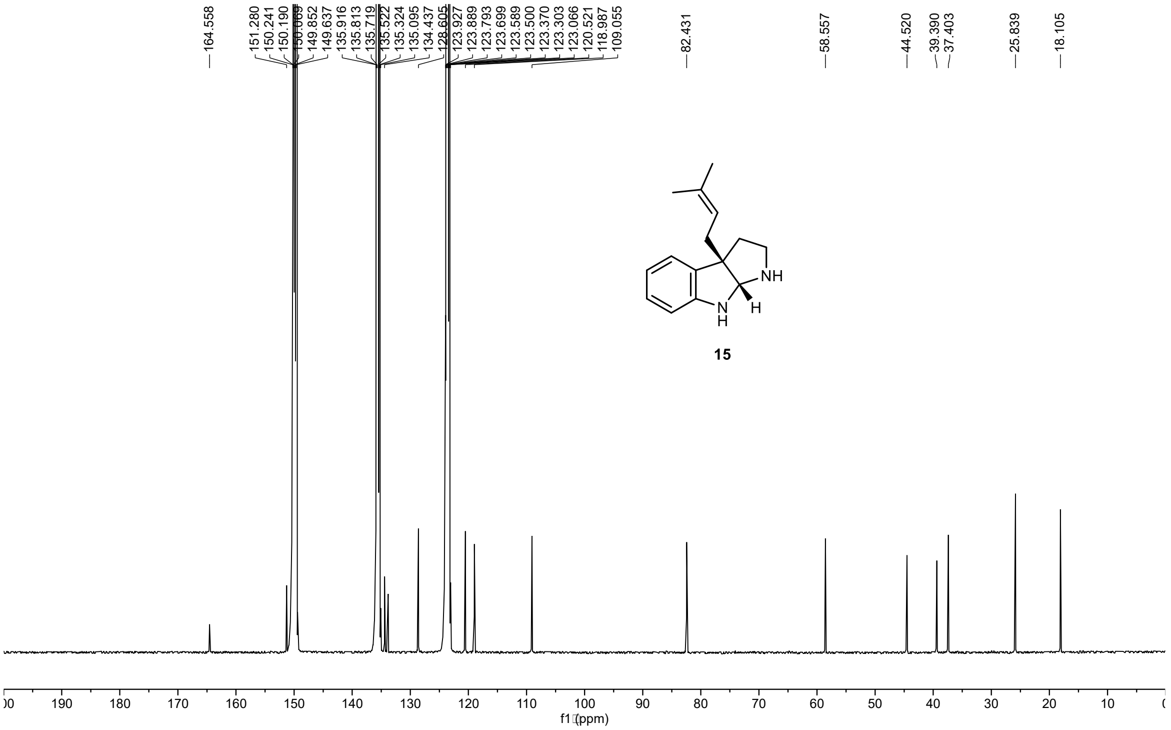

Fig. S69. ^{13}C NMR spectrum of **15** in pyridine-d_5 (125 MHz).

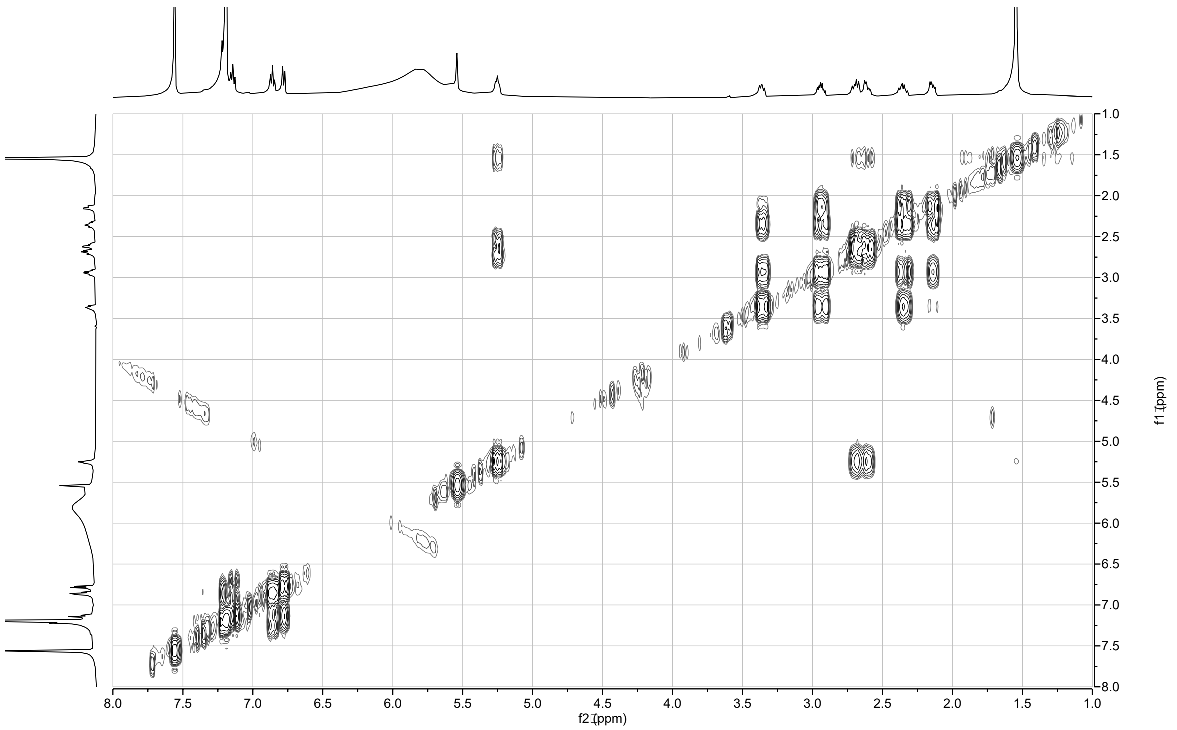

Fig. S70. ^{1}H-^{1}H COSY spectrum of **15** in pyridine-d_5 (500 MHz).

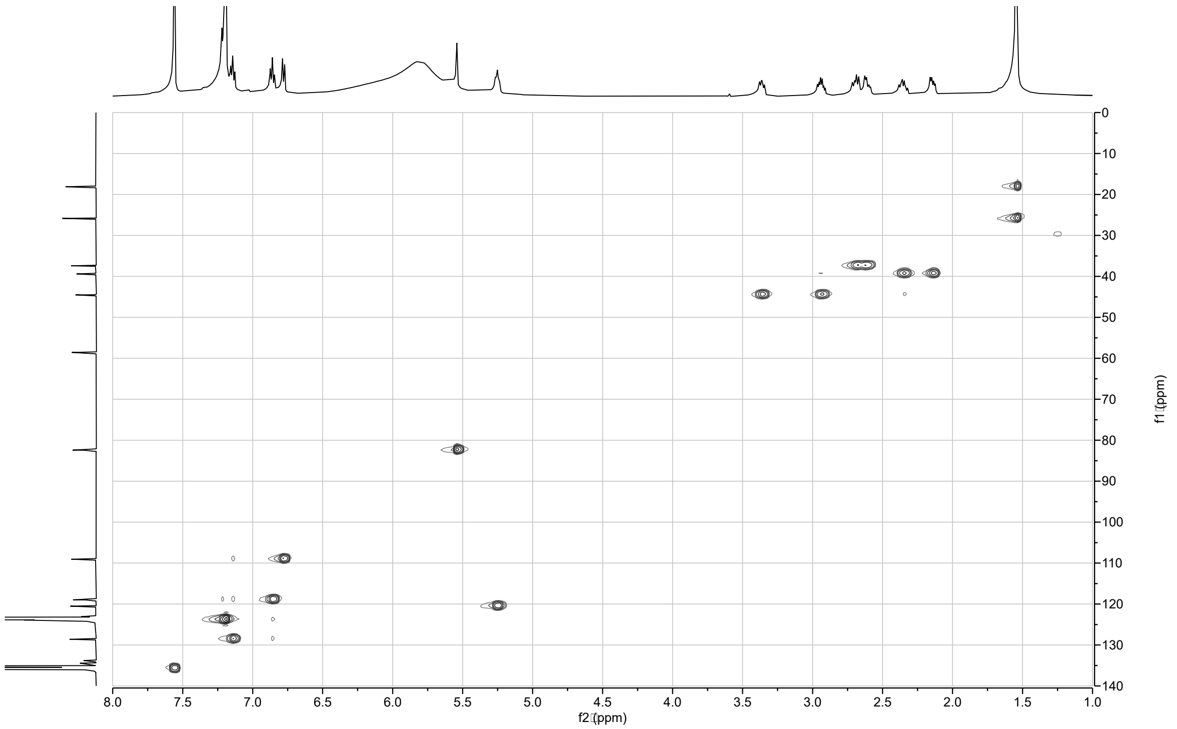

Fig. S71. HSQC spectrum of **15** in pyridine-d_5 (500 MHz).

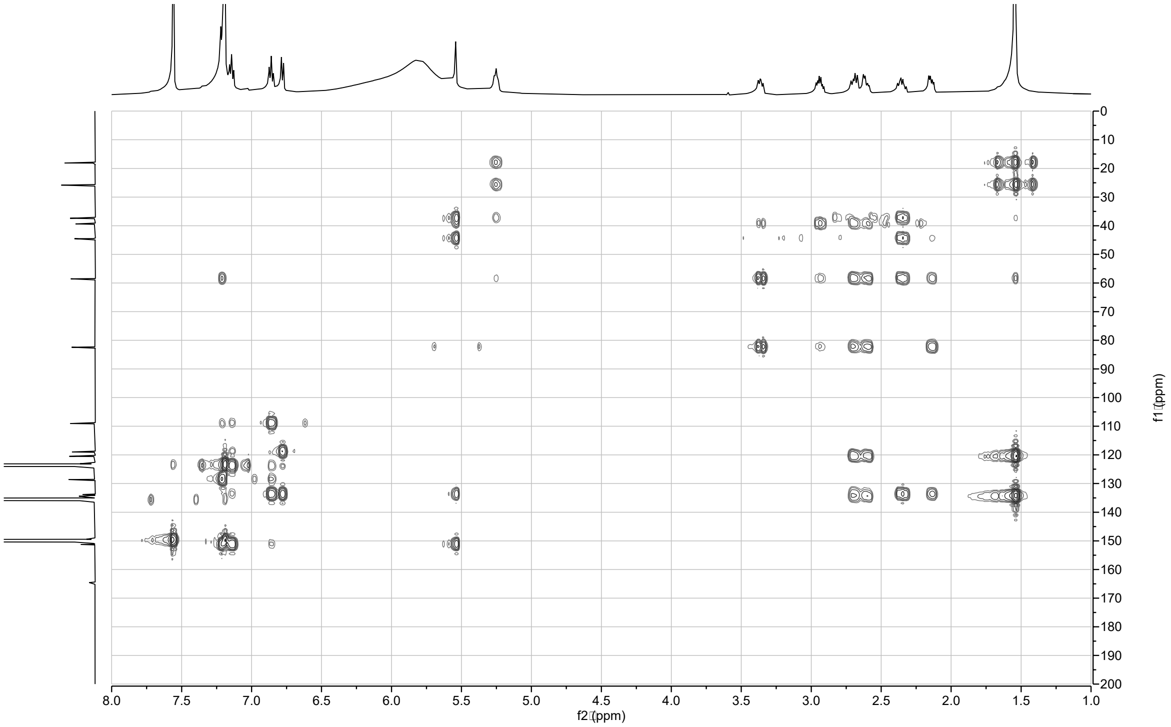

Fig. S72. HMBC spectrum of **15** in pyridine-d_5 (500 MHz).

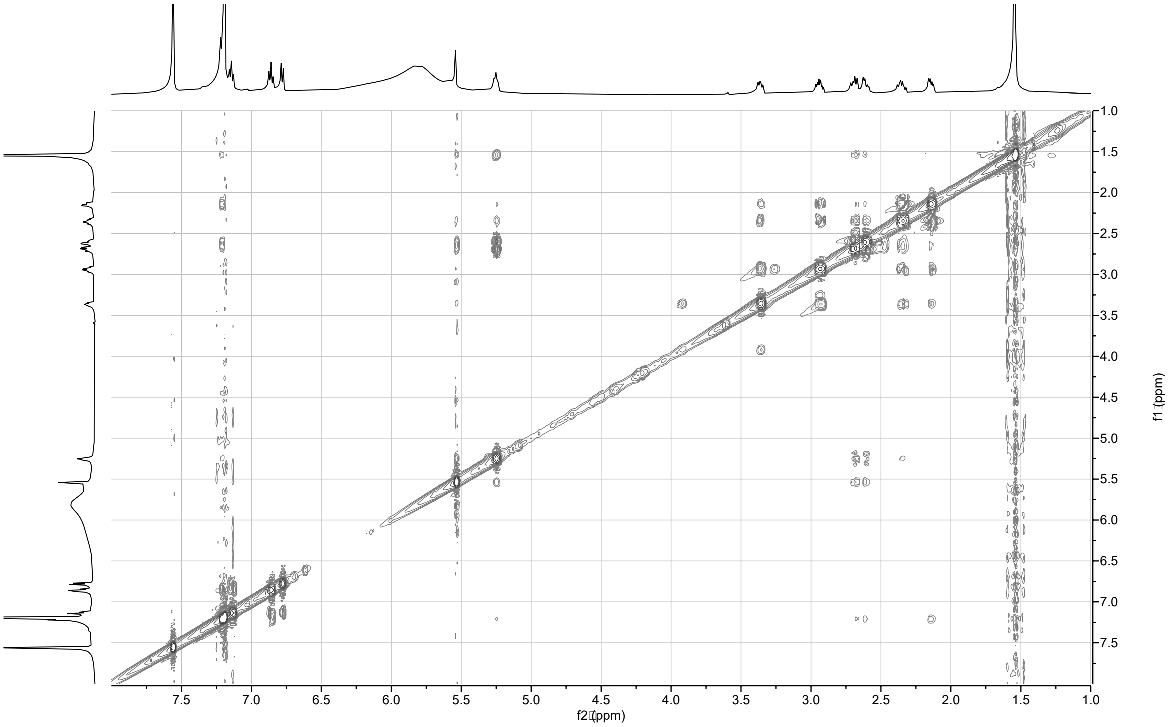

Fig. S73. ROESY spectrum of **15** in pyridine-d_5 (500 MHz).

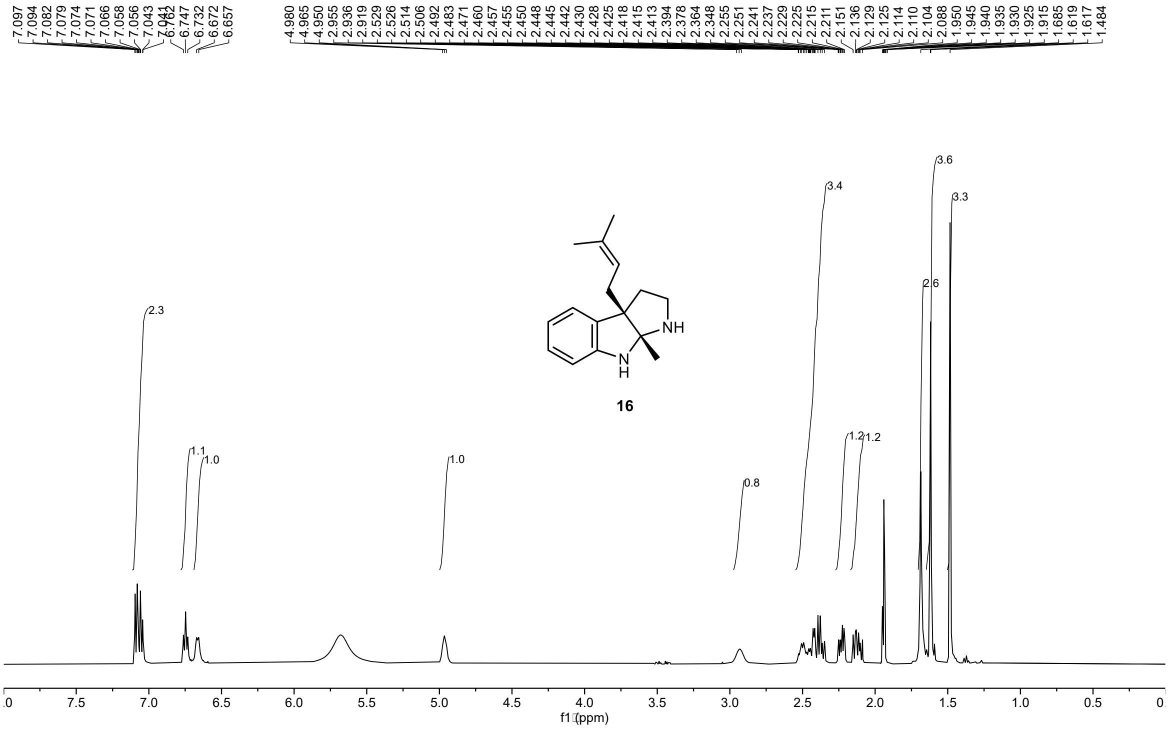

Fig. S74. ^{1}H NMR spectrum of **16** in acetonitrile-d_3 (500 MHz).

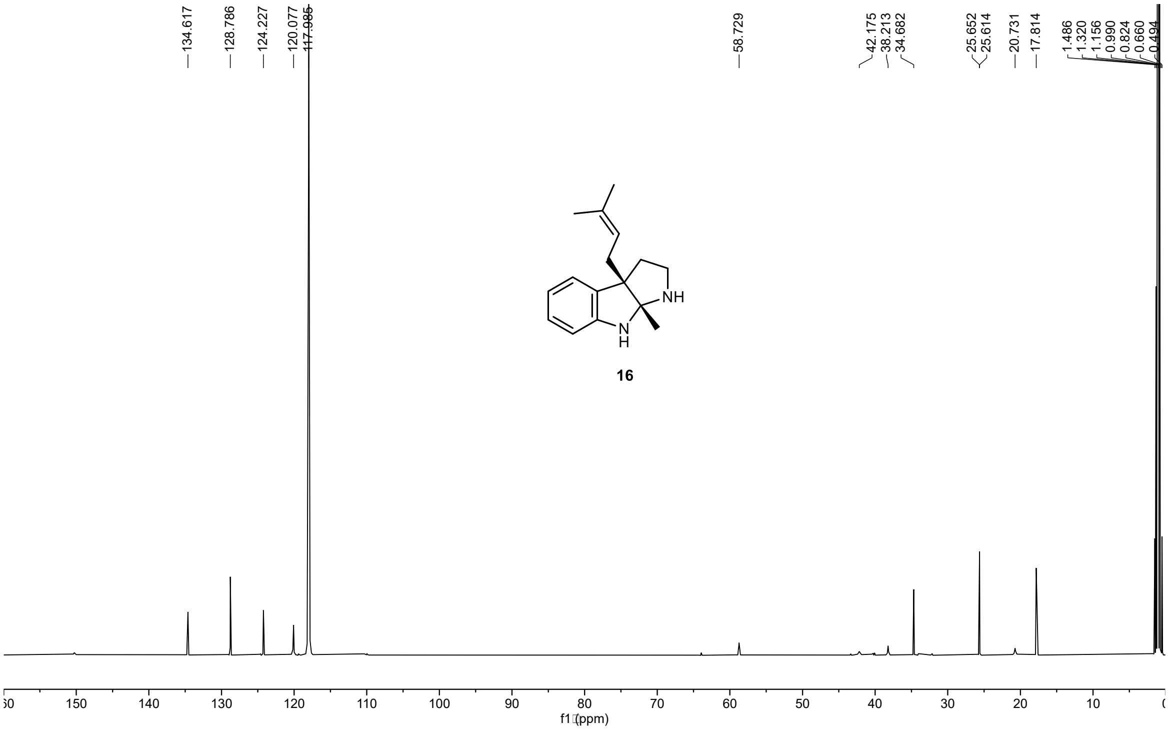

Fig. S75. ^{13}C NMR spectrum of **16** in acetonitrile-d_3 (125 MHz).

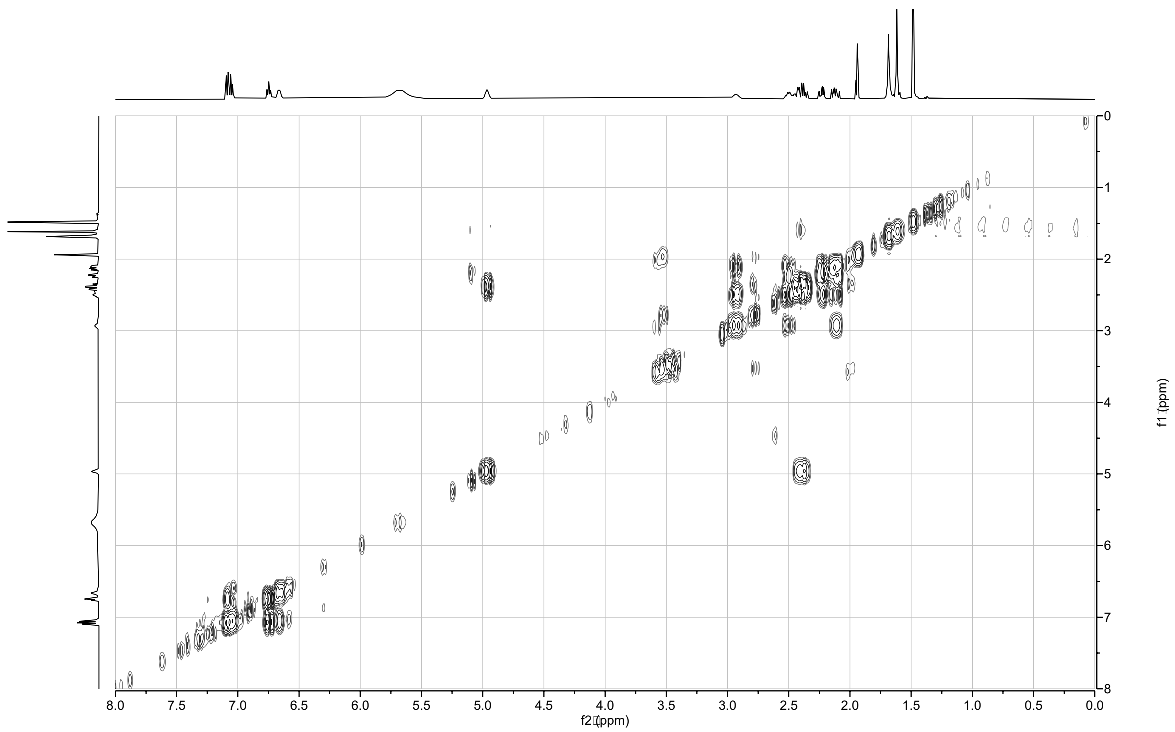

Fig. S76. ^{1}H-^{1}H COSY spectrum of **16** in acetonitrile-*d*3 (500 MHz).

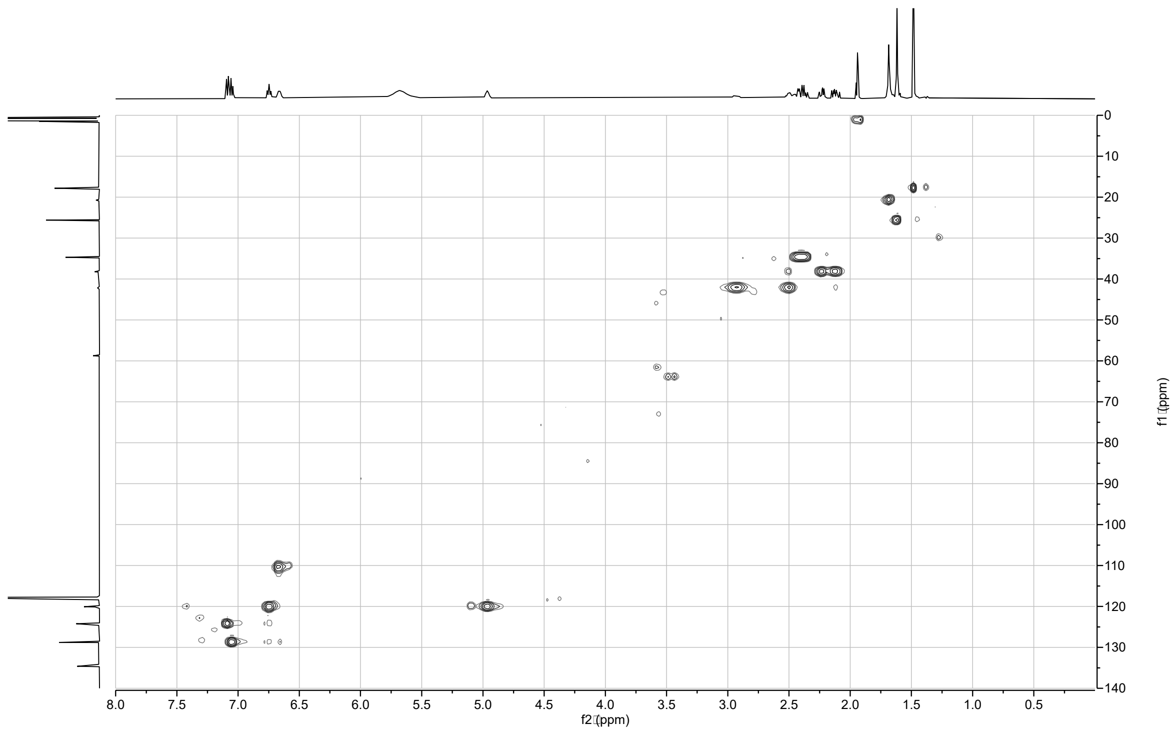

Fig. S77. HSQC spectrum of **16** in acetonitrile-*d*3 (500 MHz).

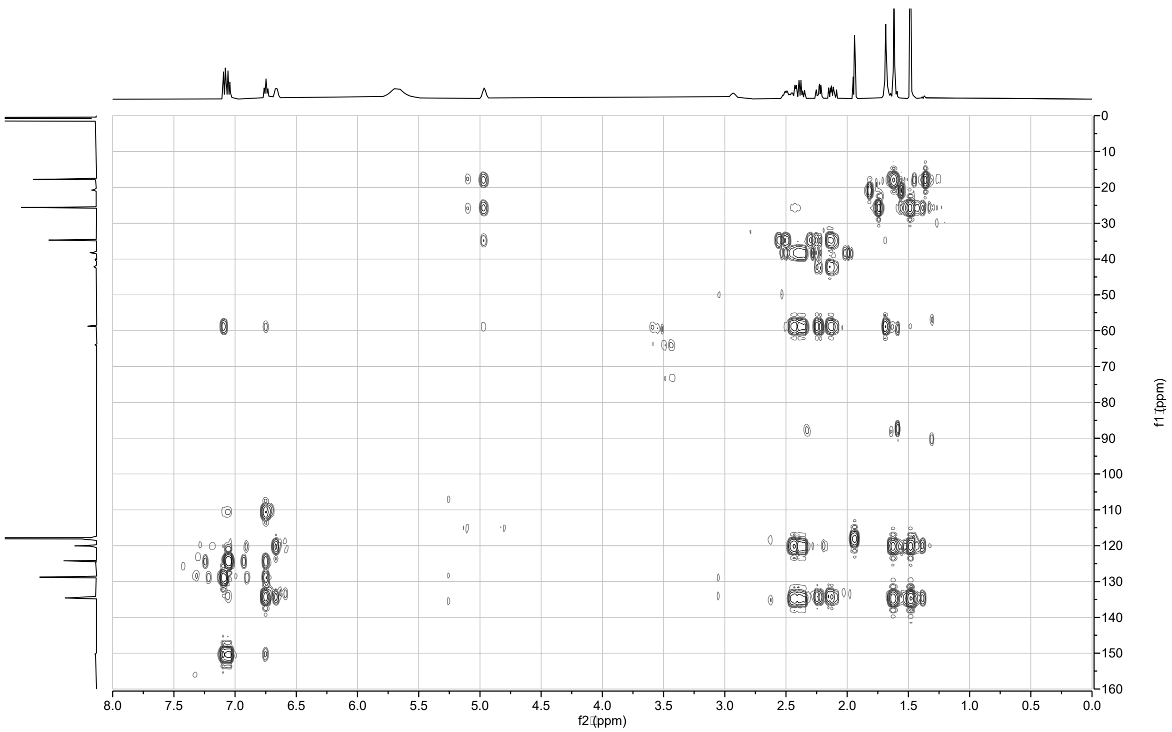

Fig. S78. HMBC spectrum of **16** in acetonitrile-d_3 (500 MHz).

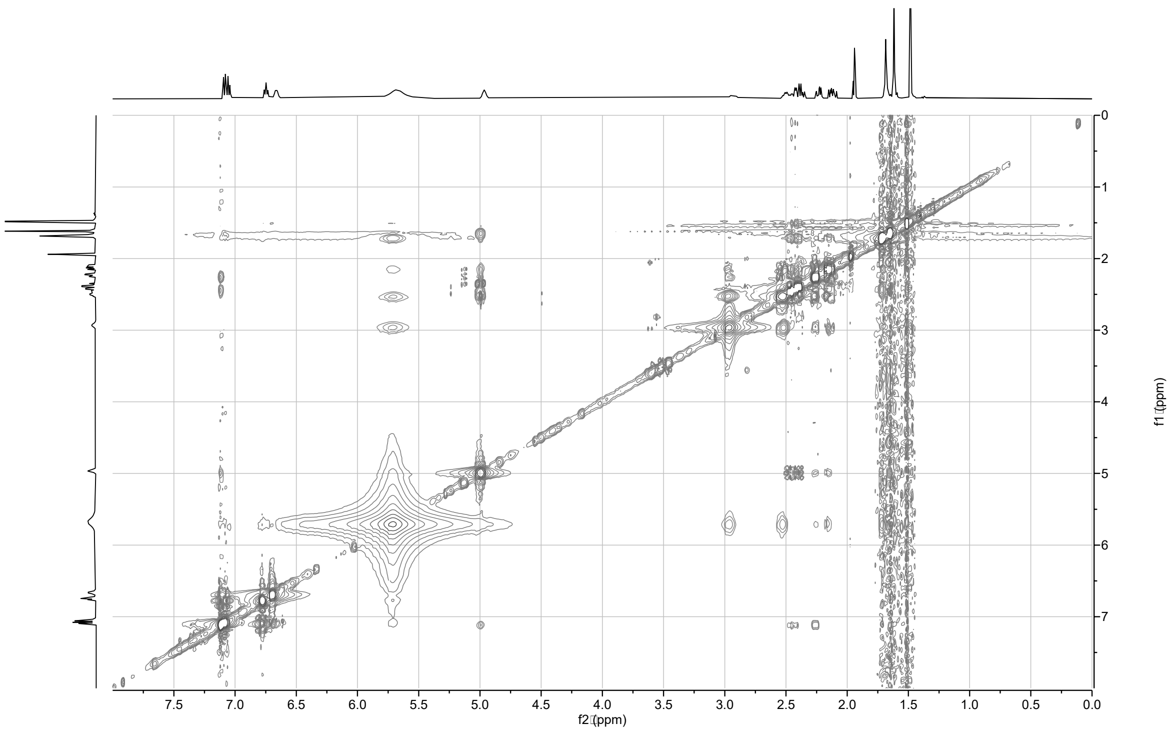

Fig. S79. NOESY spectrum of **16** in acetonitrile-d_3 (500 MHz).

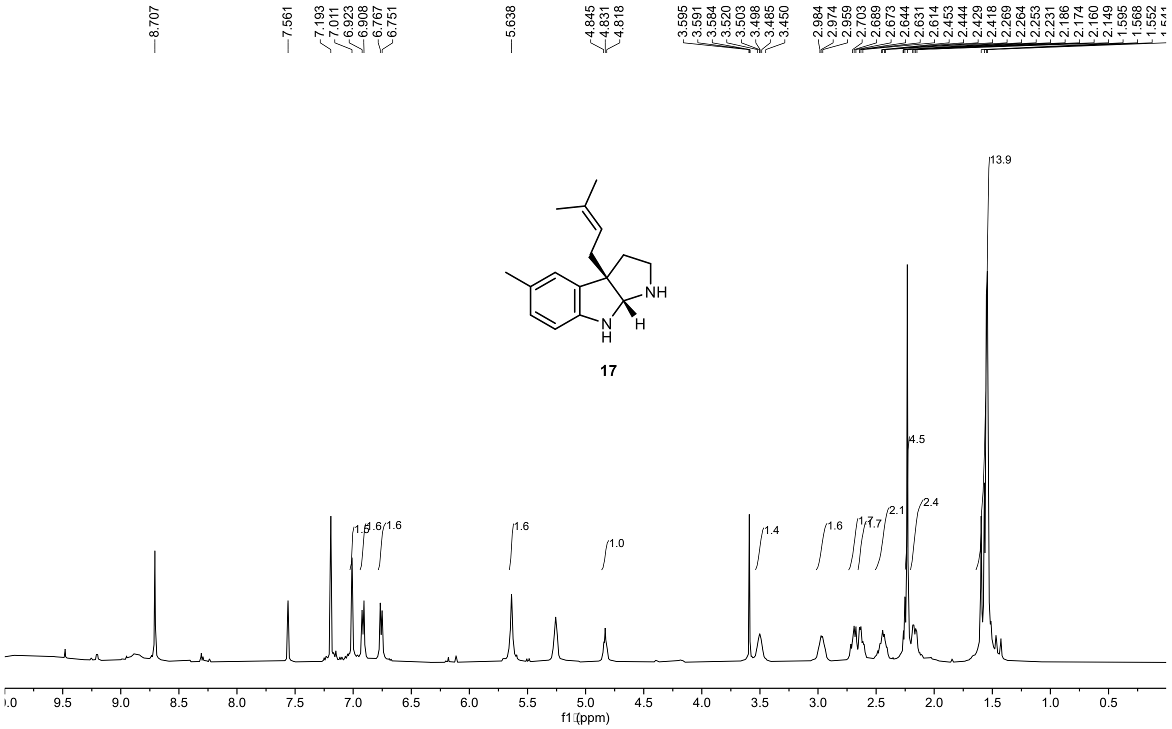

Fig. S80. ^{1}H NMR spectrum of **17** in pyridine-d_5 (500 MHz).

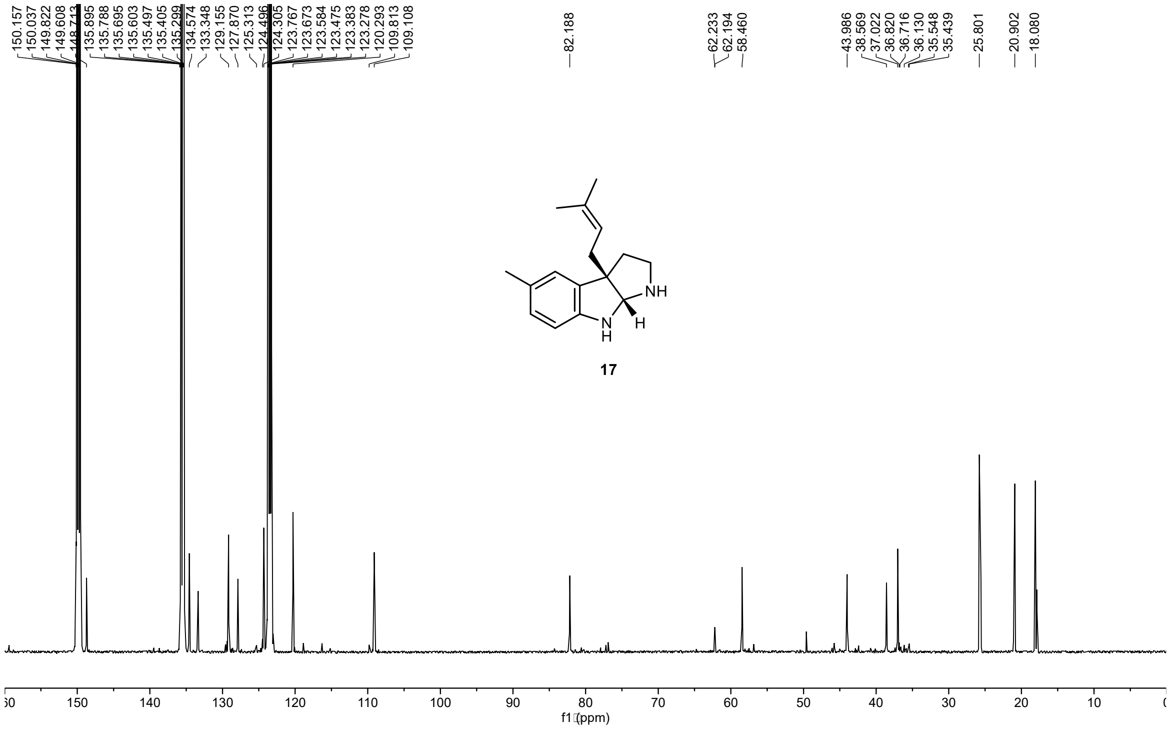

Fig. S81. ^{13}C NMR spectrum of **17** in pyridine-d_5 (125 MHz).

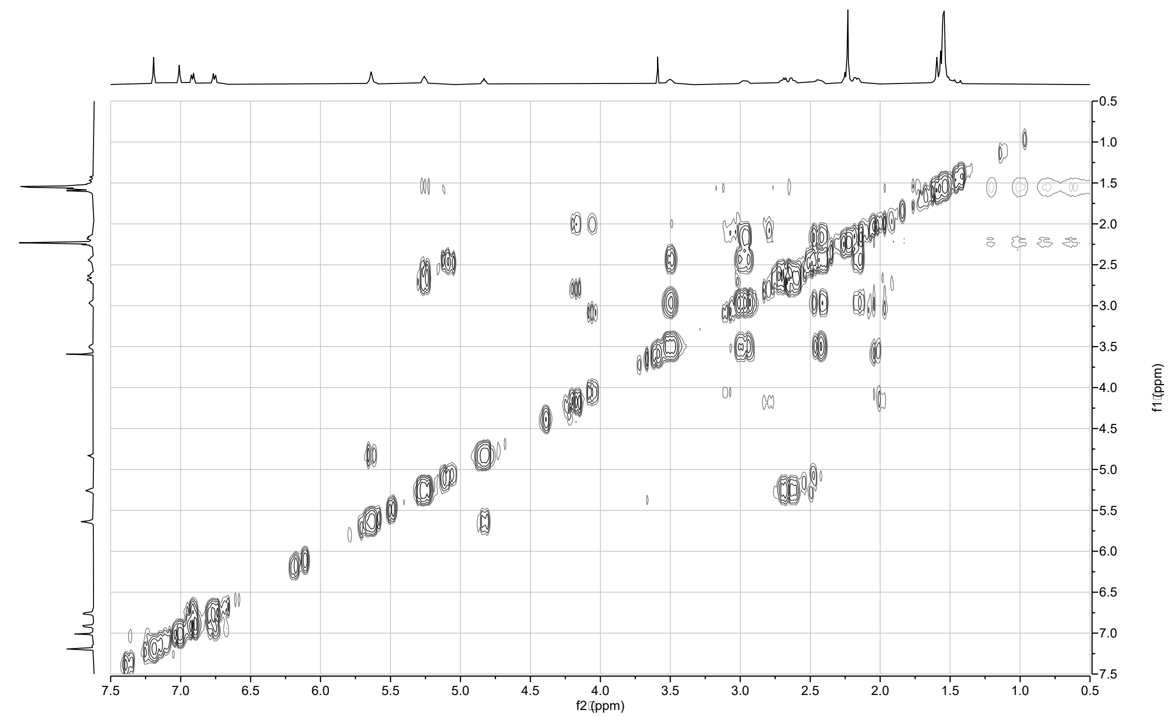

Fig. S82. ^1H-^1H COSY spectrum of **17** in pyridine-d_5 (500 MHz).

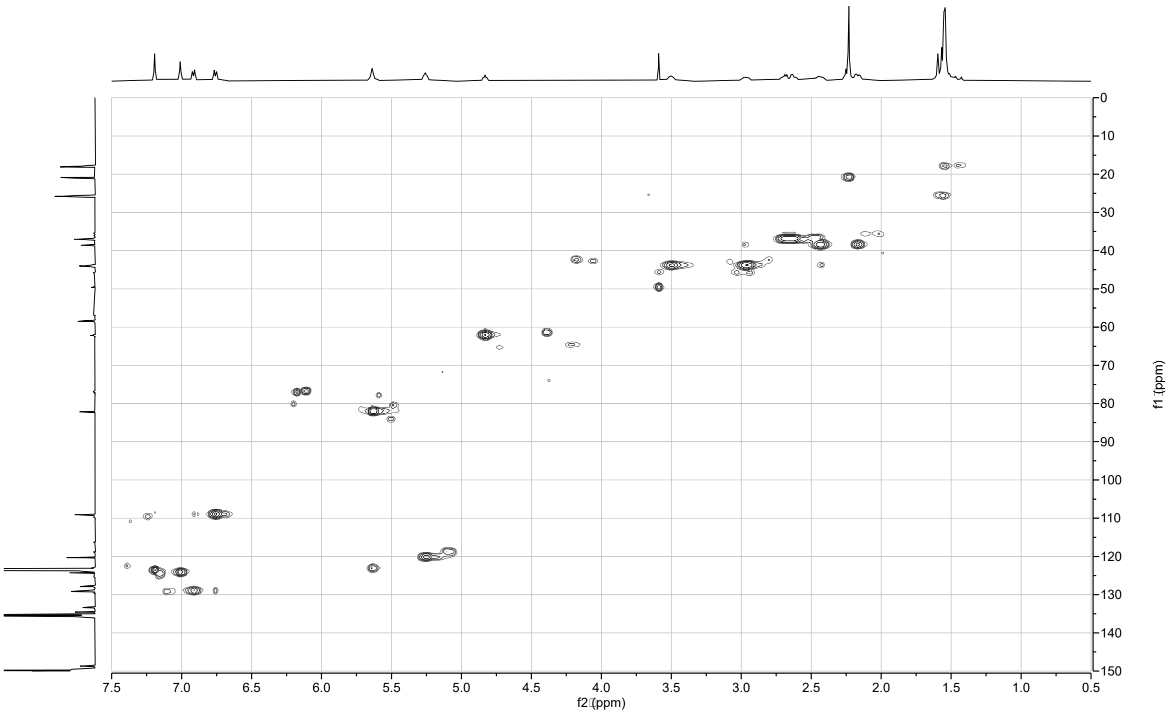

Fig. S83. HSQC spectrum of **17** in pyridine-d_5 (500 MHz).

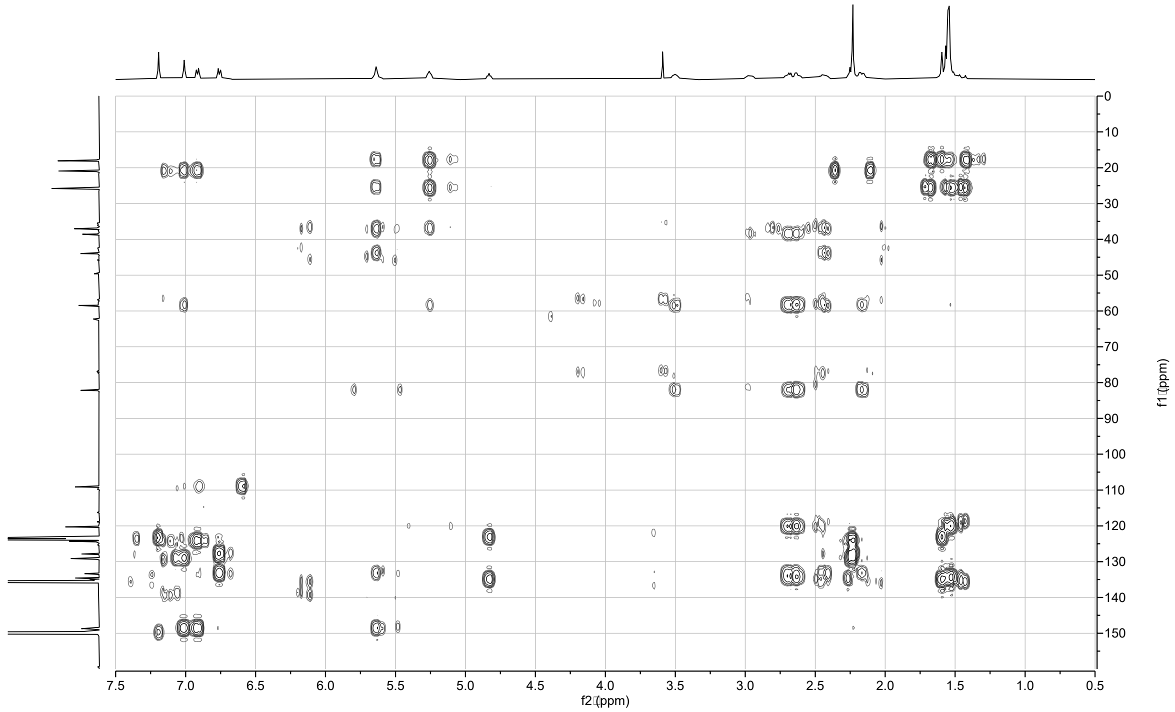

Fig. S84. HMBC spectrum of **17** in pyridine-d_5 (500 MHz).

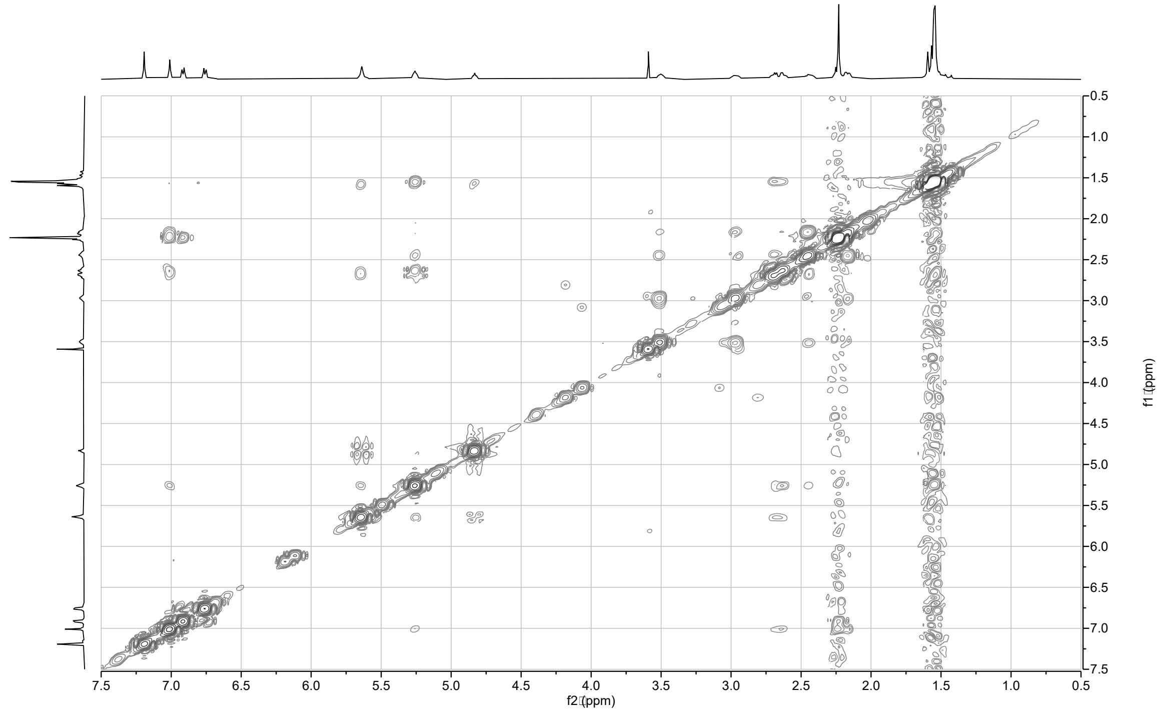

Fig. S85. ROESY spectrum of **17** in pyridine-d_5 (500 MHz).

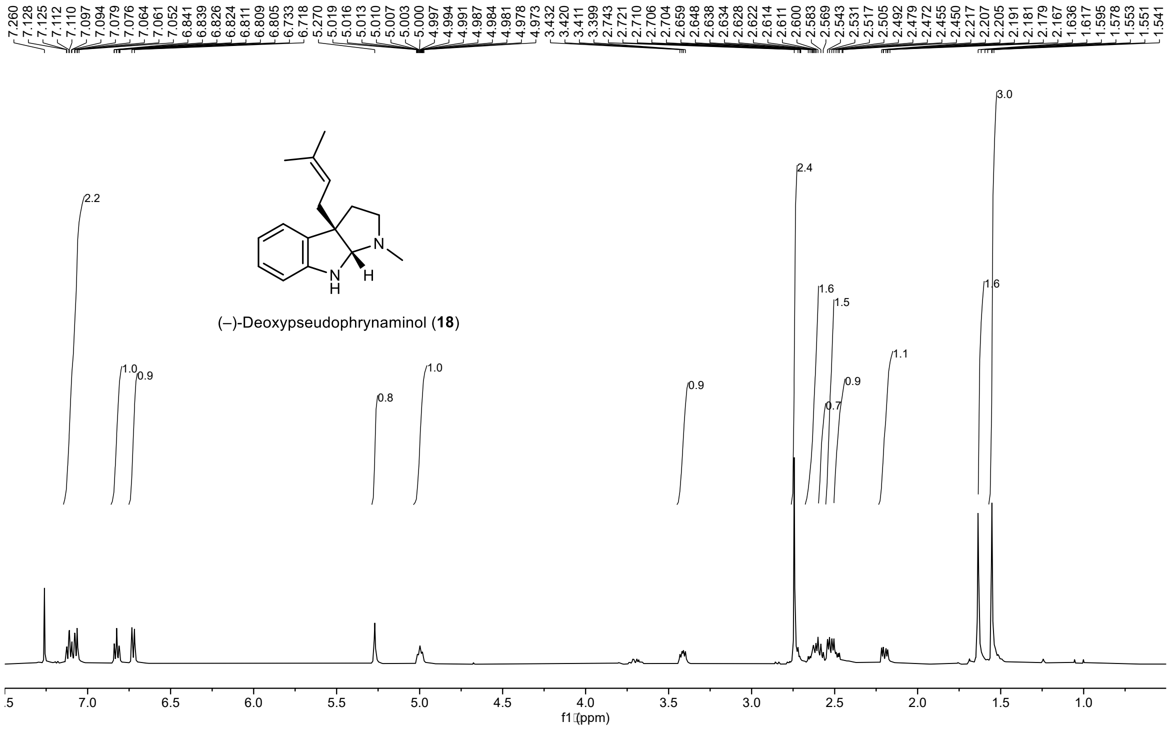

Fig. S86. ^{1}H NMR spectrum of (–)-deoxypseudophrynaminol (**18**) in $CDCl_3$ (500 MHz).

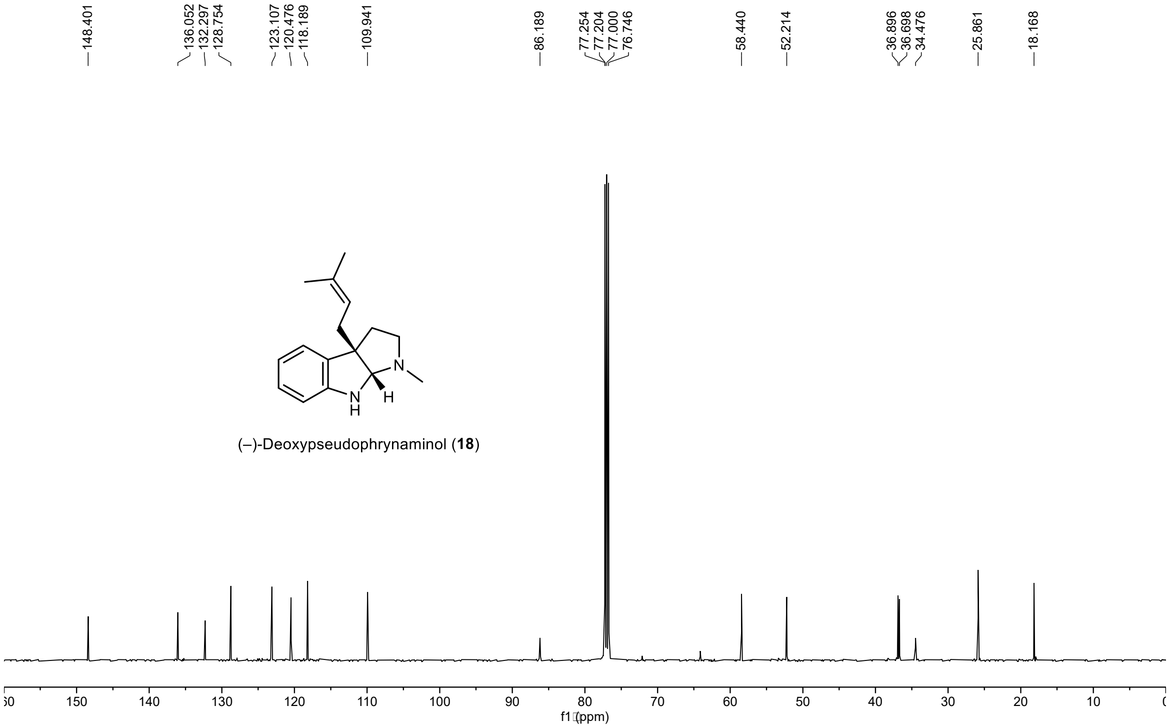

Fig. S87. ^{13}C NMR spectrum of (–)-deoxypseudophrynaminol (**18**) in $CDCl_3$ (500 MHz).

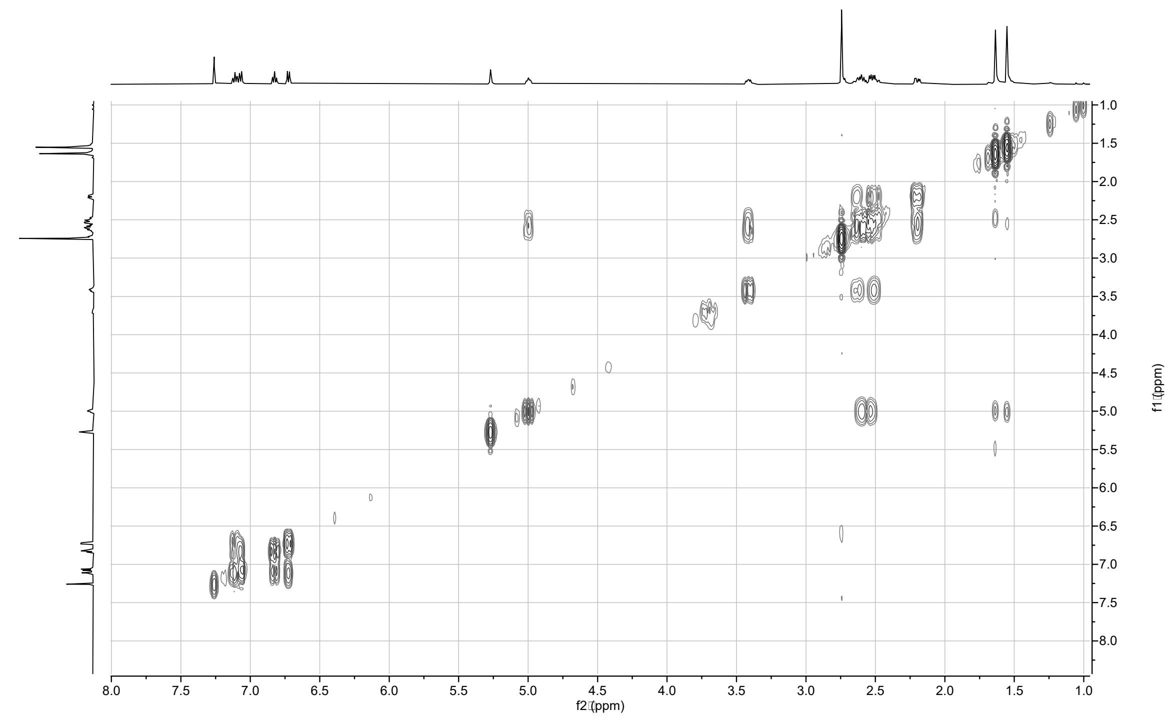

Fig. S88. ^{1}H-^{1}H COSY spectrum of (–)-deoxypseudophrynaminol (**18**) in $CDCl_3$ (500 MHz).

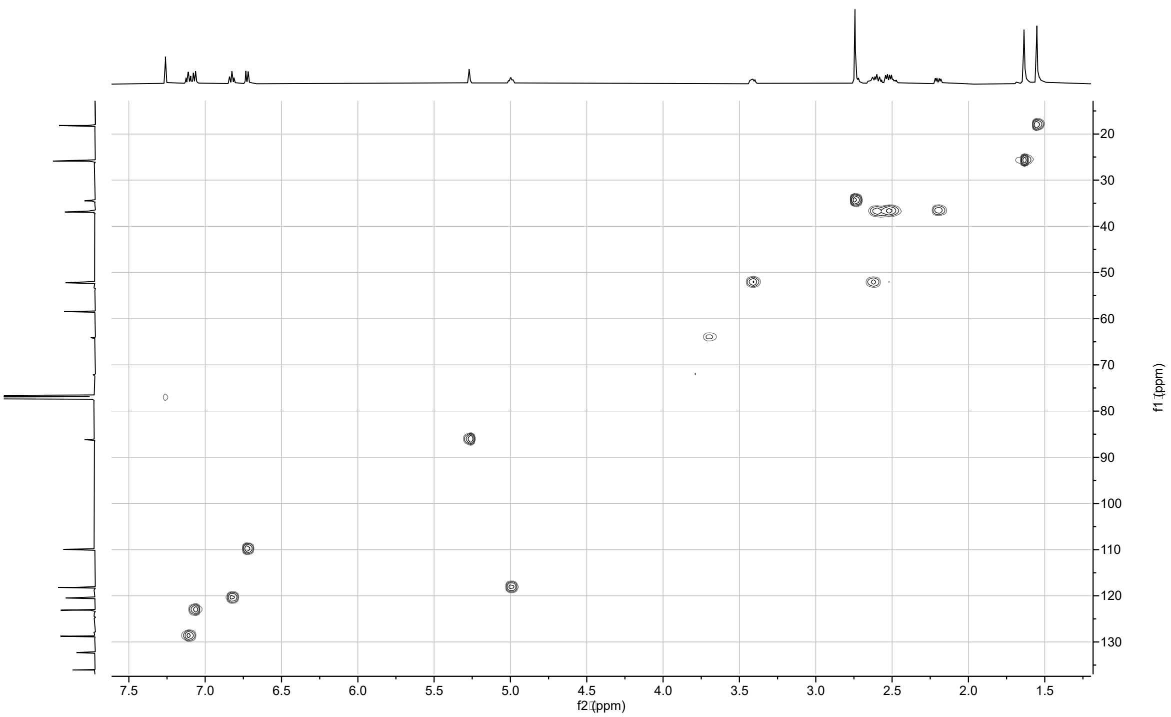

Fig. S89. HSQC spectrum of (–)-deoxypseudophrynaminol (**18**) in $CDCl_3$ (500 MHz).

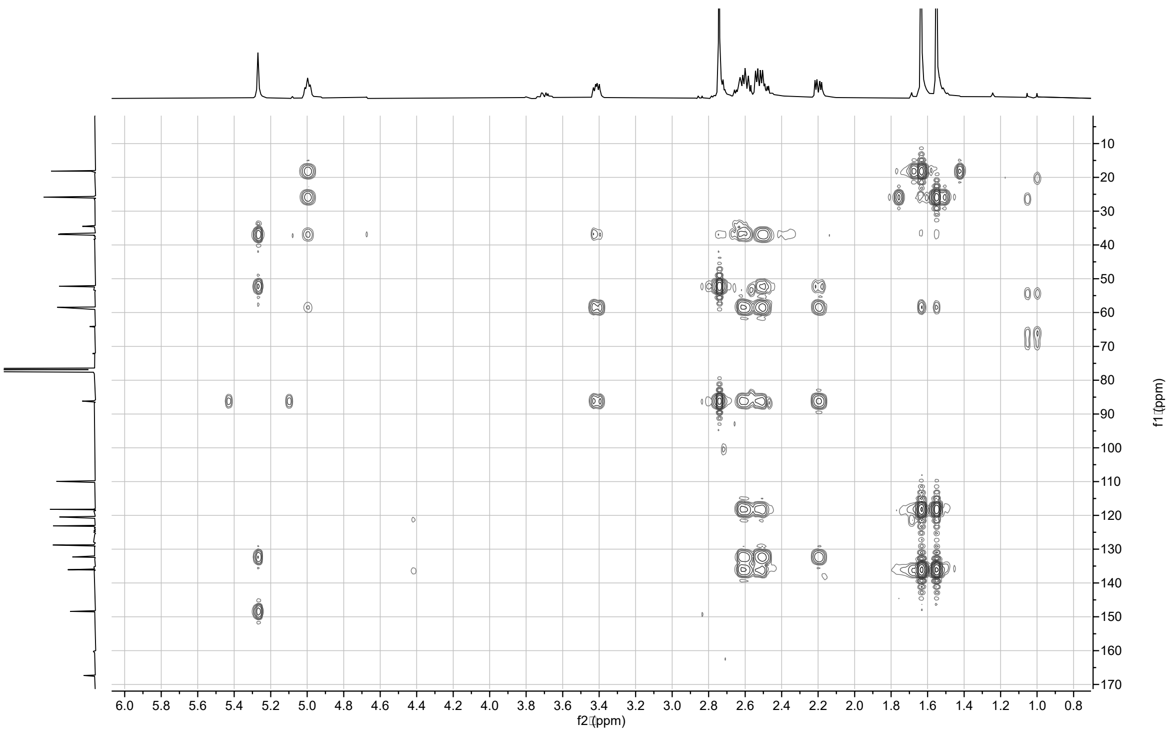

Fig. S90. HMBC spectrum of (–)-deoxypseudophrynaminol (**18**) in $CDCl_3$ (500 MHz).

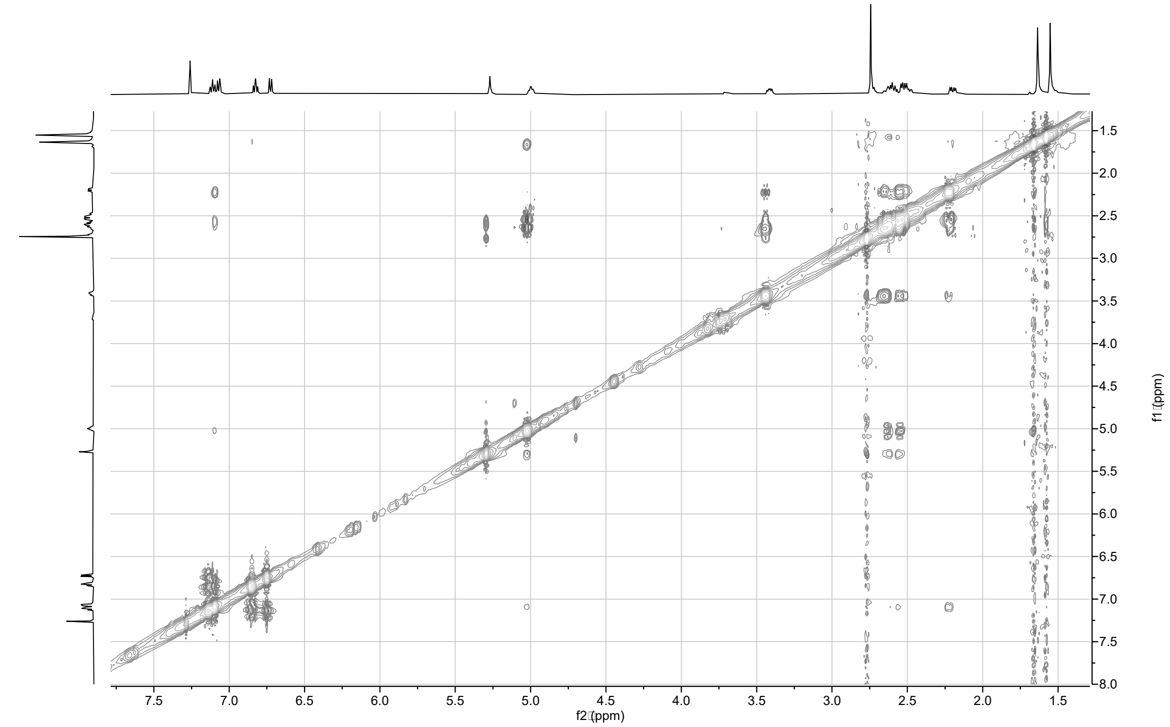

Fig. S91. NOESY spectrum of (–)-deoxypseudophrynaminol (**18**) in $CDCl_3$ (500 MHz).

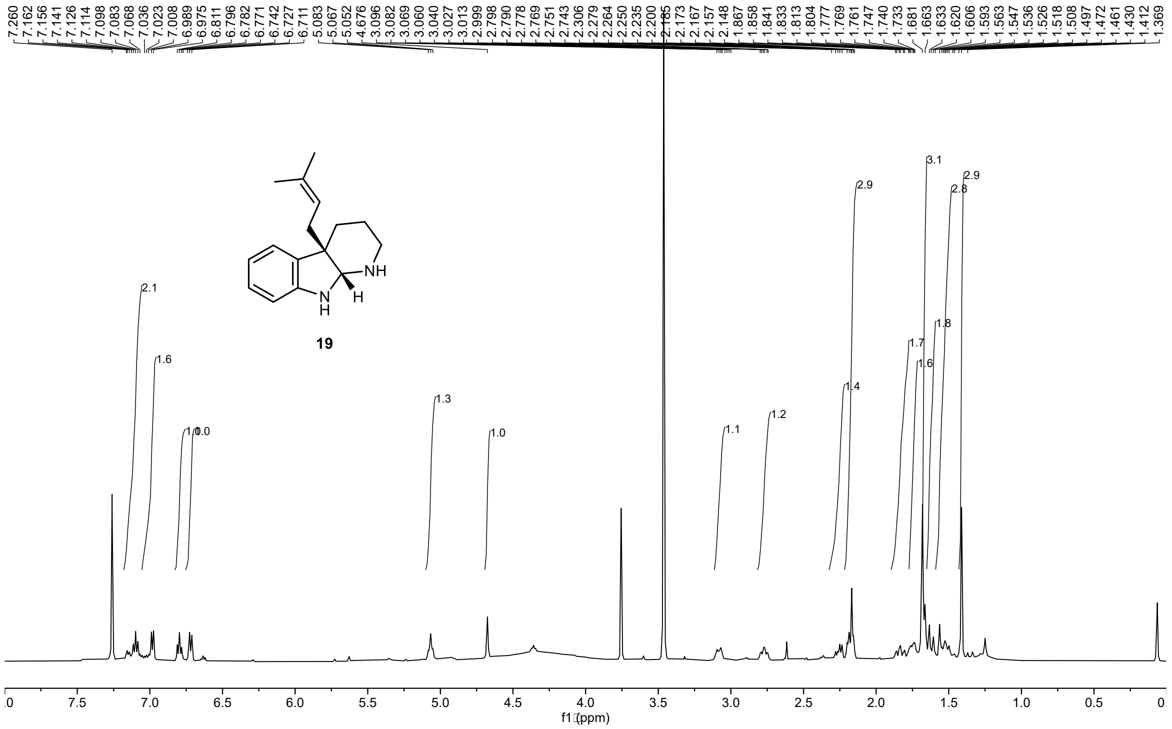

Fig. S92. ^{1}H NMR spectrum of **19** in $CDCl_3$ (500 MHz).

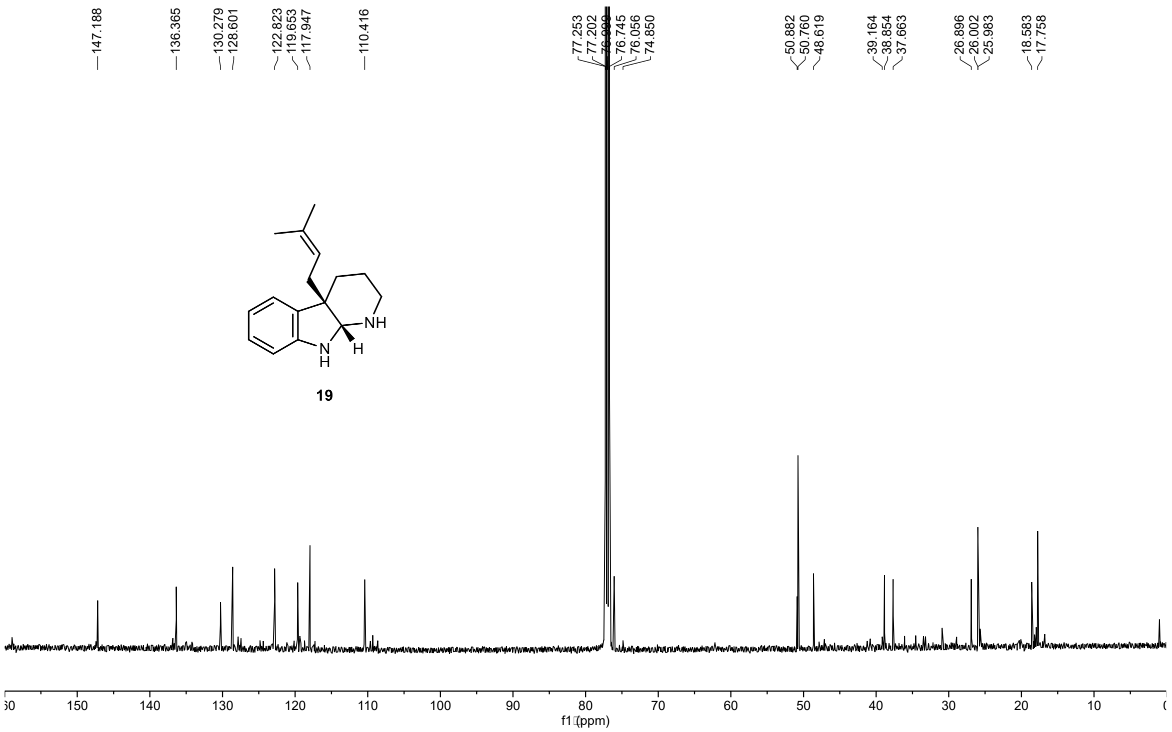

Fig. S93. ^{13}C NMR spectrum of **19** in $CDCl_3$ (500 MHz).

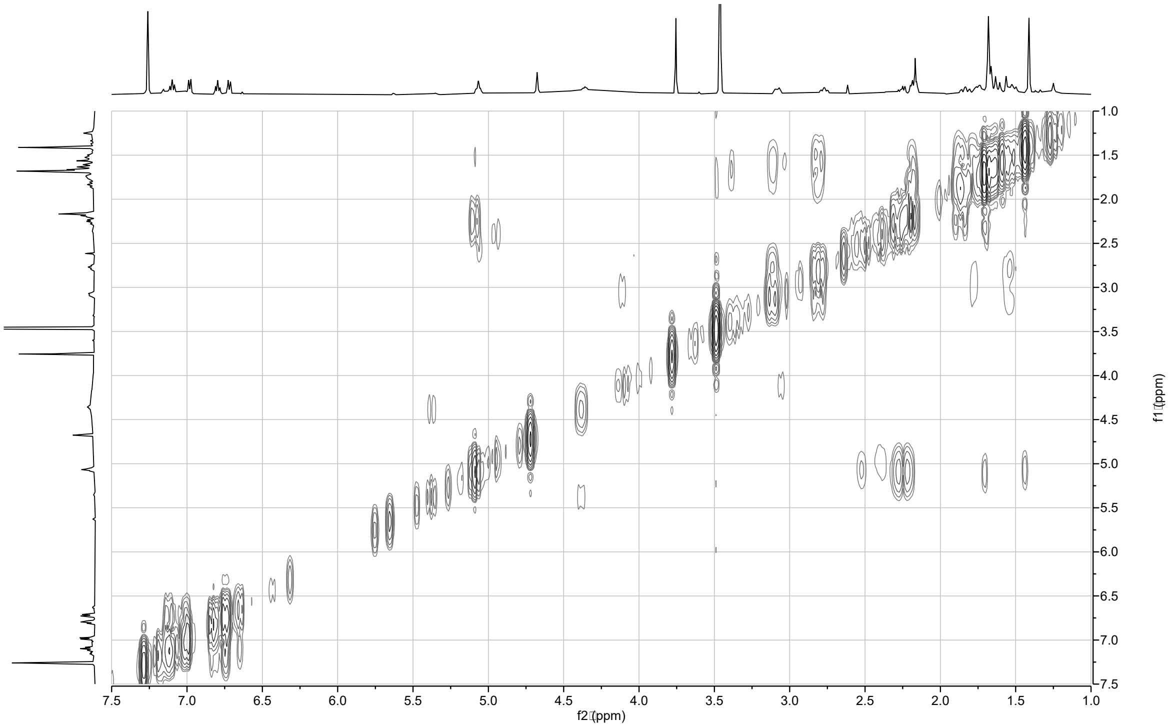

Fig. S94. ^{1}H-^{1}H COSY spectrum of **19** in $CDCl_3$ (500 MHz).

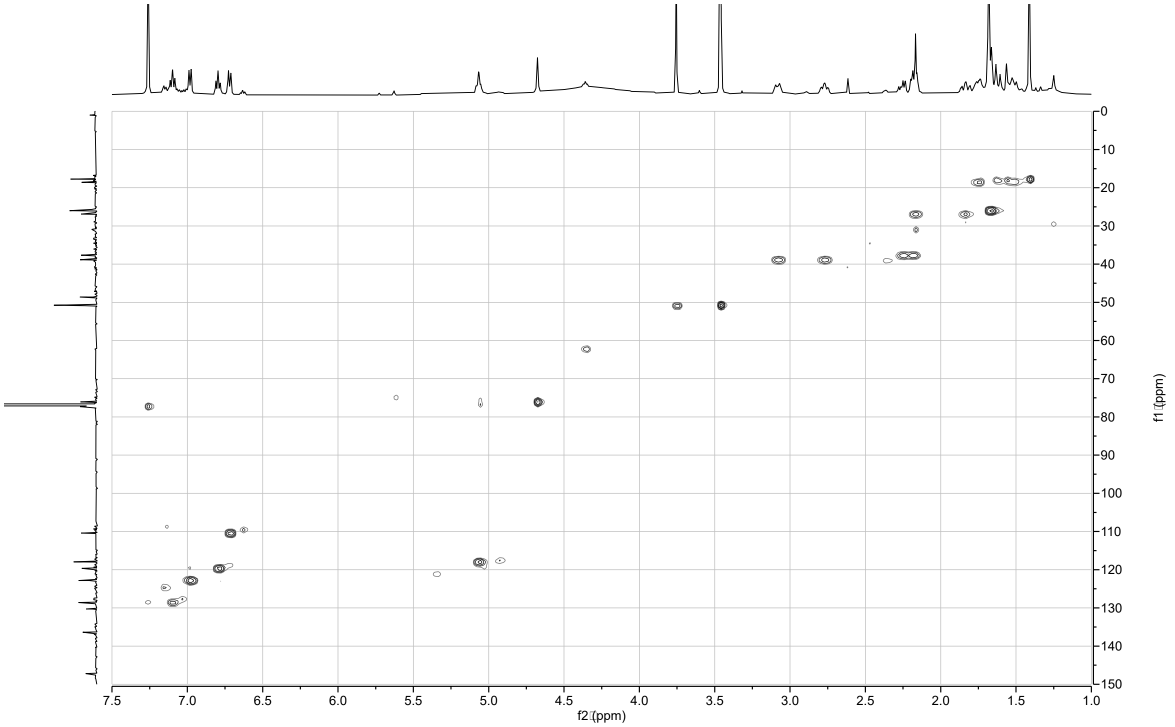

Fig. S95. HSQC spectrum of **19** in $CDCl_3$ (500 MHz).

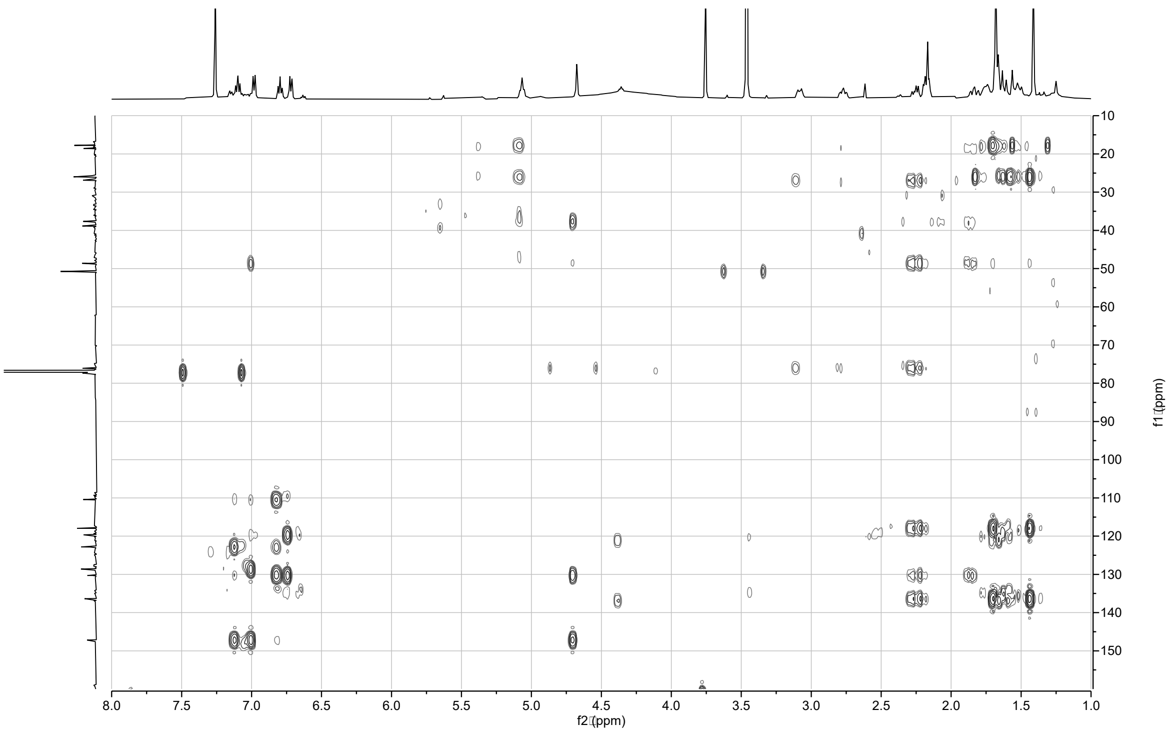

Fig. S96. HMBC spectrum of **19** in $CDCl_3$ (500 MHz).

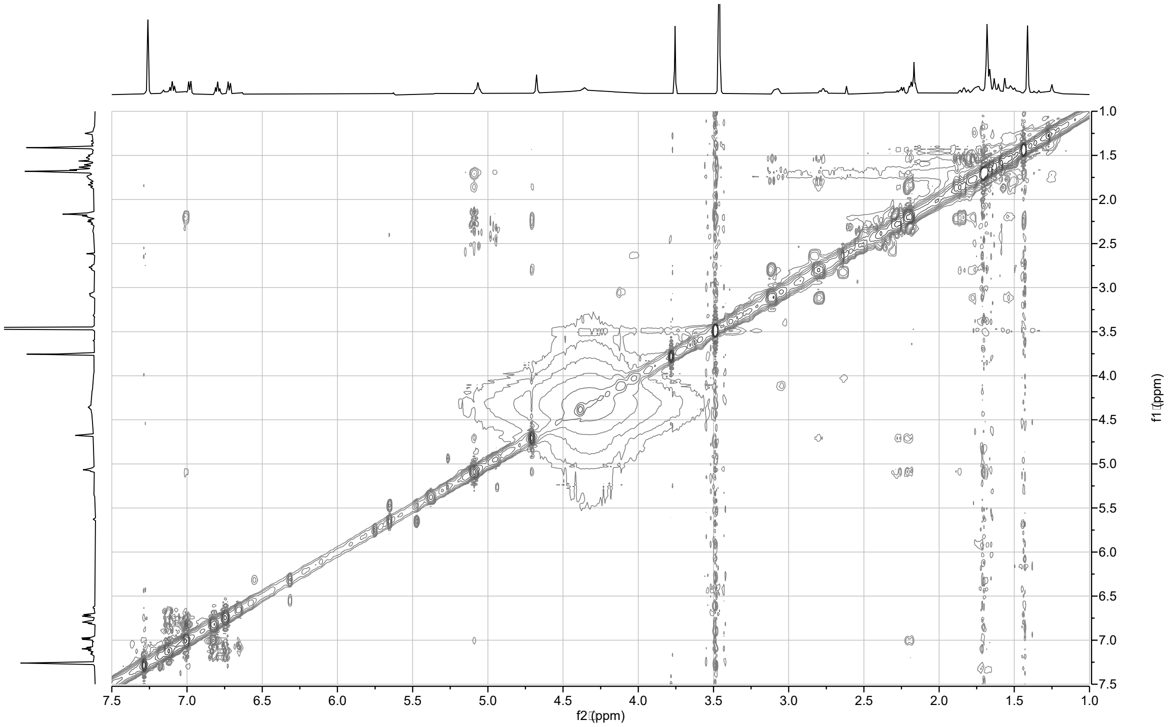

Fig. S97. NOESY spectrum of **19** in $CDCl_3$ (500 MHz).

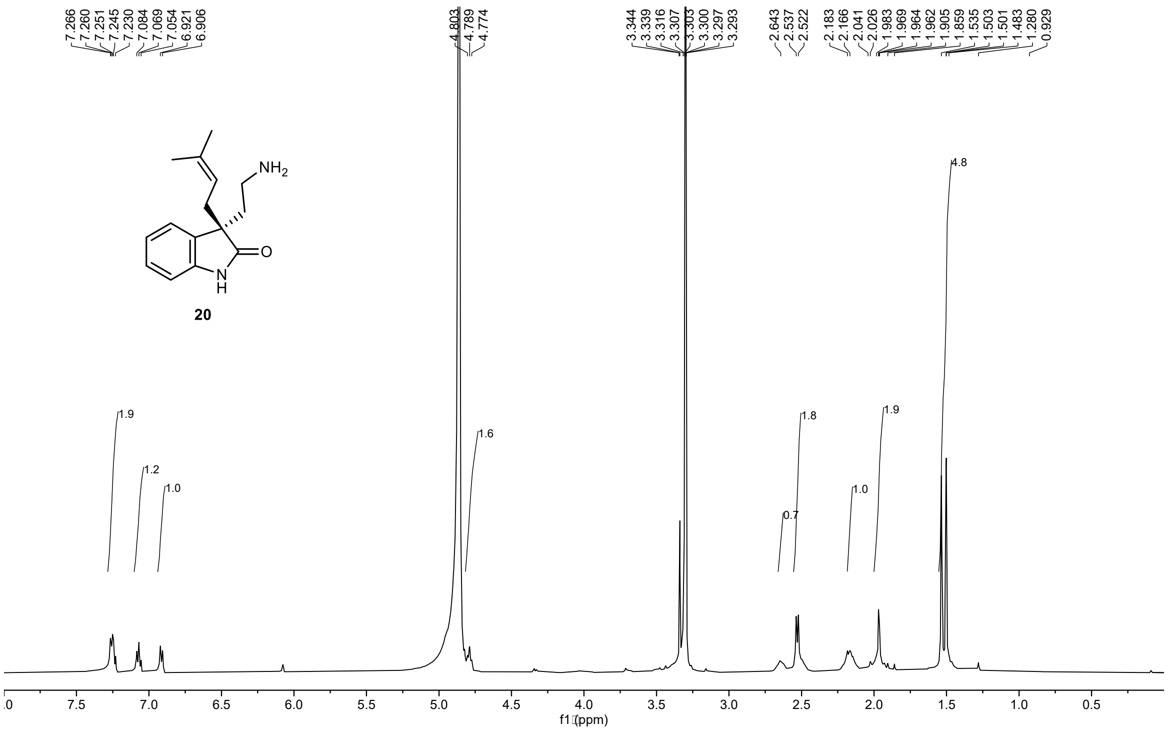

Fig. S98. ^{1}H NMR spectrum of **20** in CD_3OD (500 MHz).

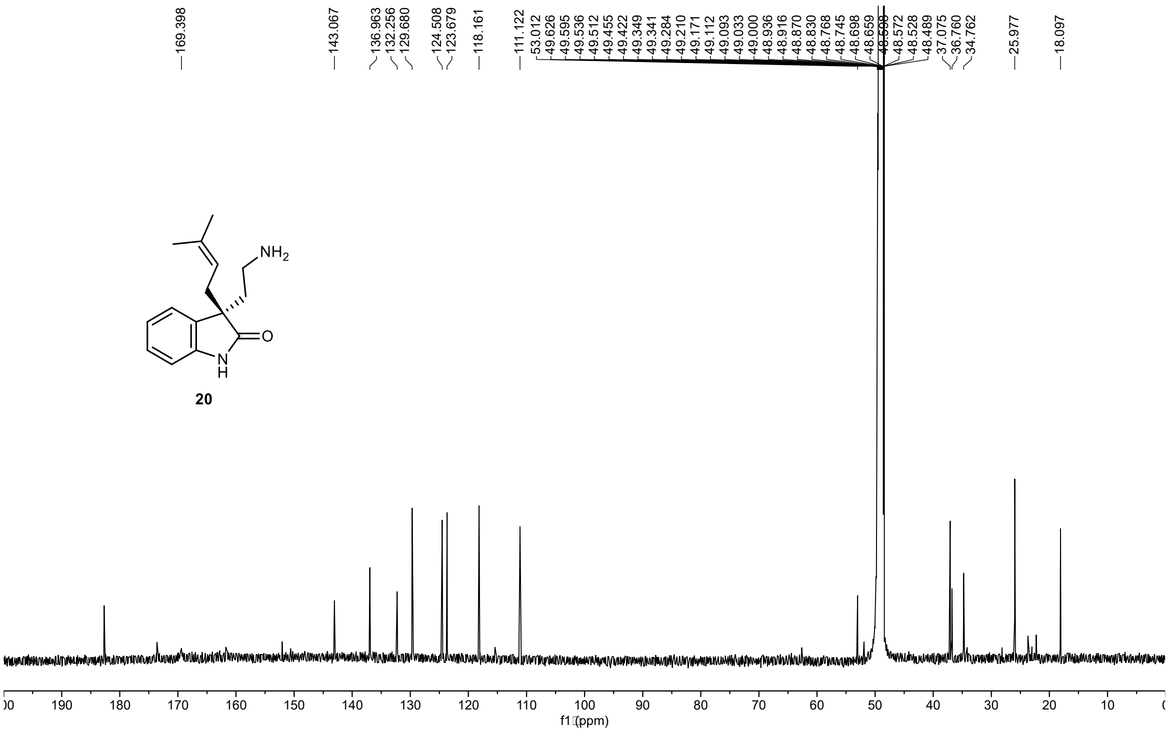

Fig. S99. ^{13}C NMR spectrum of **20** in CD_3OD (125 MHz).

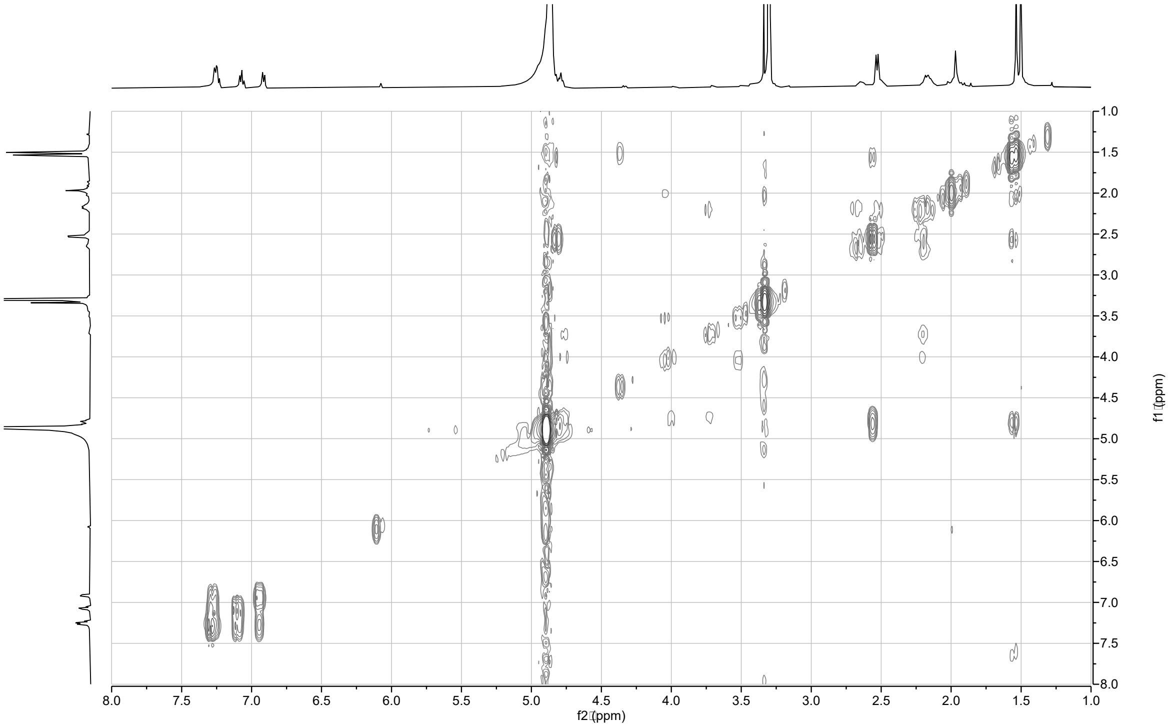

Fig. S100. ^{1}H-^{1}H COSY spectrum of **20** in CD_3OD (500 MHz).

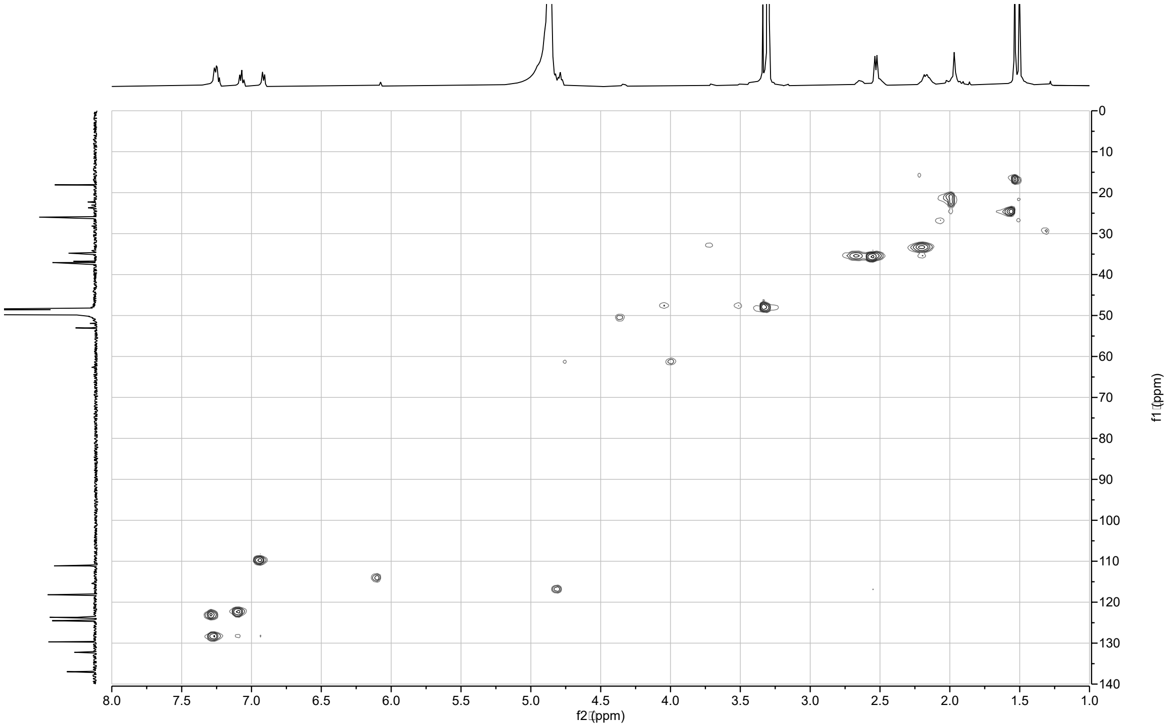

Fig. S101. HSQC spectrum of **20** in CD_3OD (500 MHz).

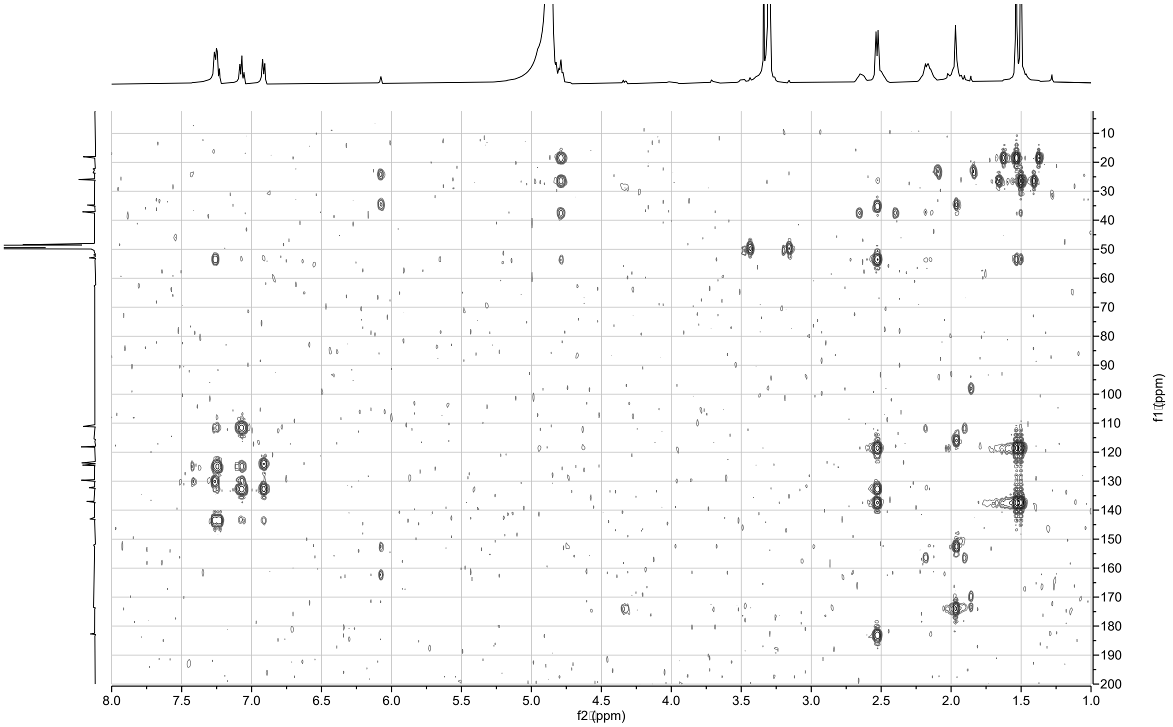

Fig. S102. HMBC spectrum of **20** in CD_3OD (500 MHz).

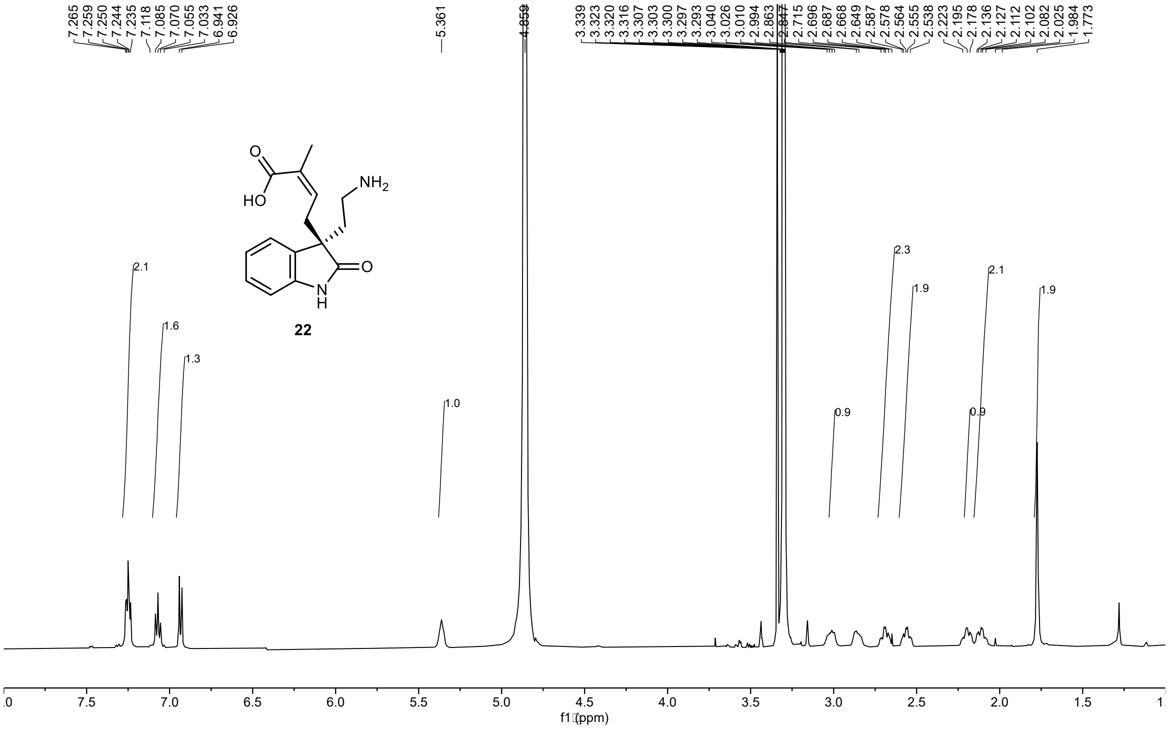

Fig. S103. ^1H NMR spectrum of **22** in CD_3OD (500 MHz).

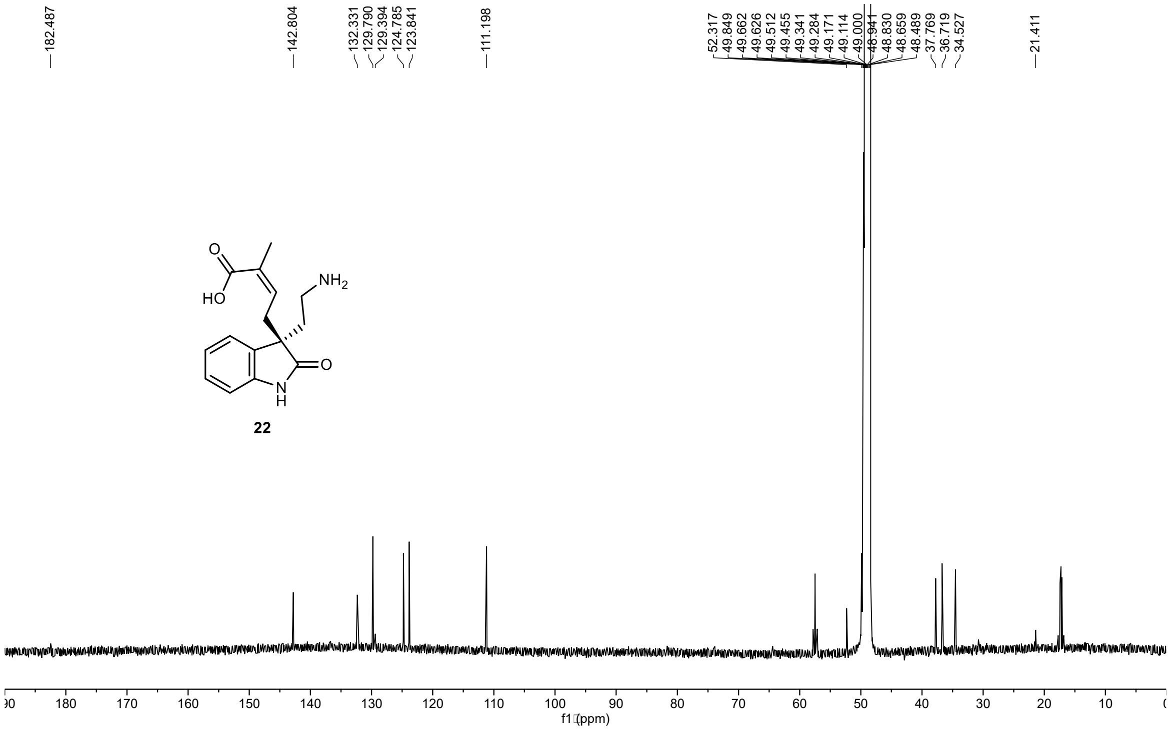

Fig. S104. ^{13}C NMR spectrum of **22** in CD_3OD (125 MHz).

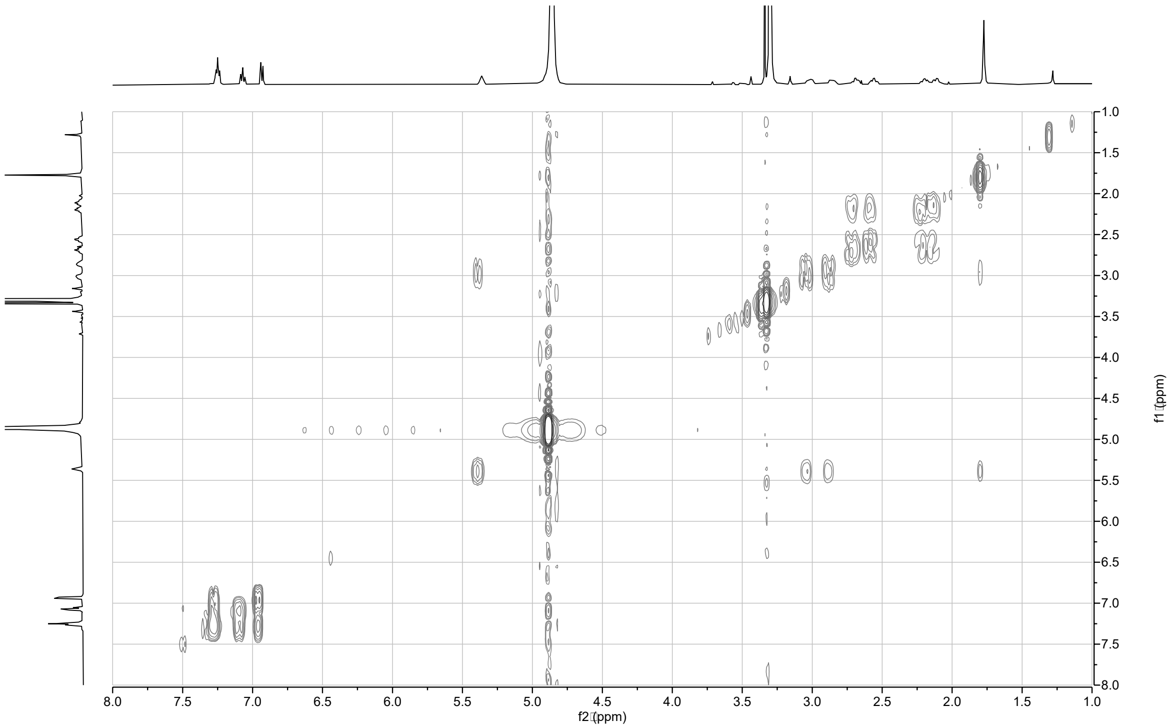

Fig. S105. ^{1}H-^{1}H COSY spectrum of **22** in CD_3OD (500 MHz).

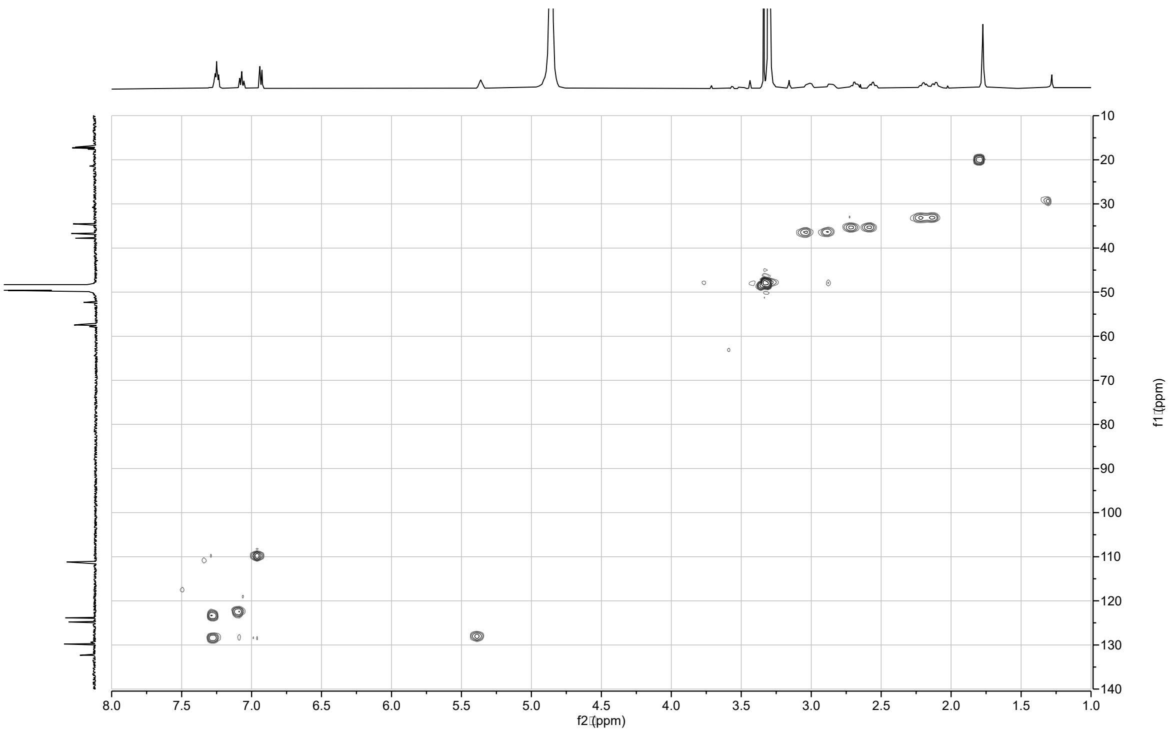

Fig. S106. HSQC spectrum of **22** in CD_3OD (500 MHz).

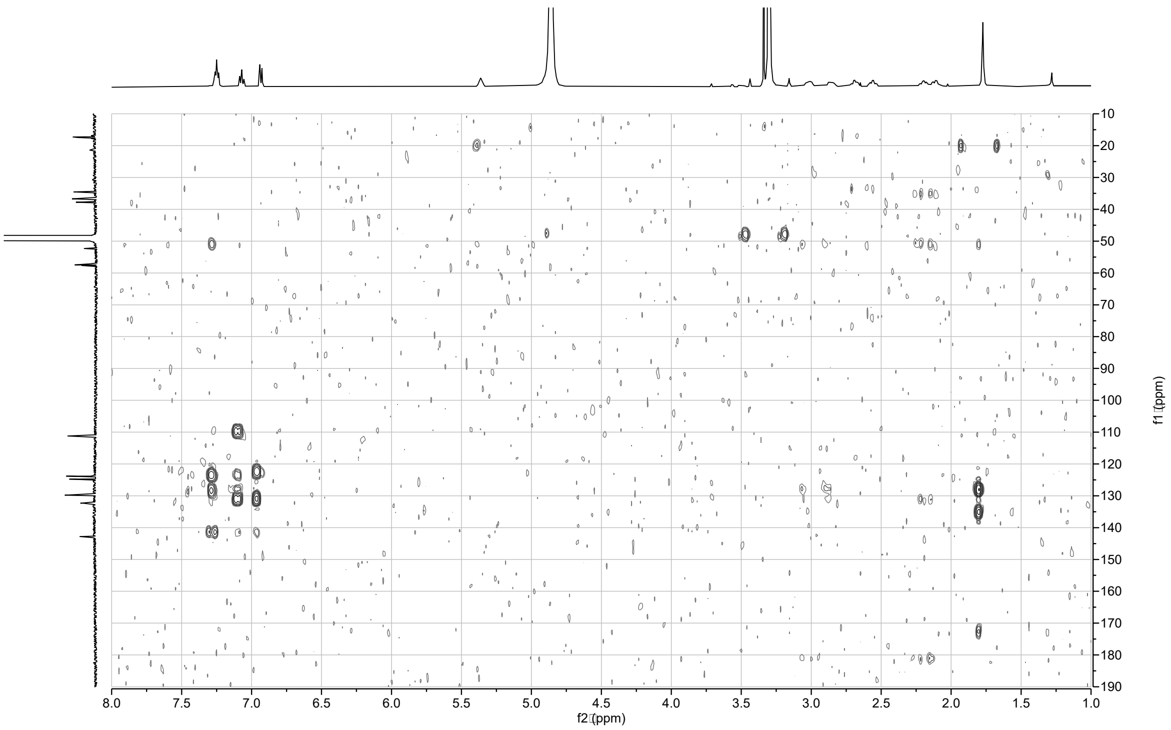

Fig. S107. HMBC spectrum of **22** in CD_3OD (500 MHz).

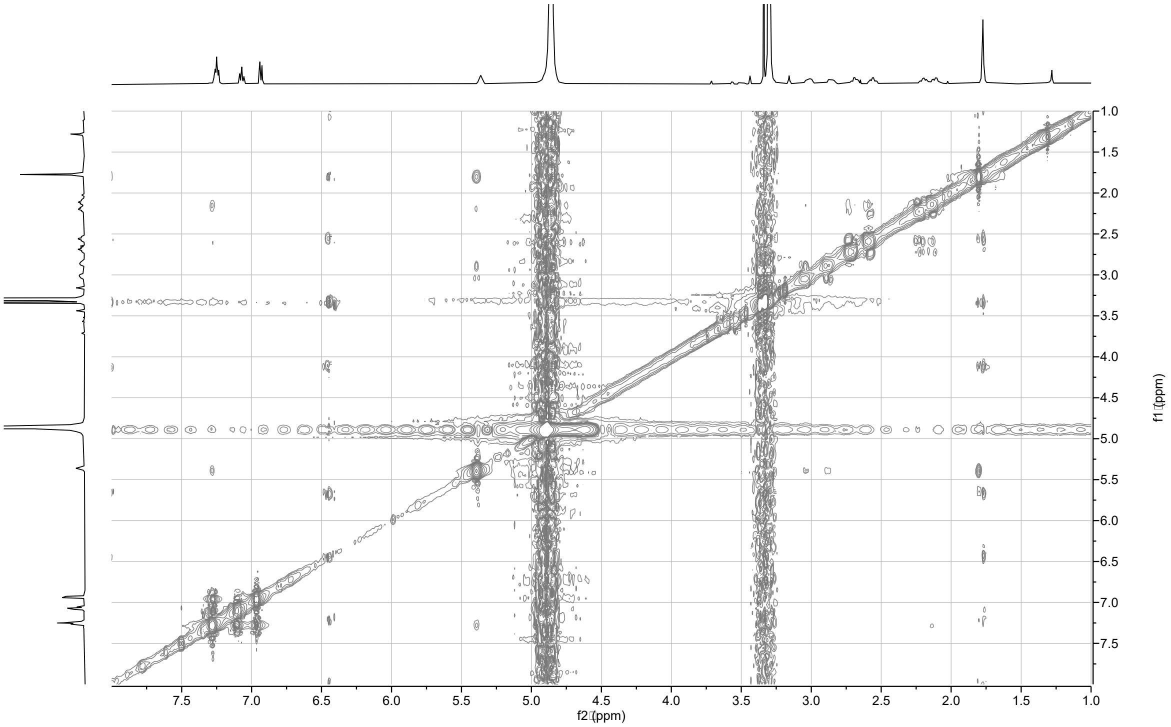

Fig. S108. NOESY spectrum of **22** in CD_3OD (500 MHz).

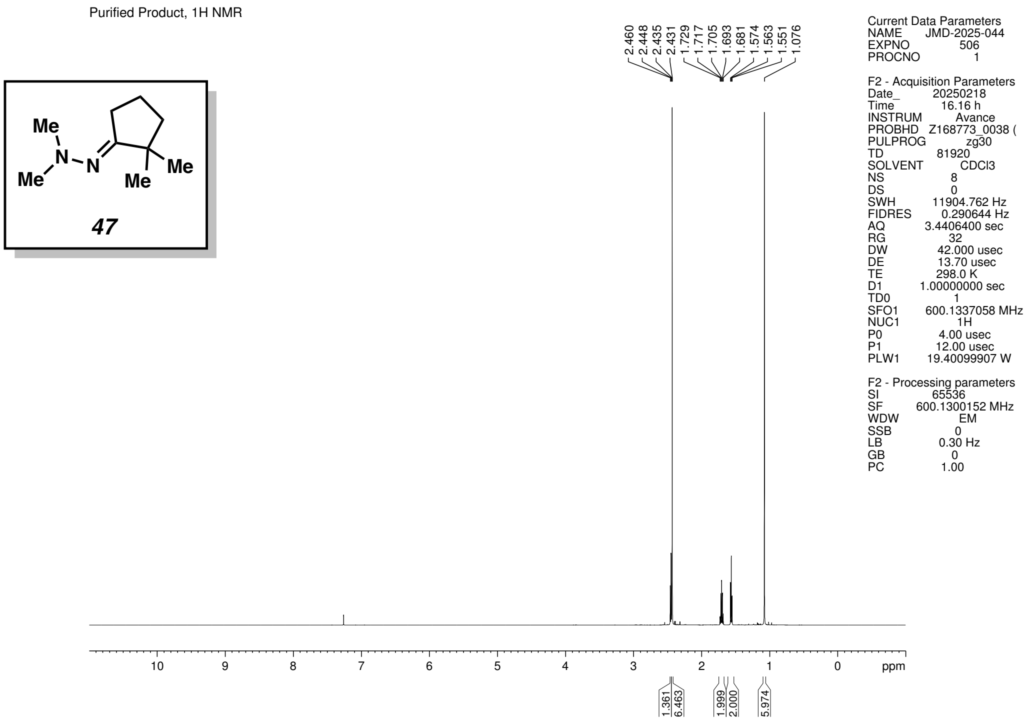

Fig. S109. ^{1}H NMR spectrum of **47** in $CDCl_3$ (600 MHz).

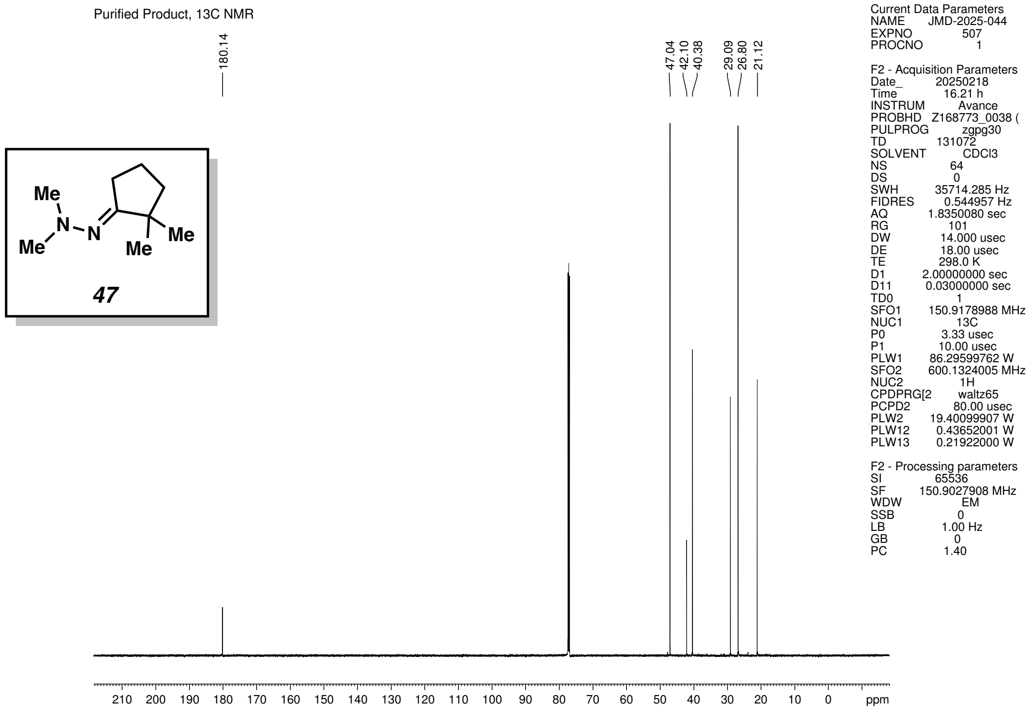

Fig. S110. ^{13}C NMR spectrum of **47** in $CDCl_3$ (151 MHz).

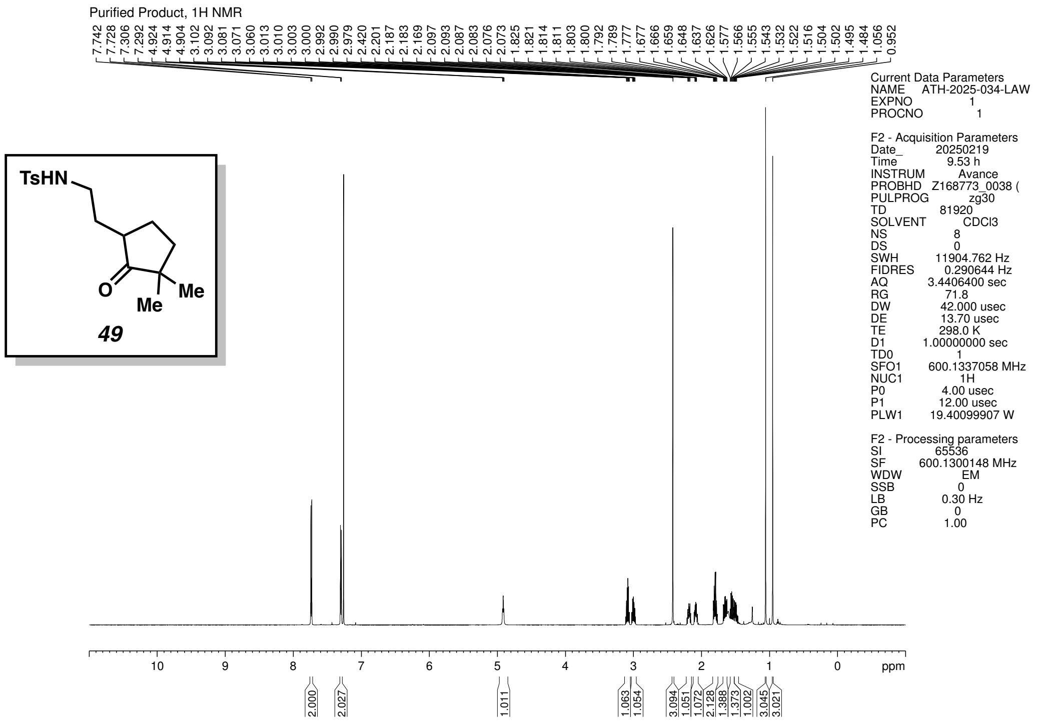

Fig. S111. ^{1}H NMR spectrum of **49** in $CDCl_3$ (600 MHz).

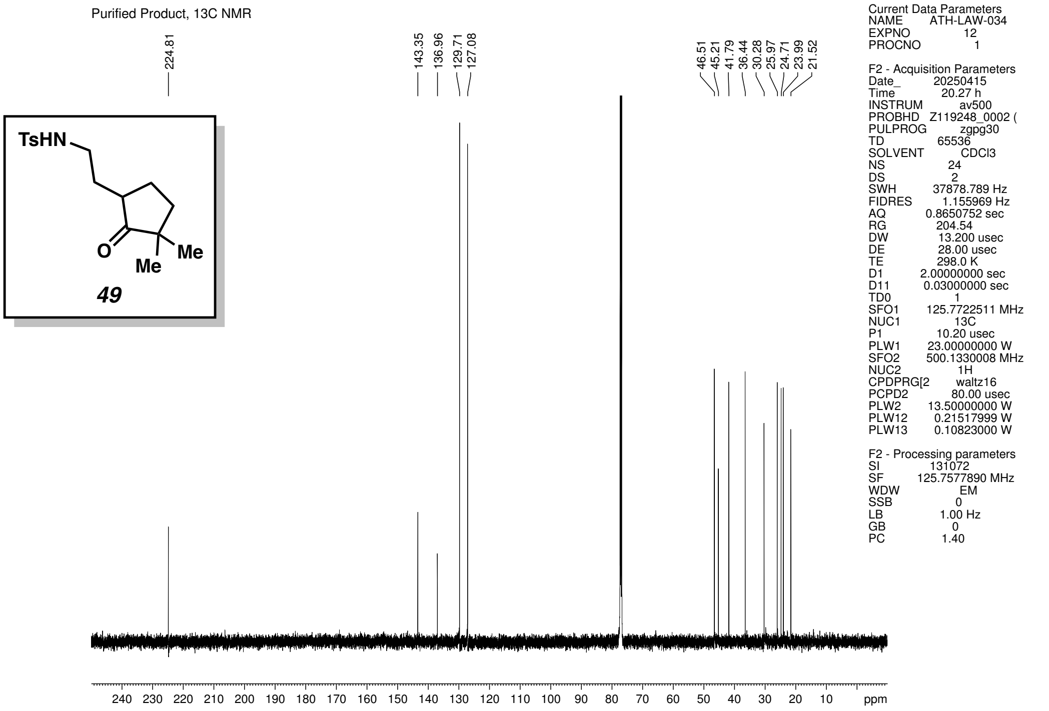

Fig. S112. ^{13}C NMR spectrum of **49** in $CDCl_3$ (126 MHz).

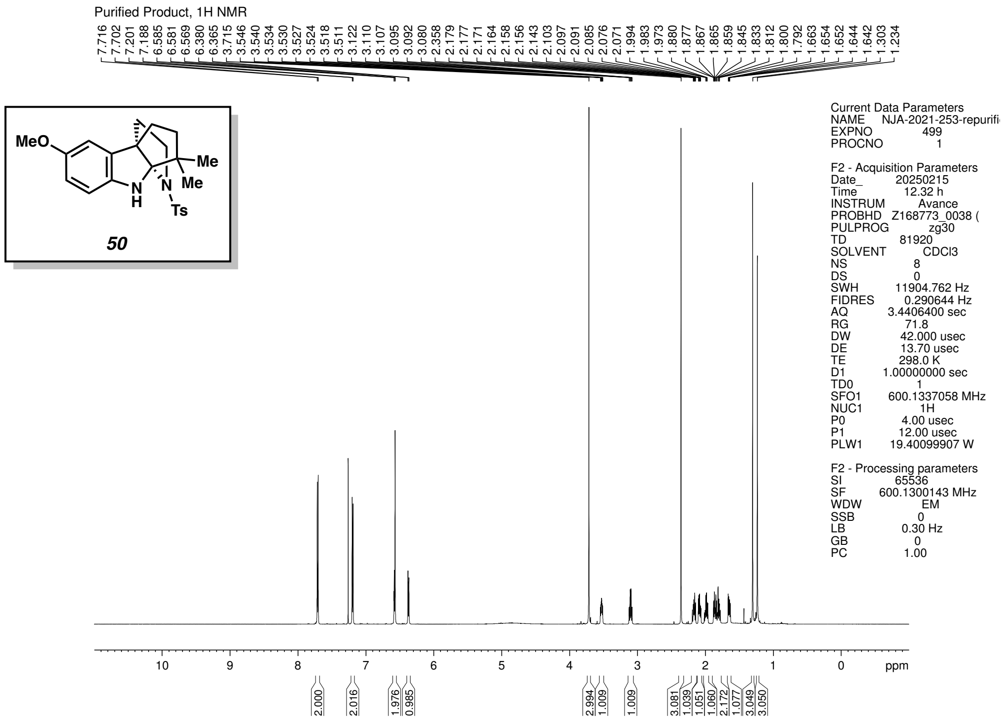

Fig. S113. ^{1}H NMR spectrum of **50** in $CDCl_3$ (600 MHz).

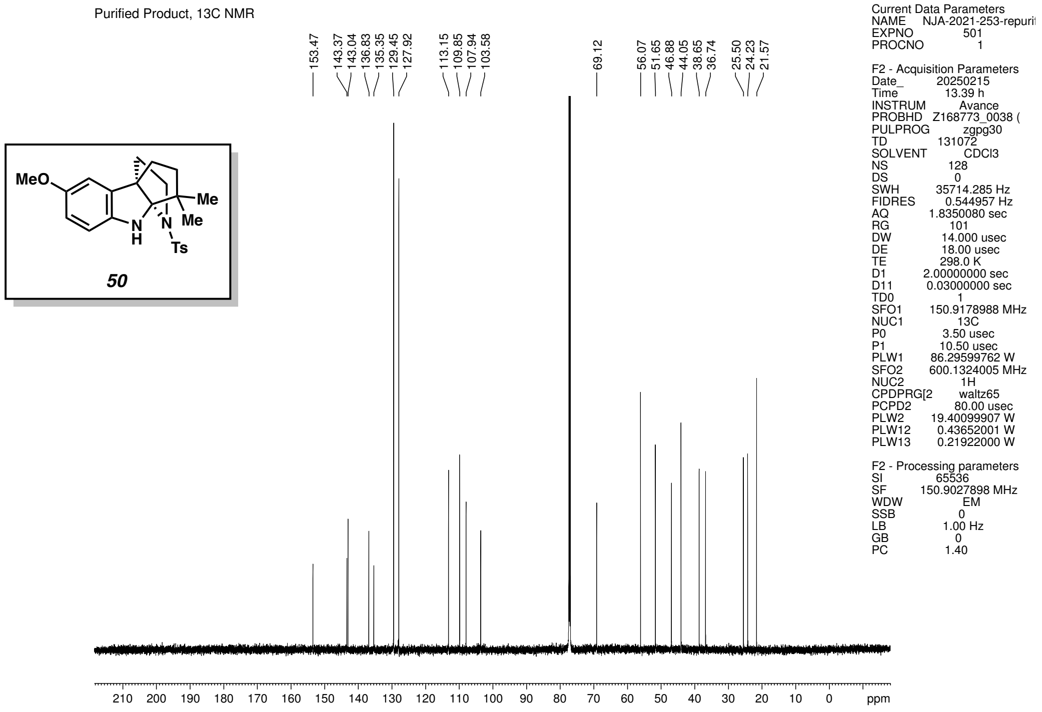

Fig. S114. ^{13}C NMR spectrum of **50** in $CDCl_3$ (151 MHz).

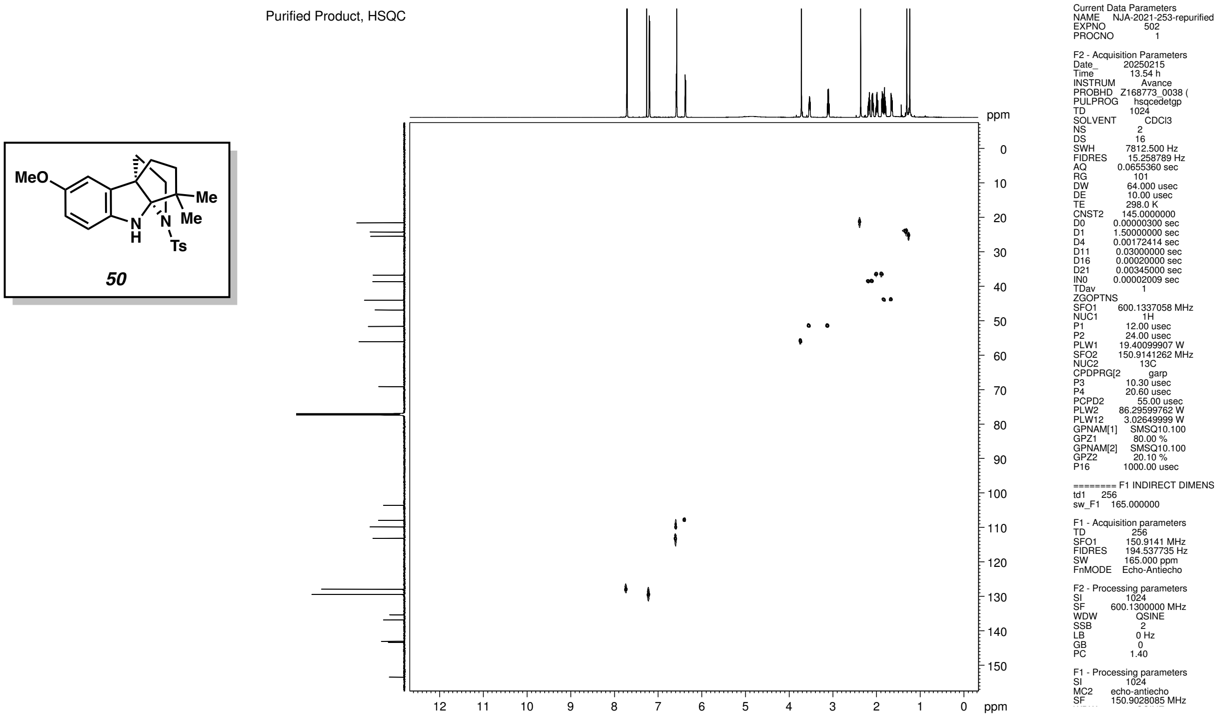

Fig. S115. HSQC spectrum of **50** in $CDCl_3$ (600 MHz).

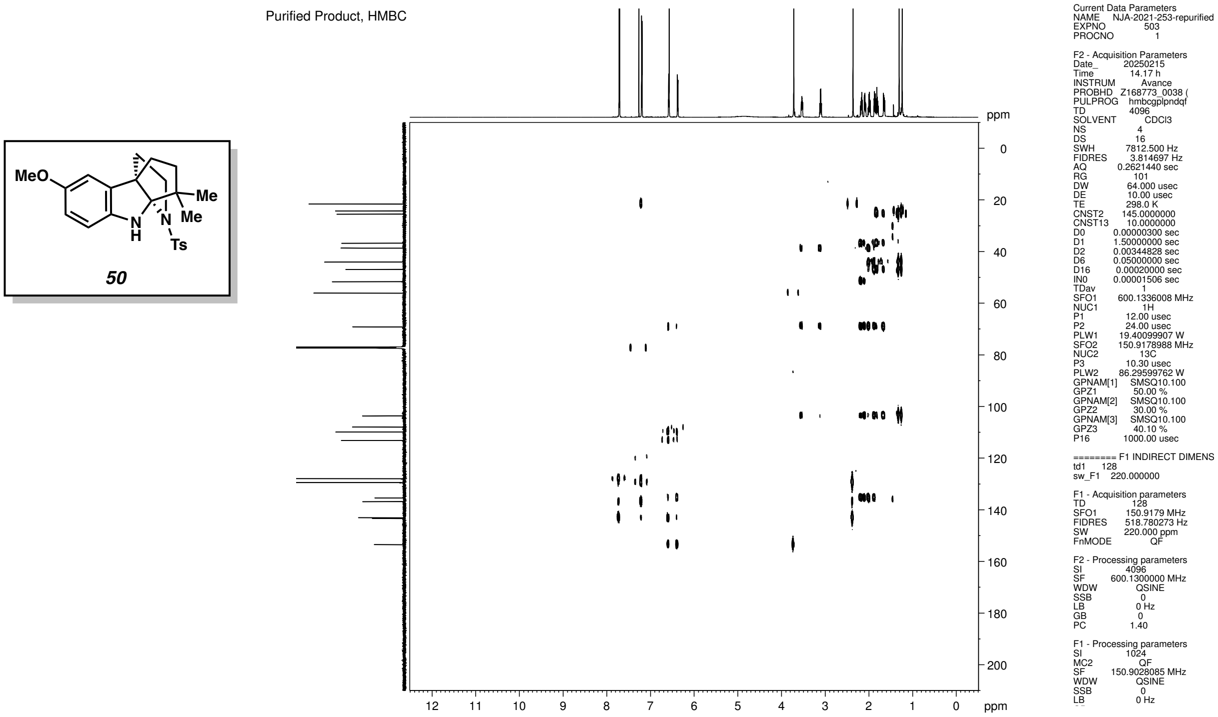

Fig. S116. HMBC spectrum of **50** in $CDCl_3$ (600 MHz).

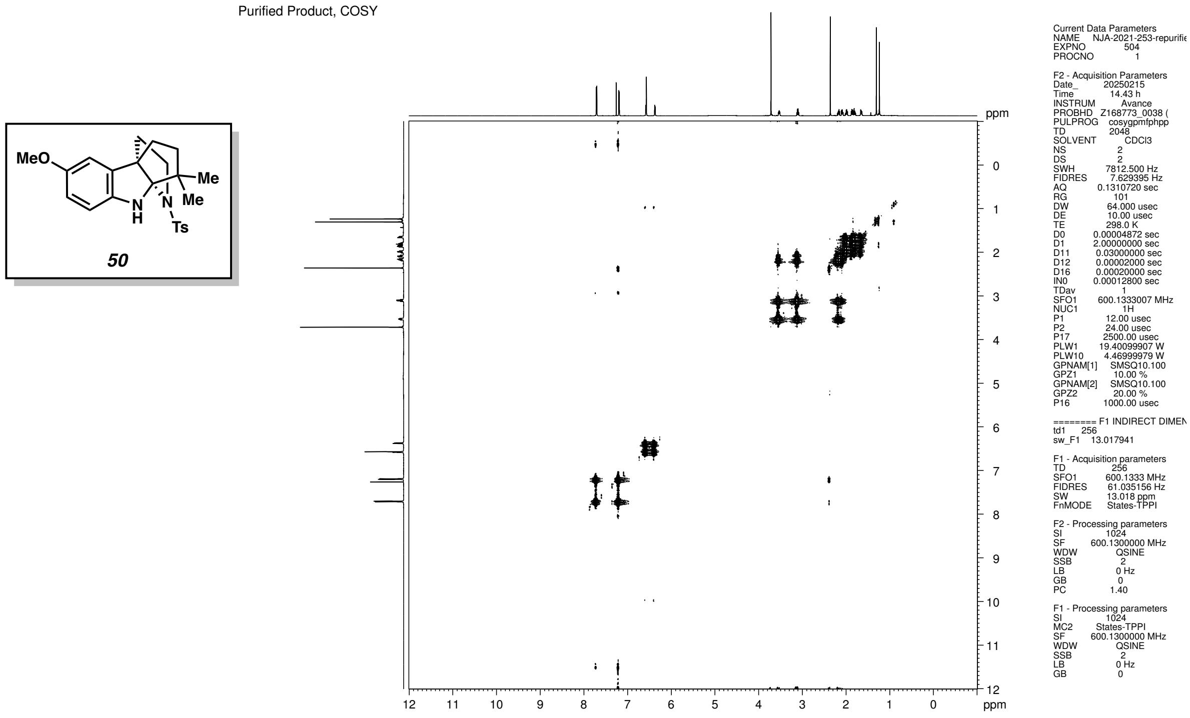

Fig. S117. ${}^{1}H$-${}^{1}H$ COSY spectrum of **50** in $CDCl_3$ (600 MHz).

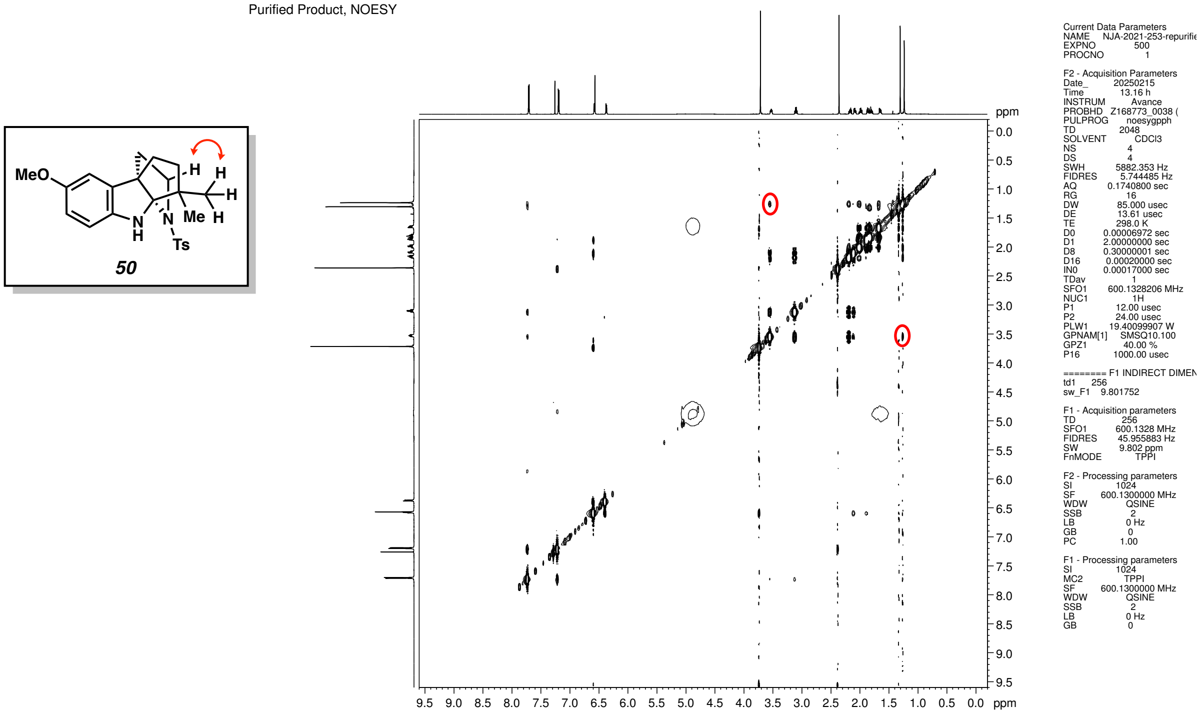

Fig. S118. NOESY spectrum of **50** in $CDCl_3$ (600 MHz).

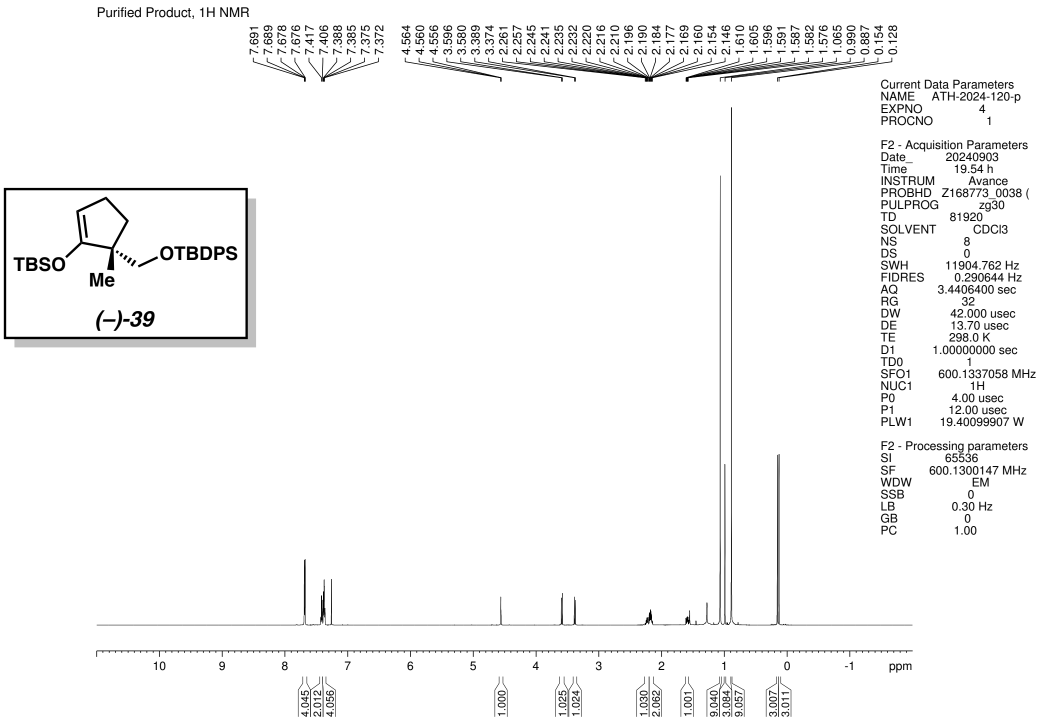

Fig. S119. ^{1}H NMR spectrum of (–)-**39** in $CDCl_3$ (600 MHz).

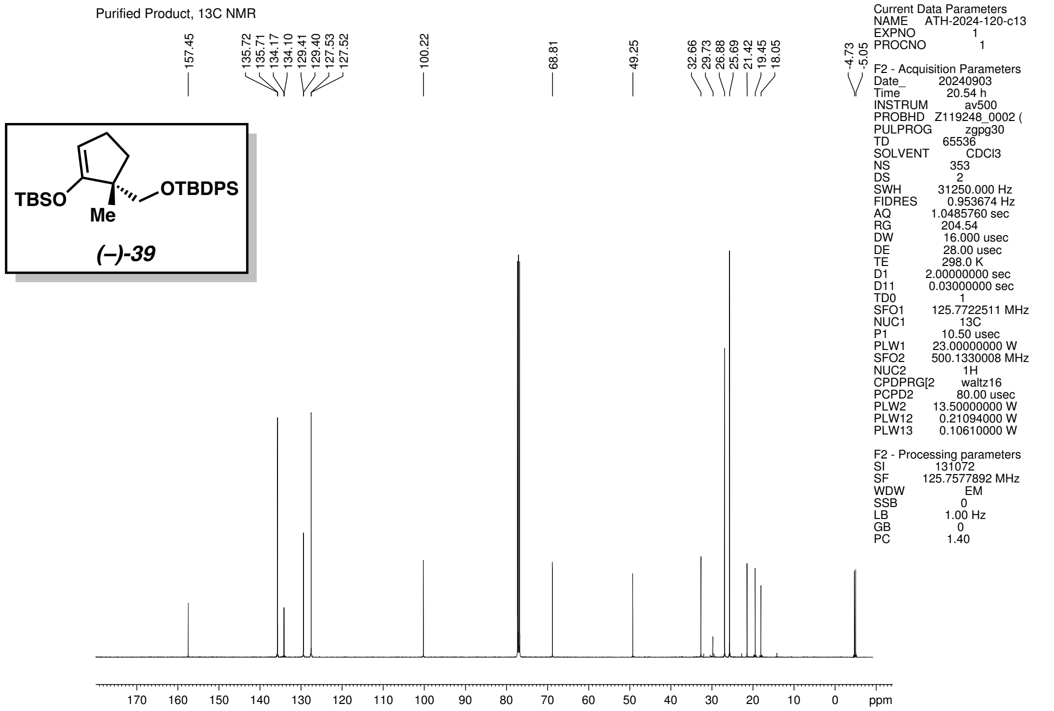

Fig. S120. ^{13}C NMR spectrum of (–)-**39** in $CDCl_3$ (126 MHz).

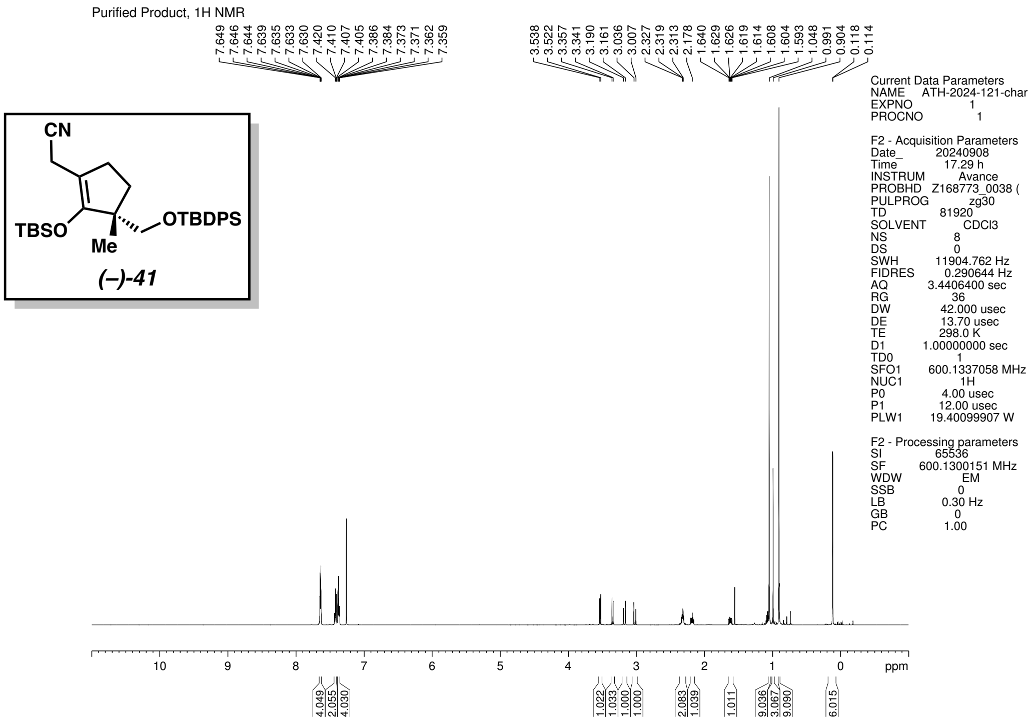

Fig. S121. ^{1}H NMR spectrum of (–)-**41** in $CDCl_3$ (600 MHz).

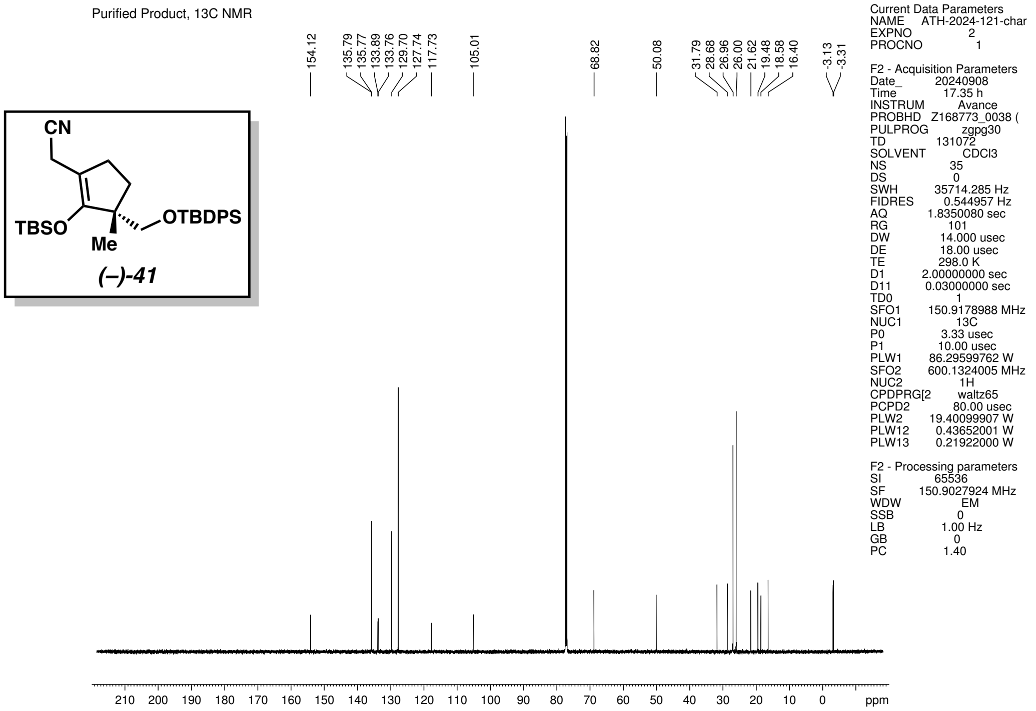

Fig. S122. ^{13}C NMR spectrum of (–)-**41** in $CDCl_3$ (151 MHz).

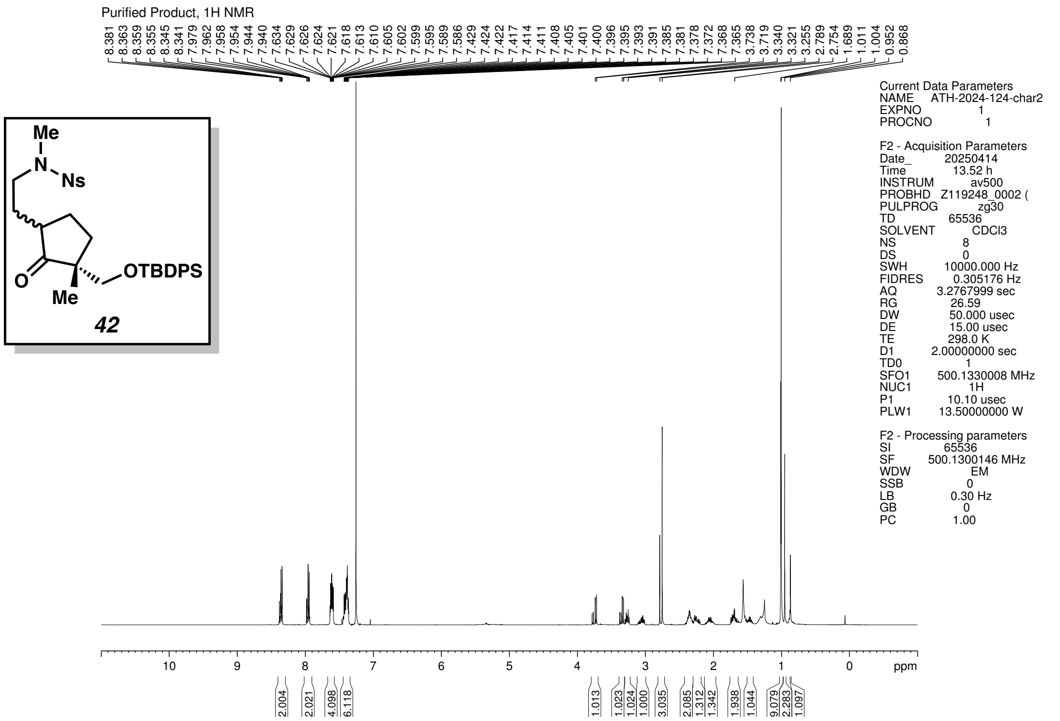

Fig. S123. ^{1}H NMR spectrum of **42** in $CDCl_3$ (500 MHz).

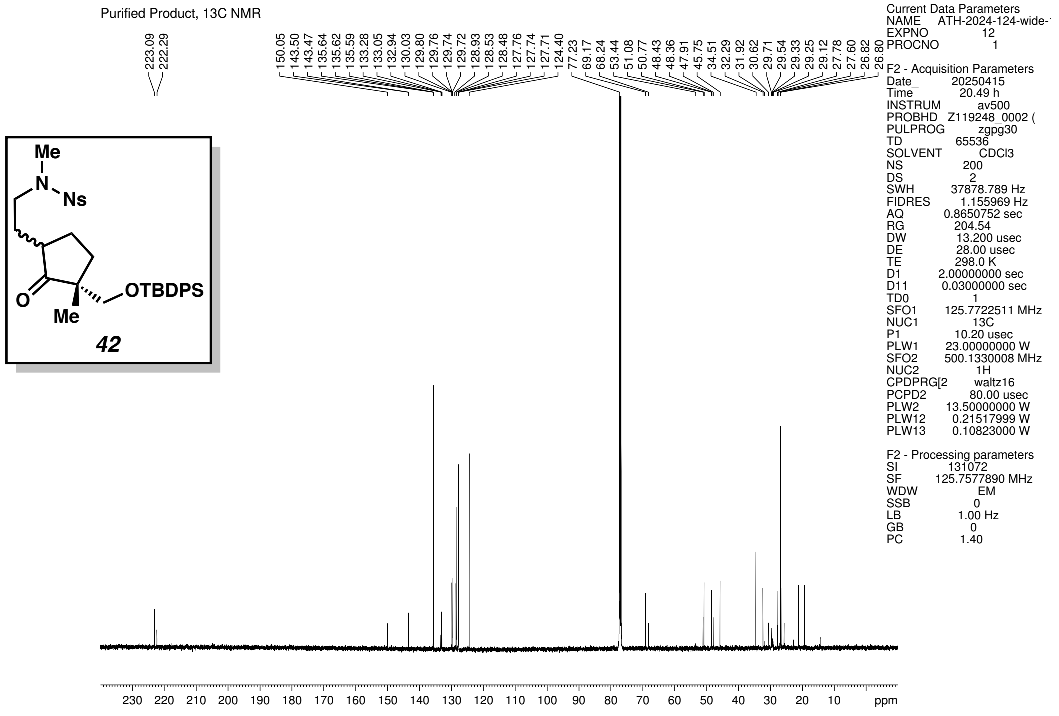

Fig. S124. ^{13}C NMR spectrum of **42** in $CDCl_3$ (126 MHz).

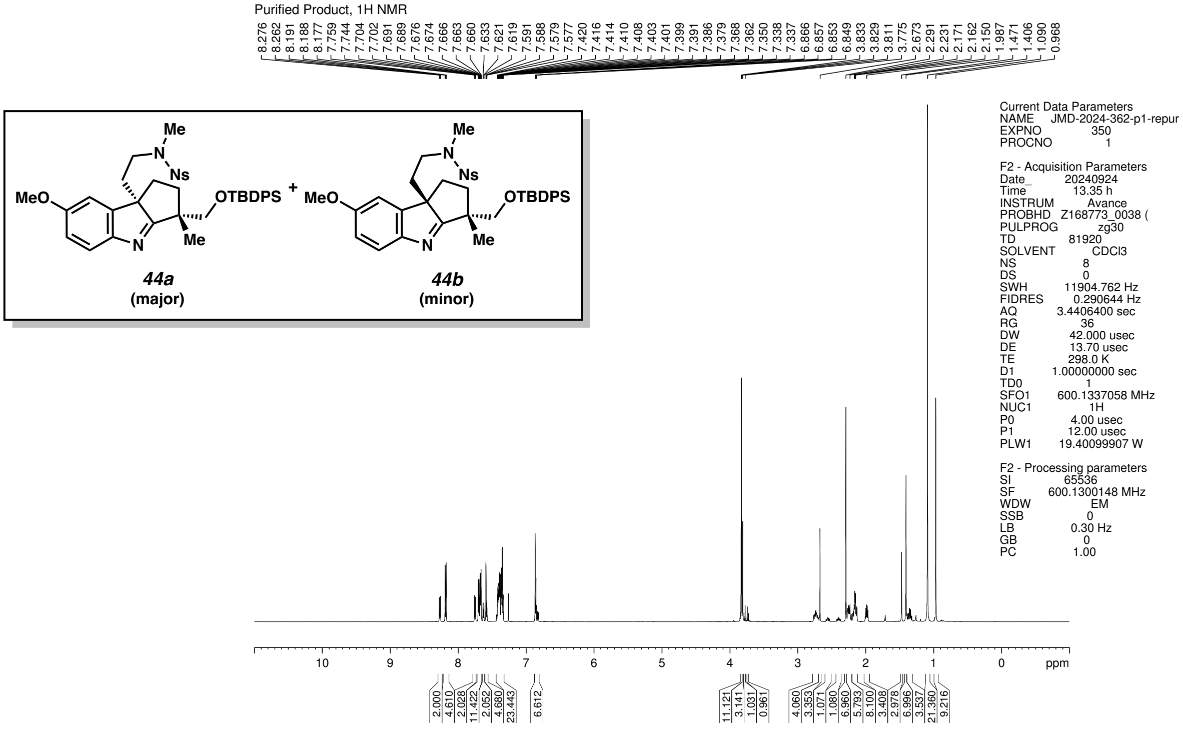

Fig. S125. ^{1}H NMR spectrum of **44a** and **44b** in $CDCl_3$ (600 MHz).

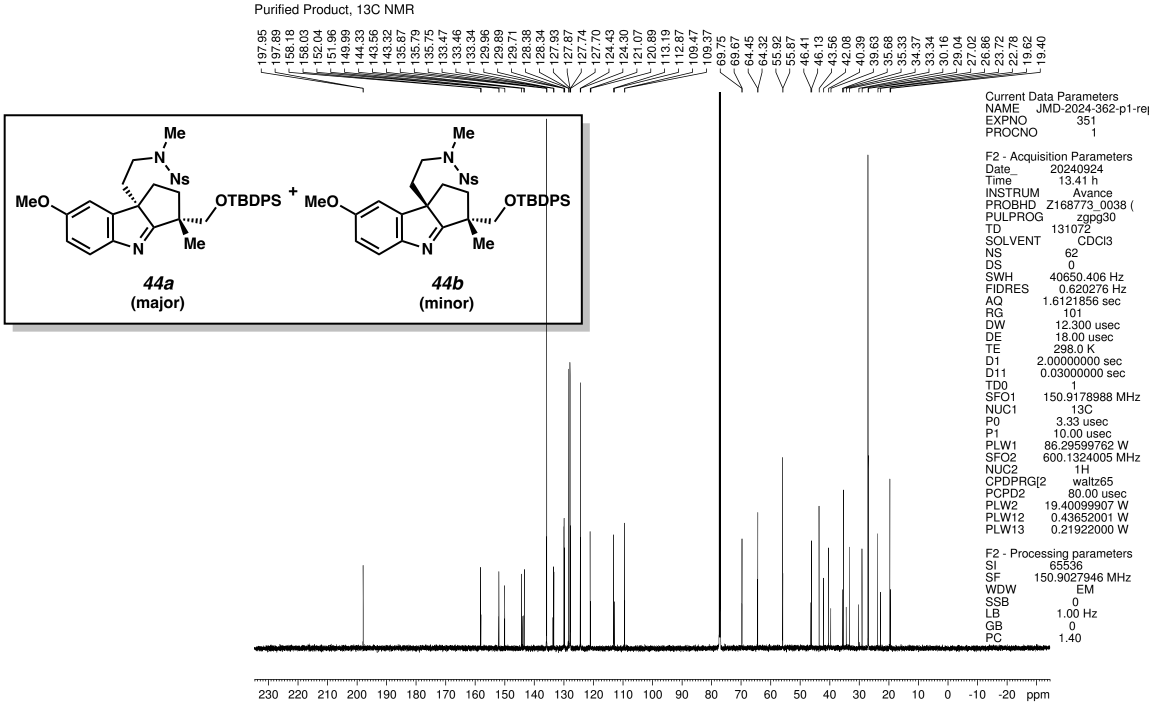

Fig. S126. ^{13}C NMR spectrum of **44a** and **44b** in $CDCl_3$ (151 MHz).

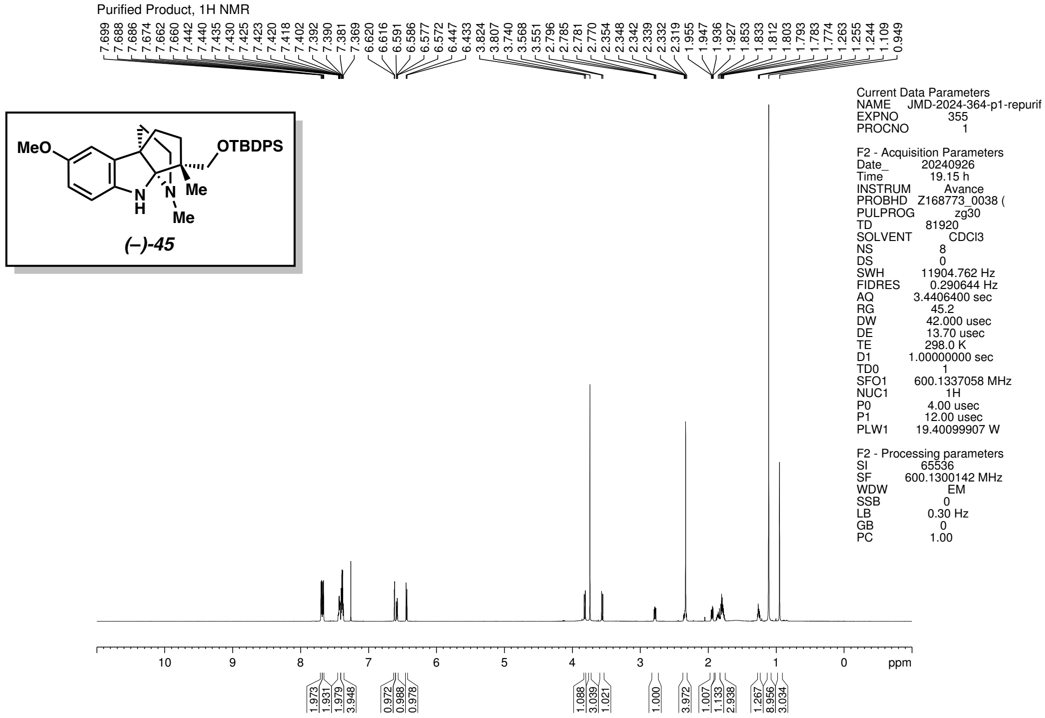

Fig. S127. ^{1}H NMR spectrum of (–)-**45** in $CDCl_3$ (600 MHz).

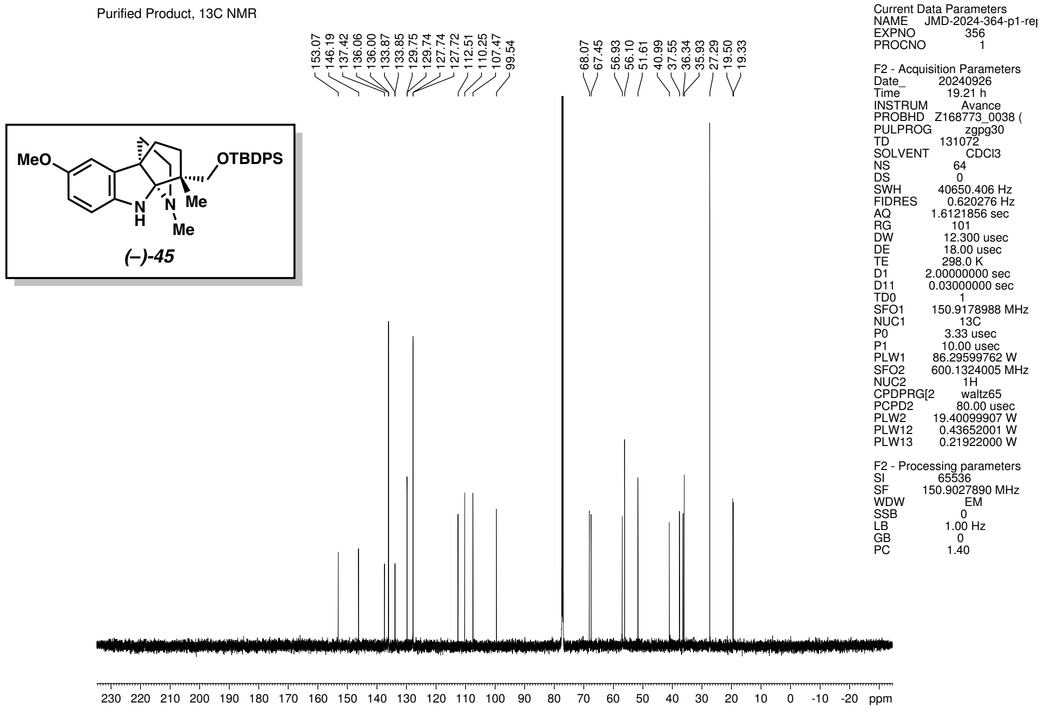

Fig. S128. ^{13}C NMR spectrum of (–)-**45** in $CDCl_3$ (151 MHz).

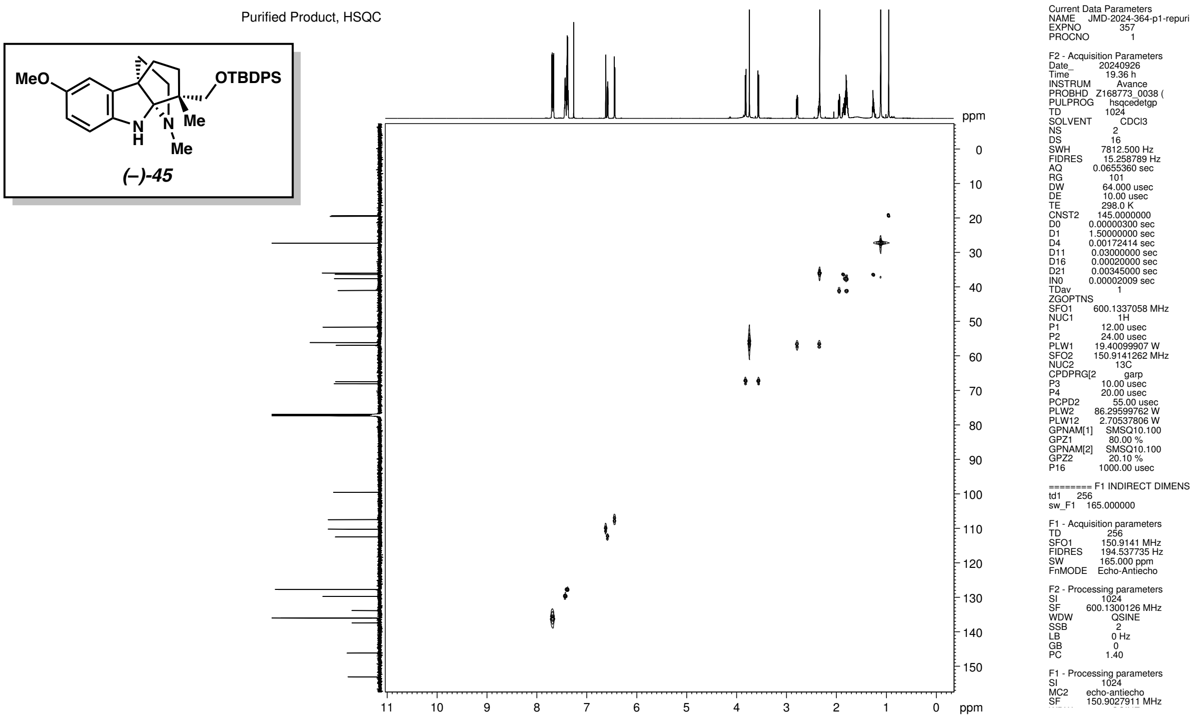

Fig. S129. HSQC spectrum of (–)-**45** in $CDCl_3$ (600 MHz).

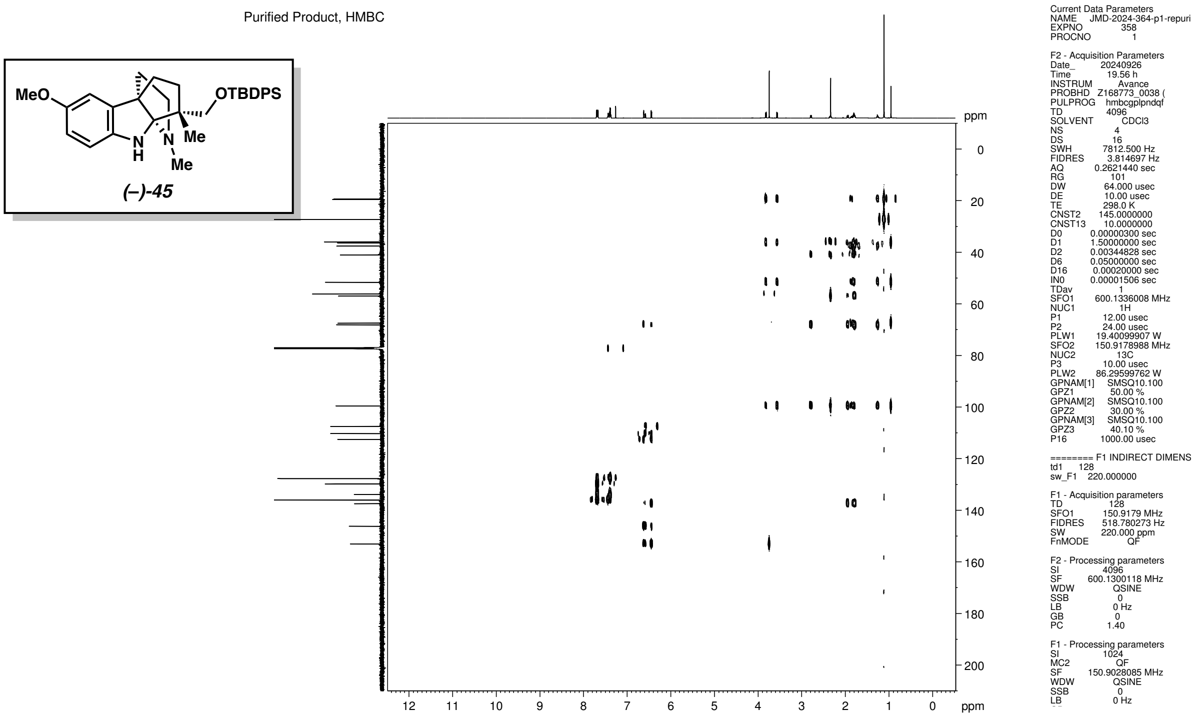

Fig. S130. HMBC spectrum of (–)-**45** in $CDCl_3$ (600 MHz).

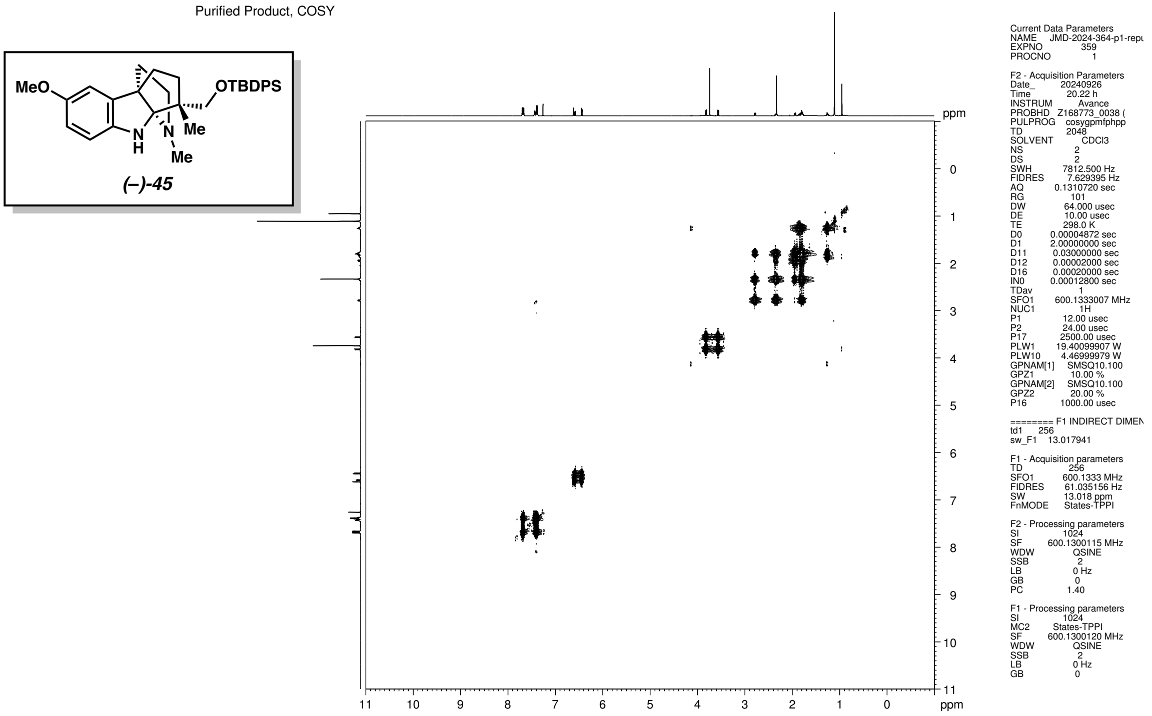

Fig. S131. ^{1}H-^{1}H COSY spectrum of (–)-**45** in $CDCl_3$ (600 MHz).

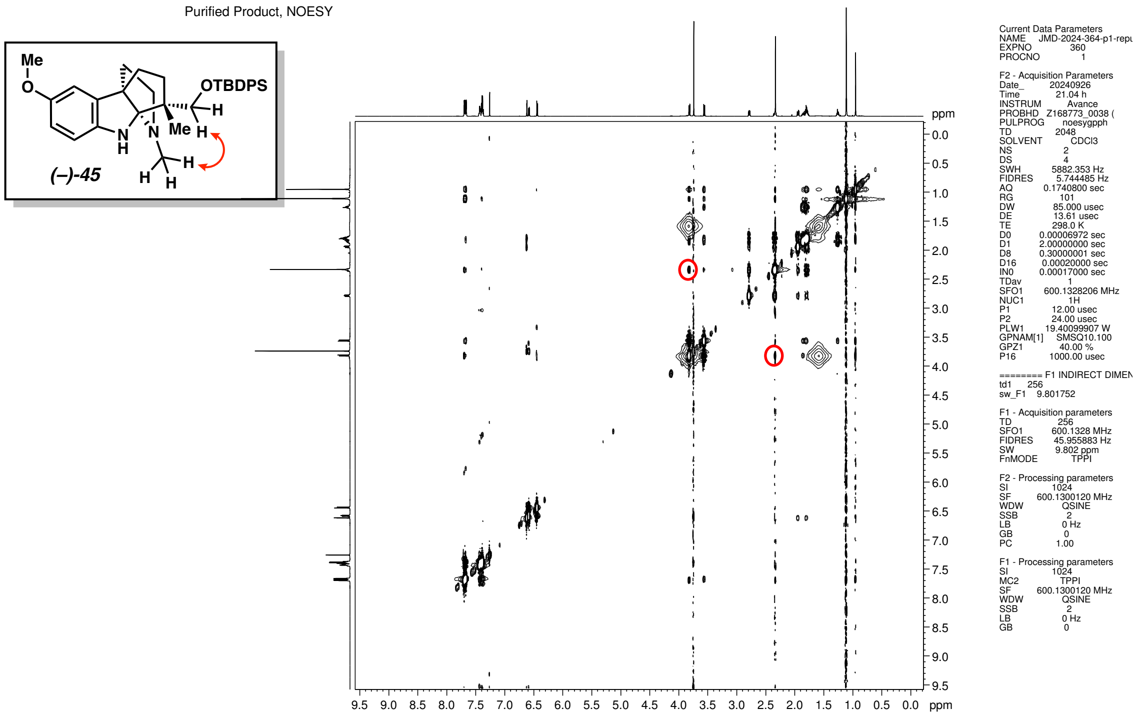

Fig. S132. NOESY spectrum of (–)-**45** in $CDCl_3$ (600 MHz).

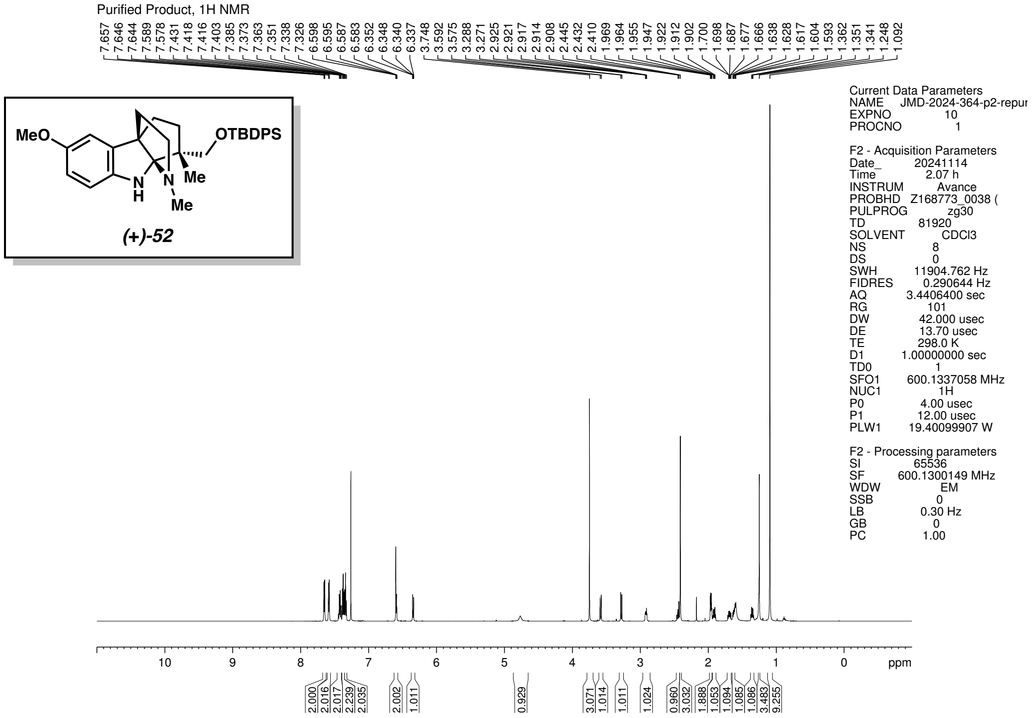

Fig. S133. ^{1}H NMR spectrum of (+)-**52** in $CDCl_3$ (600 MHz).

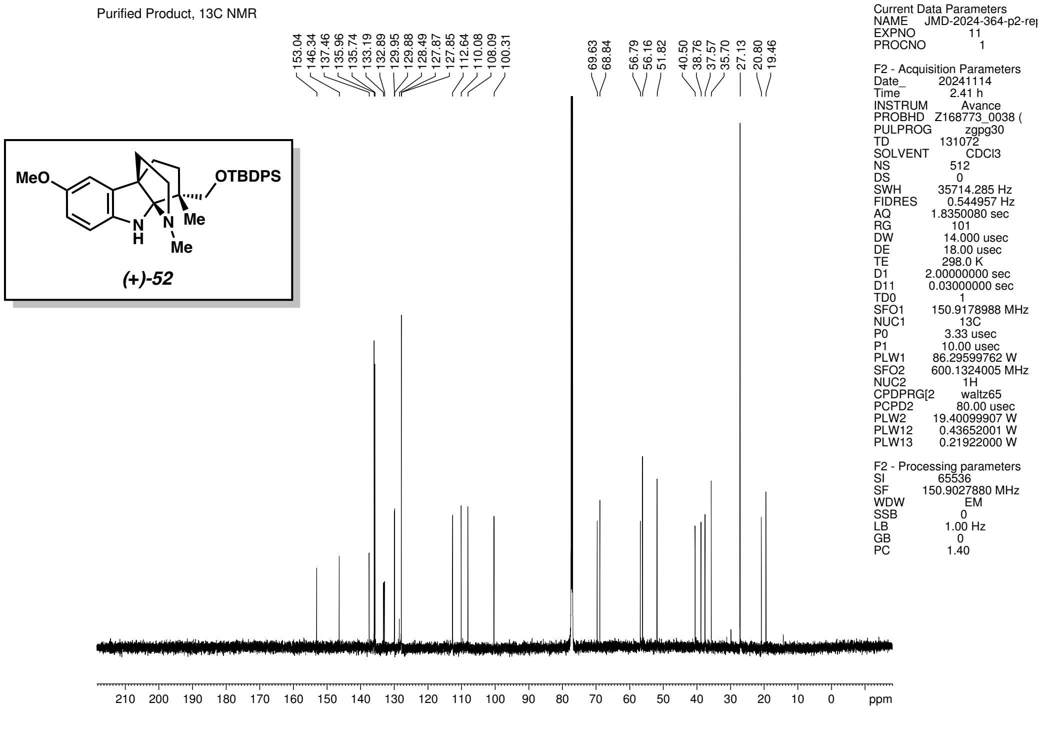

Fig. S134. ^{13}C NMR spectrum of (+)-**52** in $CDCl_3$ (151 MHz).

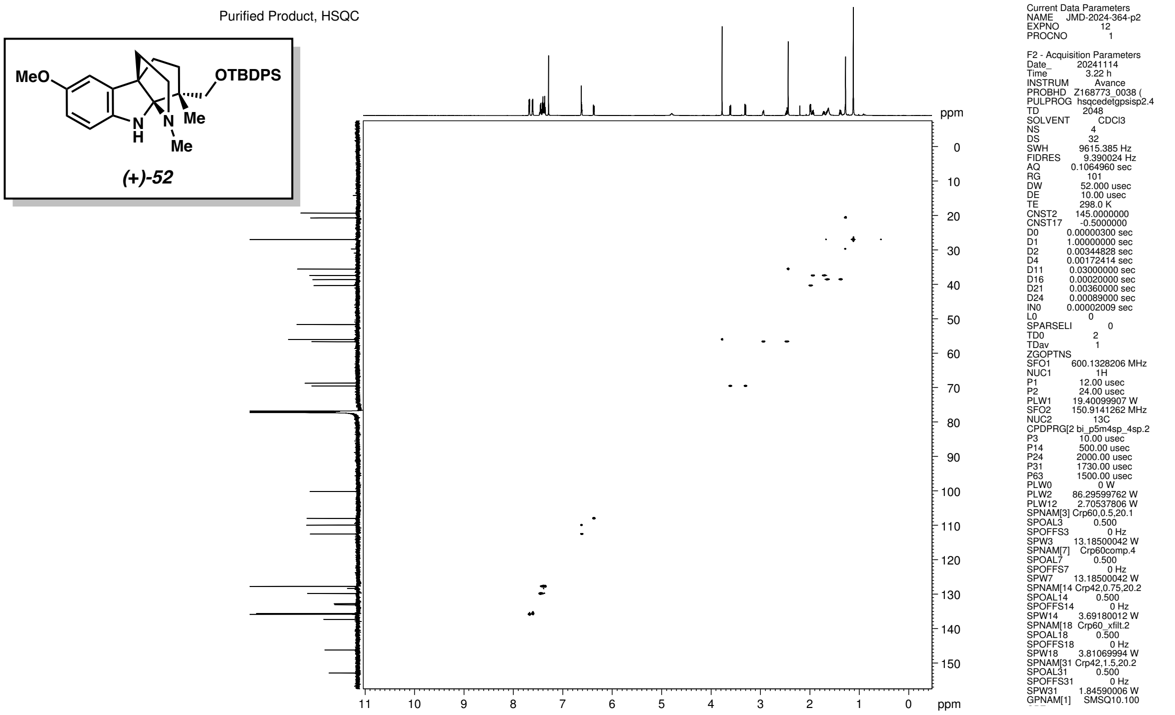

Fig. S135. HSQC spectrum of (+)-**52** in $CDCl_3$ (600 MHz).

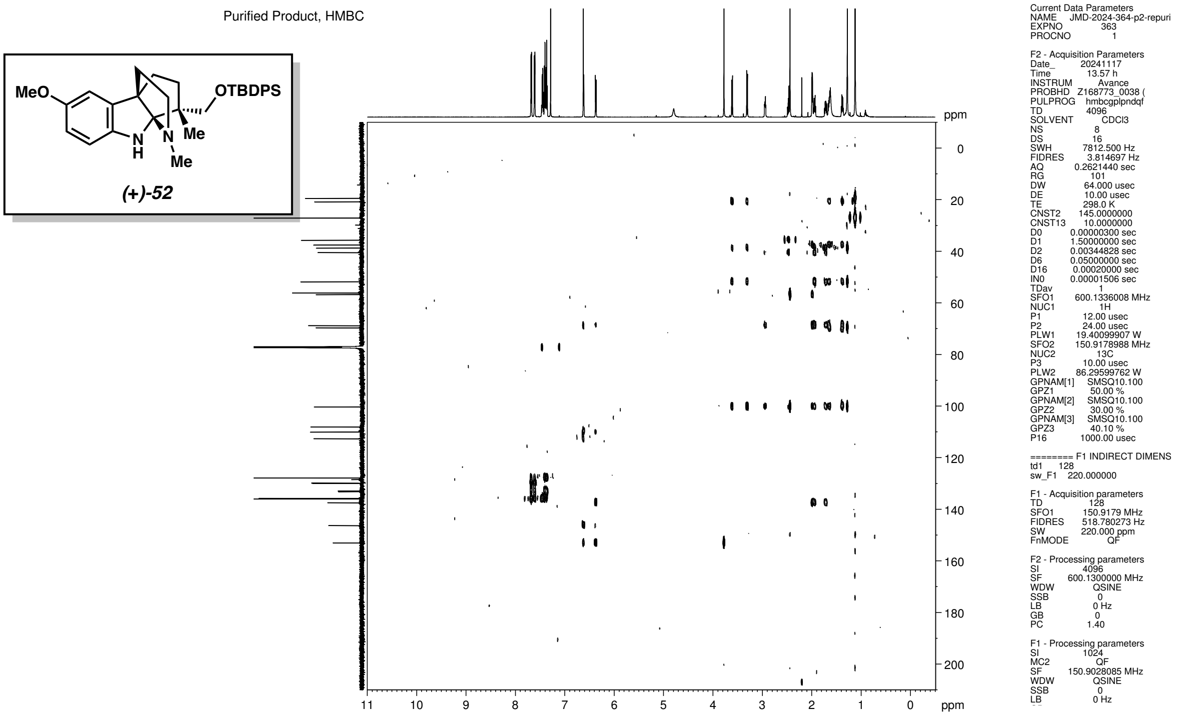

Fig. S136. HMBC spectrum of (+)-**52** in $CDCl_3$ (600 MHz).

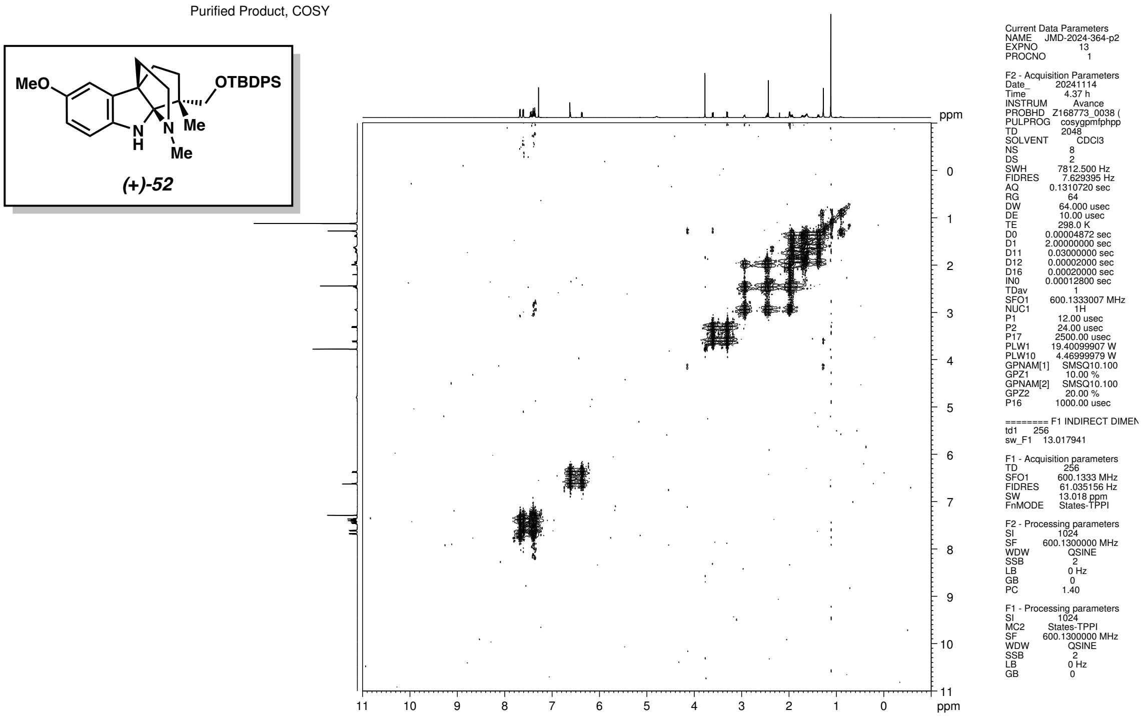

Fig. S137. ^{1}H-^{1}H COSY spectrum of (+)-**52** in $CDCl_3$ (600 MHz).

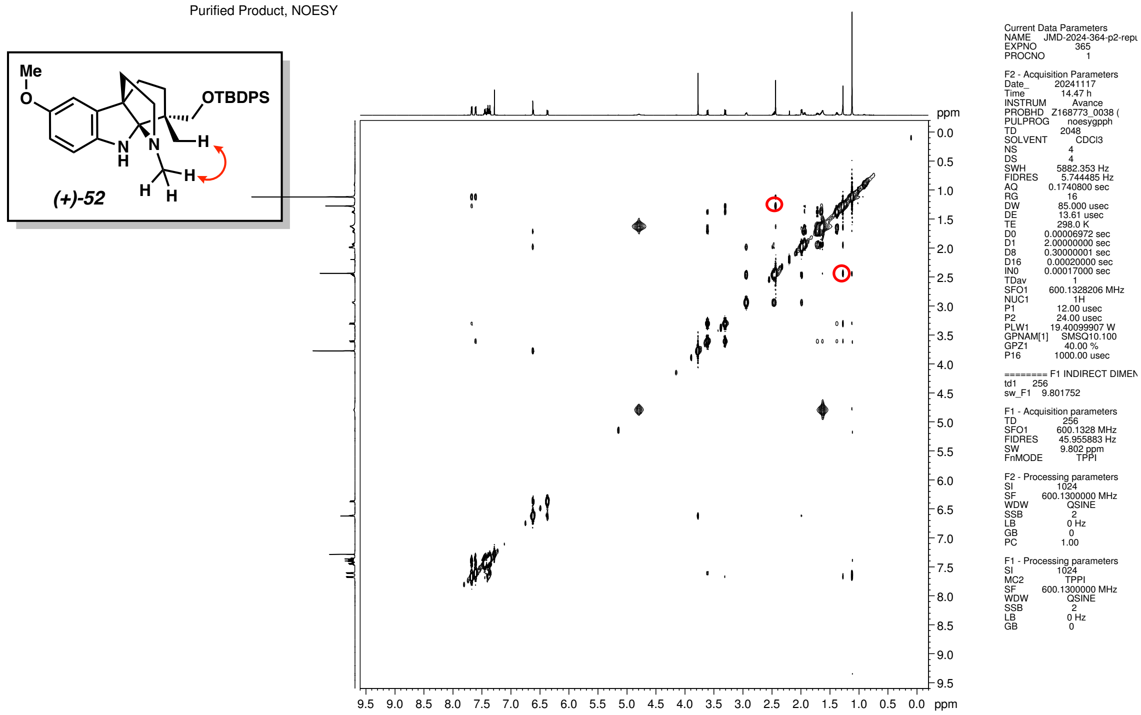

Fig. S138. NOESY spectrum of (+)-**52** in $CDCl_3$ (600 MHz).

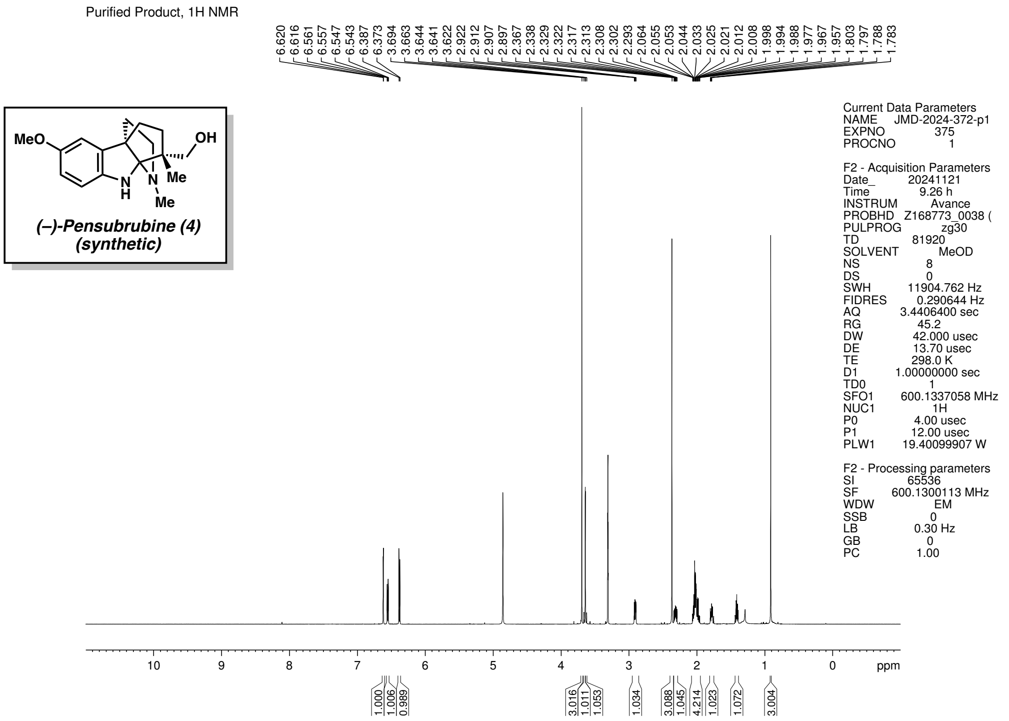

Fig. S139. ^{1}H NMR spectrum of synthetic (–)-pensubrubine (**4**) in CD_3OD (600 MHz).

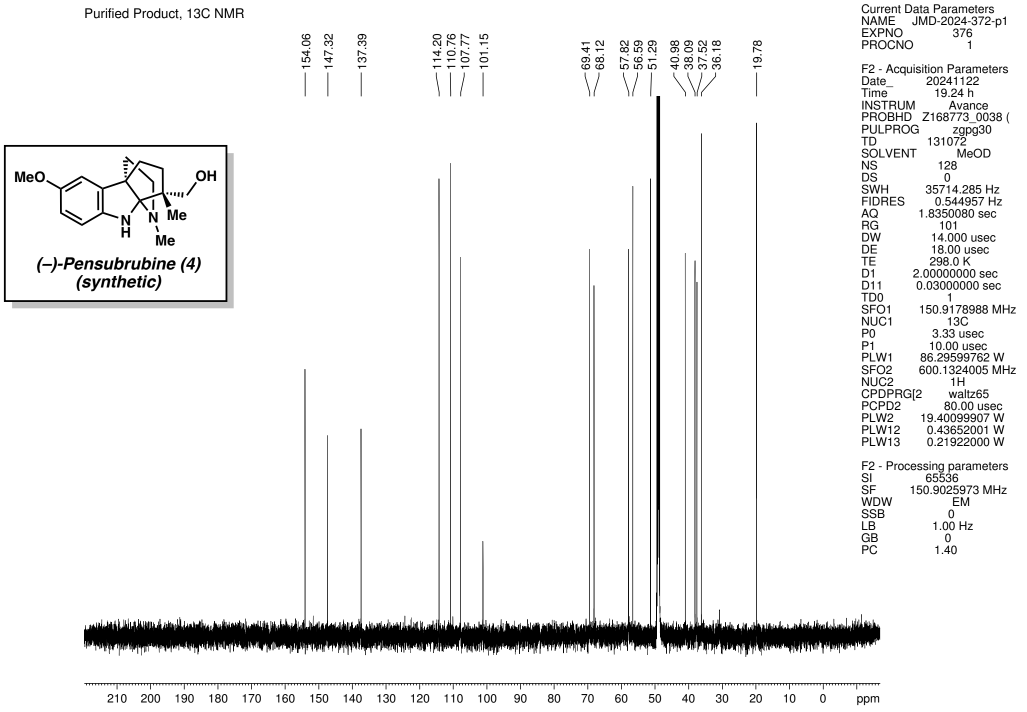

Fig. S140. ^{13}C NMR spectrum of synthetic (–)-pensubrubine (**4**) in CD_3OD (151 MHz).

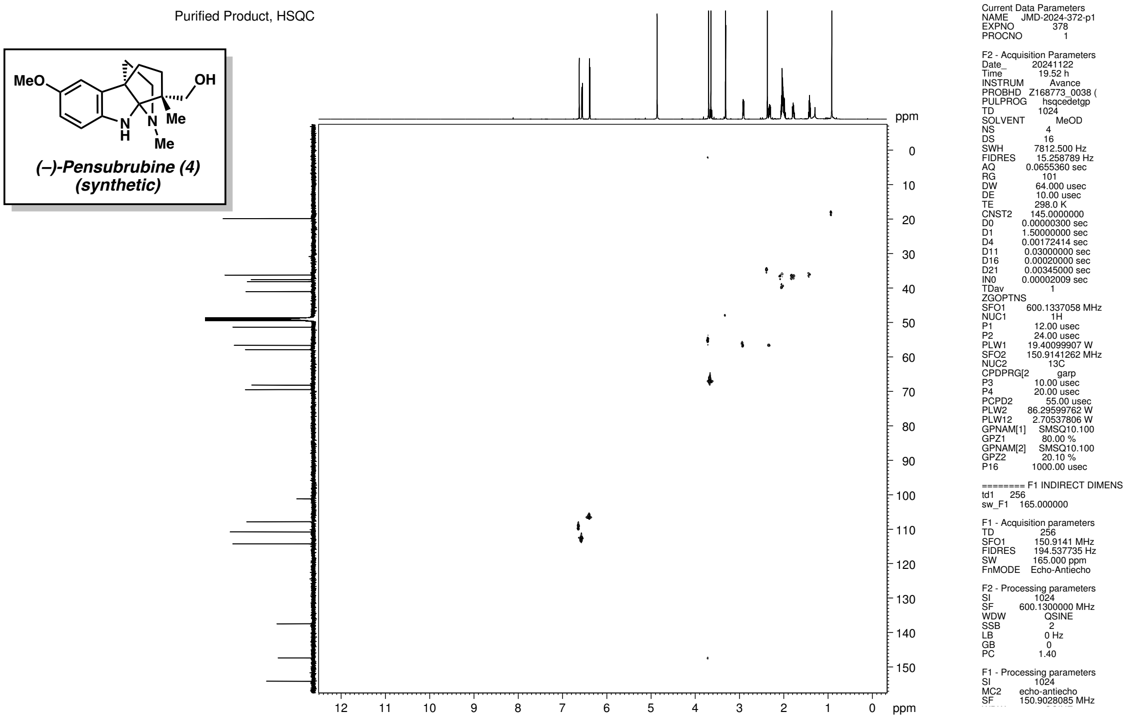

Fig. S141. HSQC spectrum of synthetic (–)-pensubrubine (**4**) in CD_3OD (600 MHz).

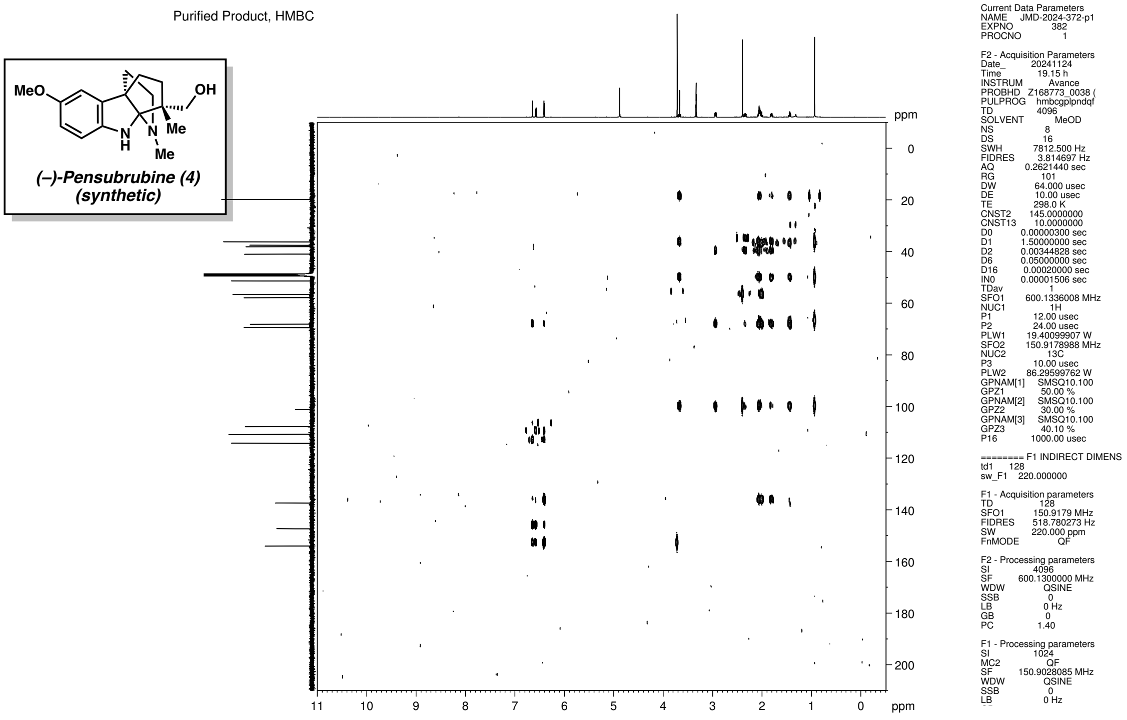

Fig. S142. HMBC spectrum of synthetic (–)-pensubrubine (**4**) in CD_3OD (600 MHz).

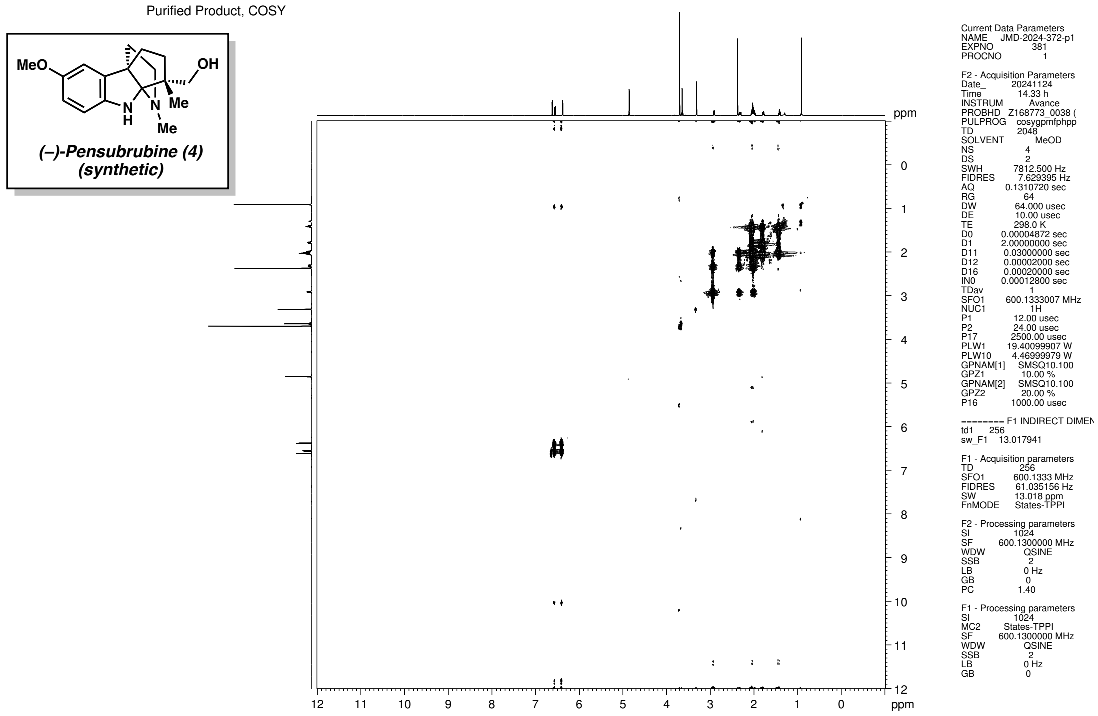

Fig. S143. ^{1}H-^{1}H COSY spectrum of synthetic (–)-pensubrubine (**4**) in CD_3OD (600 MHz).

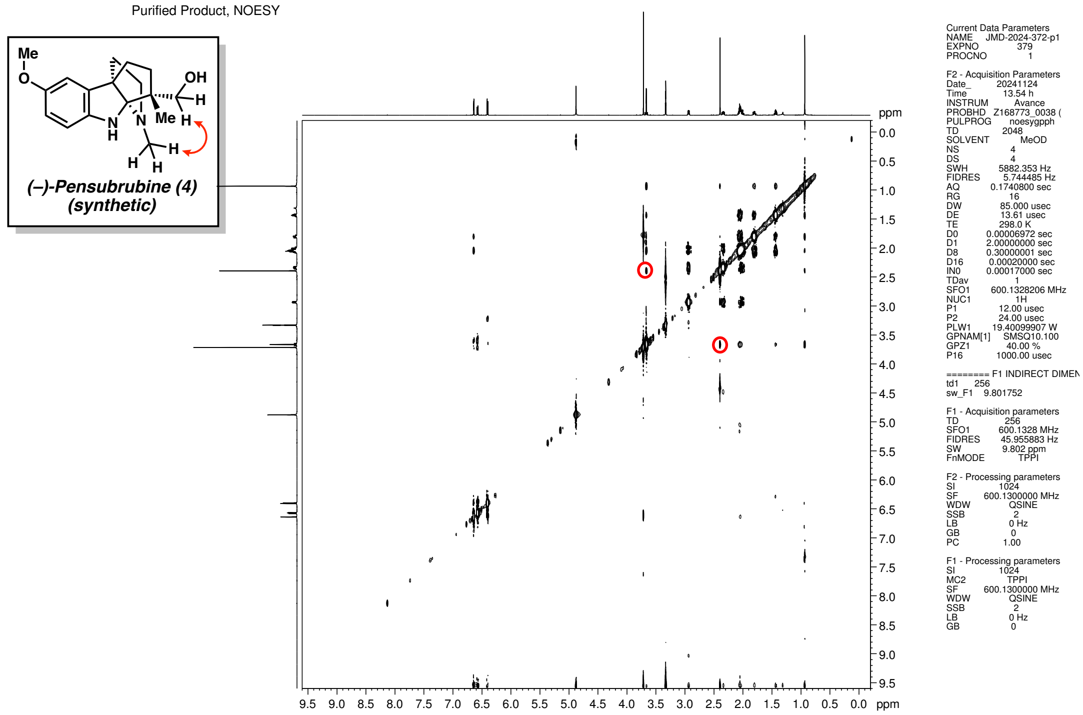

Fig. S144. NOESY spectrum of synthetic (–)-pensubrubine (**4**) in CD_3OD (600 MHz).

References

1. Schweitzer-Chaput, B., Horwitz, M. A., de Pedro Beato, E. & Melchiorre, P. Photochemical generation of radicals from alkyl electrophiles using a nucleophilic organic catalyst. *Nat. Chem.* **11**, 129–135 (2019).
2. Dey, R., Kumar, P. & Banerjee, P. Lewis acid catalyzed annulation of cyclopropane carbaldehydes and aryl hydrazines: Construction of tetrahydropyridazines and application toward a one–pot synthesis of hexahydropyrrolo[1,2-b]pyridazines. *J. Org. Chem.* **83**, 5438–5449 (2018).
3. Widen, J. C. et al. Helenalin analogues targeting NF-κB p65: Thiol reactivity and cellular potency studies of varied electrophiles. *ChemMedChem* **13**, 303–311 (2018).
4. Revol, G., Fuchs, C. & Zard, S. Z. A short formal total synthesis of (±)-hirsutic acid. *Can. J. Chem.* **90**, 927–931 (2012).
5. Ramachandran, P. V., Chen, G.-M. & Brown, H. C. Chiral synthesis via organoboranes. 42. Selective reductions. 57. Efficient kinetic resolution of representative α-tertiary ketones with *B*-chlorodiisopinocampheylborane. *J. Org. Chem.* **61**, 88 (1996).
