# Supplementary material for: Propellane Alkaloid Biosynthesis and Total Synthesis via Interrupted Reaction Pathways
Source: ACS Cent Sci. 2026 Apr 3;12(4):532–42. doi: 10.1021/acscentsci.6c00057 (PMC13107220; doi:10.1021/acscentsci.6c00057)
Supplement: Supplementary file 2 [file oc6c00057_si_002.pdf]

oc-2026-00057k.R1

Name: Peer Review Information for "Propellane alkaloid biosynthesis and total synthesis via interrupted reaction pathways"

First Round of Reviewer Comments

Reviewer: 1

Comments to the Author

The authors report a careful and interesting study on the synthesis of propellane alkaloids. The study is multifaceted in the sense that it combines molecular biology, bio-organic chemistry/natural product biosynthesis and organic synthesis including structure elucidation. This makes this manuscript fit the scope of ACS Central Science.

The study convincingly elucidates the biosynthesis of a recently isolated class of propellane alkaloids, the subrubines. Genome mining led to the reconstitution of the biosynthesis pathway and this was confirmed by a (total) synthesis of one of the products. The work is sound, well-documented and it adds to the field.

I have a number of small comments that I noted in the paper, mainly textual. I have two more major points. The first one: How is established what the structure of the major diastereomer of 44 is? Second one: The authors stress the central position of interrupted reaction pathways both in the biosynthesis and in the chemical synthesis. It is already in the title of the manuscript and used as a central " scaffold". This is in two ways somewhat problematic. It makes the paper difficult to understand (including the title) until one arrives, while reading, at the explanation of these " interruptions". I suggest the authors to reconsider this aspect. A second point is that for an organic chemist it is difficult to discriminate between the " conventional pathway" and the interrupted pathway. When does one call a reaction an interruption of another reaction? This is arguably easier in the biosynthesis. And third: the " biosynthesis interruption" has nothing to do with the " synthesis interruption" . So all in all this feels kind of twisted.

I request the authors to reflect on this, without denying the level of the work and manuscript.

Reviewer: 2

Comments to the Author

In this well-written paper, Billingley et al. report their discovery of subrubines, a new class of fungal indole alkaloids with a diaza[3.3.3]propellane group. The authors also elucidate the biosynthetic pathway of these compounds and describe their total synthesis. Inspired by the notion that interrupted chemical reactions can result in novel biochemical transformations, the authors begin by identifying enzyme candidates capable of catalyzing these reactions. First, they selected a Tyr-to-Phe mutation, which has been shown to interrupt the canonical reaction pathway in fungal ene-reductases and redirect the flow toward new products. Through genome mining, they identified a variant of the mutant reductase associated with an uncharacterized, putative biosynthetic gene cluster. Using a combination of directed mutagenesis and comparative metabolomics and genomics, they identified the metabolic products of the gene cluster. The authors then elucidated the biosynthetic pathway by fully reconstructing it in vivo in an Aspergillus nidulans heterologous host. As predicted, the mutant reductase acts in a non-canonical fashion, enabling the formation of the propellane scaffold. The authors also discovered a new type of prenyltransferase that acts on free tryptamines rather than enzyme-bound substrates and probed its substrate specificity in vitro. Finally, the authors present a synthetic route that produces the subrubine scaffold in a stereoselective manner. Notably, the authors did not take a biomimetic approach when designing their synthetic route. Rather, their strategy is based on interrupted Fischer indolization, a technique pioneered by one of the authors. This strategy is mechanistically unrelated to the interrupted ene-reduction described in the biochemical section of the article.

The findings of this study have a wide range of applications. The importance of searching for interrupted enzymatic reactions is clearly demonstrated. This will encourage the field to investigate natural mutant enzyme variants, which could lead to the discovery of non-canonical enzyme reactivities. Characterizing the fungal pathway to diaza[3.3.3]propellane indole alkaloids could lead to elucidating the biosynthesis of plant alkaloids with similar structures, many of which exhibit interesting bioactivities. The study also establishes a foundation for the synthetic and biosynthetic production of key intermediates in a stereoselective manner. Access to these intermediates will facilitate the study of

molecular targets and mechanisms of these alkaloids' activity, as well as the production of non-natural homologs.

I find the study to be well-presented and well-executed. The pathway proposal is, for the most part, well supported, and the synthetic approach is sound and clearly outlined in the main text. I have a few minor comments that I am confident the authors will address before the paper is publishable in ACS Central Science. My comments focus on the biochemical part, which is closer to my area of expertise.

Specific comments (minor changes):

1) The authors mention finding a putative subrubine gene cluster in only two organisms in the NCBI database: Penicillium subrubescens and Aspergillus terreus NIH2624. However, a BLASTp search reveals that very similar enzymes are also present in at least a third organism: Penicillium ochrochloron. Could the authors justify their omission?

2) The authors start reconstructing the biosynthetic pathway in vivo by co-expressing the first two enzymes from P. subrubescens in both Aspergillus and Saccharomyces. Despite the considerably higher product titer in Saccharomyces, the authors proceed to elucidate the remaining steps in Aspergillus. The reason for choosing this expression host is unclear to readers outside the field. Later, the authors report significant interference between the expressed pathway and the host's metabolism, which hinders their ability to assign a function to some enzymes in the subrubine cluster. Could the authors justify their choice of expression host?

3) Figure 4a shows the stepwise extension of the pathway with concurrent depletion of upstream intermediates in vivo. When SubK is added, the considerable decrease in the levels of intermediate 4 is not accompanied by the appearance of new peaks of comparable size. This is also shown in figure S29. What do the authors attribute this apparent loss in flux to? Are esters other than 1 and 3 produced in the heterologous host? Do the authors detect any new compounds upon expression of subK?

4) Figure 4b could be improved by reporting how many biological replicates were analyzed and what error bars are shown. The same applies for other figures in the

supplementary figures where quantitative data are presented. Could the authors include this information?

5) In the caption of figure S19b, the authors may have confused C3 of indole with C4 of indole. Could the authors please verify?

6) There are some inconsistencies between the NMR spectra and the corresponding tabulated data. For example, according to table S19 I should expect to find a correlation between the amide carbon C2 at 174.8 ppm and proton H1' at 2.53 ppm in the HMBC spectrum of compound 20. This correlation cannot be seen in figure S102. Inconsistencies may also be present in other spectra/table. Could the authors revise all their spectral data before resubmitting?

7) In figure S26, chromatogram iv, the authors may have written the wrong enzyme combination.

8) "Concurrent with our biosynthetic investigation, ..." The general reader would expect that the authors would use the same kind of interrupted chemical reaction in both the biochemical and synthetic part. Therefore, I was somewhat surprised that the total synthesis is mechanistically completely unrelated to the biosynthesis. I think it would be important to explain the rationale of the (diverging, non-biomimetic) synthetic route.

9) Conclusion/Discussion: Please consider presenting this work in a broader context (e.g. flavoenzymes and biocatalysts from specialized metabolism, there are also some reviews on the subject).

10) Please fix the placement of the citation marks.

11) Figure 2D and elsewhere: note that "wild-type" is the adjective; should read "wild-type name organism" or "name organism wild type".

12) Fig. 3b check table format (e.g. % better flushed right)

Reviewer: 3

Comments to the Author

See attached file with my detailed review of the manuscript

This ACS Central Science manuscript reports the discovery of a new family of fungal pyrrolidinoindoline alkaloids ("subrubines," 1–6) featuring a rare, compact diaza[3.3.3]propellane core, and it links their formation to "interrupted" reactivity both in biosynthesis and in total synthesis. The authors motivate a genome-mining strategy focused on noncanonical old yellow enzyme (OYE)-type ene-reductases bearing an active-site Tyr-to-Phe substitution (position analogous to Y196 in EasA), hypothesizing that loss of the canonical proton donor could divert an enolate intermediate toward nonstandard C–C bond formation. They identify an uncharacterized cluster (sub) in *Aspergillus terreus* (and a syntenous cluster in *Penicillium subrubescens*) encoding SubA/SubE and multiple tailoring enzymes including a ψOYE (SubF) and an NRPS module (SubK). The work then combines heterologous expression in *A. nidulans*, metabolite isolation/structure elucidation, pathway logic supported by shunt products, and a concise enantioselective total synthesis of (–)-pensubrubine (4) in seven steps, with stereochemical assignment supported by matching characterization data and explicit claims of unambiguous absolute stereochemistry evidence.

Overall importance and fit for ACS Central Science are strong. The manuscript is not simply a new natural product report, it advances a generalizable concept for discovery: mining enzyme "active-site logic" (a specific catalytic residue substitution) as a beacon for noncanonical biochemistry, then using that to access both new molecules and new transformations. The combination of (i) discovery of structurally unusual alkaloids, (ii) credible biosynthetic pathway reconstruction through genetics/heterologous expression and shunt-product logic, and (iii) parallel development of an efficient total synthesis that reinforces stereochemical assignments, makes the work more compelling than any single component alone.

Overall, the originality is high but may be overstated (see below as a major concern). The diaza[3.3.3]propellane pyrrolidinoindoline core is explicitly positioned as rare in nature and challenging synthetically, with limited prior biosynthetic understanding and few chemical methods available, and the authors' "interrupted reaction" framing provides a unifying conceptual advance across bio- and chemical synthesis. The genome-mining angle is also differentiating: rather than mining for scaffold enzymes alone (PKS/NRPS/TS), the study mines for a specific catalytic "signature" likely to cause reactivity divergence (Tyr→Phe at Y196-equivalent), then ties that signature to a new cluster and new chemistry.

Technical quality and reproducibility are generally good, with a few places where the manuscript would benefit from stronger causal evidence and clearer boundaries between data and inference.

Structure elucidation rigor (strength, with one suggested enhancement): The SI indicates conventional, appropriate analytical coverage for new natural products, including 1D and 2D NMR (explicitly described), HRMS, UV, and optical rotation measurements. The main text also points to NOESY for a key stereochemical feature ((Z)-prenyl in 22). This is largely consistent with community expectations for natural product structure elucidation. One improvement would be to make the minimum "structure proof package" explicit in the main text (even if spectra remain in SI): for each new subrubine, state clearly which 2D experiments were used to establish connectivity (HSQC/HMBC/COSY) and which were used to establish relative configuration (NOESY/ROESY), and clarify how absolute configuration was determined for each compound (especially those not covered by total synthesis correlation).

Biosynthetic causality for the key SubF step (main area to strengthen): The central claim is that SubF (a ψOYE with the Tyr→Phe substitution) catalyzes an interrupted ene-reduction culminating in a reductive Mannich cyclization that builds the propellane core. The pathway logic is plausible and the shunt products are informative, but the mechanistic attribution would be more convincing with at least one additional direct line of evidence. Specific suggestions:

a) Include an in vitro reconstitution focused narrowly on the SubF transformation using the best available substrate surrogate (e.g., chemically prepared enal 24 or a close analog) and purified SubF, showing formation of the cyclized product (or a trapped intermediate) vs. canonical reduction products.

b) Leverage the built-in hypothesis around the active-site residue: the SI indicates construction of an F196Y mutation in subF (primer “subF_F196Y” is listed), implying the authors have the tools to test residue-function relationships. Demonstrating that restoring Tyr shifts product outcome toward conventional OYE behavior (or otherwise attenuates cyclization) would directly connect the mining logic to biochemical function, and would elevate the mechanistic credibility significantly.
c) Consider isotope labeling (e.g., deuteride delivery from NAD(P)H or solvent D2O) to support the proposed sequence of hydride addition and lack of protonation, and/or to distinguish competing mechanistic possibilities for C–C bond formation.

Where computational modeling is used, add experimental anchors: The SI mentions use of AlphaFold/AlphaFill/SwissDock logic to rationalize SubE prenylation route selection (Path B favored) based on distances and active-site residues. This is fine as hypothesis-generation, but the paper should be careful not to overstate mechanistic certainty from docking/model placement. A simple mutational test (single residue predicted to influence the route) or product outcome changes with substrate analogs would help keep the mechanistic narrative rigorous.

Reporting clarity for biological relevance: The manuscript frames pyrrolidinoindoline alkaloids as broadly bioactive, but the results emphasize chemistry and biosynthesis more than biological evaluation. ACS Central Science does not require in vivo efficacy, but the paper would benefit from either (i) a concise initial bioactivity screen (even limited panels) to position the molecules biologically, or (ii) a clear statement that biological profiling is outside scope and will be reported separately. Right now, readers may expect some functional data given the natural product framing.

Major Concern:

The manuscript presents the concept of “interrupted reactions” as a unifying theme linking the SubF-catalyzed biosynthetic transformation and the interrupted Fischer indolization employed in total synthesis. While this framing is rhetorically compelling, I have reservations about whether the term “interrupted” reflects a fundamentally new mechanistic paradigm or instead describes a well-established phenomenon of enzymatic plasticity and altered intermediate fate in natural product biosynthesis (e.g. well-known examples include “abortive” or “shunt” cyclization, enolate diversion in PKS and □-branching tailoring steps, terpene cyclases producing alternative carbocation trajectories, and enzymes that suppress canonical quenching pathways to name a few).

In the proposed SubF mechanism, hydride addition to an enal generates an enolate that, rather than undergoing canonical protonation (as in typical OYE chemistry), proceeds to intramolecular C–C bond formation. Conceptually, this represents divergence in enolate fate driven by altered activesite functionality (Tyr→Phe substitution). However, examples of enzyme-mediated diversion of reactive intermediates, particularly enolates and carbocations are abundant throughout biosynthesis . Enzymes frequently suppress canonical quenching pathways and redirect intermediates toward alternative bond-forming events. As currently presented, it is not fully clear what distinguishes this case mechanistically from broader principles of catalytic divergence and pathway evolution.

The manuscript would benefit from clarifying whether "interrupted reactions" is intended as:

1. A descriptive metaphor linking biosynthetic and synthetic strategy, or
2. A predictive and mechanistically distinct principle grounded in experimentally validated catalytic control.

If the latter is intended, stronger causal evidence directly connecting the Tyr→Phe substitution to suppression of protonation and promotion of C–C bond formation would significantly strengthen the claim. For example, demonstration that reverting Phe back to Tyr alters product outcome, or that SubF can be shown in vitro to favor cyclization over canonical reduction, would elevate the conceptual advance beyond reframing known enzymatic plasticity.

Absent such mechanistic validation, the authors are encouraged to moderate the framing and more explicitly situate this chemistry within the broader context of enzyme-enabled intermediate diversion in natural product biosynthesis. Doing so would not diminish the significance of the discovery but would sharpen the conceptual claims and prevent overstatement of novelty.

Recommendation: moderate revision where the authors add at least one direct SubF functional test, ideally the F196Y "revertant" experiment. The work is novel, conceptually interesting, and technically solid in most respects. The main risk is that the manuscript's headline mechanistic claim (SubF-catalyzed reductive Mannich cyclization enabled by Tyr→Phe) reads stronger than the direct causal evidence currently shown in the experiments. Strengthening

that single point would materially improve rigor, elevate impact, and pre-empt predictable reviewer skepticism about whether the cyclization is unequivocally enzyme-enabled versus inferred from pathway logic.

Author's Response to Peer Review Comments:

Reply to reviewer comments are in blue.

Changes to text are shown in "*blue italics*" in quotations.

Reviewer 1

Recommendation: Publish in ACS Central Science after minor revisions noted.

1. How is established what the structure of the major diastereomer of 44 is?

We appreciate the reviewer's careful observation. The relative stereochemistry of major diastereomer **44** is proposed based on the assigned structure of the major product in the subsequent step (i.e. **45**). We assumed the C3 and C3' quaternary stereocenters would not undergo epimerization under the given reaction conditions.

We have added the following sentence to the manuscript text to clarify this point.

Page 13: "*The C3 and C3' stereocenters of the major epimer (as depicted) was assigned based on NOESY analysis of the subsequent intermediate in our synthesis (i.e., **45**) based on the assumption that both of these quaternary stereocenters were not prone to epimerization.*"

2. The authors stress the central position of interrupted reaction pathways both in the biosynthesis and in the chemical synthesis. It is already in the title of the manuscript and used as a central "scaffold". This is in two ways somewhat problematic. It makes the paper difficult to understand (including the title) until one arrives, while reading, at the explanation of these "interruptions". I suggest the authors to reconsider this aspect. A second point is that for an organic chemist it is difficult to discriminate between the "conventional pathway" and the interrupted pathway. When does one call a reaction an interruption of another reaction? This is arguably easier in the biosynthesis. And third: the "biosynthesis interruption" has nothing to do with the "synthesis interruption". So all in all this feels kind of twisted.

I request the authors to reflect on this, without denying the level of the work and manuscript.

We thank the reviewer for this helpful perspective. As the reviewer notes, the use of interrupted reaction paths is essential to how this manuscript was assembled (title, abstract, main text, etc)

partially because we find this to be an interesting concept that is perhaps not widely appreciated throughout organic chemistry.

The key resource we are relying on is Ref 1 by Yudin and coworkers (*Nat. Rev. Chem*. **2021**, **5**, 604–623), where the authors highlight that "interrupted" is widely used, but has not been well defined. They highlight all of the following as known uses of the terminology: "an intermediate undergoing the conventional pathway is redirected to a more favourable alternative route; the conventional pathway is no longer available, which compels the intermediate to take on a new route; the reaction takes on a new pathway that diverges from the reactant; an intermediate in the conventional pathway is trapped owing to a change in reaction conditions; pericyclic reactions that proceed stepwise through non-concerted bond-breaking and bond-forming events."

Thus, the use of "interrupted" is appropriate in our manuscript, but we recognize that we should have included the actual definitions in our manuscript. We have now edited the introduction accordingly and hope this improves the clarity. Specifically, we have included the following text:

"*As reviewed by Yudin and co-workers, several types of interrupted processes exist including: a) pathways in which an intermediate is redirected from a conventional pathway to a more favorable alternative, b) scenarios where a given intermediate takes on a new route owing to a conventional pathway no longer being available, c) new pathways that simply diverge from a reactant, and d) processes in which a change in reaction conditions leads to an intermediate in a conventional pathway undergoing a different reaction outcome*."

Additionally, we agree that the biosynthesis and chemical synthesis "interruptions" are different and have revised our text to specify this. We hope this minimizes confusion for the reader, since both approaches still meet the "interrupted" definitions. The specific changes are as follows:

Abstract: adjusted to specify that the approaches use "*distinct interrupted reaction pathways*"

Introduction: adjusted to specify that the synthetic route "*relies on a mechanistically-distinct interrupted process (i.e., interrupted Fischer indolization)*".

Total synthesis discussion: added sentence to specify the distinction: "*Additionally, the interrupted Fischer indolization approach is mechanistically-distinct from the previously discussed biosynthesis, which renders it an effective complementary tactic for accessing the subrubine natural products*."

Conclusion: similarly adjusted to highlight the "*two distinct interrupted processes in the bio- and total synthesis of subrubine alkaloids*".

Reviewer 2

Recommendation: Publish in ACS Central Science after minor revisions noted.

1) The authors mention finding a putative subrubine gene cluster in only two organisms in the NCBI database: Penicillium subrubescens and Aspergillus terreus NIH2624. However, a BLASTp search reveals that very similar enzymes are also present in at least a third organism: Penicillium ochrochloron. Could the authors justify their omission?

We thank the reviewer for this helpful note. Indeed, a third syntenous subrubine gene cluster from *Penicillium ochrochloron* appeared in the NCBI Database in November 2024 after our bioinformatic analysis was performed. We are grateful for this observation and have modified the following sentence in order to clarify omission of this gene cluster from our analysis:

"*The remaining uncharacterized BGC (sub) is present in the widely studied Aspergillus terreus NIH2624 (Fig. 2c), with a syntenous BGC found in just two other organism in the NCBI database, Penicillium subrubescens and Penicillium ochrochloron.*"

2) The authors start reconstructing the biosynthetic pathway in vivo by co-expressing the first two enzymes from P. subrubescens in both Aspergillus and Saccharomyces. Despite the considerably higher product titer in Saccharomyces, the authors proceed to elucidate the remaining steps in Aspergillus. The reason for choosing this expression host is unclear to readers outside the field. Later, the authors report significant interference between the expressed pathway and the host's metabolism, which hinders their ability to assign a function to some enzymes in the subrubine cluster. Could the authors justify their choice of expression host.

We thank the reviewer for this insightful recommendation. Reconstitution of the pathway beyond the first two enzymes was attempted in yeast using pre-spliced cDNA isolated from *A. nidulans*. We observed neither depletion of pathway intermediates nor accumulation of downstream products. Standard approaches to improve P450 function, including *P. subrubescens* CPR coexpression, media optimization, vector copy number tuning, and expression of *A. terreus* homologs, proved unsuccessful.

In order to clarify for readers outside of the field, we have added the following:

Complete heterologous reconstitution section: "*To determine the next steps in diaza[3.3.3]propellane formation, the P450-encoding genes (subB, subG, and subJ) were individually coexpressed alongside subAE in both S. cerevisiae and A. nidulans. Only coexpression of subB in A. nidulans led to the depletion of* ***15*** *with the concomitant appearance of the oxindole* ***20*** *as confirmed by NMR analysis (Figs. S20, S98–S102, and Table S19). Accordingly, A. nidulans was chosen as the heterologous host for the remaining reconstitution experiments.*"

3) Figure 4a shows the stepwise extension of the pathway with concurrent depletion of upstream intermediates in vivo. When SubK is added, the considerable decrease in the levels of intermediate 4 is not accompanied by the appearance of new peaks of comparable size. This is also shown in figure S29. What do the authors attribute this apparent loss in flux to? Are esters other than 1 and 3 produced in the heterologous host? Do the authors detect any new compounds upon expression of subK?

We thank the reviewer for this question. We were equally intrigued by the apparent shift in metabolic flux upon expression of *subK*. One hypothesis noted in the paper – "the most likely explanation is upregulated detoxification pathways due to yet undetermined biological activities of subrubine esters." The reviewer's comment motivated us to revisit our *A. nidulans* strains expressing the complete subrubine pathway. We inspected LC-MS chromatograms for a specific exact mass shared by all subrubine esters. Indeed, subrubine A and subrubine C were the only two compounds to emerge bearing this characteristic mass fragment, indicating **1** and **3** are the only esters produced upon introduction of *subK*.

4) Figure 4b could be improved by reporting how many biological replicates were analyzed and what error bars are shown. The same applies for other figures in the supplementary figures where quantitative data are presented. Could the authors include this information?

We thank the reviewer for this helpful comment. We have added the following description to Fig. 4b and Fig. S10:

"*Error bars denote standard deviation across n = 3 biological replicates*." We

have also added the following description to Fig. S18:

"*Error bars denote standard deviation across n = 3 technical replicates*."

5) In the caption of figure S19b, the authors may have confused C3 of indole with C4 of indole. Could the authors please verify?

We thank the reviewer for this helpful observation. We have updated the caption of Fig. 19b to reflect this correction.

6) There are some inconsistencies between the NMR spectra and the corresponding tabulated data. For example, according to table S19 I should expect to find a correlation between the amide carbon C2 at 174.8 ppm and proton H1' at 2.53 ppm in the HMBC spectrum of compound 20. This correlation cannot be seen in figure S102. Inconsistencies may also be present in other spectra/table. Could the authors revise all their spectral data before resubmitting?

We thank the reviewer for this helpful observation. The chemical shift of C2 in Compound 20 is 182.7 ppm, not 174.8 ppm; we have corrected this value in Table S20. Indeed, the corresponding HMBC correlation may be observed in Fig. SS102.

We have carefully re-examined all NMR tables and spectra and have ensured full consistency across the datasets.

7) In figure S26, chromatogram iv, the authors may have written the wrong enzyme combination.

We thank the reviewer for this helpful observation. We have updated the label of Fig. S26, chromatogram iv to read "*subAEBGH*" accordingly.

8) "Concurrent with our biosynthetic investigation, …" The general reader would expect that the authors would use the same kind of interrupted chemical reaction in both the biochemical and synthetic part. Therefore, I was somewhat surprised that the total synthesis is mechanistically completely unrelated to the biosynthesis. I think it would be important to explain the rationale of the (diverging, non-biomimetic) synthetic route.

We realize the confusion and have removed the text "Concurrent with our biosynthetic investigation, …" (both in the manuscript main text and conclusion).

In addition, as noted in our response to Reviewer 1, our revised manuscript includes the definitions of interrupted processes and highlights throughout that the biosynthesis and chemical synthesis interrupted pathways are mechanistically distinct. Additionally, the section on our total synthesis, we explain that the Fischer approach is an "effective complementary tactic".

9) Conclusion/Discussion: Please consider presenting this work in a broader context (e.g. flavoenzymes and biocatalysts from specialized metabolism, there are also some reviews on the subject).

We appreciate the reviewer's insightful comment. We have added the following sentence to our conclusion and cited the reviews by Walsh/Wencewicz and Liu:

"*This C–C bond formation strategy underscores the versatility of two-electron reaction manifolds accessed by flavoenzymes in biosynthetic pathways*.[27,28]"

10) Please fix the placement of the citation marks.

We thank the reviewer for the feedback. We have confirmed that all citation placements and References are consistent with ACS Central Science style.

11) Figure 2D and elsewhere: note that "wild-type" is the adjective; should read "wild-type name organism" or "name organism wild type".

We thank the reviewer for identifying this error. We have corrected the spelling in Fig. 2d, Fig. S7, as well as throughout the manuscript and SI.

12) Fig. 3b check table format (e.g. % better flushed right)

We thank the reviewer for the recommendation. We have updated the alignment of the conversions presented in Fig. 3b to enhance readability.

Reviewer 3

Recommendation: Major revisions required.

Comments:

Technical quality and reproducibility are generally good, with a few places where the manuscript would benefit from stronger causal evidence and clearer boundaries between data and inference.

Structure elucidation rigor (strength, with one suggested enhancement): The SI indicates conventional, appropriate analytical coverage for new natural products, including 1D and 2D NMR (explicitly described), HRMS, UV, and optical rotation measurements. The main text also points to NOESY for a key stereochemical feature ((Z)-prenyl in 22). This is largely consistent with community expectations for natural product structure elucidation. One improvement would be to make the minimum "structure proof package" explicit in the main text (even if spectra remain in SI): for each new subrubine, state clearly which 2D experiments were used to establish connectivity (HSQC/HMBC/COSY) and which were used to establish relative configuration (NOESY/ROESY), and clarify how absolute configuration was determined for each compound (especially those not covered by total synthesis correlation).

We thank the reviewer for the insightful perspective. In order to improve the explicitness of structural determination for all subrubines, we have added the following sentence to the main text:

"*2D NMR experimentation was used to establish connectivities (HSQC/HMBC/COSY) and relative configurations (NOESY/ROESY) of all newly described subrubines, with absolute configuration of the C3–C2–C3' stereotriad ultimately deduced by enantioselective total synthesis of (–)-**4**.*"

Biosynthetic causality for the key SubF step (main area to strengthen): The central claim is that SubF (a ψOYE with the Tyr→Phe substitution) catalyzes an interrupted ene-reduction culminating in a reductive Mannich cyclization that builds the propellane core. The pathway logic is plausible and the shunt products are informative, but the mechanistic attribution would be more convincing with at least one additional direct line of evidence. Specific suggestions: a) Include an in vitro reconstitution focused narrowly on the SubF transformation using the best available substrate surrogate (e.g., chemically prepared enal 24 or a close analog) and purified SubF, showing formation of the cyclized product (or a trapped intermediate) vs. canonical reduction products. b) Leverage the built-in hypothesis around the active-site residue: the SI indicates construction of an F196Y mutation in subF (primer "subF_F196Y" is listed), implying the authors have the tools to test residue-function relationships. Demonstrating that restoring Tyr shifts product outcome toward conventional OYE behavior (or otherwise attenuates cyclization) would directly connect the mining logic to biochemical function, and would elevate the mechanistic credibility significantly.

c) Consider isotope labeling (e.g., deuteride delivery from NAD(P)H or solvent D2O) to support the proposed sequence of hydride addition and lack of protonation, and/or to distinguish competing mechanistic possibilities for C–C bond formation.

Please see our response to this below in the "major concern" section.

Where computational modeling is used, add experimental anchors: The SI mentions use of AlphaFold/AlphaFill/SwissDock logic to rationalize SubE prenylation route selection (Path B favored) based on distances and active-site residues. This is fine as hypothesis-generation, but the paper should be careful not to overstate mechanistic certainty from docking/model placement. A simple mutational test (single residue predicted to influence the route) or product outcome changes with substrate analogs would help keep the mechanistic narrative rigorous.

We thank the reviewer for the helpful comment. We agree that computational modelling should be used for hypothesis generation and experimental substantiation, and that as previously written, the mechanistic certainty in the Fig. S19 caption is overstated. We have updated the caption as follows:

"*Fig. S19. Comparison of DMATS active sites and potential mechanism of prenylation. a, Crystal structure of FgaPT2, dimethylallyltryptophan synthase, complexed with L-tryptophan and DMASPP is shown. Two routes to dimethylallyltryptophan were previously identified by Tanner. Based on active site geometry, Path A and Path B were deemed equally plausible; mutational studies have provided evidence that Path B involving a Cope rearrangement is the favored route. b, AlphaFold model of SubE was predicted; ligands were placed using AlphaFill and SwissDock. Two potential routes to* ***15*** *are shown. The longer distance (6.7 Å) from indole C3 to DMAPP C1 and absence of the active site base (I178 SubE vs. K174 in FgaPT2) suggest that Path B may be favored, a possibility that warrants further investigation.*"

Reporting clarity for biological relevance: The manuscript frames pyrrolidinoindoline alkaloids as broadly bioactive, but the results emphasize chemistry and biosynthesis more than biological evaluation. ACS Central Science does not require in vivo efficacy, but the paper would benefit from either (i) a concise initial bioactivity screen (even limited panels) to position the molecules biologically, or (ii) a clear statement that biological profiling is outside scope and will be reported separately. Right now, readers may expect some functional data given the natural product framing.

We thank the reviewer for this thoughtful recommendation. We agree that establishing the biological context of the subrubine alkaloids will further strengthen their significance. A comprehensive biological evaluation lies beyond the scope of the present study, which focuses on elucidating their biosynthesis and enabling synthetic access to these compounds. As such, we have added the following sentence to the conclusion:

"Together, these complementary approaches provide reliable access to subrubines and related analogues, setting the stage for future investigations into the biological function of this unique family of alkaloids."

Major Concern:

The manuscript presents the concept of "interrupted reactions" as a unifying theme linking the SubF-catalyzed biosynthetic transformation and the interrupted Fischer indolization employed in total synthesis. While this framing is rhetorically compelling, I have reservations about whether

the term “interrupted” reflects a fundamentally new mechanistic paradigm or instead describes a well-established phenomenon of enzymatic plasticity and altered intermediate fate in natural product biosynthesis (e.g. well-known examples include “abortive” or “shunt” cyclization, enolate diversion in PKS and -branching tailoring steps, terpene cyclases producing alternative carbocation trajectories, and enzymes that suppress canonical quenching pathways to name a few). In the proposed SubF mechanism, hydride addition to an enal generates an enolate that, rather than undergoing canonical protonation (as in typical OYE chemistry), proceeds to intramolecular C–C bond formation. Conceptually, this represents divergence in enolate fate driven by altered active-site functionality (Tyr→Phe substitution). However, examples of enzyme-mediated diversion of reactive intermediates, particularly enolates and carbocations are abundant throughout biosynthesis. Enzymes frequently suppress canonical quenching pathways and redirect intermediates toward alternative bond-forming events. As currently presented, it is not fully clear what distinguishes this case mechanistically from broader principles of catalytic divergence and pathway evolution.

The manuscript would benefit from clarifying whether “interrupted reactions” is intended as:

1. A descriptive metaphor linking biosynthetic and synthetic strategy, or
2. A predictive and mechanistically distinct principle grounded in experimentally validated catalytic control.

If the latter is intended, stronger causal evidence directly connecting the Tyr→Phe substitution to suppression of protonation and promotion of C–C bond formation would significantly strengthen the claim. For example, demonstration that reverting Phe back to Tyr alters product outcome, or that SubF can be shown in vitro to favor cyclization over canonical reduction, would elevate the conceptual advance beyond reframing known enzymatic plasticity.

Absent such mechanistic validation, the authors are encouraged to moderate the framing and more explicitly situate this chemistry within the broader context of enzyme-enabled intermediate diversion in natural product biosynthesis. Doing so would not diminish the significance of the discovery but would sharpen the conceptual claims and prevent overstatement of novelty.

We greatly appreciate this feedback, which are shared by Reviewers 1 and 2. As described in our responses to those reviewers, we now have included definitions of interrupted reactions (based on Yudin’s review) and adjusted language throughout to make it more clear that the interrupted pathways for the biosynthesis and chemical synthesis are distinct and complementary.

Further to this reviewer’s point, we are using “interrupted reactions” here as an umbrella term to cover both biosynthetic and synthetic access to the molecules. We agree that the SubF example fits within the broader landscape of enzymatic intermediate diversion, rather than representing a new mechanistic paradigm, and we realize that our introductory language was perhaps overstating. We have adjusted the early text to now say the following:

"Interrupted enzymatic pathways are also common, wherein active site mutations reroute canonical intermediates to generate novel structures[2–7] or, more broadly, any scenario in which a biosynthetic intermediate is redirected from a more conventional pathway."

Also, in the section on 'Discovery and Isolation of Subrubines', we removed the word 'rare' while discussing interrupted enzymatic reactions. We have also added this sentence: "As noted earlier, interrupted enzymatic reactions are common if one considers a broad definition in which a biosynthetic intermediate is redirected from a more conventional pathway. One elegant example..."

In summary, we agree with the reviewer's wise feedback and feel the changes we have made accordingly greatly improve our manuscript.

Recommendation: moderate revision where the authors add at least one direct SubF functional test, ideally the F196Y "revertant" experiment. The work is novel, conceptually interesting, and technically solid in most respects. The main risk is that the manuscript's headline mechanistic claim (SubF-catalyzed reductive Mannich cyclization enabled by Tyr→Phe) reads stronger than the direct causal evidence currently shown in the experiments. Strengthening that single point would materially improve rigor, elevate impact, and pre-empt predictable reviewer skepticism about whether the cyclization is unequivocally enzyme-enabled versus inferred from pathway logic.

We greatly appreciate the reviewer's helpful comment. We realize that as previously written, we implied an explicit mechanistic link between the removal of the tyrosine proton and the new functionality (as was the case in the carbocyclization report by Breinbauer). The F196Y "revertant" experiment described by Reviewer 3 was performed. We believe Reviewer 3 is correct to point out that this functional test should be included, and agree that readers will be particularly interested in characterization of SubF.

Though we were unable to isolate or synthesize the proposed SubF substrate or a corresponding precursor/analog that would have enabled *in vitro* analysis, we were able to successfully perform the *in vivo* revertant experiment. These results, as described below, confirm that the Tyr→Phe mutation is not required for cyclization activity, and that reversion back to tyrosine decreases total subrubine produced by less than 10%. This decrease was not accompanied by any apparent increase in reduced shunt products, suggesting the F196Y "revertant" of SubF does not favor a canonical reduction reaction. We agree with Reviewer 3's comment that the proposed SubF mechanism is an example of active site plasticity and agree that the observed Mannich reaction is not "enabled" by the Y196F substitution, but instead other active site residues that reroute the canonical enolate intermate towards cyclization. Accordingly, SubF contains an active site mutation *signaling* a noncanonical function, and meets Yudin's definition of an "interrupted reaction" in which a biosynthetic intermediate is redirected from a more conventional pathway.

We have adjusted Fig. 4b and updated the corresponding SI Tables. We also added the following text to describe the F196Y revertant experiment:

"*We next turned to in vivo mutagenesis of SubF in A. nidulans to evaluate the role of the active site F196 (F176 in SubF) in propellane formation. Notably, reversion of the Phe back to Tyr resulted in only a small decrease (<10%) in the amount of total* **6** *produced in the heterologous host (Fig. 4b), with no apparent formation of ene-reduced shunt products. Therefore, the propellane synthase activity of SubF can be attributed to broader active site residues that reroute the canonical enolate intermediate towards an intramolecular Mannich cyclization. This is consistent with prior work by Cheng et al., in which the corresponding Tyr to Phe mutation in EasA retained significant isomerase activity.*[5] *Thus, while presence of F196 serves as a useful genome-mining signature, it is not strictly required for noncanonical functionality in SubF.*"

We also added the following text in the genome mining section to clarify OYE residue numbering as well as the origin of the Y196F designation:

"Replacement of this tyrosine with phenylalanine (designated Y196F in the model yeast enereductase) was shown to decrease the canonical reaction rate by six orders of magnitude."

Additionally, we have made the following changes to reflect the conclusion that the Mannich cyclization is not enabled by the Y196F mutation:

Moderated the third sentence in the abstract: "*We demonstrate through complete pathway reconstitution that the putatively annotated ene-reductase SubF functions as the propellane synthase that directs an enolate intermediate towards an intramolecular Mannich cyclization*."

Updated the mechanistic description in the conclusion: "*The key biosynthetic step in the formation of subrubine alkaloids is an interrupted ene-reduction catalyzed by the noncanonical enereductase SubF, that reroutes the enolate intermediate from the conventional protonation pathway to undergo a reductive Mannich cyclization*."

Additional comments from Reviewer 3 provided in attached file:

Page 2, Line 11: for the reader who has not started reading yet this in unclear

We thank the reviewer for this insightful comment. We edited the introduction so that the definition utilized by Yudin is introduced as early as possible, in hopes that this improves the clarity. Specifically, we have included the following text:

"*As reviewed by Yudin and co-workers, several types of interrupted processes exist including: (a) pathways in which an intermediate is redirected from a conventional pathway to a more favorable alternative, (b) scenarios where a given intermediate takes on a new route owing to a conventional pathway no longer being available, (c) new pathways that simply diverges from a reactant, and (d) processes in which a change in reaction conditions leads to an intermediate in a conventional pathway undergoing a different reaction outcome*."

Page 2, Line 14: what does "each" refer to here?

We thank the reviewer for the comment and have removed the word "*each*" in order to improve clarity.

Page 2, Line 30: I would write "Fischer indole synthesis"

We thank the reviewer for the comment. We use the phrase "*interrupted Fischer indolization*" to match the terminology that is broadly used in the literature.

Page 3, Line 12: it is not so clear to me what the original enzymatic sequence is, if the current one is interrupted

We thank the reviewer for the comment. We have clarified what is meant by the "specific original enzymatic sequence" by updating this sentence as follows:

"*We subsequently elucidate the biosynthesis of these natural products, uncovering an interrupted ene-reduction that results in an unusual reductive Mannich cyclization to build the azapropellane core.*"

Page 12, Line 3: this paragraph carefully explains the interrupted Fischer indole reaction. Maybe a bit too extensive, because as such the interrupted Fisher indole synthesis is (well) known, at least to the synthetic chemist, and also the scheme is clear. So consider condensing this paragraph

We thank the reviewer for the recommendation. We anticipate that this manuscript will be of interest to readers with a biochemistry background who may be unfamiliar with the interrupted Fischer indole reaction, and included this level of detail for clarity.

Page 13, Line 6: How is this established

We appreciate the reviewer's careful observation. As described in our response to Reviewer 1, the relative stereochemistry of major diastereomer **44** is proposed based on the assigned structure of the major product in the subsequent step (i.e. **45**). We assumed the C3 and C3' quaternary stereocenters would not undergo epimerization under the given reaction conditions.

We have added the following sentence to the manuscript text to clarify this point.

Page 13: "*The C3 and C3' stereocenters of the major epimer (as depicted) were assigned based on NOESY analysis of the subsequent intermediate in our synthesis (i.e.,* ***45****) based on the assumption that both of these quaternary stereocenters were not prone to epimerization*."

Page 13, Line 50: In my opinion, the use of the scaffold "interrupted reactions" is a bit far-fetched. There is no clear link between the biosynthesis and the chemical synthesis there, and it is very difficult for the reader to follow the interruption aspect, also because the reader needs to know what is actually interrupted (once this is made clear it becomes more easy). So I would trim down this "interruption" aspect

We thank the reviewer for this helpful perspective. As both Review 1 and Reviewer 3 note, the use of interrupted reaction paths is essential to how this manuscript was assembled (title, abstract,

main text, etc) partially because we find this to be an interesting concept that is perhaps not widely appreciated throughout organic chemistry. We also agree that the biosynthesis and chemical synthesis “interruptions” are different and have revised our text to specify this. We hope this minimizes confusion for the reader, since both approaches still meet the “interrupted” definitions. The specific changes are as follows:

Abstract: adjusted to specify that the approaches use “*distinct interrupted reaction pathways*”

Introduction: adjusted to specify that the synthetic route “*relies on a mechanistically-distinct interrupted process (i.e., interrupted Fischer indolization)”.*

Total synthesis discussion: added sentence to specify the distinction: “*Additionally, the interrupted Fischer indolization approach is mechanistically-distinct from the previously discussed biosynthesis, which renders it an effective complementary tactic for accessing the subrubine natural products*.”

Conclusion: similarly adjusted to highlight the “*pivotal role of two distinct interrupted processes in the bio- and total synthesis of subrubine alkaloids*” as well as removal of the final sentence to de-emphasize the relationship between the biosynthesis and total synthesis.

oc-2026-00057k.R2

Name: Peer Review Information for "Propellane alkaloid biosynthesis and total synthesis via interrupted reaction pathways"

Second Round of Reviewer Comments

Reviewer: 2

Comments to the Author

The authors have fully addressed all of my points. The paper can now be published as is.

Author's Response to Peer Review Comments:

We have changed cover option to NO.
